# Global, regional, and national burden of suicide, 1990–2021: a systematic analysis for the Global Burden of Disease Study 2021

**DOI:** 10.1016/S2468-2667(25)00006-4

**Published:** 2025-02-19

**Authors:** GBD 2021 Suicide Collaborators, Nicole Davis Weaver, Gregory J Bertolacci, Emily Rosenblad, Sama Ghoba, Matthew Cunningham, Kevin S Ikuta, Madeline E Moberg, Vincent Mougin, Chieh Han, Eve E Wool, Yohannes Habtegiorgis Abate, Habeeb Omoponle Adewuyi, Qorinah Estiningtyas Sakilah Adnani, Leticia Akua Adzigbli, Aanuoluwapo Adeyimika Afolabi, Suneth Buddhika Agampodi, Bright Opoku Ahinkorah, Aqeel Ahmad, Danish Ahmad, Sajjad Ahmad, Ayman Ahmed, Haroon Ahmed, Hanadi Al Hamad, Yazan Al-Ajlouni, Rasmieh Mustafa Al-amer, Mohammed Albashtawy, Wafa A Aldhaleei, Syed Shujait Ali, Waad Ali, Mahmoud A Alomari, Mohammed A Alsabri, Nelson Alvis-Guzman, Yaser Mohammed Al-Worafi, Alireza Amindarolzarbi, Sohrab Amiri, Tudorel Andrei, Saeid Anvari, Jalal Arabloo, Demelash Areda, Anton A Artamonov, Tahira Ashraf, Seyyed Shamsadin Athari, Maha Moh'd Wahbi Atout, Ahmed Y Azzam, Ashish D Badiye, Nayereh Baghcheghi, Saeed Bahramian, Maciej Banach, Suzanne Lyn Barker-Collo, Till Winfried Bärnighausen, Amadou Barrow, Azadeh Bashiri, Hameed Akande Bashiru, Mohammad-Mahdi Bastan, Kavita Batra, Ravi Batra, Mohsen Bayati, Corina Benjet, Habib Benzian, Paola Bertuccio, Akshaya Srikanth Bhagavathula, Priyadarshini Bhattacharjee, Corey B Bills, Sri Harsha Boppana, Guilherme Borges, Hamed Borhany, Yasser Bustanji, Florentino Luciano Caetano dos Santos, Giulio Castelpietra, Arthur Caye, Muthia Cenderadewi, Rama Mohan Chandika, Eeshwar K Chandrasekar, Periklis Charalampous, Yifan Chen, Ritesh Chimoriya, Hitesh Chopra, Sonali Gajanan Choudhari, Dinh-Toi Chu, Isaac Sunday Chukwu, Muhammad Chutiyami, Richard G Cowden, Berihun Assefa Dachew, Omid Dadras, Xiaochen Dai, Koustuv Dalal, Lalit Dandona, Rakhi Dandona, Samuel Demissie Darcho, Reza Darvishi Cheshmeh Soltani, Claudio Alberto Dávila-Cervantes, Alejandro de la Torre-Luque, Shayom Debopadhaya, Louisa Degenhardt, Ivan Delgado-Enciso, Emina Dervišević, Michael J Diaz, Deepa Dongarwar, Ojas Prakashbhai Doshi, Haneil Larson Dsouza, Samuel C Dumith, Senbagam Duraisamy, Ejemai Eboreime, Ferry Efendi, Michael Ekholuenetale, Rabie Adel El Arab, Muhammed Elhadi, Gihan ELNahas, Chadi Eltaha, Syed Emdadul Emdadul Haque, Sharareh Eskandarieh, Ayesha Fahim, Andre Faro, Ali Fatehizadeh, Patrick Fazeli, Alireza Feizkhah, Ginenus Fekadu, Nuno Ferreira, Florian Fischer, Richard Charles Franklin, Ni Kadek Yuni Fridayani, Márió Gajdács, Aravind P Gandhi, Balasankar Ganesan, Miglas Welay Gebregergis, Mesfin Gebrehiwot, Teferi Gebru Gebremeskel, Molla Getie, Delaram J Ghadimi, Khalid Yaser Ghailan, Ahmad Ghashghaee, Ali Gholamrezanezhad, Pouya Goleij, Ayman Grada, Michal Grivna, Shi-Yang Guan, Snigdha Gulati, Sapna Gupta, Reyna Alma Gutiérrez, Roberth Steven Gutiérrez-Murillo, Erin B Hamilton, Nasrin Hanifi, Ikramul Hasan, Mahgol Sadat Hassan Zadeh Tabatabaei, Simon I Hay, Mohammad Heidari, Mehdi Hemmati, Nguyen Quoc Hoan, Mehdi Hosseinzadeh, Sorin Hostiuc, Junjie Huang, Hong-Han Huynh, Segun Emmanuel Ibitoye, Olayinka Stephen Ilesanmi, Irena M Ilic, Milena D Ilic, Mustapha Immurana, Arit Inok, Chidozie Declan Iwu, Haitham Jahrami, Sanobar Jaka, Reza Jalilzadeh Yengejeh, Zixiang Ji, Shuai Jin, Nitin Joseph, Charity Ehimwenma Joshua, Jacek Jerzy Jozwiak, Zubair Kabir, Vidya Kadashetti, Kehinde Kazeem Kanmodi, Rami S Kantar, Neeti Kapoor, Ibraheem M Karaye, Shilpi Karmakar, Harkiran Kaur, Jessica A Kerr, Himanshu Khajuria, Ajmal Khan, Khaled Khatab, Khalid A Kheirallah, Kwanghyun Kim, Min Seo Kim, Shivakumar KM KM Shivakumar, Ali-Asghar Kolahi, Hamid Reza Koohestani, Varun Krishna, Nuworza Kugbey, Mukhtar Kulimbet, G Anil Kumar, Manasi Kumar, Satyajit Kundu, Ville Kytö, Iván Landires, Nhi Huu Hanh Le, Doo Woong Lee, Wei-Chen Lee, Yo Han Lee, Stephen S Lim, Jialing Lin, Richard T Liu, José Francisco López-Gil, Giancarlo Lucchetti, Zheng Feei Ma, Venkatesh Maled, Kashish Malhotra, Ahmad Azam Malik, Agustina Maria Marconi, Ramon Martinez-Piedra, Roy Rillera Marzo, Yasith Mathangasinghe, Pallab K Maulik, Hadush Negash Meles, Ritesh G Menezes, Tuomo J Meretoja, Tomislav Mestrovic, Irmina Maria Michalek, Ted R Miller, Moonis Mirza, Awoke Misganaw, Chaitanya Mittal, Abdalla Z Mohamed, Nouh Saad Mohamed, Abdollah Mohammadian-Hafshejani, Ali H Mokdad, Sabrina Molinaro, Lorenzo Monasta, AmirAli Moodi Ghalibaf, Shane Douglas Morrison, Rohith Motappa, Faraz Mughal, Francesk Mulita, Yanjinlkham Munkhsaikhan, Christopher J L Murray, Sathish Muthu, Woojae Myung, Ayoub Nafei, Pirouz Naghavi, Ganesh R Naik, Gurudatta Naik, Zuhair S Natto, Muhammad Naveed, Shadan Navid, Biswa Prakash Nayak, Athare Nazri-Panjaki, Henok Biresaw Netsere, Sudan Prasad Neupane, Hoang Anh Hoang Nguyen, Nhien Ngoc Y Nguyen, Phat Tuan Nguyen, Phuong The Nguyen, Van Thanh Nguyen, Ali Nikoobar, Isabel Noguer, Shuhei Nomura, Chisom Adaobi Nri-Ezedi, Virginia Nuñez-Samudio, Ogochukwu Janet Nzoputam, Bogdan Oancea, Michael Safo Oduro, In-Hwan Oh, Sylvester Reuben Okeke, Yinka Doris Oluwafemi, Sok King Ong, Michal Ordak, Heather M Orpana, Esteban Ortiz-Prado, Uchechukwu Levi Osuagwu, Alicia Padron-Monedero, Jagadish Rao Padubidri, Raul Felipe Palma-Alvarez, Anamika Pandey, Ashok Pandey, Ioannis Pantazopoulos, Seoyeon Park, Sungchul Park, Ava Pashaei, Jay Patel, Shrikant Pawar, Prince Peprah, Mario F P Peres, Ionela-Roxana Petcu, Anil K Philip, Michael R Phillips, Zahra Zahid Piracha, Jalandhar Pradhan, Elton Junio Sady Prates, Dimas Ria Angga Pribadi, Jagadeesh Puvvula, Ibrahim Qattea, Gangzhen Qian, Venkatraman Radhakrishnan, Pankaja Raghav, Sarvenaz Rahimibarghani, Afarin Rahimi-Movaghar, Vafa Rahimi-Movaghar, Md. Mosfequr Rahman, Mosiur Rahman, Muhammad Aziz Rahman, Mohammad Rahmanian, Pushp Lata Rajpoot, Mahmoud Mohammed Ramadan, Shakthi Kumaran Ramasamy, Smitha Rani, Mithun Rao, Sowmya J Rao, Mohammad-Mahdi Rashidi, Prateek Rastogi, Devarajan Rathish, David Laith Rawaf, Lennart Reifels, Mohsen Rezaeian, Taeho Gregory Rhee, Jennifer Rickard, Leonardo Roever, Moustaq Karim Khan Rony, Chandan S N, Basema Ahmad Saddik, Farideh Sadeghian, Mohammad Reza Saeb, Umar Saeed, Sahar Saeedi Moghaddam, Mehdi Safari, Dominic Sagoe, Narjes Saheb Sharif-Askari, Pragyan Monalisa Sahoo, Soumya Swaroop Sahoo, Payman Salamati, Dauda Salihu, Sohrab Salimi, Giovanni A Salum, Sonia Sameen, Abdallah M Samy, Milena M Santric-Milicevic, Chinmoy Sarkar, Gargi Sachin Sarode, Sachin C Sarode, Brijesh Sathian, Austin E Schumacher, Mario Šekerija, Mohammad H Semreen, Sadaf G Sepanlou, Mahan Shafie, Samiah Shahid, Ahmed Shaikh, Masood Ali Shaikh, Amin Sharifan, Javad Sharifi Rad, Anupam Sharma, Vishal Sharma, Rahim Ali Sheikhi, Mahabalesh Shetty, Pavanchand H Shetty, Premalatha K Shetty, Velizar Shivarov, Sina Shool, Paramdeep Singh, Puneetpal Singh, Surjit Singh, Bogdan Socea, Dan J Stein, Murray B Stein, Jing Sun, Chandan Kumar Swain, Lukasz Szarpak, Sree Sudha T Y, Seyyed Mohammad Tabatabaei, Celine Tabche, Minale Tareke, Mohamad-Hani Temsah, Chern Choong Thum, Tenaw Yimer Tiruye, Marcos Roberto Tovani-Palone, Nghia Minh Tran, Thang Huu Tran, Nguyen Tran Minh Duc, Samuel Joseph Tromans, Thien Tan Tri Tai Truyen, Guesh Mebrahtom Tsegay, Munkhtuya Tumurkhuu, Sanaz Vahdati, Asokan Govindaraj Vaithinathan, Pascual R Valdez, Tommi Juhani Vasankari, Massimiliano Veroux, Georgios-Ioannis Verras, Manish Vinayak, Theo Vos, Mandaras Tariku Walde, Yanzhong Wang, Joseph L L Ward, Nuwan Darshana Wickramasinghe, Marcin W Wojewodzic, Renjulal Yesodharan, Arzu Yiğit, Dehui Yin, Paul Yip, Dong Keon Yon, Naohiro Yonemoto, Chuanhua Yu, Iman Zare, Mohammed G M Zeariya, Haijun Zhang, Claire Chenwen Zhong, Bin Zhu, Abzal Zhumagaliuly, Mohsen Naghavi

## Abstract

**Background:**

Deaths from suicide are a tragic yet preventable cause of mortality. Quantifying the burden of suicide to understand its geographical distribution, temporal trends, and variation by age and sex is an essential step in suicide prevention. We aimed to present a comprehensive set of global, regional, and national estimates of suicide burden.

**Methods:**

We produced estimates of the number of deaths and age-standardised mortality rates of suicide globally, regionally, and for 204 countries and territories from 1990 to 2021, and disaggregated these results by age and sex. The Global Burden of Diseases, Injuries, and Risk Factors Study (GBD) 2021 estimates of deaths attributable to suicide were broken down into two comprehensive categories: those by firearms and those by other specified means. For this analysis, we also produced estimates of mean age at the time of death from suicide, incidence of suicide attempts compared with deaths, and age-standardised rates of suicide by firearm. We acquired data from vital registration, verbal autopsy, and mortality surveillance that included 23 782 study-location-years of data from GBD 2021. Point estimates were calculated from the average of 1000 randomly selected possible values of deaths from suicide by age, sex, and geographical location. 95% uncertainty intervals (UIs) were derived from the 2·5th and 97·5th percentiles from a 1000-draw distribution.

**Findings:**

Globally, 746 000 deaths (95% UI 692 000–800 000) from suicide occurred in 2021, including 519 000 deaths (485 000–556 000) among males and 227 000 (200 000–255 000) among females. The age-standardised mortality rate has declined over time, from 14·9 deaths (12·8–15·7) per 100 000 population in 1990 to 9·0 (8·3–9·6) per 100 000 in 2021. Regionally, mortality rates due to suicide were highest in eastern Europe (19·2 [17·5–20·8] per 100 000), southern sub-Saharan Africa (16·1 [14·0–18·3] per 100 000), and central sub-Saharan Africa (14·4 [11·0–19·1] per 100 000). The mean age at which individuals died from suicide progressively increased during the study period. For males, the mean age at death by suicide in 1990 was 43·0 years (38·0–45·8), increasing to 47·0 years (43·5–50·6) in 2021. For females, it was 41·9 years (30·9–46·7) in 1990 and 46·9 years (41·2–52·8) in 2021. The incidence of suicide attempts requiring medical care was consistently higher at the regional level for females than for males. The number of deaths by suicide using firearms was higher for males than for females, and substantially varied by country and region. The countries with the highest age-standardised rate of suicides attributable to firearms in 2021 were the USA, Uruguay, and Venezuela.

**Interpretation:**

Deaths from suicide remain variable by age and sex and across geographical locations, although population mortality rates have continued to improve globally since the 1990s. This study presents, for the first time in GBD, a quantification of the mean age at the time of suicide death, alongside comprehensive estimates of the burden of suicide throughout the world. These analyses will help guide future approaches to reduce suicide mortality that consider a public health framework for prevention.

**Funding:**

Bill & Melinda Gates Foundation.

## Introduction

Suicide is increasingly recognised as an urgent public health issue.^1^, ^2^, ^3^, ^4^, ^5^ Efforts to initiate suicide prevention from a public health perspective have been motivated by WHO's Thirteenth General Programme of Work^4^ and Comprehensive Mental Health Action Plan,^6^ along with the UN's inclusion in the Sustainable Development Goals of target 3.4.2 to reduce suicide mortality.^7^ With recognition by the UN and WHO that suicide is a public health issue, the international discussion on suicide is beginning to acknowledge that suicide often arises from environmental, contextual, social, and biological factors, and must be addressed through the full spectrum of universal, targeted, and indicated public health interventions.^5^, ^8^

Suicide is a complex phenomenon with relationships to various multifaceted issues.^9^ Individuals who are living with a mental disorder have increased rates of suicide, although the magnitude of this relationship varies based on the population and study methodology.^10^, ^11^, ^12^ Substance use and substance use disorders are also associated with an increased risk of suicide.^13^ Victims of interpersonal violence, intimate partner violence, sexual violence, and childhood trauma have a substantial increase in likelihood of suicidality.^14^, ^15^, ^16^, ^17^ Social isolation has become increasingly recognised as an important risk factor for suicide.^18^ Ease of access to lethal means, such as firearms and pesticides, is associated with higher rates of suicide, and restricting access to lethal means can be an effective suicide prevention intervention.^19^, ^20^, ^21^ Poverty and social deprivation are also associated with suicide.^22^, ^23^ The combination of these risk factors—mental disorders, substance use, trauma or violence, social and cultural factors, ease of access to lethal means, poverty, and deprivation^10^, ^14^, ^18^, ^19^, ^22^, ^24^—are all important potential contributors to global suicide incidence.

**Research in context**

**Evidence before this study**

The Sustainable Development Goals have established a global target to reduce deaths from suicide by a third by 2030 (indicator 3.4.2). WHO's Comprehensive Mental Health Action Plan has also set targets and strategies for reduction by the same year. Timely and detailed assessments of the burden of suicide are needed to track progress towards these initiatives. We searched for articles on PubMed published before May 1, 2024, using the search terms “suicide”, or “self-harm”, and “burden”. Our literature search retrieved numerous contemporary studies investigating deaths from suicide among specific populations, but the Global Burden of Diseases, Injuries, and Risk Factors Study (GBD) produces the only comprehensive estimates of suicide mortality at the global, regional, and national levels, disaggregated by age and sex, and inclusive of more than three decades of temporal trends. Other estimates, such as those provided by reports from WHO, are constrained by either more limited location detail or a narrower timeframe.

**Added value of this study**

Changing trends in suicide mortality across the world underscore the importance of timely and thorough analyses of its burden. This study provides updated, comprehensive estimates of deaths from suicide, building upon the previously published estimates from GBD 2016, which covered 1990 to 2016. Additionally, we have expanded our analyses to include additional age groups, mean age at the time of death by suicide, frequency of firearm use as a means of suicide, and the incidence of suicide attempts that required medical care compared with suicide deaths. We identified areas and demographic groups most affected by suicide in 2021, along with time trends of suicide mortality by location, age, and sex, from 1990 to 2021. Our estimates provide the most comprehensive analysis of the global burden of suicide to date, with results from 204 countries and territories. To our knowledge, this is the only study to report detailed results of deaths from suicide with this level of time and location detail.

**Implications of all the available evidence**

Quantifying trends in suicide is essential to provide appropriate and timely action. Despite global improvements in the age-standardised death rate over the past 31 years, certain demographic groups are showing rising rates of suicide, revealing specific locations and age groups that urgently need support and enhanced public health intervention strategies. Our analysis of suicide attempts compared with deaths by sex and location showed that males die from suicide more frequently, even though females are far more likely to attempt it. Such findings have important implications for suicide prevention strategies, particularly for highly lethal means, such as those involving firearms. Suicide is amenable to public health intervention: identifying populations most at risk is an essential component of a comprehensive suicide prevention strategy. Understanding the distribution of suicide mortality is fundamental for directing resources and efforts most effectively.

Preventing suicide requires a detailed understanding of existing patterns and trends, and how these vary between locations and demography, yet existing studies on the global burden of suicide of this nature are limited. We used results from the Global Burden of Diseases, Injuries, and Risk Factors Study (GBD) 2021, which estimated global health loss across 288 causes of disease and injury in 204 countries and territories from 1990 to 2021,^25^ to analyse patterns in suicide by age and sex. Additionally, GBD results were reanalysed to calculate, for the first time, the mean age at the time of suicide death, study-specific age groupings, and incidence mortality ratios. The aim of this study is to quantify patterns in suicide across time and by location, age, sex, and fraction of deaths compared with suicide attempts. By identifying populations most at risk for suicide, we can better inform decision makers, stimulate discussion, and spark innovation around targeted suicide prevention efforts. Since data on suicide remain sparse in some locations, these estimates could also motivate improved data collection efforts that would enable us to provide more accurate and comprehensive estimates moving forward, a key step in reducing the burden of suicide around the world. This manuscript was produced as part of the GBD Collaborator Network and in accordance with the GBD Protocol.^26^

## Methods

### Overview

We analysed GBD 2021 estimates of mortality, incidence, and mean age of death due to suicide for 204 countries and territories from 1990 to 2021. Detailed methods for GBD have been published previously.^25^, ^27^, ^28^ GBD 2021 estimates of deaths attributable to suicide were broken down into two comprehensive categories: those by firearms and those by other specified means. GBD's definition of suicide is “deliberate bodily damage inflicted on oneself resulting in death or injury”, and includes ICD-9 codes E950–E959 and ICD-10 codes X60–X64.9, X66–X84.9, and Y87.0. Our suicide estimates do not include assisted suicide or euthanasia.

This study complies with GATHER throughout (appendix 1 p 6).^29^ Software packages used in the cause-of-death analysis for GBD 2021 were Python (version 3.10.4), Stata (version 13.1), and R (version 4.2.1).

### Suicide mortality

Suicide mortality data preparation requires an abundance of caution due to the sensitivity of the subject and stigma associated with suicide in many cultures. Some deaths recorded on death certificates do not include ICD codes that fall under a range of codes that belong to a GBD cause, such as those mentioned above, and therefore cannot be directly assigned to a cause of death in the GBD cause hierarchy. Within the GBD framework, these are codes that are used to classify causes of death or morbidity that are either too vague, non-specific, or do not accurately represent a true underlying cause of death or disease.^30^, ^31^ According to previously described methods of redistribution, we identified cases of misassignment of suicide and reassigned those to this cause by age, sex, location, and year based on multiple causes of death data (data for which additional cause-of-death codes other than the underlying cause of death are listed) and other statistical analyses.^30^ In 2015, in the raw data, there were 303 541 deaths directly assigned to suicide in ICD-9 and ICD-10 vital registration sources. During the redistribution process, 75 149 (25%) deaths were added to these estimates. 82% of deaths that were added to suicide estimates during code redistribution came from ICD codes Y10–Y34, undetermined intent (appendix 1 p 14). Redistribution of deaths assigned to ICD codes Y10–Y33 used a regression method, and deaths assigned to Y34 used a separate method using multiple causes of death data. The strategies for producing redistribution weights for Y10–Y33 and Y34 have been described previously.^25^, ^30^

Estimation models for suicide used data acquired from vital registration, verbal autopsy, and mortality surveillance, including 23 782 study-location-years of data in GBD 2021. After data were redistributed as necessary, estimates for suicide were modelled exclusively using the Cause of Death Ensemble model (CODEm), which is a Bayesian hierarchical spatiotemporal ensemble modelling tool used to estimate causes with continuous, predictable mortality. In addition to observed datapoints, covariates were included to estimate location-years without data (appendix 1 pp 21–22).

We used the GBD standard-population structure to estimate age-standardised mortality rates per 100 000 population.^25^ Point estimates were calculated from the average of 1000 randomly selected possible values of deaths from suicide by age, sex, and geographical location. 95% uncertainty intervals (UIs) were derived from the 2·5th and 97·5th percentiles from a 1000-draw distribution.^25^

### Suicide incidence

In GBD 2021, there were 212 total data sources available to inform incidence estimates of suicide attempts, such as hospital data on inpatient and outpatient admissions, surveys, and scientific literature.^27^ Using these data sources, we estimated the incidence of suicide attempts requiring medical care. These methods are described extensively in previous publications.^27^, ^32^ We used DisMod-MR 2.1, a Bayesian meta-regression tool, to estimate the incidence rates for suicide injuries warranting inpatient admission.^27^, ^32^ Data sources from non-inpatient data (eg, outpatient or survey data) were adjusted for consistency among the model inputs in terms of case definition.^32^

In addition to drawing on our incidence data sources, as a compartmental model to reflect the epidemiological pathways of injuries, DisMod-MR 2.1 incorporated estimates of cause-specific mortality rates for suicide and excess mortality rates informed by the Healthcare Access and Quality Index, thereby enhancing the accuracy and consistency of global suicide burden estimates.^27^, ^32^ We also estimated the ratio of outpatient to inpatient admissions, and used this information to generate estimates of total incidence of suicide injuries warranting medical care through our non-fatal injury estimation pipeline.^32^

The incidence–mortality ratio was calculated by dividing the number of all suicide attempts, including fatal and non-fatal suicide attempts, by the number of suicide deaths.

### Calculation of mean age of death

Calculation of mean age at the time of death from suicide was done using GBD modelled death estimates. GBD produces cause-of-death estimates for every location-year-age group, even when there are no direct cause-of-death data available. GBD uses standard 5-year age groups from 5–9 years to 90–94 years; the remaining non-standard age groups consist of ages 0–6 days, 7–27 days, 1–5 months, 6–11 months, 12–23 months, 2–4 years, and 95 years and older. For this calculation, each GBD age group is assigned a distinct age of death by taking the average age of each age group. For example, the age group 15–19 years can be assigned to have a distinct age of death of 17 years old. The only age group without a discernable average is 95 years and older, which was assigned a distinct age of 95 years.

In GBD estimates, each modelled death falls into one of these age groups and can be assigned a distinct age of death. Distinct ages are then summed together for a given demographic consisting of a given location-year-sex-cause. This value is then divided by the total number of deaths for the same demographic to quantify the mean age of death.

### Calculation of custom age ranges

To produce custom age groups, multiple years were aggregated together to decrease sensitivity to variations from year to year in smaller countries. For a given age group, the total deaths over the past 5 years were summed together and divided by the sum of the population over the past 5 years. This value was then multiplied by 100 000 to produce mortality rates per 100 000. Five custom age groups were calculated (10–29, 30–49, 50–69, and ≥70 years).

### Role of the funding source

Coauthors affiliated with the funder of the study provided feedback on initial maps and drafts of this manuscript; otherwise, the funder had no role in study design, data collection, data analysis, data interpretation, or writing of the report.

## Results

### Trends in suicide mortality rates across time and location

Globally, 746 000 deaths (95% UI 692 000–800 000) from suicide occurred in 2021, of which 519 000 (485 000–556 000) were among males and 227 000 (200 000–255 000) were among females (table 1). The overall age-standardised mortality rate in 2021 was higher for males (12·8 deaths [12·0–13·7] per 100 000) than for females (5·4 [4·8–6·0] per 100 000; table 2). The age-standardised mortality rate for all sexes combined decreased over the study period, from 14·9 deaths (12·8–15·7) per 100 000 in 1990 to 9·0 deaths (8·3–9·6) per 100 000 in 2021, representing a decline of 39·5% (29·6–43·7). However, the rate of decrease was lower for males (33·5% [24·4–38·7]) than for females (50·3% [38·1–56·4]). Globally, there was no notable difference in age-standardised mortality rate before and after the start of the COVID-19 pandemic: in 2019, it was 9·2 deaths (8·6–9·7) per 100 000 and in 2021 it was 9·0 deaths (8·3–9·6) per 100 000.

**Table 1:** Numbers of deaths due to suicide and suicide attempts, incidence–mortality ratios, and number and percentage of suicide deaths that occurred from firearms, for males and females, globally and by region, 2021

	**Males**					**Females**				
	Suicide deaths (thousands)	Incident cases* (thousands)	Incidence–mortality ratio†	Suicide deaths from firearms		Suicide deaths (thousands)	Incident cases* (thousands)	Incidence–mortality ratio†	Suicide deaths from firearms	
				Count (thousands)	Percentage of all suicide deaths				Count (thousands)	Percentage of all suicide deaths
Global	519 (485–556)	2210 (1860–2590)	4·27 (3·54–5·16)	50·3 (40·1–62·1)	9·7% (7·9–12·0)	227 (200–255)	3270 (2730–3910)	14·47 (11·31–18·11)	6·6 (5·0–8·1)	2·9% (2·1–3·7)
Andean Latin America	2·46 (1·98–2·89)	6·13 (5·17–7·10)	2·52 (1·96–3·26)	0·1 (0·1–0·2)	5·3% (3·7–7·3)	0·783 (0·608–0·956)	8·59 (7·37–9·95)	11·11 (8·30–14·81)	0·0 (0·0–0·0)	1·3% (0·8–1·9)
Australasia	2·96 (2·78–3·12)	14·2 (12·8–15·6)	4·81 (4·31–5·37)	0·2 (0·2–0·2)	7·2% (6·9–7·5)	0·940 (0·892–0·982)	18·7 (16·7–20·8)	19·85 (17·56–22·34)	0·0 (0·0–0·0)	0·7% (0·7–0·8)
Caribbean	3·42 (2·99–3·86)	6·11 (5·43–6·75)	1·79 (1·51–2·12)	0·2 (0·1–0·3)	5·8% (4·1–8·1)	0·951 (0·794–1·12)	7·85 (6·78–9·06)	8·32 (6·54–10·36)	0·0 (0·0–0·0)	2·5% (1·5–3·4)
Central Asia	7·09 (6·43–7·77)	38·1 (34·8–41·6)	5·39 (4·68–6·13)	0·2 (0·2–0·2)	2·5% (2·3–2·8)	2·13 (1·89–2·39)	36·7 (32·7–41·1)	17·25 (14·53–20·21)	0·0 (0·0–0·0)	0·4% (0·4–0·5)
Central Europe	12·8 (11·9–13·6)	58·8 (52·3–65·5)	4·60 (4·01–5·28)	0·8 (0·6–1·0)	6·0% (4·7–7·4)	2·88 (2·61–3·17)	41·9 (36·9–47·3)	14·58 (12·40–16·95)	0·0 (0·0–0·0)	1·1% (0·8–1·5)
Central Latin America	14·0 (12·6–15·6)	29·8 (24·3–36·1)	2·14 (1·67–2·67)	1·9 (1·6–2·2)	13·5% (12·6–14·4)	3·17 (2·78–3·54)	31·2 (25·2–37·9)	9·89 (7·67–12·26)	0·2 (0·1–0·2)	4·9% (4·5–5·3)
Central sub-Saharan Africa	8·54 (6·46–11·8)	20·2 (16·9–23·6)	2·42 (1·65–3·35)	0·3 (0·1–0·8)	3·8% (1·4–7·7)	2·48 (1·70–3·64)	16·7 (13·7–20·4)	6·96 (4·28–9·90)	0·2 (0·1–0·3)	6·2% (2·5–12·4)
East Asia	81·7 (64·4–104)	364 (299–435)	4·53 (3·34–6·17)	0·0 (0·0–0·1)	0·1% (0·0–0·1)	55·5 (43·2–74·4)	571 (475–676)	10·50 (7·21–13·87)	0·0 (0·0–0·0)	0·0% (0·0–0·0)
Eastern Europe	39·6 (35·7–43·5)	204 (169–243)	5·18 (4·18–6·19)	1·4 (1·3–1·6)	3·6% (3·5–3·7)	9·10 (8·18–10·2)	133 (111–157)	14·65 (11·81–18·03)	0·0 (0·0–0·0)	0·3% (0·3–0·3)
Eastern sub-Saharan Africa	22·0 (18·9–27·0)	53·4 (44·1–63·5)	2·44 (1·83–3·09)	1·0 (0·4–2·0)	4·6% (1·7–8·5)	6·54 (5·22–8·15)	53·6 (43·7–65·6)	8·32 (6·01–11·25)	0·5 (0·2–0·9)	7·2% (3·0–12·6)
High-income Asia Pacific	25·6 (21·5–26·8)	127 (108–148)	4·99 (4·17–6·20)	0·0 (0·0–0·0)	0·1% (0·1–0·1)	10·5 (8·81–11·3)	178 (156–201)	16·93 (14·49–20·41)	0·0 (0·0–0·0)	0·0% (0·0–0·0)
High-income North America	43·1 (41·7–44·4)	270 (224–319)	6·28 (5·18–7·52)	22·5 (21·7–23·3)	52·3% (51·8–52·8)	12·4 (11·9–12·8)	380 (315–449)	30·69 (25·25–36·51)	3·5 (3·7–3·3)	28·2% (27·5–28·9)
North Africa and Middle East	15·5 (12·8–17·9)	103 (85·6–122)	6·70 (5·26–8·60)	1·5 (0·6–2·3)	9·9% (4·7–15·7)	5·71 (4·37–6·87)	134 (110–163)	23·83 (17·93–32·20)	0·3 (0·4–0·1)	4·9% (2·3–7·3)
Oceania	0·471 (0·398–0·561)	2·92 (2·32–3·58)	6·26 (4·48–7·99)	0·0 (0·0–0·0)	4·6% (1·6–7·1)	0·187 (0·148–0·282)	2·40 (1·97–2·95)	13·12 (7·99–17·17)	0·0 (0·0–0·0)	0·0% (0·0–0·1)
South Asia	131 (112–149)	491 (388–603)	3·78 (2·92–4·96)	11·1 (4·5–20·9)	8·4% (3·6–15·5)	81·6 (63·2–94·9)	1230 (975–1530)	15·22 (11·41–20·69)	1·2 (0·4–2·2)	1·4% (0·5–2·6)
Southeast Asia	25·4 (21·4–29·5)	134 (113–157)	5·34 (4·21–6·70)	0·7 (0·3–1·4)	2·9% (1·4–5·4)	8·20 (6·67–10·4)	124 (103–148)	15·31 (11·17–19·60)	0·0 (0·0–0·1)	0·4% (0·2–0·9)
Southern Latin America	6·08 (5·78–6·39)	34·8 (31·4–38·3)	5·73 (5·15–6·40)	1·0 (1·0–1·1)	17·0% (16·3–17·6)	1·41 (1·33–1·50)	33·7 (29·7–37·8)	23·93 (20·82–27·52)	0·1 (0·1–0·1)	7·2% (6·6–8·0)
Southern sub-Saharan Africa	10·0 (8·63–11·5)	30·4 (23·6–37·6)	3·06 (2·30–4·14)	0·4 (0·1–0·6)	3·6% (1·3–6·0)	2·55 (1·97–3·17)	22·6 (17·9–27·8)	8·96 (6·27–12·25)	0·1 (0·0–0·1)	2·6% (0·9–4·5)
Tropical Latin America	13·7 (13·2–14·3)	19·7 (15·5–24·3)	1·44 (1·13–1·79)	1·4 (1·3–1·4)	10·0% (9·6–10·4)	3·83 (3·66–3·98)	19·9 (16·0–23·9)	5·21 (4·19–6·32)	0·1 (0·1–0·2)	3·8% (3·6–4·0)
Western Europe	34·4 (32·8–35·9)	145 (132–158)	4·22 (3·83–4·66)	4·3 (4·0–4·6)	12·5% (12·1–12·9)	10·9 (10·2–11·4)	170 (151–189)	15·57 (13·77–17·70)	0·2 (0·2–0·2)	1·6% (1·5–1·6)
Western sub-Saharan Africa	19·5 (15·6–23·1)	58·4 (48·1–69·4)	3·04 (2·33–4·04)	1·1 (0·3–2·0)	5·8% (1·9–9·4)	5·56 (3·72–7·22)	61·7 (50·2–75·5)	11·40 (8·19–17·29)	0·2 (0·1–0·3)	3·9% (2·1–5·6)

Counts are presented to 3 significant figures.

* Includes suicide deaths and non-fatal suicide attempts that resulted in medical care.

† Number of incident cases divided by number of deaths.

**Table 2:** Age-standardised mortality rates due to suicide per 100 000 population, by sex and region in 1990, 2019, and 2021, and percentage changes, 1990–2021 and 2019–21

	**Mortality rate per 100 000**			**Percentage change**	
	1990	2019	2021	1990–2021	2019–21
**All sexes combined**					
Global	14·9 (12·8 to 15·7)	9·2 (8·6 to 9·7)	9·0 (8·3 to 9·6)	−39·5% (−43·7 to −29·6)	−2·1% (−6·7 to 3·4)
Andean Latin America	4·3 (3·8 to 4·8)	5·4 (4·5 to 6·1)	4·8 (3·9 to 5·7)	13·4% (−9·1 to 36·4)	−10·0% (−19·7 to −0·2)
Australasia	12·9 (12·5 to 13·2)	12·0 (11·4 to 12·5)	11·2 (10·6 to 11·7)	−13·3% (−18·0 to −8·6)	−6·9% (−8·4 to −5·2)
Caribbean	13·2 (12·4 to 13·8)	9·0 (8·1 to 9·9)	8·5 (7·5 to 9·6)	−35·8% (−43·0 to −27·6)	−5·2% (−12·3 to 2·8)
Central Asia	11·5 (11·1 to 12·0)	10·3 (9·8 to 10·8)	9·7 (8·8 to 10·7)	−15·4% (−24·0 to −6·9)	−5·4% (−11·9 to 1·3)
Central Europe	16·8 (16·5 to 17·2)	10·8 (10·5 to 11·0)	10·1 (9·5 to 10·8)	−39·8% (−43·8 to −36·0)	−5·7% (−11·5 to −0·4)
Central Latin America	4·7 (4·6 to 4·8)	6·6 (6·4 to 6·8)	6·5 (5·9 to 7·1)	38·9% (25·5 to 53·0)	−1·9% (−9·0 to 6·6)
Central sub-Saharan Africa	16·4 (13·2 to 19·7)	14·4 (11·0 to 19·1)	14·4 (11·0 to 19·1)	−11·9% (−29·7 to 16·5)	−0·0% (−5·0 to 5·6)
East Asia	21·1 (14·3 to 24·1)	7·3 (6·3 to 8·8)	7·2 (6·0 to 8·8)	−65·7% (−72·6 to −47·5)	−0·7% (−18·5 to 21·0)
Eastern Europe	24·9 (24·6 to 25·2)	19·9 (19·1 to 20·8)	19·2 (17·5 to 20·8)	−23·1% (−29·7 to −16·6)	−3·9% (−12·9 to 4·8)
Eastern sub-Saharan Africa	16·6 (14·4 to 18·4)	12·4 (10·8 to 14·4)	12·2 (10·6 to 14·4)	−26·5% (−37·3 to −9·6)	−1·4% (−7·4 to 4·5)
High-income Asia Pacific	14·7 (14·1 to 18·5)	14·4 (12·3 to 14·8)	14·1 (12·2 to 14·6)	−4·1% (−33·7 to 1·6)	−1·7% (−3·0 to −0·4)
High-income North America	11·9 (11·8 to 12·1)	13·2 (12·9 to 13·4)	12·8 (12·4 to 13·2)	7·3% (4·0 to 10·9)	−2·7% (−4·5 to −0·8)
North Africa and Middle East	4·7 (3·7 to 5·2)	3·6 (3·0 to 4·0)	3·4 (2·8 to 3·9)	−26·6% (−35·6 to −3·6)	−3·9% (−6·9 to −0·8)
Oceania	7·2 (6·0 to 8·4)	5·6 (4·8 to 6·7)	5·4 (4·6 to 6·4)	−25·4% (−34·7 to −6·1)	−3·60% (−7·7 to 0·4)
South Asia	16·8 (12·9 to 18·7)	11·6 (9·8 to 12·6)	11·4 (9·7 to 12·7)	−31·7% (−41·0 to −9·2)	−1·2% (−10·1 to 8·5)
Southeast Asia	7·0 (6·2 to 7·7)	4·8 (4·2 to 5·4)	4·7 (4·0 to 5·4)	−33·2% (−42·3 to −20·9)	−2·1% (−9·5 to 5·8)
Southern Latin America	12·2 (11·8 to 12·6)	11·1 (10·7 to 11·5)	10·0 (9·5 to 10·5)	−18·2% (−23·0 to −13·6)	−10·1% (−12·0 to −8·0)
Southern sub-Saharan Africa	18·0 (14·3 to 21·2)	15·9 (14·0 to 18·1)	16·1 (14·0 to 18·3)	−10·5% (−22·1 to 2·4)	1·1% (−5·2 to 7·3)
Tropical Latin America	6·4 (6·2 to 6·6)	7·0 (6·8 to 7·2)	7·0 (6·8 to 7·3)	9·5% (5·5 to 13·8)	0·0% (−2·5 to 2·6)
Western Europe	12·8 (12·5 to 13·0)	8·2 (7·9 to 8·4)	7·5 (7·3 to 7·8)	−40·8% (−42·5 to −38·9)	−7·4% (−8·5 to −6·3)
Western sub-Saharan Africa	10·2 (8·1 to 11·9)	9·8 (7·7 to 11·4)	9·5 (7·5 to 11·0)	−6·8% (−24·6 to 24·6)	−2·7% (−14·5 to 10·7)
**Males**					
Global	19·3 (17·1 to 20·5)	13·2 (12·4 to 13·9)	12·8 (12·0 to 13·7)	−33·5% (−38·7 to −24·4)	−2·7% (−7·6 to 3·2)
Andean Latin America	5·9 (5·2 to 6·6)	8·2 (6·8 to 9·4)	7·4 (6·0 to 8·8)	25·6% (−0·4 to 53·6)	−9·5% (−20·3 to 1·7)
Australasia	20·8 (20·2 to 21·4)	18·5 (17·5 to 19·4)	17·2 (16·1 to 18·1)	−17·4% (−22·4 to −12·3)	−7·2% (−8·9 to −5·2)
Caribbean	18·6 (17·5 to 19·5)	14·4 (13·1 to 15·8)	13·8 (12·0 to 15·5)	−26·1% (−34·4 to −16·9)	−4·8% (−12·6 to 3·0)
Central Asia	18·1 (17·3 to 18·9)	16·3 (15·5 to 17·2)	15·4 (14·0 to 16·9)	−15·0% (−23·4 to −6·2)	−5·5% (−12·0 to 1·5)
Central Europe	27·2 (26·6 to 27·8)	18·5 (18·0 to 18·8)	17·4 (16·2 to 18·5)	−35·9% (−40·3 to −31·9)	−5·5% (−12·0 to −0·2)
Central Latin America	8·1 (7·9 to 8·3)	11·1 (10·7 to 11·6)	11·0 (9·9 to 12·2)	36·2% (21·9 to 50·6)	−1·4% (−9·2 to 7·6)
Central sub-Saharan Africa	27·0 (21·4 to 33·3)	24·4 (18·4 to 32·2)	24·4 (18·6 to 32·6)	−9·4% (−29·8 to 20·5)	0·3% (−4·6 to 5·9)
East Asia	21·8 (14·6 to 25·6)	9·2 (7·3 to 11·6)	9·1 (7·2 to 11·5)	−58·4% (−69·2 to −35·0)	−0·8% (−25·0 to 31·8)
Eastern Europe	43·8 (43·3 to 44·4)	35·9 (34·1 to 37·7)	34·2 (30·9 to 37·5)	−21·9% (−29·3 to −14·2)	−4·6% (−14·4 to 5·4)
Eastern sub-Saharan Africa	25·9 (22·0 to 29·6)	20·1 (17·4 to 23·8)	19·9 (17·3 to 23·9)	−23·1% (−35·6 to −7·5)	−1·1% (−8·0 to 6·7)
High-income Asia Pacific	19·5 (18·8 to 25·3)	20·6 (17·7 to 21·3)	20·1 (17·3 to 20·9)	3·1% (−29·9 to 9·5)	−2·5% (−4·0 to −1·1)
High-income North America	19·9 (19·6 to 20·2)	20·8 (20·4 to 21·2)	20·3 (19·6 to 20·9)	1·7% (−1·7 to 5·1)	−2·6% (−4·6 to −0·6)
North Africa and Middle East	6·0 (4·9 to 6·8)	5·0 (4·2 to 5·8)	4·8 (4·0 to 5·6)	−19·8% (−31·3 to 4·2)	−3·4% (−6·9 to −0·1)
Oceania	10·0 (8·2 to 11·8)	7·8 (6·5 to 9·0)	7·5 (6·4 to 8·8)	−25·3% (−34·9 to −6·8)	−3·2% (−7·7 to 0·7)
South Asia	19·0 (14·6 to 21·6)	14·5 (12·5 to 16·2)	14·2 (12·3 to 16·2)	−25·0% (−36·6 to −1·7)	−2·2% (−13·7 to 10·2)
Southeast Asia	10·3 (9·1 to 11·5)	7·4 (6·4 to 8·3)	7·3 (6·2 to 8·4)	−29·3% (−39·3 to −16·1)	−1·9% (−10·0 to 7·3)
Southern Latin America	20·6 (20·0 to 21·4)	18·8 (18·0 to 19·6)	16·9 (16·0 to 17·7)	−18·3% (−23·1 to −13·5)	−10·2% (−12·2 to −8·0)
Southern sub-Saharan Africa	28·3 (23·2 to 34·2)	26·5 (23·6 to 30·4)	26·7 (23·2 to 30·6)	−5·6% (−18·7 to 7·5)	0·9% (−6·1 to 8·6)
Tropical Latin America	10·4 (10·1 to 10·7)	11·4 (11·1 to 11·8)	11·4 (11·0 to 11·8)	9·3% (4·7 to 14·5)	−0·5% (−3·6 to 2·6)
Western Europe	19·4 (19·1 to 19·8)	12·8 (12·4 to 13·2)	11·8 (11·4 to 12·2)	−39·1% (−41·1 to −37·0)	−7·3% (−8·5 to −6·1)
Western sub-Saharan Africa	16·0 (12·3 to 19·2)	15·9 (12·9 to 18·9)	15·6 (12·6 to 18·5)	−2·3% (−22·7 to 32·9)	−2·0% (−15·8 to 14·5)
**Females**					
Global	10·9 (7·9 to 12·1)	5·4 (4·7 to 6·1)	5·4 (4·8 to 6·0)	−50·3% (−56·4 to −38·1)	−0·6% (−9·0 to 9·0)
Andean Latin America	2·7 (2·3 to 3·1)	2·6 (2·1 to 3·1)	2·3 (1·8 to 2·8)	−13·2% (−32·4 to 9·5)	−10·8% (−19·1 to −1·6)
Australasia	5·3 (5·1 to 5·5)	5·7 (5·4 to 5·9)	5·3 (5·1 to 5·6)	1·0% (−4·3 to 6·7)	−6·2% (−8·0 to −4·5)
Caribbean	8·2 (7·6 to 8·7)	3·9 (3·2 to 4·4)	3·6 (3·0 to 4·3)	−55·9% (−62·2 to −48·6)	−5·8% (−12·8 to 1·9)
Central Asia	5·6 (5·4 to 5·9)	4·8 (4·5 to 5·1)	4·6 (4·1 to 5·1)	−19·0% (−28·1 to −9·2)	−5·3% (−12·8 to 2·7)
Central Europe	7·5 (7·3 to 7·8)	3·6 (3·4 to 3·8)	3·3 (3·0 to 3·7)	−55·7% (−59·4 to −51·5)	−6·2% (−13·5 to 0·5)
Central Latin America	1·5 (1·4 to 1·5)	2·4 (2·3 to 2·5)	2·3 (2·0 to 2·6)	60·0% (40·1 to 79·8)	−3·3% (−12·8 to 6·0)
Central sub-Saharan Africa	6·8 (4·9 to 8·6)	6·3 (4·3 to 9·0)	6·2 (4·3 to 8·9)	−8·3% (−33·5 to 33·4)	−0·3% (−6·1 to 6·4)
East Asia	20·9 (12·2 to 25·3)	5·6 (4·3 to 7·3)	5·6 (4·4 to 7·5)	−73·2% (−80·6 to −56·2)	−0·6% (−26·4 to 37·6)
Eastern Europe	9·3 (9·1 to 9·4)	6·2 (5·9 to 6·6)	6·1 (5·5 to 6·9)	−34·2% (−41·1 to −26·4)	−1·9% (−13·6 to 10·8)
Eastern sub-Saharan Africa	7·7 (5·8 to 9·2)	5·4 (4·4 to 6·6)	5·4 (4·4 to 6·6)	−30·1% (−43·2 to −5·2)	−0·7% (−8·4 to 7·8)
High-income Asia Pacific	10·3 (9·7 to 12·8)	8·2 (6·9 to 8·6)	8·2 (7·0 to 8·7)	−20·4% (−43·2 to −14·6)	0·3% (−1·5 to 2·0)
High-income North America	4·8 (4·7 to 4·9)	5·9 (5·8 to 6·0)	5·7 (5·5 to 5·9)	19·3% (15·1 to 23·8)	−3·1% (−5·2 to −0·9)
North Africa and Middle East	3·3 (2·2 to 3·8)	2·0 (1·6 to 2·4)	1·9 (1·5 to 2·3)	−41·1% (−50·3 to −13·1)	−5·0% (−8·5 to −1·0)
Oceania	4·2 (3·3 to 5·2)	3·3 (2·7 to 4·7)	3·2 (2·5 to 4·6)	−24·1% (−37·2 to 2·0)	−4·3% (−9·4 to 0·4)
South Asia	14·3 (9·6 to 16·8)	8·6 (6·6 to 9·8)	8·7 (6·7 to 10·1)	−39·2% (−49·7 to −8·6)	0·6% (−12·0 to 15·8)
Southeast Asia	4·1 (3·1 to 4·6)	2·4 (2·0 to 3·0)	2·3 (1·9 to 2·9)	−44·0% (−53·0 to −22·6)	−2·9% (−11·1 to 6·4)
Southern Latin America	4·8 (4·6 to 4·9)	4·1 (3·9 to 4·3)	3·7 (3·5 to 3·9)	−22·5% (−27·6 to −16·8)	−10·1% (−12·6 to −7·6)
Southern sub-Saharan Africa	8·9 (6·2 to 10·4)	6·5 (5·1 to 7·8)	6·5 (5·1 to 8·1)	−26·6% (−41·3 to −9·2)	0·5% (−7·4 to 8·8)
Tropical Latin America	2·7 (2·6 to 2·8)	2·9 (2·9 to 3·0)	3·0 (2·9 to 3·1)	10·1% (5·0 to 14·8)	1·8% (−2·3 to 5·2)
Western Europe	6·9 (6·6 to 7·0)	3·8 (3·6 to 3·9)	3·5 (3·3 to 3·6)	−49·3% (−50·8 to −47·7)	−8·1% (−9·2 to −6·9)
Western sub-Saharan Africa	4·5 (3·6 to 5·6)	4·1 (2·8 to 5·1)	4·0 (2·7 to 5·1)	−10·6% (−32·0 to 19·3)	−2·0% (−14·0 to 12·1)

**Figure 1:**
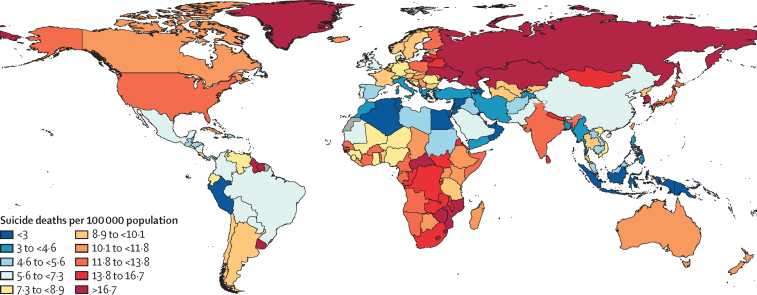
Age-standardised mortality rate from suicide per 100 000 population, males and females combined, 2021

While global declines in age-standardised mortality rate from suicide have occurred over the study period, there has been wide variability at the regional level (table 2). In 2021, the age-standardised mortality rate for all sexes combined was highest in eastern Europe (19·2 deaths [95% UI 17·5 to 20·8] per 100 000), southern sub-Saharan Africa (16·1 deaths [14·0 to 18·3] per 100 000), and central sub-Saharan Africa (14·4 deaths [11·0 to 19·1 per 100 000]; figure 1). Since 1990, four regions have had increases in age-standardised mortality rate for all sexes combined, the largest increase being in central Latin America, followed by Andean Latin America, Tropical Latin America, and high-income north America (table 2). The substantial increase in central Latin America (38·9% [25·5 to 53·0]) was the result of a 36·2% (21·9 to 50·6) increase among males and a 60·0% (40·1 to 79·8) increase among females. In Andean Latin America, however, the increase occurred only among males, with a rise of 25·6% (–0·4 to 53·6), while the rate for females declined by 13·2% (–9·5 to 32·4). Conversely, high-income north America had an increase in age-standardised mortality rate between 1990 and 2021 for females (19·3% [15·1 to 23·8]), with only a slight change in the rate for males (1·7% [–1·7 to 5·1]). Tropical Latin America, however, had a similar rate of increase for males (9·3% [4·7 to 14·5]) and females (10·1% [5·0 to 14·8]). Overall, eastern Europe had the highest age-standardised mortality rate for males (34·2 deaths [30·9 to 37·5] per 100 000) in 2021, despite decreasing by 21·9% (14·2 to 29·3) since 1990. Among females, the age-standardised mortality rate in 2021 was highest in south Asia (8·7 [6·7 to 10·1]), despite a decrease of 39·2% (8·6 to 49·7) since 1990.

The largest improvement occurred in east Asia, where the age-standardised mortality rate decreased by 65·7% (95% UI 47·5–72·6), from 21·1 deaths (14·3–24·1) per 100 000 in 1990, to 7·2 deaths (6·0–8·8) per 100 000 in 2021 (table 2), with decreases among males (58·4% [35·0–69·2]) and females (73·2% [56·2–80·6]). The second largest overall decline occurred in western Europe, which had a reduction of 40·8% (38·9–42·5), followed by central Europe, with a decline of 39·8% (36·0–43·8). Age-standardised mortality rates due to suicide in 1990, 2019, and 2021 along with percent changes from 1990 to 2021 at the national level are available in appendix 1 (pp 26–38).

### Trends in suicide mortality rates by age group

Age-specific mortality rates by sex among four distinct age groups are shown in appendix 1 (pp 16–17, 24–25). Globally, the highest mortality rate occurred in those aged 70 years and older among both males (37·9 deaths [95% UI 33·4–41·4] per 100 000) and females (15·6 [12·7–18·0] per 100 000), with declines in mortality rate occurring among males and females with each younger age group. Among females aged 10–29 years, south Asia had the highest mortality rate (11·6 [9·5–13·6] per 100 000) among all GBD regions. Among males of the same age group, the mortality rate was highest in eastern Europe (25·7 [24·0–27·5] per 100 000). In the 30–49 years age group, the highest regional rates were estimated in the high-income Asia Pacific region for females (11·8 [9·5–12·7] per 100 000) and in eastern Europe for males (59·2 [55·5–63·3] per 100 000). Central sub-Saharan Africa had the highest rates of suicide deaths for females in the 50–69 years age group (15·1 [9·4–22·7] per 100 000), while for males in this age group eastern Europe had the highest rate (53·9 [50·3–58·1] per 100 000), closely followed by central sub-Saharan Africa (51·9 [36·0–73·5] per 100 000). Among those aged 70 and older, the mortality rate for males was highest in central sub-Saharan Africa (122·0 [85·9–172·0] per 100 000), while for females it was highest in east Asia (30·9 [23·4–40·1] per 100 000). The rates of suicide by age group at the national level are presented in appendix 1 (pp 64–78).

We provide the ranking of suicides among the leading causes of death from 2017 to 2021 in appendix 1 (pp 24–25). At the global level, suicide was the third leading cause of death among males and females aged 10–29 years. Notably, suicide ranked as the number one cause of death among both males and females of that age group in Australasia, high-income Asia Pacific, southern Latin America, and western Europe. Suicide was the leading cause of death in several other age groups at the regional level. The rank of suicides by age group at the national level is presented in appendix 1 (pp 64–78).

### Trends in mean age at time of death by location

**Figure 2:**
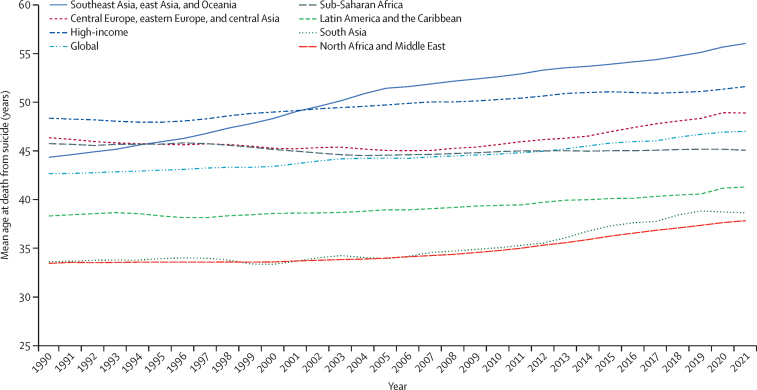
Time trend of mean age of death due to suicide from 1990 to 2021, for all ages and sexes combined, by super-region

Patterns of change in the mean age at the time of death from suicide varied by super-region (figure 2). The global mean age at death from suicide was 42·6 years (95% UI 36·8 to 45·1) in 1990 and 47·0 years (43·3 to 50·3) in 2021. For males globally, the mean age at death increased by 4·0 years (0·0 to 9·8), from 43·0 years (38·0 to 45·8) in 1990 to 47·0 years (43·5 to 50·6) in 2021 (appendix 1 pp 39–50). Similarly, for females globally, the mean age increased by 5·0 years (–0·4 to 14·9), from 41·9 years (30·9 to 46·7) in 1990 to 46·9 years (41·2 to 52·8) in 2021.

Among regions, east Asia had the largest increase in age at death from suicide, from 45·1 years (95% UI 30·9–51·7) to 58·8 (48·8–71·9) for all sexes combined (appendix 1 pp 39–50). In the eastern sub-Saharan Africa and western sub-Saharan Africa regions, the mean age of death from suicide among males decreased between 1990 and 2021. For all sexes combined, western sub-Saharan Africa had a lower mean age of death in 2021 than in 1990. Across the study period, the south Asia super-region and north Africa and the Middle East super-region consistently had the youngest mean age of death from suicide (figure 2), while the high-income super-region and southeast Asia, east Asia, and Oceania super-region had the oldest.

National-level estimates are provided for mean age and disaggregated by sex in appendix 1 (pp 39–50), including the differences in mean age by Socio-demographic Index (SDI). The mean age increased by several years within locations at each level of the SDI, except in locations characterised as low SDI, where the mean age was largely unchanged from 1990 to 2021 (appendix 1 p 39).

### Firearm-related suicide

**Figure 3:**
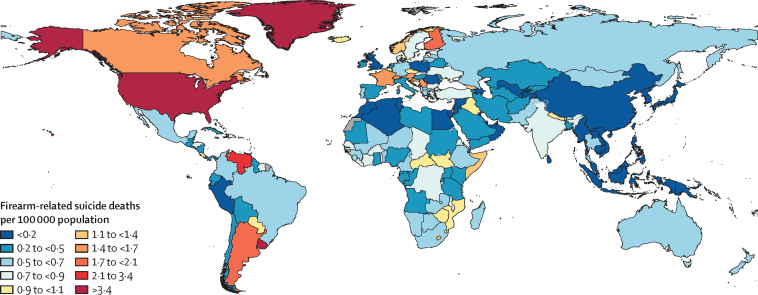
Age-standardised mortality rate for firearm-related suicides per 100 000 population, males and females combined, 2021

Figure 3 shows a global map of firearm-related suicides. The countries with the highest age-standardised mortality rate due to suicide from firearms in 2021 were the USA (6·19 deaths [95% UI 5·96–6·42] per 100 000 population), Uruguay (3·61 [3·34–3·87] per 100 000), and Venezuela (3·04 [2·31–3·88] per 100 000 (appendix pp 79–87; GBD Compare). The territory of Greenland also had a high rate (12·9 [7·70–19·4] per 100 000), although, given its small population size, the total number of firearm-related suicides was low in 2021 (8·25 deaths [4·83–12·3]). Among males who died from suicide globally in 2021, 9·7% (7·9–12·0) of deaths were caused by firearms (table 1). For females, the percentage of suicide deaths from firearms was 2·9% (2·1–3·7). High-income north America had the highest proportion of suicide deaths due to firearms in 2021, by a large margin, for both males (52·3% [51·8–52·8]) and females (28·2% [27·5–28·9]). The second-leading region by proportion of suicides due to firearms was southern Latin America (17·0% [16·3–17·6] for males and 7·2% [6·6–8·0] for females. Among all sexes combined, there was no substantial difference between the mean age of suicide death by firearm (46·5 years [39·5–53·4]) versus suicide by any other means (47·0 years [43·5–50·7]; appendix 1 p 23). However, females who died from firearm suicides were on average younger (43·8 years [34·8–52·1]) than females who died from other forms of suicide (47·0 years [41·3–53·3]).

### Incidence of suicide attempts

We calculated the incidence–mortality ratio to assess the frequency of suicide attempts requiring medical care that did not result in a death, and found stark variance between males and females (table 1; appendix 1 pp 51–63). In 2021, the incidence–mortality ratio of suicide globally was much higher for females (14·47 [95% UI 11·31–18·11]) than for males (4·27 [3·54–5·16]). This trend was consistent for all 21 regions, with incidence–mortality ratios being around 2–4 times higher for females than for males. This difference was highest in north America, with one in 30·7 attempts (25·2–36·5) resulting in a death for females, and one in 6·3 attempts (5·2–7·5) resulting in a death for males.

## Discussion

Deaths from suicide remain an important cause of preventable mortality across the globe, with 746 000 such deaths in 2021. Trends in age-standardised mortality rate over the past several decades show large improvements, with a decline of 39·5% since 1990, but this trend is not consistent throughout the world or by demographic group. Age-specific and sex-specific estimates yield important insights, showing that the number of deaths for males was more than twice that for females, and the mean age of death at the time of suicide has been steadily rising. Several regions also saw sharp increases in age-standardised mortality rate over the study period, and firearms as a means of suicide remain an important contributor in some locations. An understanding of persistent demographic differences in suicide patterns and the changing distribution across age groups over time is essential to support targeted suicide reduction efforts.

In addition to geographical trends, our study identifies several age-specific and sex-specific patterns of suicide, which could have important implications for prevention efforts. Our analysis of mean age at the time of death shows that both males and females are dying from suicide later in life, relative to the mean age at the time of death in 1990. This rise in mean age of death might be influenced by efforts focused on the prevention of suicide among younger people, such as providing access to essential screening services or expanding mental health programmes for youth.^33^ Strong health systems have the potential to support individuals with mental health problems, in addition to providing potentially life-saving treatment for less lethal suicide methods. In communities with limited access to high-quality health care and a scarcity of mental health programmes, however, particularly in low socioeconomic settings, the mean age of suicide is much lower. Conversely, the rise in mean age of suicide deaths in some locations might indicate deficiencies in socioeconomic support systems and a paucity of strategies for ageing populations.^6^ Factors such as isolation, absence of social or familial support, inadequate social security and retirement plans, chronic illness, insufficient or low-coverage insurance, and the absence of mental health resources for middle-aged and older adults are critical risk factors contributing to suicide in middle and older age groups.^6^

Although the mean age at death is generally trending upward, our results show it remains a persistent leading cause of death for young people, between age 10 years and 49 years, in many areas of the world. Young people in central and eastern Europe have a high risk for suicide that can be attributed to alcohol and substance use,^34^ as well as to mental and behavioural health problems.^35^ Young adults in central Asia also have higher age-specific mortality rates that have been associated with higher rates of mental illness, declines in the standard of living, unemployment, and feelings of ambiguity or hopelessness about the future.^36^ One study looking at adolescent suicides across low-income and middle-income countries noted that the driving factors of youth suicide often differ between high-income and low-income areas.^37^ For example, young people living in some low-income nations are more impacted by political tensions, limited health-care resources, poverty, or a higher disease burden.^37^ Our study identifies suicide as a leading cause of death for young people in both high-income and low-income locations. Some studies implicate interpersonal issues such as family conflict as a leading driver of youth suicide in some higher-income nations.^38^, ^39^ It is important to note that risk factors for suicide among younger generations are complex and often the result of a combination of psychological, cultural, and social influences. An abundance of caution must be taken when designing intervention approaches for young people, as drivers of suicide in young people are highly variable and must incorporate a tailored, public health approach to prevention.

Differences between male and female suicides have long been studied.^40^, ^41^, ^42^ Our study shows that female suicide mortality rates have largely declined since 1990, by as much as 50% globally across all ages which included remarkable reductions in east Asia (73%), the Caribbean (56%), and central Europe (56%). Unfortunately, suicide mortality rates among females in central Latin America, high-income north America, and tropical Latin America have increased over the study period. Similar to 1990, countries in south Asia and high-income Asia Pacific still carry some of the highest burden of female suicide mortality. In 2020, in India, the highest suicide death rates were among educated women, with family problems being the most commonly cited contributing factor.^43^ The varying patterns of suicide across locations for females emphasise the need for local strategies for suicide prevention. On both a global scale and for each of 21 GBD regions, the incidence of suicide attempts among females was notably higher than that among males, despite the overall age-standardised mortality rate being much higher for males across the period studied. Females overwhelmingly attempted suicide more often than males, but males are far more likely to die from it, a pattern that has been referred to as the gender paradox of suicide.^44^ A leading factor is the choice of means of suicide, where males are more likely to use highly lethal methods, such as firearms.^45^

Firearms are considered the most lethal means of suicide,^46^ but our study shows that only a few countries had a high proportion of firearm-related suicide deaths, particularly the USA, Uruguay, and Venezuela. Notably, in 2021, 90·0% of suicide deaths by a firearm were located in areas with just 52·1% of the global population.^25^ Abundant research has shown that high rates of firearm-related suicide deaths are linked to rates of firearm possession.^47^, ^48^, ^49^ Particularly within the USA, research consistently finds a correlation between firearm ownership and suicide,^50^, ^51^, ^52^ and that owning a firearm increases the risk of death from suicide.^53^, ^54^ One study looking at data from the Small Arms Survey and the National Violent Death Reporting System found that firearm suicide rates are about twice as high among USA-born suicide decedents compared with their foreign-born counterparts.^55^

Many individual and societal factors are associated with suicide, including mental illness,^56^ early developmental experiences,^18^, ^23^ social isolation,^18^ and economic insecurity.^20^ The stigmatised nature of these underlying risk factors can prevent individuals from receiving appropriate help in many communities.^6^ Deaths from suicide are preventable, and many studies show the need to implement proximal, evidence-based interventions directed at suicide, including those in the WHO Mental Health Action Plan,^6^ and the WHO Live Life Implementation Guide,^20^ as well as improving more distal social determinants of health. Successful strategies to reduce suicide differ between high-income and low-income settings, however.^20^ Interventions directed at low-income countries should incorporate a comprehensive approach that goes beyond mental health care and addresses the socioeconomic factors contributing to distress. Strengthening social support systems is crucial, as strong community networks can provide a buffer against the social isolation often associated with suicide risk.^24^ Additionally, public awareness campaigns that reduce stigma surrounding suicide and encourage help-seeking behaviour can create a more supportive environment. Promoting protective factors, such as family stability and social integration, and addressing harmful cultural practices that might increase suicide risk are crucial in the long-term reduction of suicides.^20^ Although interventions focused exclusively on mental health might be insufficient in some cases, it remains crucial to improve access to mental health care, particularly in low-income settings. Integrating mental health care into primary health systems can improve access and reduce stigma, making services more widely available to underserved populations.^6^

Our study has several limitations, including those that are described for the overall GBD enterprise.^27^ Estimates of suicide mortality and suicide hospitalisations are subject to a lag in reporting and a scarcity of data in some locations, particularly from low-income and middle-income countries. According to previous studies^57^ and WHO,^58^ suicide is under-reported more often than other causes of death for numerous reasons. There is often a delay in assigning the intent to a death until a post-mortem examination is completed in many countries. It is likely that such cases will be recorded as undetermined until then. When high-quality data are available, the reported cause of death might not have enough detail to assign directly to a GBD cause. We redistributed these incorrectly or vaguely assigned causes of death to appropriate, plausible underlying causes of death.^25^, ^30^ Additionally, the years of estimation since the COVID-19 pandemic face unique challenges that will not be completely understood until additional data are available. Those challenges include the impact of post-COVID-19 condition (also known as long COVID),^59^ as well as the impact of social challenges during the pandemic.^60^ Furthermore, the calculation of mean age at the time of suicide death is not standardised for different population age structures. Because of this, aggregate estimates are driven by the most populous locations. Consequently, it is not immediately clear whether an increase in the mean age at death is due to an improvement in suicide reduction among younger populations or whether it is due to an ageing population.^61^ Lastly, the method of suicide that is chosen has crucial implications for policy and intervention strategies. Currently, we are only able to provide estimates of means of suicide by two categories: those from firearms and those from other means. While estimates of suicides by firearm are imperative for reduction efforts in a small number of countries identified in this study, other means of suicides, such as hangings and poisonings, are far more prevalent in other areas of the world and require additional data collection efforts to quantify and understand their burden.

Future cycles of GBD will expand upon the estimation of causes of death by disaggregating broad categories into more detailed causes, where possible. Future iterations will focus on collecting and analysing data to further inform the burden of suicide by quantifying additional means. Subsequent studies will be able to offer a more complete understanding of how additional means of suicide—including hangings, poisonings (those by pesticides and those by non-pesticide substances), and self-immolation—vary by location, sex, and age. We anticipate that this increased understanding will enable and strengthen more effective and actionable insights for policy making.

Progress in reducing suicide mortality has been laudable in some regions and within some age groups; however, even in areas where reduction efforts have been sustained, suicide remains an important preventable contributor to the burden of disease across all locations. Contemporary analyses by location that encompass details on age and sex are necessary for informing future suicide prevention initiatives, along with modifying existing ones. Caution must be given to ensure that strategies are not applied to locations and populations where they are not well suited; suicide prevention approaches are not universally transferrable. More evidence regarding effective reduction strategies to fit within the context of regional and national needs is necessary to reduce this important and preventable cause of death.

### GBD 2021 Suicide Collaborators

### Affiliations

### Contributors

### Data sharing

To download the data used in these analyses, please visit the Global Health Data Exchange at https://ghdx.healthadata.org/record/ihme-data/gbd-2021-suicide-mortality-incidence-1990-2021.

## Declaration of interests

T Bärnighausen reports grants or contracts paid to their institution from National Institutes of Health, Alexander von Humboldt Foundation, German National Research Foundation (DFG), EU, German Ministry of Education and Research, German Ministry of the Environment, Wellcome, and KfW; payment or honoraria for lectures, presentations, speakers bureaus, manuscript writing or educational events from PLOS Medicine as Editor-in-Chief; participation on Advisory Boards for NIH-funded research projects in Africa on Climate Change and Health, unpaid; stock or stock options in CHEERS, an SME focusing on approaches to measure climate change and health-related variables in population cohorts (EUR 5,000 total stock ownership); all outside the submitted work. A Faro reports support for the preset manuscript from Bolsa Produtividade. I Ilic reports support for their participation in the present manuscript from the Ministry of Education, Science and Technological Development, Republic of Serbia (project No 175042, 2011-2023). M Ilic reports support for their participation in the present manuscript from the Ministry of Technological Development and Innovation of the Republic of Serbia (number 451-03-47/2023-01/200111). J J Jozwiak reports payment or honoraria for lectures, presentations, speakers' bureaus, manuscript writing or educational events from NOVARTIS, ADAMED, and AMGEN; outside the submitted work. R Liu reports grants or contracts from National Institute of Mental Health (grant numbers R01 MH115905, RF1 MH120830, R01 MH124899, R21 MH130767, awarded to Massachusetts General Hospital); consulting fees from Relmada Therapeutics; payment or honoraria for lectures, presentations, speakers bureaus, manuscript writing or educational events from Miami International Child and Adolescent Mental Health Conference, Massachusetts General Hospital, University of California (San Francisco); support for attending meetings or travel from the American Foundation for Suicide Prevention; participation on a data safety monitoring board (DSMB) or advisory board for the University of Pennsylvania (Chair for DSMB), University of Minnesota, and Massachusetts General Hospital; leadership or fiduciary role in other board, society, committee or advocacy group (paid or unpaid) with the Association for Behavioral and Cognitive Therapies and the American Psychological Association, all outside the submitted work. T Miller reports grants or contracts from National Institute on Mental Health, Santa Clara County Department of Health, and Everytown for Gun Safety, all payments made to their institution; payment for expert testimony from Everytown for Gun Safety, all payments made to their institution, all outside the submitted work. L Monasta reports support for the present manuscript from the Italian Ministry of Health (Ricerca Corrente 34/2017), payments made to the Institute for Maternal and Child Health IRCCS Burlo Garofolo. F Mughal reports support for the present manuscript from the National Institute for Health and Care Research (NIHR) Doctoral Fellowship (NIHR300957). The views expressed in this Article are those of the authors and not necessarily those of NHS, NIHR, or the Department for Health and Social Care. S Nomura reports support for the present manuscript from the Ministry of Education, Culture, Sports, Science and Technology of Japan (grant 24H00663) and the Precursory Research for Embryonic Science and Technology from the Japan Science and Technology Agency (grant JPMJPR22R8). B Oancea reports grants or contracts from the MRID (project PNRR-I8 number 842027778, contract number 760096). R F Palma-Alvarez reports payment or honoraria for lectures, presentations, speakers bureaus, manuscript writing or educational events from Angelini, Casen Recordati, Lundbeck, Neuraxpharm, Rubió, Servier, and Takeda; support for attending meetings or travel from Angelini, Lundbeck, Janssen, Italfarmaco, Advanz Pharma, and Takeda, all outside the submitted work. V Sharma acknowledges support from DFSS (MHA)'s research project (DFSS28(1)2019/EMR/6) at the Institute of Forensic Science & Criminology, Panjab University, Chandigarh, India, outside the submitted work. V Shivarov has one patent issued from the Bulgarian Patent Office; stock or stock options in ICONplc; and is an employee of ICONplc, all outside the submitted work. D J Stein reports personal fees from Discovery Vitality, Johnson & Johnson, Kanna, L'Oreal, Lundbeck, Orion, Sanofi, Servier, Takeda, and Vistagen, all outside the submitted work. S J Tromans reports grants or contracts from Jazz Pharmaceuticals, NHS Digital, and the NIHR, all payments made to their institutions; leadership or fiduciary roles in board, society, committee or advocacy groups (paid or unpaid) with Neurodevelopmental Psychiatry Special Interest Group and Psychiatry of Intellectual Disability Faculty, is an Editorial Board Member for *Progress in Neurology, Advances in Mental Health and Intellectual Disability, Advances in Autism*, and *BMC Psychiatry*, and is the Editor of *Psychiatry of Intellectual Disability Across Cultures*.
