# Correction to *Lancet Public Health* 2025; 10: e189–202

**DOI:** 10.1016/S2468-2667(25)00177-X

**Published:** 2025-07-28

*GBD 2021 Suicide Collaborators. Global, regional, and national burden of suicide, 1990–2021: a systematic analysis for the Global Burden of Disease Study 2021.* Lancet Public Health *2025; **10:** e189–202*—For this Article, Kairi Kolves, Kewal Krishan, and David C Schwebel should have been included in the list of GBD 2021 Suicide Collaborators, and the name of collaborator Shivakumar KM was incorrect. These corrections have been made as of July 28, 2025.

