# Supplementary material for: Global, regional, and national burden of suicide, 1990–2021: a systematic analysis for the Global Burden of Disease Study 2021
Source: Lancet Public Health. 2025 Feb 19;10(3):e189–202. doi: 10.1016/S2468-2667(25)00006-4 (PMC11876099; doi:10.1016/S2468-2667(25)00006-4)
Supplement: Supplementary appendix 1 [file mmc1.pdf]

THE LANCET
Public Health

Supplementary appendix 1

This appendix formed part of the original submission and has been peer reviewed. We post it as supplied by the authors.

Supplement to: GBD 2021 Suicide Collaborators. Global, regional, and national burden of suicide, 1990–2021: a systematic analysis for the Global Burden of Disease Study 2021. *Lancet Public Health* 2025; published online Feb 19. https://doi.org/10.1016/S2468-2667(25)00006-4.

Appendix: supplementary methods and results for "Global, regional, and national burden of suicide 1990 to 2021: a systematic analysis for the Global Burden of Disease Study 2021"

Table of Contents

Section 1: List of abbreviations 4

Section 2: Summary of suicide modelling 5

Section 3: Guidelines for Accurate and Transparent Health Estimates Reporting (GATHER) compliance 6

GATHER Checklist 6

Section 4: References 7

Section 5: Supplemental tables and figures 8

Appendix Figure S1: Flowchart of the analytical strategy for the fatal suicide estimation process 8

Appendix Figure S2: Mean age of death from suicide from 1990 to 2021, all ages and both sexes combined, globally 9

Appendix Figure S3: Mean age of death due to suicide by country and sex in 2021 10

Appendix Figure S4: Selected covariates for suicide model, males 11

Appendix Figure S5: Selected covariates for suicide model, females 11

Appendix Figure S6: Standard betas from selected covariates for the female model 13

Appendix Figure S7: Standard betas from selected covariates for the male model 13

Appendix Figure S8: Deaths due to suicide coming from each garbage package in 2015 14

Appendix Figure S9: Source count of suicide data from 1980 to 2021 15

Appendix Figure S10: Age distribution for suicide death rate per 100 000 by country for males, 2017 to 2021 16

Appendix Figure S11: Age distribution for suicide death rate per 100 000 by country for females, 2017 to 2021 16

Appendix Figure S12: Female age-standardised death rate due to suicide by super-region from 1990-2021. 18

Appendix Figure S13: Male age-standardised death rate due to suicide by super-region from 1990-2021. 19

Appendix Table S1: Total number of suicide deaths, rate, and fraction of total deaths globally by GBD age group for males, females, and both sexes combined in 2021. 20

Appendix Table S2: Covariates for suicide, suicide by firearm, and suicide by other specified means in CODEm 21

Appendix Table S3: Mean age at the time of death from suicide for males, females, and both sexes combined, globally, and mean age at the time of death from suicide by firearms compared to suicide by other means. 23

Appendix Table S4: Mortality rate and rank of suicide by age groups 10-29, 30-49, 50-69, and 70+. Estimates are provided at the global and regional level, 2017 to 2021. 24

Appendix Table S5: Age-standardised mortality rate due to suicide in 1990, 2019, and 2021 and percent changes from 2019 to 2021, for males, females, and both sexes combined. Estimates provided at the global, super-region, regional, and national level. 26

Appendix Table S6: Mean age at the time of death due to suicide, for males, females, and both sexes combined in 1990 and 2021. Estimates provided at the global, super-region, regional, and national level. 39

Appendix Table S7: Number of deaths, incidence of suicide, and percent of suicide deaths using firearms, for males and females in 2021. Estimates provided at the global, super-region, regional, and national level. 51

Appendix Table S8: The rate and rank of suicides by age groups 10-29, 30-49, 50-69, and 70+ for males and females. Estimates provided at the global, super-region, regional, and national level....................................64

Appendix Table S9: Suicide by Firearm Age Standardized Mortality Rate per 100k in 2021. for males, females and both sexes combined. Estimates provided at the global, super-region, regional, and national level. 79

Section 1: List of abbreviations

Abbreviation	Full phrase
ASMR	Age-standardised mortality rate
CoD	causes of death
CODEm	Cause of Death Ensemble modelling
DisMod-MR	disease model-Bayesian meta-regression
GATHER	Guidelines for Accurate and Transparent Health Estimates Reporting
GBD	Global Burden of Diseases, Injuries, and Risk Factors Study
GHDx	Global Health Data Exchange
ICD	International Classification of Diseases
IHME	Institute for Health Metrics and Evaluation
SDG	Sustainable Development Goals
SDI	Socio-demographic Index
SEV	summary exposure value
ST-GPR	spatiotemporal Gaussian process regression
UI	uncertainty interval
UN	United Nations
VA	verbal autopsy
VR	vital registration
WHO	World Health Organization

Section 2: Summary of suicide modelling

Suicide is defined as “the act of taking one’s own life”. It does not include “assisted suicide” or “euthanasia”.[1] The International Statistical Classification of Diseases (ICD-10) codes for suicide are X60-X84. Data for suicide are acquired from VR data, verbal autopsy, and mortality surveillance. Estimates for suicide were modelled exclusively in CODEm using covariates described in appendix table S2.

Data for suicide included vital registration (VR) data, survey data, verbal autopsy (VA) data, and surveillance data. Suicide has two most-detailed models in the cause of death ensemble model (CODEm): suicide by firearm and suicide by other specified means.

Suicide mortality was modelled in the cause of death ensemble model (CODEm) platform. CODEm is a Bayesian statistical model and uses spatial priors from a hierarchical structure to inform the mortality models. CODEm is based on five general principles: identifying all available data, maximising the comparability and quality of the dataset, and developing a diverse set of plausible models. CODEm produces a large suite of models based on either cause fraction or mortality rate, uses linear and space-time Gaussian process regression (ST-GPR), and a covariate selection process. Each sub-model is evaluated using out-of-sample predictive validity. Thirty percent of the data are excluded from the initial model fits and 15% are used to evaluate component models and 15% used to build the ensembles. The sub-models are ranked using 15% of the data based on their out-of-sample predictive validity. The proportion weighting of the ensemble sub-models is evaluated using the remaining 15% of the hold-out data. This weighting scheme evaluates ensemble models that are built with ranked sub-models contributing proportionally more or fewer draws to the final ensemble. The final ensemble model is evaluated against other ensemble models using the same fit statistics (in-sample, out-of-sample root mean squared error and data coverage). Detailed information on this process can be found in published studies.[2–4]

Covariates for suicide were selected independently for each sub-model and the selection is based on an algorithm that captures biologically plausible relationships between the covariates and interpersonal violence mortality and provides a diversity of possible models. A list of covariates that the models select from is provided in appendix section 5, table S2. For every covariate, the direction of effect and a level of biologic proximity to suicide mortality was defined by the modeller. Each model includes all combinations of covariates if the direction of effect is along the assumed direction and the coefficient is significant at the $p < 0{\cdot}05$ level.

Suicide mortality is estimated for 23 age groups, 204 locations, both sexes, and every year from 1980-2021 using separate models for males and females. Data-rich and data-poor geographic locations were modelled separately, and these models were then hybridised for a global model. This was to maintain proper uncertainty in the models where trusted data on causes of death exist. For a detailed description of the input data coverage, completeness, and reliability of the cause of death data in GBD 2021, please refer to the scoring system described in the GBD 2021 Causes of Death manuscript.[3] Suicide mortality estimates are then squeezed into an overall mortality envelope by age/sex/location/year in a process called CoDCorrect. This step is to ensure internal consistency among causes of death and that the sum of cause-specific mortality is the same as the estimated all-cause mortality.

A detailed flowchart showing the analytical strategy for the fatal suicide estimation process is provided in appendix section 5, figure S1.

Section 3: Guidelines for Accurate and Transparent Health Estimates Reporting (GATHER) compliance

This study complies with GATHER recommendations. The GATHER recommendations can be found on the GATHER website.

GATHER Checklist

Item #	Checklist item	Reported location on page #
Objectives and funding		
1	Define the indicator(s), populations (including age, sex, and geographic entities), and time period(s) for which estimates were made.	2
2	List the funding sources for the work.	2
Data Inputs		
For all data inputs from multiple sources that are synthesized as part of the study:		
3	Describe how the data were identified and how the data were accessed.	5
4	Specify the inclusion and exclusion criteria. Identify all ad-hoc exclusions.	5
5	Provide information on all included data sources and their main characteristics. For each data source used, report reference information or contact name/institution, population represented, data collection method, year(s) of data collection, sex and age range, diagnostic criteria or measurement method, and sample size, as relevant.	5
6	Identify and describe any categories of input data that have potentially important biases (e.g., based on characteristics listed in item 5).	5
For data inputs that contribute to the analysis but were not synthesized as part of the study:		
7	Describe and give sources for any other data inputs.	5
For all data inputs:		
8	Provide all data inputs in a file format from which data can be efficiently extracted (e.g., a spreadsheet rather than a PDF), including all relevant meta-data listed in item 5. For any data inputs that cannot be shared because of ethical or legal reasons, such as third-party ownership, provide a contact name or the name of the institution that retains the right to the data.	Data inputs and/or contact information available at https://ghdx.healthdata.org/gbd-2021/sources
Data analysis		
9	Provide a conceptual overview of the data analysis method. A diagram may be helpful.	Appendix p 8
10	Provide a detailed description of all steps of the analysis, including mathematical formulae. This description should cover, as relevant, data cleaning, data pre-processing, data adjustments and weighting of data sources, and mathematical or statistical model(s).	5-6
11	Describe how candidate models were evaluated and how the final model(s) were selected.	5-6
12	Provide the results of an evaluation of model performance, if done, as well as the results of any relevant sensitivity analysis.	N/A
13	Describe methods for calculating uncertainty of the estimates. State which sources of uncertainty were, and were not, accounted for in the uncertainty analysis.	5-6
14	State how analytic or statistical source code used to generate estimates can be accessed.	5-6
Results and Discussion		
15	Provide published estimates in a file format from which data can be efficiently extracted.	Published estimates are available in the main text results section and in the supplementary appendix. CSV files are available upon request to the corresponding author
16	Report a quantitative measure of the uncertainty of the estimates (e.g. uncertainty intervals).	7-9
17	Interpret results in light of existing evidence. If updating a previous set of estimates, describe the reasons for changes in estimates.	7-9
18	Discuss limitations of the estimates. Include a discussion of any modelling assumptions or data limitations that affect interpretation of the estimates.	11-12

Section 4: References

1 WHO. Preventing suicide: a global imperative. Geneva: World Health Organization, 2014 https://iris.who.int/handle/10665/131056 (accessed July 23, 2024).

2 Foreman KJ, Lozano R, Lopez AD, Murray CJ. Modeling causes of death: an integrated approach using CODEm. *Popul Health Metr* 2012; **10**: 1.

3 GBD 2021 Causes of Death Collaborators. Global burden of 288 causes of death and life expectancy decomposition in 204 countries and territories and 811 subnational locations, 1990-2021: a systematic analysis for the Global Burden of Disease Study 2021. *Lancet* 2024; **403**: 2100–32.

4 GBD 2021 Diseases and Injuries Collaborators. Global incidence, prevalence, years lived with disability (YLDs), disability-adjusted life-years (DALYs), and healthy life expectancy (HALE) for 371 diseases and injuries in 204 countries and territories and 811 subnational locations, 1990-2021: a systematic analysis for the Global Burden of Disease Study 2021. *Lancet* 2024; **403**: 2133–61.

Section 5: Supplemental tables and figures

Appendix Figure S1: Flowchart of the analytical strategy for the fatal suicide estimation process

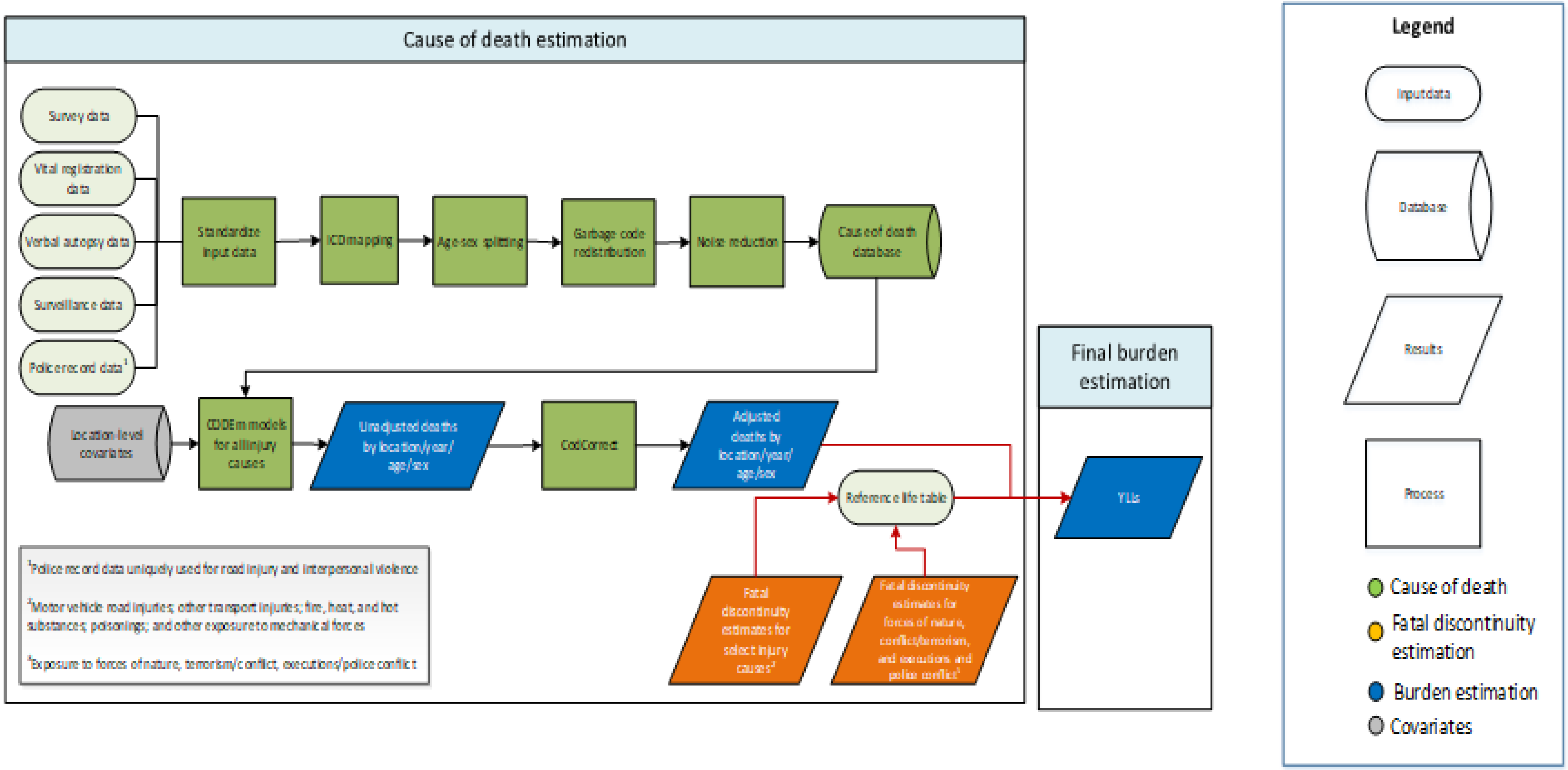

Appendix Figure S2: Mean age of death from suicide from 1990 to 2021, all ages and both sexes combined, globally

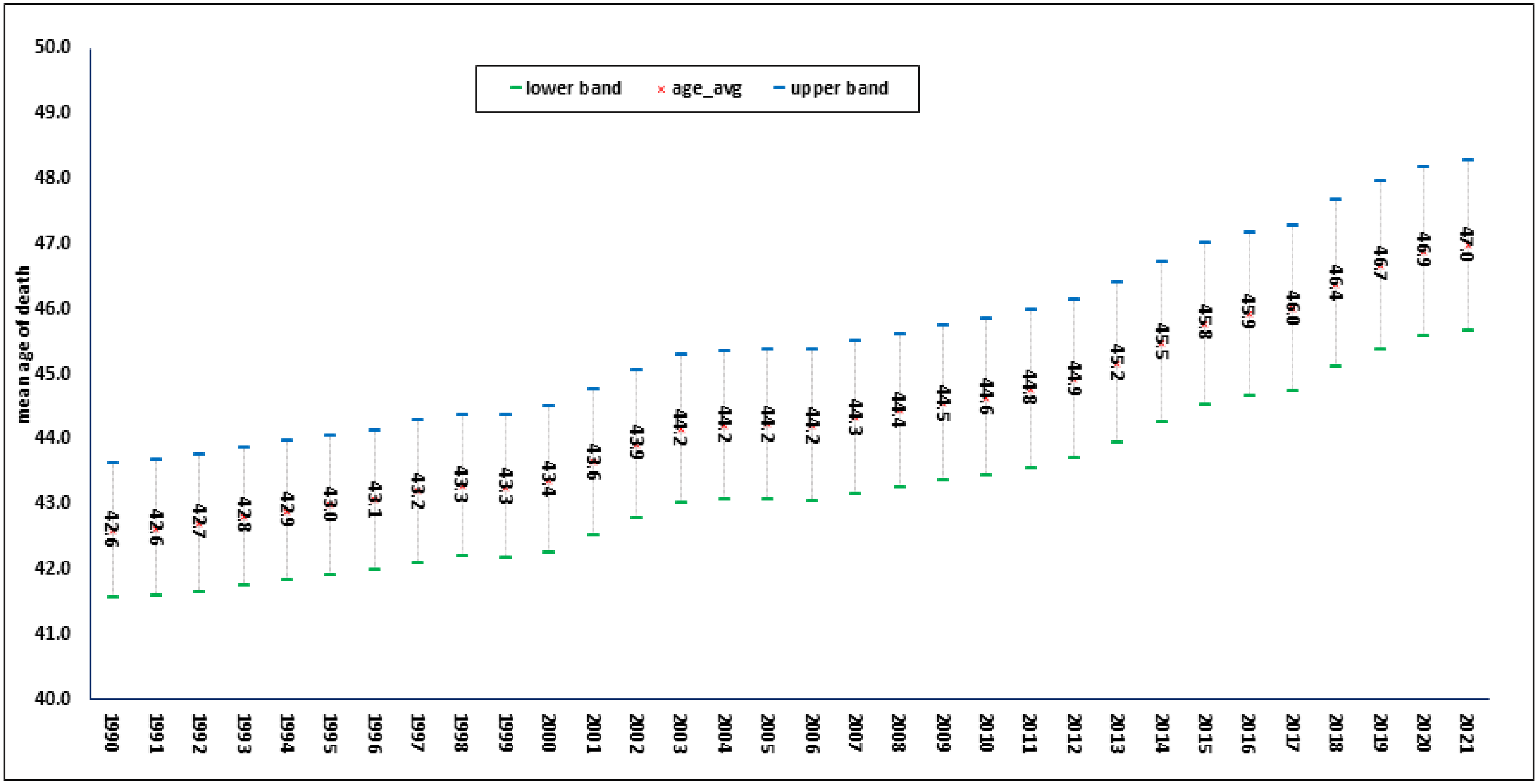

Appendix Figure S3: Mean age of death due to suicide by country and sex in 2021

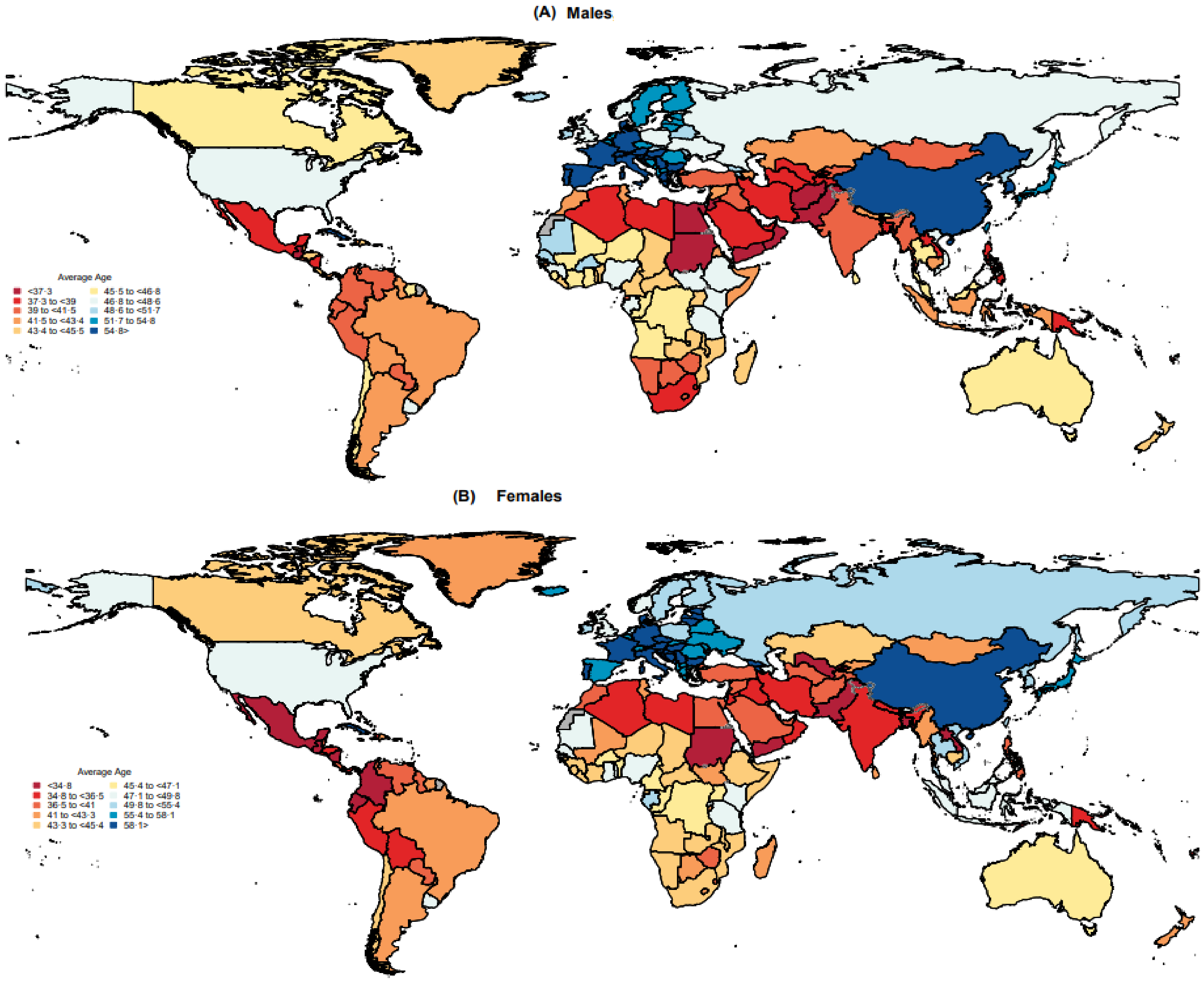

Appendix Figure S4: Selected covariates for suicide model, males

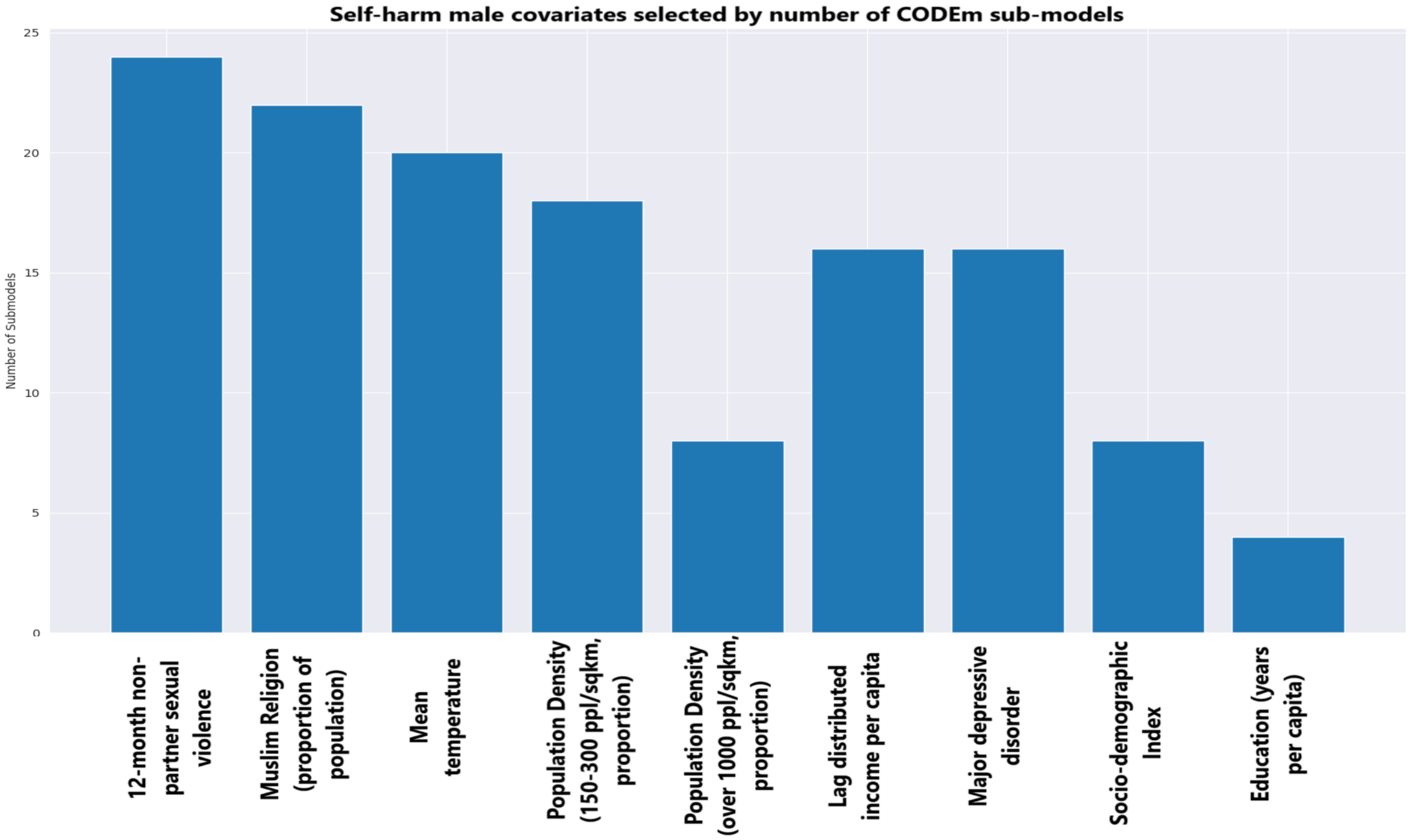

Appendix Figure S5: Selected covariates for suicide model, females

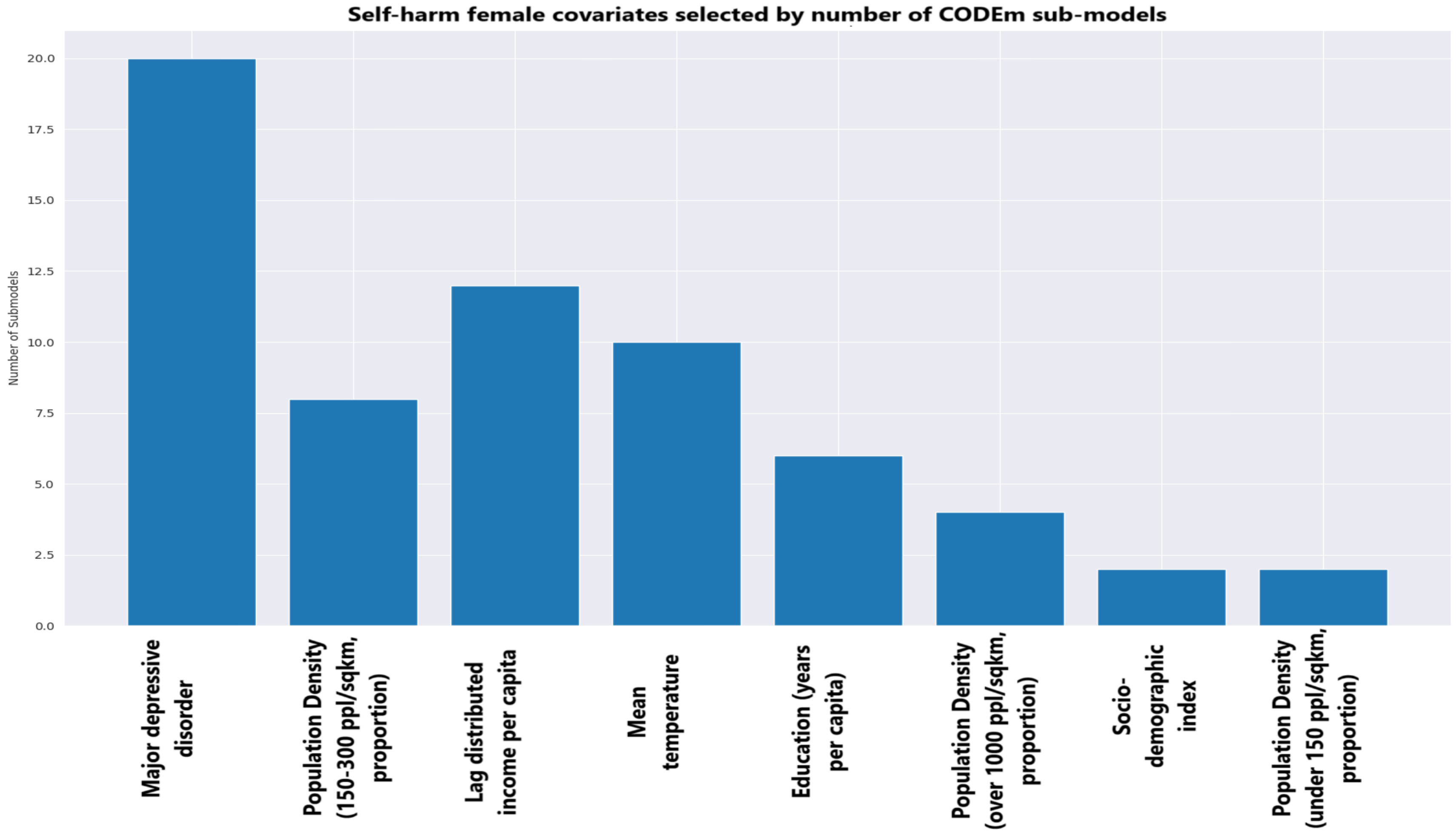
Self-harm female covariates selected by number of CODEm sub-models
Number of Submodels
20.0
17.5
15.0
12.5
10.0
7.5
5.0
2.5
0.0
Major depressive disorder
Population Density (150-300 ppl/sqkm, proportion)
Lag distributed income per capita
Mean temperature
Education (years per capita)
Population Density (over 1000 ppl/sqkm, proportion)
Socio-demographic index
Population Density (under 150 ppl/sqkm, proportion)

Appendix Figure S6: Standard betas from selected covariates for the female model

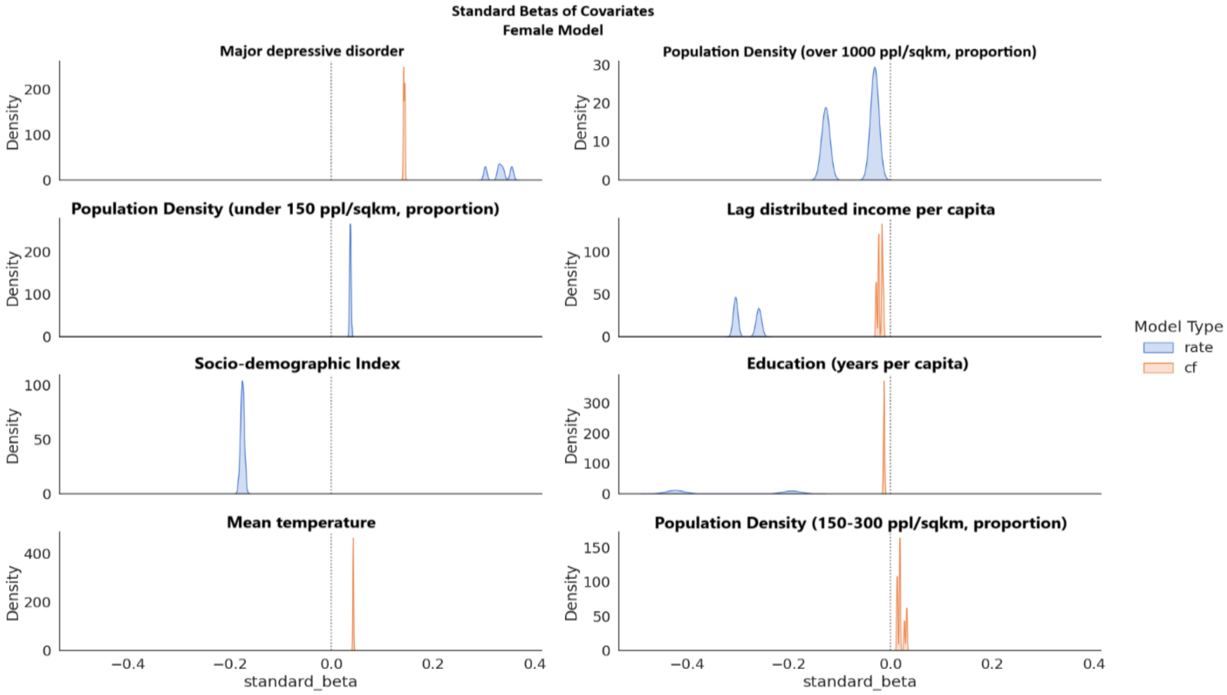

Appendix Figure S7: Standard betas from selected covariates for the male model

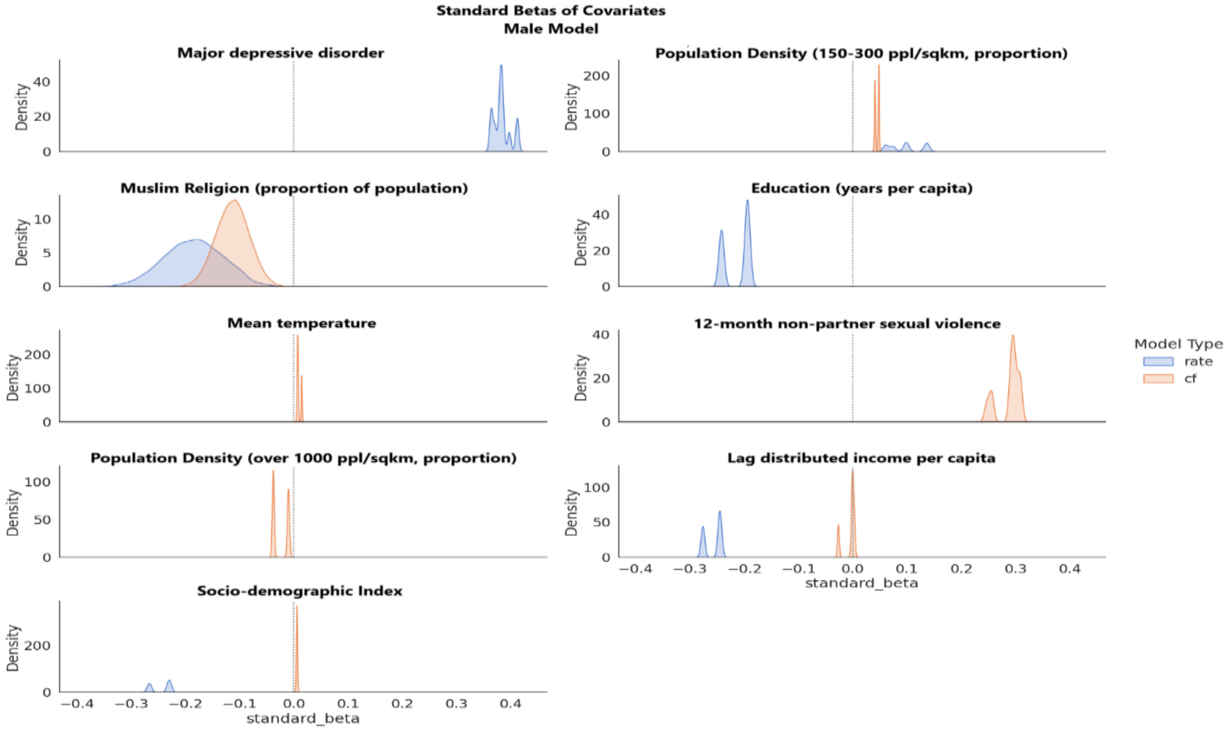

Appendix Figure S8: Deaths due to suicide coming from each garbage package in 2015

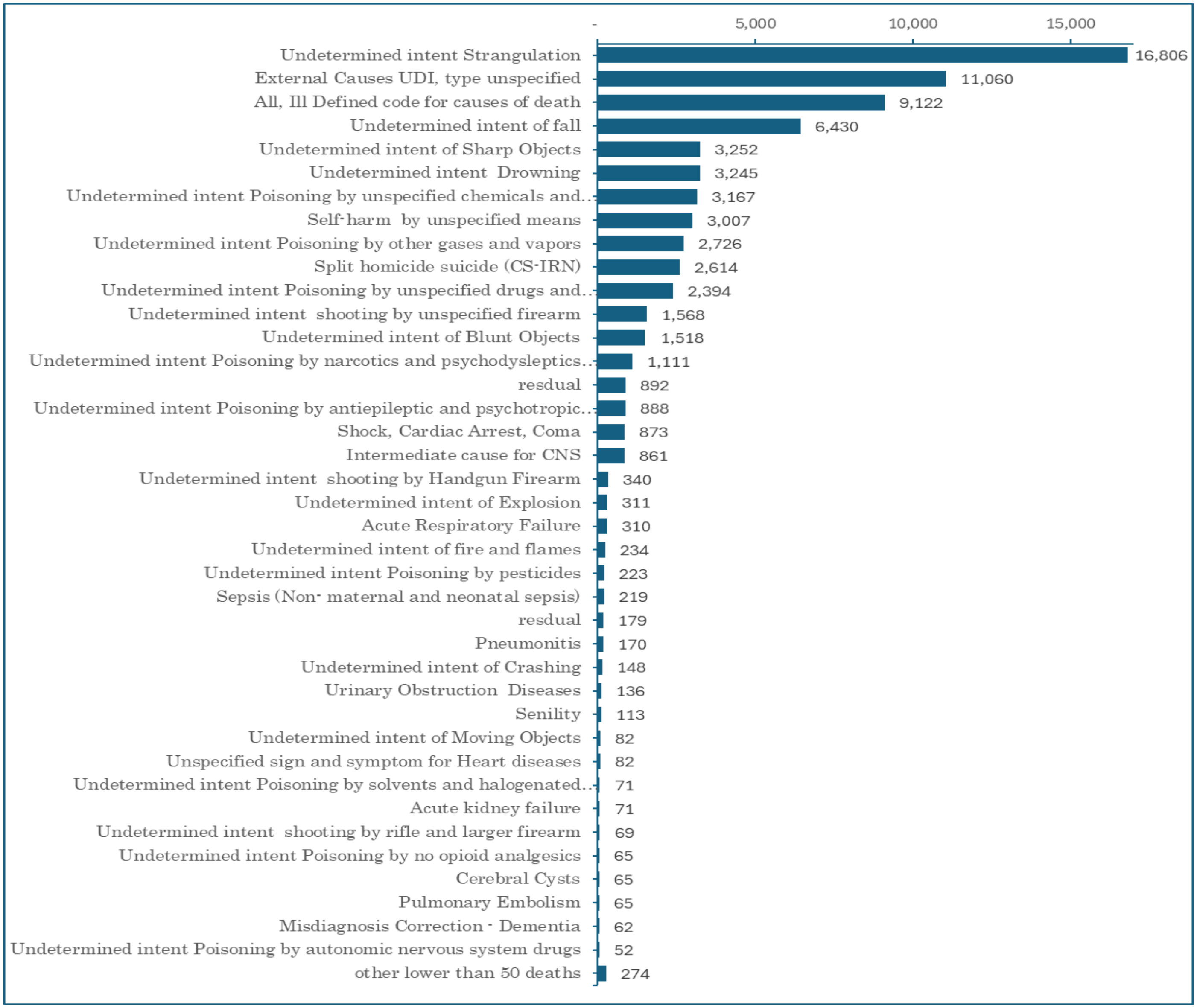

Appendix Figure S9: Source count of suicide data from 1980 to 2021

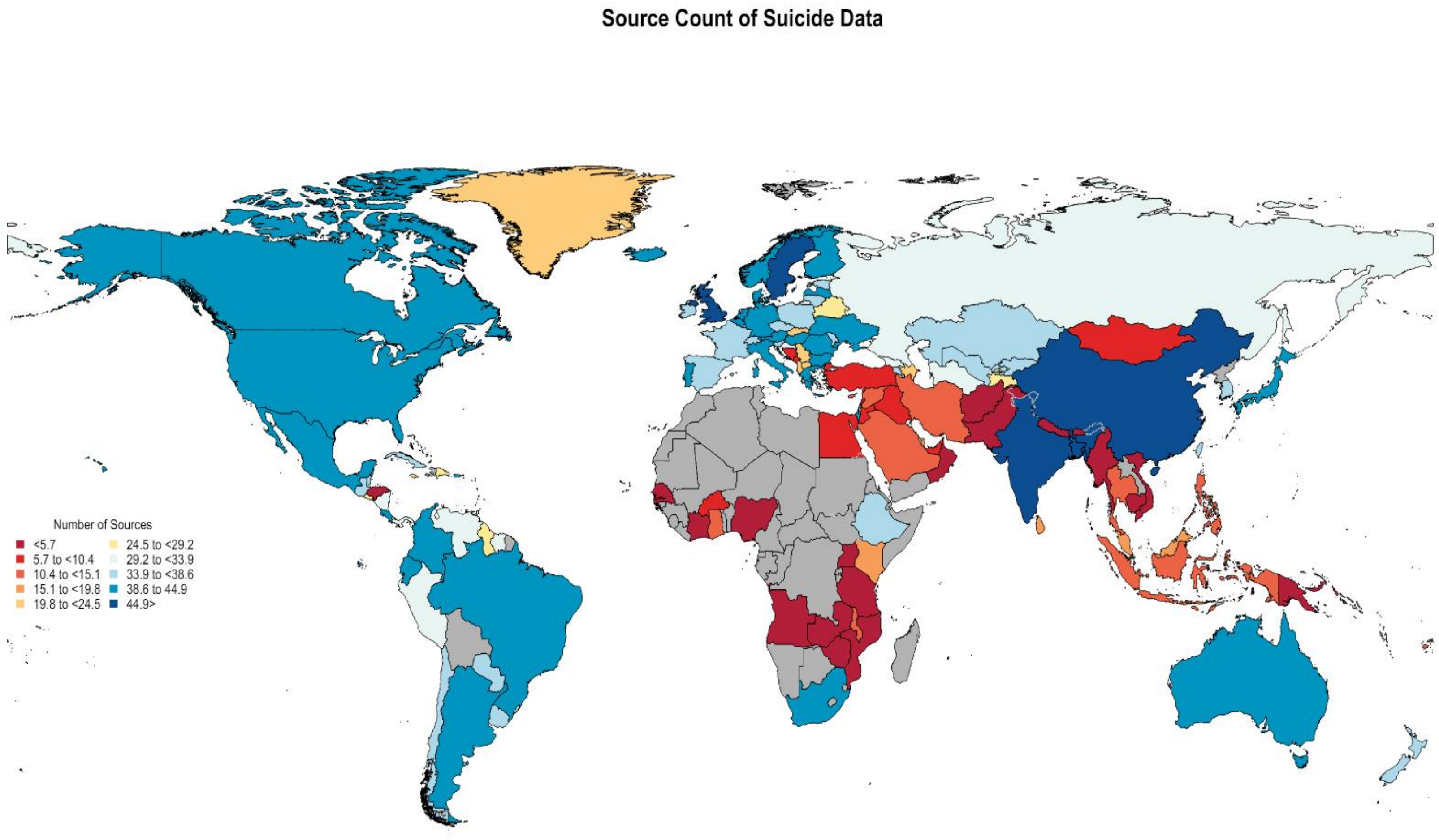

Appendix Figure S10: Age distribution for suicide death rate per 100 000 by country for males, 2017 to 2021

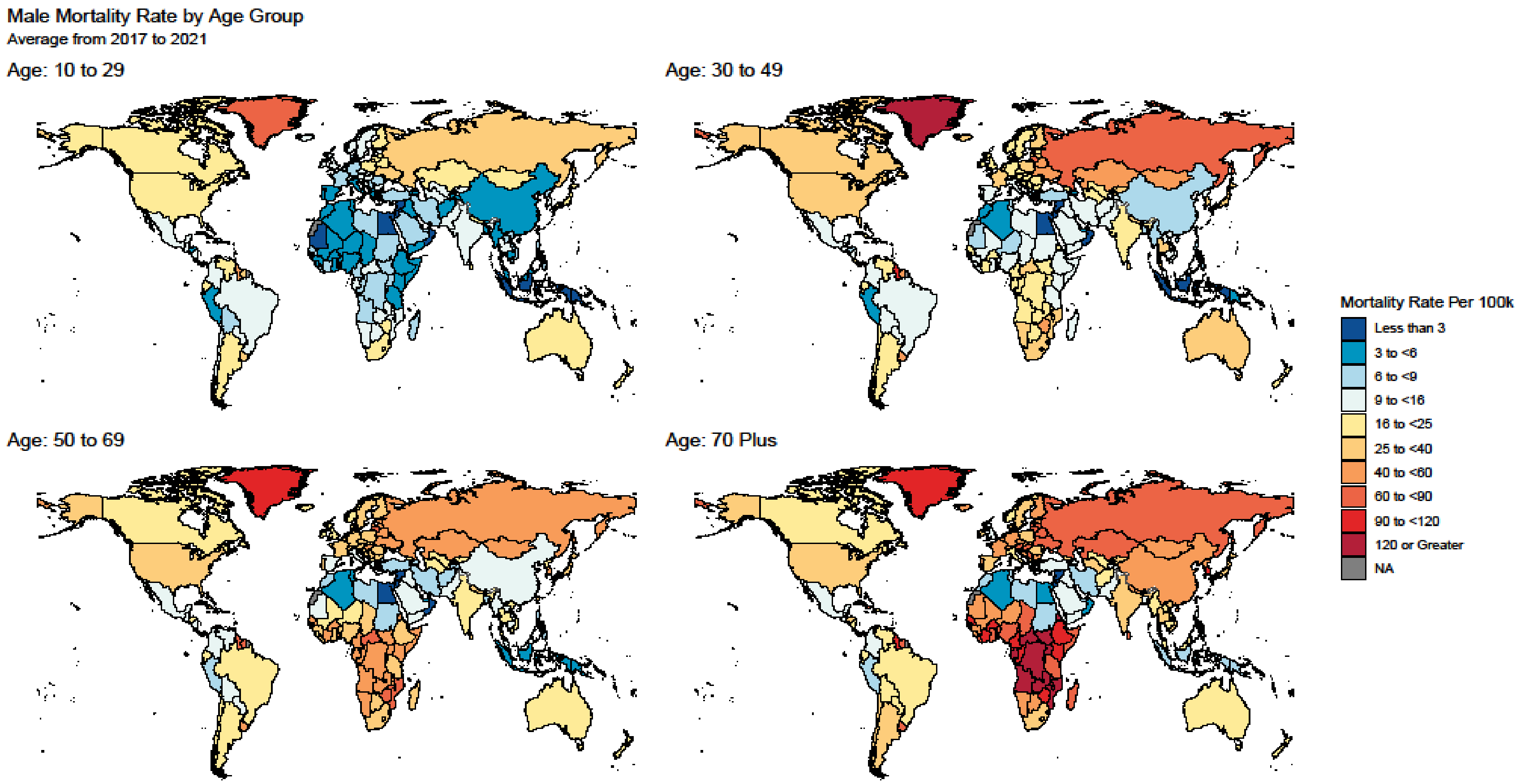

Appendix Figure S11: Age distribution for suicide death rate per 100 000 by country for females, 2017 to 2021

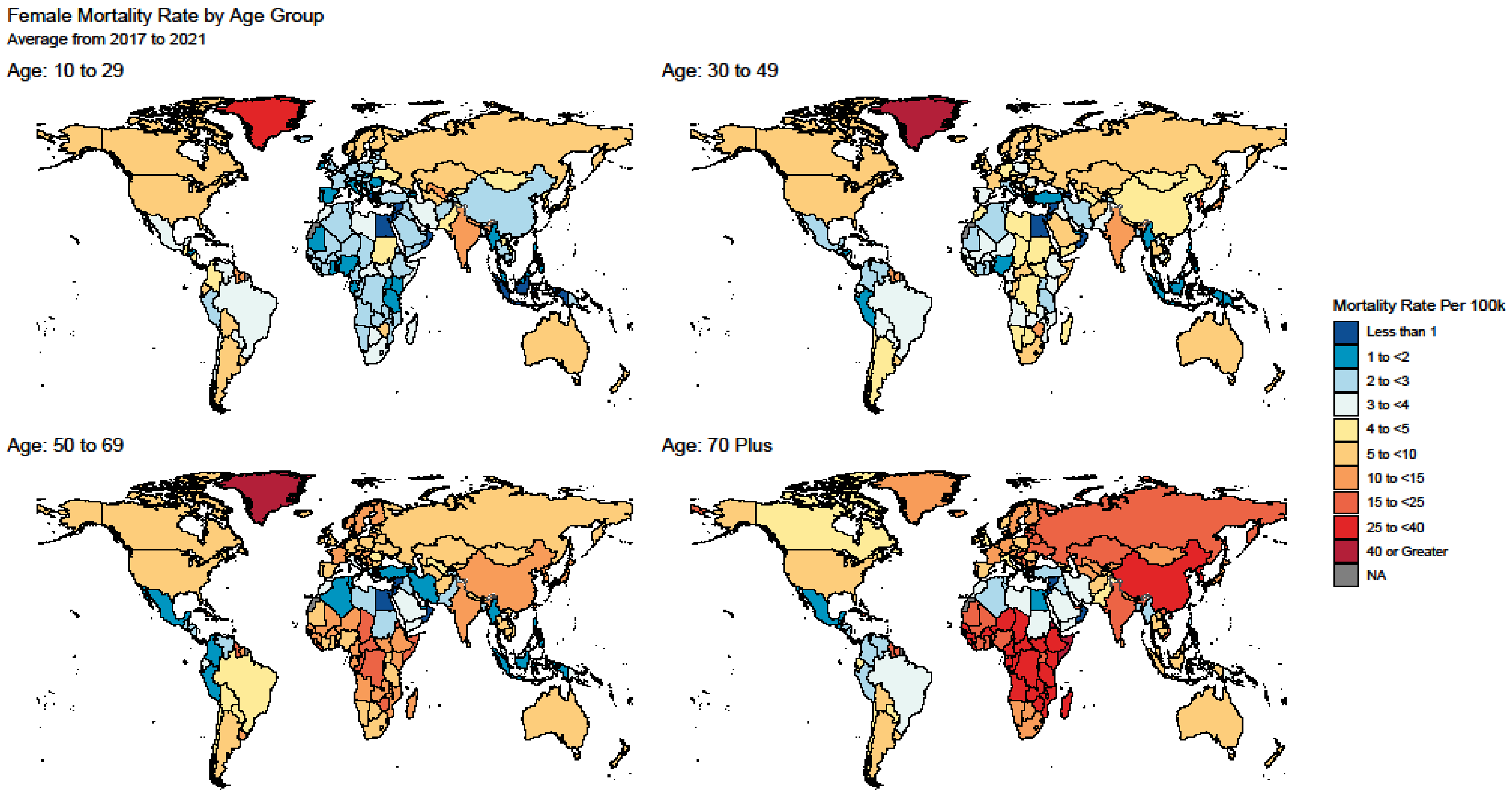

Female Mortality Rate by Age Group
Average from 2017 to 2021
Age: 10 to 29
Age: 30 to 49
Age: 50 to 69
Age: 70 Plus
Mortality Rate Per 100k
Less than 1
1 to <2
2 to <3
3 to <4
4 to <5
5 to <10
10 to <15
15 to <25
25 to <40
40 or Greater
NA

Appendix Figure S12: Female age-standardised death rate due to suicide by super-region from 1990-2021.

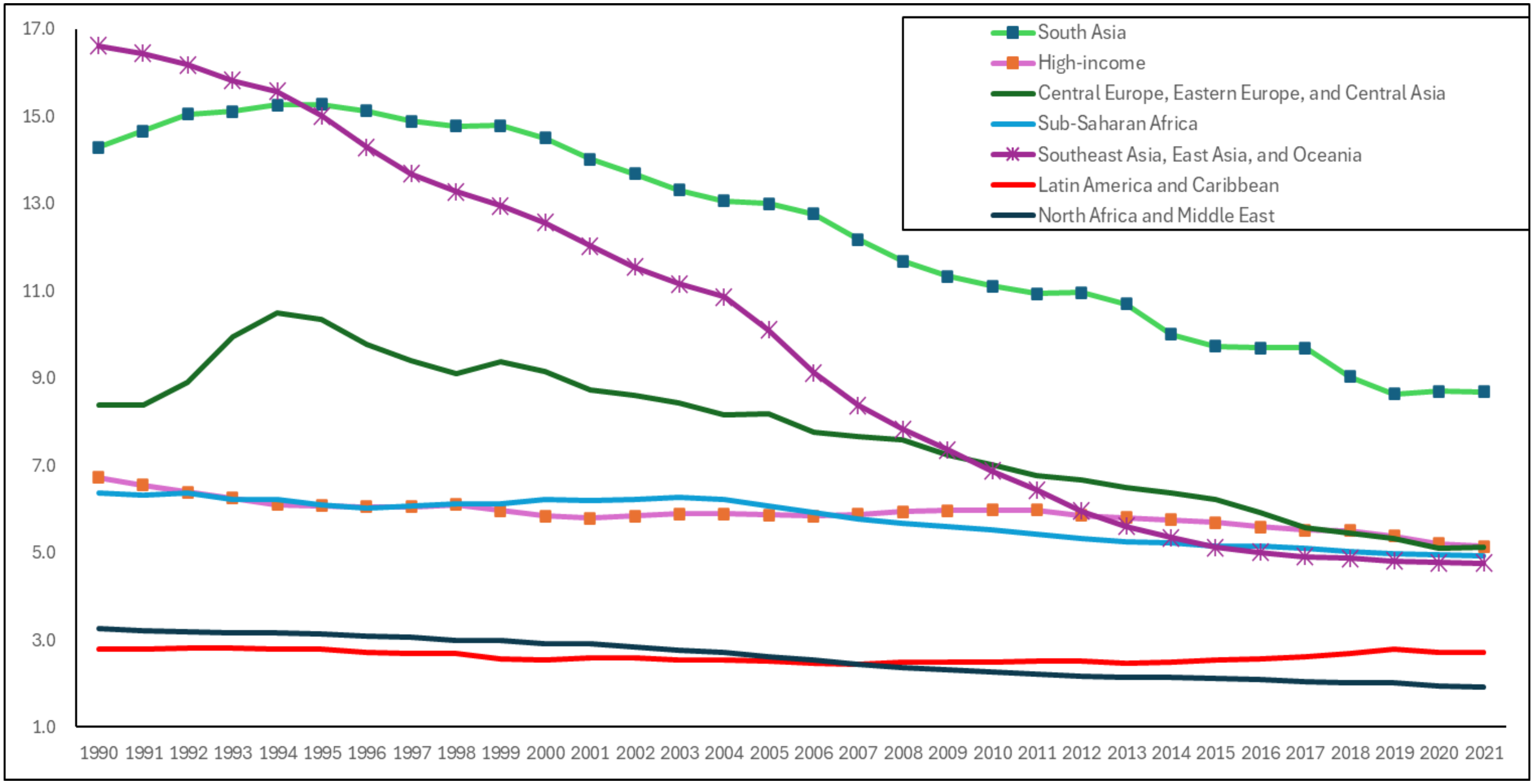

Appendix Figure S13: Male age-standardised death rate due to suicide by super-region from 1990-2021.

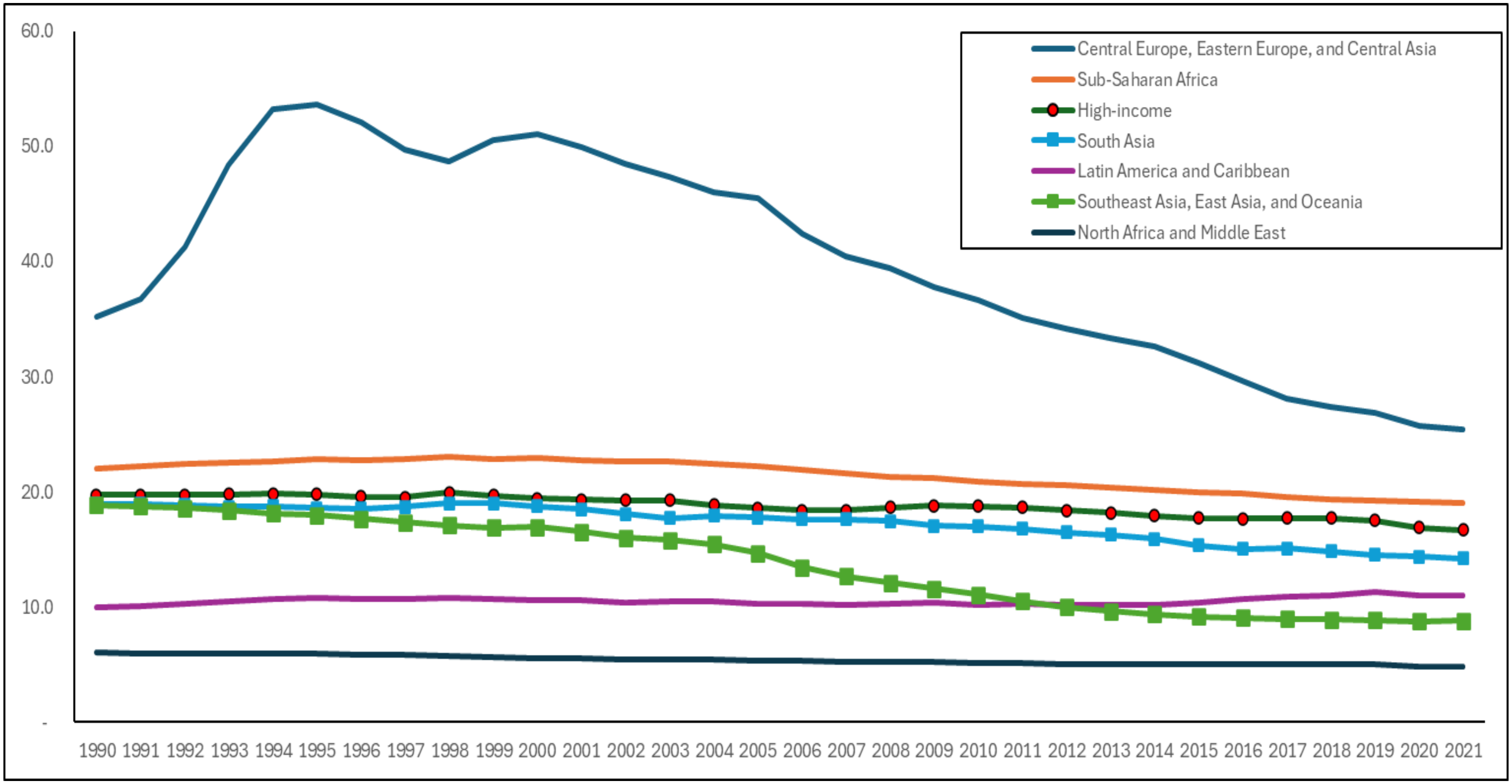

Appendix Table S1: Total number of suicide deaths, rate, and fraction of total deaths globally by GBD age group for males, females, and both sexes combined in 2021.

	Deaths			Mortality Rate Per 100k			Fraction of Total Deaths		
Age Group	Both	Male	Female	Both	Male	Female	Both	Male	Female
All Ages	746000 (692000–800000)	519000 (485000–556000)	227000 (200000–255000)	9·46 (8·77–10·1)	13·1 (12·3–14·1)	5·78 (5·10–6·49)	0·0110 (0·0102–0·0117)	0·0138 (0·0129–0·0147)	0·00753 (0·00660–0·00838)
10 to 14	7780 (6790–9380)	4520 (3930–5470)	3260 (2740–4000)	1·17 (1·02–1·41)	1·31 (1·14–1·59)	1·01 (0·848–1·24)	0·0256 (0·0226–0·0308)	0·0266 (0·0236–0·0325)	0·0244 (0·0204–0·0301)
15 to 19	41900 (38600–46400)	23000 (20900–25300)	19000 (16800–21700)	6·72 (6·18–7·44)	7·17 (6·54–7·89)	6·24 (5·53–7·16)	0·0742 (0·0686–0·0825)	0·0676 (0·0613–0·0743)	0·0843 (0·0750–0·0964)
20 to 24	63200 (58200–68200)	41800 (38400–45000)	21500 (18400–24500)	10·6 (9·74–11·4)	13·8 (12·7–14·8)	7·31 (6·27–8·34)	0·0857 (0·0796–0·0915)	0·0919 (0·0848–0·0981)	0·0757 (0·0656–0·0862)
25 to 29	66600 (61900–71500)	46900 (43500–50000)	19700 (17000–22800)	11·3 (10·5–12·2)	15·8 (14·6–16·8)	6·77 (5·83–7·83)	0·0780 (0·0732–0·0836)	0·0881 (0·0824–0·0943)	0·0613 (0·0529–0·0695)
30 to 34	66300 (61500–71600)	48900 (45600–53300)	17300 (14500–19800)	11·0 (10·2–11·8)	16·0 (14·9–17·4)	5·80 (4·84–6·62)	0·0593 (0·0553–0·0636)	0·0681 (0·0641–0·0732)	0·0433 (0·0358–0·0486)
35 to 39	62000 (57000–66400)	46000 (42600–49300)	16000 (13800–18100)	11·1 (10·2–11·8)	16·3 (15·0–17·4)	5·77 (4·96–6·53)	0·0448 (0·0414–0·0474)	0·0515 (0·0478–0·0548)	0·0327 (0·0280–0·0362)
40 to 44	58500 (54200–62800)	44200 (41200–47600)	14300 (12100–16100)	11·7 (10·8–12·5)	17·5 (16·3–18·9)	5·75 (4·89–6·49)	0·0326 (0·0301–0·0345)	0·0379 (0·0349–0·0402)	0·0227 (0·0194–0·0252)
45 to 49	55400 (50800–59500)	41000 (38000–44400)	14500 (12100–16200)	11·7 (10·7–12·6)	17·2 (16·0–18·7)	6·14 (5·13–6·87)	0·0244 (0·0224–0·0260)	0·0280 (0·0260–0·0300)	0·0179 (0·0150–0·0197)
50 to 54	55700 (51100–59900)	39900 (36900–43200)	15800 (13000–18100)	12·5 (11·5–13·5)	18·0 (16·6–19·5)	7·07 (5·82–8·10)	0·0182 (0·0170–0·0195)	0·0204 (0·0193–0·0218)	0·0144 (0·0121–0·0161)
55 to 59	55500 (50600–60000)	39600 (36200–42600)	15900 (13700–18000)	14·0 (12·8–15·2)	20·3 (18·6–21·9)	7·89 (6·80–8·97)	0·0135 (0·0122–0·0144)	0·0154 (0·0141–0·0164)	0·0103 (0·00877–0·0114)
60 to 64	45500 (41700–49000)	32200 (29200–34800)	13300 (11200–15000)	14·2 (13·0–15·3)	20·7 (18·8–22·4)	8·09 (6·82–9·13)	0·00883 (0·00804–0·00939)	0·0101 (0·00921–0·0107)	0·00675 (0·00570–0·00755)
65 to 69	44000 (39800–48200)	29800 (26700–32600)	14300 (11800–16600)	16·0 (14·4–17·5)	22·6 (20·3–24·7)	9·90 (8·16–11·5)	0·00666 (0·00602–0·00715)	0·00750 (0·00678–0·00806)	0·00540 (0·00449–0·00606)
70 to 74	40200 (35800–44100)	26800 (23500–29500)	13400 (11200–15300)	19·5 (17·4–21·4)	27·8 (24·4–30·6)	12·2 (10·2–14·0)	0·00535 (0·00476–0·00574)	0·00616 (0·00543–0·00655)	0·00424 (0·00356–0·00476)
75 to 79	32800 (28500–36200)	21700 (19300–23900)	11000 (9220–12900)	24·8 (21·6–27·4)	36·4 (32·2–40·0)	15·3 (12·8–17·9)	0·00447 (0·00390–0·00484)	0·00535 (0·00471–0·00576)	0·00337 (0·00283–0·00384)
80 to 84	26300 (22800–29100)	17000 (14700–18700)	9290 (7440–10900)	30·0 (26·0–33·3)	46·3 (40·2–51·0)	18·2 (14·6–21·3)	0·00333 (0·00292–0·00362)	0·00431 (0·00378–0·00465)	0·00235 (0·00189–0·00270)
85 to 89	17300 (14500–19400)	11200 (9710–12200)	6080 (4590–7340)	37·8 (31·8–42·4)	64·9 (56·3–70·8)	21·3 (16·1–25·8)	0·00262 (0·00223–0·00289)	0·00361 (0·00313–0·00389)	0·00174 (0·00131–0·00204)
90 to 94	6080 (5020–6790)	3770 (3230–4160)	2310 (1730–2720)	34·0 (28·1–38·0)	64·7 (55·4–71·4)	19·2 (14·3–22·6)	0·00154 (0·00127–0·00169)	0·00240 (0·00204–0·00260)	0·000969 (0·000731–0·00113)
95 plus	1320 (1020–1500)	739 (591–821)	580 (424–689)	24·2 (18·7–27·5)	48·9 (39·1–54·3)	14·7 (10·8–17·5)	0·000809 (0·000628–0·000915)	0·00152 (0·00122–0·00169)	0·000506 (0·000371–0·000593)

Appendix Table S2: Covariates for suicide, suicide by firearm, and suicide by other specified means in CODEm

**Suicide**		
**Level**	**Covariate**	**Direction**
**1**	12-month non-partner sexual violence	1
**1**	Liters of alcohol consumed per capita	1
**1**	Major depressive disorder	1
**1**[i]	Muslim Religion (proportion of population)	1
**1**	Population-weighted mean temperature	1
**2**	Healthcare access and quality index	-1
**2**	Population Density (150-300 ppl/sqkm, proportion)	1
**2**	Population Density (300-500 ppl/sqkm, proportion)	-1
**2**	Population Density (500-1000 ppl/sqkm, proportion)	-1
**2**	Population Density (over 1000 ppl/sqkm, proportion)	-1
**2**	Population Density (under 150 ppl/sqkm, proportion)	1
**3**	Education (years per capita)	-1
**3**	LDI (I$ per capita)	-1
**3**	Socio-demographic Index	-1
**Suicide by firearm**		
**Level**	Covariate	Direction
**1**	12-month non-partner sexual violence	1
**1**	Liters of alcohol consumed per capita	1
**1**	Log-transformed SEV scalar: Self Harm	1
**1**	Major depressive disorder	1
**1**	Population-weighted mean temperature	1
**2**	Healthcare access and quality index	-1
**2**	Population Density (150-300 ppl/sqkm, proportion)	1
**2**	Population Density (300-500 ppl/sqkm, proportion)	-1
**2**	Population Density (500-1000 ppl/sqkm, proportion)	-1
**2**	Population Density (over 1000 ppl/sqkm, proportion)	-1
**2**	Population Density (under 150 ppl/sqkm, proportion)	1
**3**	Education (years per capita)	-1
**3**	LDI (I$ per capita)	-1
**3**	Socio-demographic Index	-1
**Suicide by other specified means**		

Level	Covariate	Direction
**1**	12-month non-partner sexual violence	1
**1**	Liters of alcohol consumed per capita	1
**1**	Log-transformed SEV scalar: Self Harm	1
**1**	Major depressive disorder	1
**1**	Population-weighted mean temperature	1
**2**	Healthcare access and quality index	-1
**2**	Population Density (150-300 ppl/sqkm, proportion)	1
**2**	Population Density (300-500 ppl/sqkm, proportion)	-1
**2**	Population Density (500-1000 ppl/sqkm, proportion)	-1
**2**	Population Density (over 1000 ppl/sqkm, proportion)	-1
**2**	Population Density (under 150 ppl/sqkm, proportion)	1
**3**	Education (years per capita)	-1
**3**	LDI (I$ per capita)	-1
**3**	Socio-demographic Index	-1

Appendix Table S3: Mean age at the time of death from suicide for males, females, and both sexes combined, globally, and mean age at the time of death from suicide by firearms compared to suicide by other means.

Average Age of Death by Cause				
**Location**	**Sex**	**suicide**	**suicide by firearm**	**suicide by other means**
Global	Both	47·0 (43·3–50·3)	46·5 (39·5–53·4)	47·0 (43.5–50.7)
Global	Male	47·0 (43·5–50·6)	46·9 (39·3–54·6)	47·0 (43·0–50·9)
Global	Female	46·9 (41·2–52·8)	43·8 (34·8–52·1)	47·0 (41·3–53·3)

Appendix Table S4: Mortality rate and rank of suicide by age groups 10-29, 30-49, 50-69, and 70+. Estimates are provided at the global and regional level, 2017 to 2021.

	Male: 10 to 29		Female: 10 to 29		Male: 30 to 49		Female: 30 to 49		Male: 50 to 69		Female:50 to 69		Male: 70 plus		Female: 70 plus	
**Location**	**Mortality Rate**	**Rank**	**Mortality Rate**	**Rank**	**Mortality Rate**	**Rank**	**Mortality Rate**	**Rank**	**Mortality Rate**	**Rank**	**Mortality Rate**	**Rank**	**Mortality Rate**	**Rank**	**Mortality Rate**	**Rank**
Global	9·49 (8·81–10·2)	3	5·31 (4·62–6·04)	3	17·1 (15·9–18·2)	7	6·01 (5·12–6·69)	11	20·8 (19·1–22·3)	17	8·35 (7·05–9·42)	25	37·9 (33·4–41·4)	28	15·6 (12·7–18·0)	47
Andean Latin America	8·46 (6·98–10·1)	3	3·96 (3·09–4·99)	4	9·49 (7·60–11·6)	9	2·41 (1·82–3·02)	19	11·3 (8·79–13·8)	19	2·32 (1·75–2·95)	43	14·1 (11·0–17·0)	44	3·37 (2·54–4·26)	70
Australasia	18·0 (16·5–19·3)	1	5·87 (5·40–6·33)	1	27·5 (25·0–29·9)	1	8·13 (7·35–8·93)	2	23·5 (21·2–25·9)	6	7·62 (6·77–8·58)	14	21·2 (18·3–23·9)	40	6·14 (4·93–7·14)	62
Caribbean	7·97 (6·42–9·74)	3	3·46 (2·37–4·77)	6	18·0 (15·3–21·2)	8	4 (3·14–5·01)	16	26·0 (22·8–29·7)	16	6·59 (5·69–7·62)	26	48·5 (42·6–54·6)	22	9·56 (8·28–10·9)	55
Central Asia	12·7 (11·8–13·7)	2	6·41 (5·83–7·07)	1	25·4 (23·5–27·3)	4	4·82 (4·38–5·33)	9	23·1 (21·1–25·2)	16	4·73 (4·32–5·20)	30	30·6 (27·9–33·4)	25	11·7 (10·5–12·7)	37
Central Europe	12·0 (11·5–12·6)	2	2·25 (2·10–2·42)	2	25·5 (24·3–26·6)	2	4·32 (4·04–4·70)	8	34·2 (32·3–36·1)	14	7·03 (6·53–7·66)	24	43·5 (40·0–46·5)	25	9·84 (8·81–10·8)	46
Central Latin America	12·5 (11·6–13·4)	3	3·68 (3·40–3·96)	3	15·4 (14·4–16·5)	9	2·75 (2·53–2·98)	18	13·0 (11·9–14·1)	16	2·01 (1·84–2·18)	50	15·8 (14·3–17·1)	43	2·03 (1·80–2·21)	80
Central Sub-Saharan Africa	7·38 (4·88–11·5)	9	2·53 (1·52–4·26)	13	20·5 (14·0–29·8)	10	4·22 (2·73–6·57)	20	51·9 (36·0–73·5)	15	15·1 (9·38–22·7)	24	122 (85·9–172)	16	29·7 (19·2–44·1)	35
East Asia	4·61 (3·76–5·66)	3	2·83 (2·20–4·06)	2	8·44 (6·73–10·7)	8	5 (3·80–7·14)	6	14·4 (11·0–18·6)	15	10·3 (7·83–13·7)	16	48·2 (38·2–59·5)	20	30·9 (23·4–40·1)	21
Eastern Europe	25·7 (24·0–27·5)	1	4·88 (4·57–5·22)	2	59·2 (55·5–63·3)	2	8·78 (8·17–9·47)	8	53·9 (50·3–58·1)	9	9·17 (8·55–9·87)	22	68·4 (62·9–73·9)	19	17·9 (16·0–19·2)	34
Eastern Sub-Saharan Africa	6·75 (5·48–8·49)	9	2·35 (1·72–3·03)	14	16·3 (13·2–20·3)	12	3·44 (2·65–4·37)	20	40·2 (33·4–48·6)	14	11·6 (9·20–14·3)	28	108 (92·3–125)	18	29·2 (23·6–36·0)	36
High-income Asia Pacific	15·4 (14·4–16·0)	1	7·80 (6·79–8·16)	1	29·8 (25·7–31·5)	1	11·8 (9·51–12·7)	1	37·5 (30·3–40·9)	7	12·3 (10·2–13·5)	9	43·8 (34·7–48·5)	24	17·1 (13·1–20·0)	34

High-income North America	19·8 (19·1–20·5)	1	5·34 (5·15–5·55)	3	29·3 (28·3–30·3)	2	9·15 (8·83–9·50)	4	30·7 (29·7–31·7)	10	9·66 (9·22–10·0)	19	35·0 (31·6–37·1)	31	5·22 (4·47–5·65)	66
North Africa and Middle East	4·93 (4·01–5·82)	5	2·47 (1·86–3·08)	6	7·34 (6·08–8·73)	7	2·72 (2·03–3·35)	16	6·19 (4·87–7·38)	28	1·93 (1·35–2·35)	44	7·66 (5·90–9·15)	49	2·92 (1·99–3·61)	72
Oceania	7·57 (5·96–10·1)	6	4·11 (2·92–7·04)	9	11·2 (8·67–14·1)	14	3·03 (2·22–4·31)	22	9·38 (7·07–11·8)	27	4·53 (3·34–6·07)	32	12·3 (9·42–15·2)	42	6·07 (4·71–7·98)	57
South Asia	12·5 (10·8–14·2)	2	11·6 (9·49–13·6)	1	21·4 (17·8–24·3)	6	10·4 (7·15–12·3)	6	20·3 (15·9–23·3)	18	10·5 (6·76–12·3)	26	28·3 (21·8–32·5)	30	14·8 (9·98–17·4)	40
Southeast Asia	5·53 (4·67–6·44)	4	1·87 (1·53–2·60)	9	9·53 (7·85–11·5)	10	2·50 (2–3·36)	20	11·4 (9·24–13·6)	21	3·78 (2·68–4·81)	34	20·6 (17·0–24·1)	37	7·93 (5·83–9·96)	52
Southern Latin America	19·2 (17·4–21·1)	1	4·73 (4·25–5·23)	1	22·6 (20·6–24·7)	1	4·96 (4·44–5·51)	6	24·8 (22·5–27·3)	15	5·38 (4·85–5·99)	30	38·7 (34·3–43·2)	30	5·63 (4·83–6·31)	66
Southern Sub-Saharan Africa	22·2 (18·3–27·5)	4	4·59 (2·90–6·34)	8	40·5 (33·3–49·3)	7	8·89 (6·62–11·7)	16	39·0 (33·2–45·7)	18	10·3 (8·23–12·7)	27	42·5 (36·6–49·1)	30	16·9 (13·5–21·0)	44
Tropical Latin America	10·7 (10·1–11·4)	3	3·10 (2·91–3·29)	3	15·7 (14·9–16·5)	7	3·94 (3·75–4·15)	13	16·7 (15·9–17·6)	21	4·13 (3·87–4·35)	36	19·3 (17·5–20·6)	46	3·67 (3·16–3·98)	72
Western Europe	7·97 (7·60–8·31)	1	2·32 (2·23–2·41)	1	18·0 (17·3–18·8)	1	5·13 (4·93–5·32)	2	22·2 (20·9–23·6)	9	7·49 (7·05–7·89)	14	33·1 (29·5–35·7)	33	8·42 (6·96–9·30)	62
Western Sub-Saharan Africa	5·30 (4·03–6·70)	8	1·67 (1·02–2·25)	18	13·5 (10·5–16·5)	12	2·56 (1·68–3·38)	21	31·0 (23·3–38·2)	16	8·79 (5·95–11·4)	26	85·3 (61·6–102)	18	22·7 (16·0–28·0)	37

Appendix Table S5: Age-standardised mortality rate due to suicide in 1990, 2019, and 2021 and percent changes from 2019 to 2021, for males, females, and both sexes combined. Estimates provided at the global, super-region, regional, and national level.

	Both sexes combined					Male					Female				
Location Name	1990	2019	2021	Percent Change From 1990 to 2021	Percent Change From 2019 to 2021	1990	2019	2021	Percent Change From 1990 to 2021	Percent Change From 2019 to 2021	1990	2019	2021	Percent Change From 1990 to 2021	Percent Change From 2019 to 2021
**Global**	**14·9 (12·8–15·7)**	**9·18 (8·58–9·70)**	**8·99 (8·34–9·64)**	**-39·5 (-43·7–-29·6)**	**-2·07 (-6·68–3·43)**	**19·3 (17·1–20·5)**	**13·2 (12·4–13·9)**	**12·8 (12·0–13·7)**	**-33·5 (-38·7–-24·4)**	**-2·68 (-7·64–3·24)**	**10·9 (7·93–12·1)**	**5·43 (4·71–6·09)**	**5·40 (4·76–6·04)**	**-50·3 (-56·4–-38·1)**	**-0·560 (-8·98–8·96)**
Low SDI	12·6 (10·7–14·1)	10·3 (9·08–11·6)	10·1 (8·88–11·6)	-19·8 (-30·3–4·24)	-1·65 (-5·81–2·86)	18·1 (14·9–20·8)	15·4 (13·6–17·6)	15·2 (13·2–17·8)	-16·1 (-27·6–9·35)	-1·49 (-6·33–3·97)	7·09 (5·40–8·85)	5·36 (4·45–6·40)	5·30 (4·35–6·36)	-25·2 (-39·4–3·46)	-1·17 (-7·11–5·42)
Low-middle SDI	14·0 (11·0–15·4)	10·2 (8·38–10·9)	10·0 (8·27–11·0)	-28·5 (-36·8–-9·43)	-1·83 (-8·54–5·40)	16·9 (13·3–18·9)	13·7 (11·5–14·9)	13·4 (11·3–14·9)	-20·6 (-31·4–-1·01)	-2·32 (-10·8–6·61)	11·0 (7·44–12·8)	6·79 (5·29–7·69)	6·76 (5·35–7·76)	-38·5 (-48·1–-12·4)	-0·519 (-11·5–11·4)
Middle SDI	15·6 (11·1–17·3)	7·96 (7·26–8·68)	7·92 (7·10–8·78)	-49·2 (-55·8–-34·0)	-0·519 (-9·38–9·42)	17·4 (13·1–19·7)	10·9 (9·90–12·1)	10·8 (9·76–12·0)	-37·9 (-47·0–-20·0)	-0·759 (-11·5–11·4)	13·9 (8·10–16·6)	5·21 (4·34–6·02)	5·22 (4·28–6·14)	-62·5 (-69·2–-47·4)	0·136 (-13·4–17·0)
High-middle SDI	16·4 (14·4–17·5)	8·41 (7·89–9·19)	8·07 (7·49–8·89)	-50·9 (-55·4–-41·5)	-3·94 (-11·5–4·50)	23·0 (20·5–24·6)	12·8 (11·9–13·9)	12·2 (11·2–13·4)	-46·9 (-52·5–-38·7)	-4·67 (-13·6–5·78)	10·8 (8·44–12·4)	4·24 (3·69–5·22)	4·14 (3·61–5·09)	-61·5 (-68·4–-50·1)	-2·32 (-17·0–16·3)
High SDI	13·7 (13·4–14·5)	11·3 (10·9–11·5)	10·8 (10·5–11·1)	-20·6 (-27·1–-18·5)	-3·94 (-4·98–-2·81)	20·7 (20·3–22·0)	17·2 (16·8–17·5)	16·5 (16·0–16·9)	-20·3 (-26·7–-18·1)	-4·04 (-5·19–-2·83)	7·28 (7·05–7·85)	5·46 (5·22–5·61)	5·26 (5·00–5·42)	-27·8 (-34·4–-25·3)	-3·80 (-5·15–-2·36)
**Central Europe, Eastern Europe, and Central Asia**	**20·7 (20·5–20·9)**	**15·4 (15·0–15·9)**	**14·7 (13·8–15·5)**	**-29·0 (-33·6–-25·0)**	**-4·70 (-10·9–1·16)**	**35·2 (34·8–35·6)**	**26·8 (26·0–27·8)**	**25·5 (23·7–27·1)**	**-27·6 (-32·7–-22·8)**	**-5·11 (-11·8–1·42)**	**8·37 (8·20–8·50)**	**5·32 (5·13–5·54)**	**5·13 (4·75–5·55)**	**-38·7 (-43·0–-33·9)**	**-3·51 (-11·5–5·03)**
Central Asia	11·5 (11·1–12·0)	10·3 (9·82–10·8)	9·73 (8·78–10·7)	-15·4 (-24·0–-6·89)	-5·41 (-11·9–1·26)	18·1 (17·3–18·9)	16·3 (15·5–17·2)	15·4 (14·0–16·9)	-15·0 (-23·4–-6·22)	-5·49 (-12·0–1·53)	5·62 (5·38–5·90)	4·80 (4·53–5·09)	4·55 (4·05–5·09)	-19·0 (-28·1–-9·19)	-5·27 (-12·8–2·72)
Armenia	4·12 (3·95–4·28)	4·71 (4·44–5·03)	4·54 (4·06–5·13)	10·2 (-2·65–27·1)	-3·71 (-12·4–5·27)	6·03 (5·72–6·38)	7·85 (7·36–8·40)	7·75 (6·93–8·76)	28·5 (11·9–49·2)	-1·30 (-10·5–8·26)	2·43 (2·25–2·61)	2·02 (1·88–2·19)	1·77 (1·56–2·04)	-26·9 (-37·8–-13·6)	-12·3 (-20·9–-2·83)
Azerbaijan	3·19 (2·84–3·62)	2·62 (2·05–3·43)	2·35 (1·75–3·14)	-26·4 (-43·9–-1·57)	-10·3 (-21·4–0·815)	4·97 (4·38–5·75)	4·18 (3·20–5·64)	3·74 (2·75–5·14)	-24·7 (-43·5–3·28)	-10·4 (-22·5–1·28)	1·69 (1·43–2·07)	1·16 (0·881–1·58)	1·04 (0·764–1·45)	-38·5 (-53·2–-20·7)	-10·2 (-21·0–1·76)
Georgia	5·14 (4·79–5·54)	7·88 (7·18–8·66)	7·24 (6·34–8·19)	40·8 (21·8–62·1)	-8·19 (-15·5–-1·37)	8·32 (7·73–8·99)	13·9 (12·7–15·3)	12·7 (11·2–14·4)	52·6 (31·5–75·4)	-8·90 (-16·1–-1·96)	2·56 (2·34–2·78)	2·45 (2·22–2·68)	2·34 (2·04–2·65)	-8·54 (-21·7–5·66)	-4·61 (-13·9–5·02)
Kazakhstan	21·3 (19·9–22·8)	19·6 (18·0–21·2)	18·0 (15·9–20·1)	-15·7 (-26·4–-3·82)	-8·29 (-17·4–1·12)	35·1 (32·5–37·8)	34·4 (31·6–37·3)	31·7 (28·2–35·4)	-9·67 (-21·7–3·78)	-7·78 (-17·1–2·30)	9·53 (8·87–10·2)	6·78 (6·20–7·33)	6·20 (5·43–6·96)	-35·0 (-43·9–-24·7)	-8·61 (-18·3–1·11)
Kyrgyzstan	15·7 (14·5–16·9)	10·1 (9·22–10·9)	10·0 (8·52–11·6)	-36·0 (-45·8–-25·1)	-0·451 (-11·3–11·1)	25·4 (23·5–27·6)	16·4 (14·8–17·9)	16·3 (13·9–19·0)	-35·8 (-45·9–-24·4)	-0·161 (-11·3–11·8)	7·06 (6·51–7·68)	4·40 (4·00–4·86)	4·30 (3·64–5·02)	-39·0 (-49·0–-27·1)	-2·28 (-13·2–10·8)
Mongolia	15·6 (12·0–20·0)	16·8 (13·5–20·1)	15·2 (12·5–18·2)	-2·64 (-24·2–27·8)	-9·31 (-20·3–2·02)	25·4 (18·9–33·0)	30·1 (23·7–36·8)	27·3 (21·9–33·5)	7·65 (-17·9–43·7)	-9·18 (-21·1–3·70)	6·56 (4·53–9·41)	4·62 (3·55–5·93)	4·19 (3·22–5·33)	-36·1 (-55·6–-5·79)	-9·24 (-21·0–3·98)
Tajikistan	6·16 (5·24–7·07)	3·98 (3·12–5·47)	3·87 (2·95–5·40)	-37·2 (-52·3–-14·9)	-2·76 (-13·7–9·38)	8·75 (7·24–10·4)	5·79 (4·27–8·15)	5·65 (3·97–8·21)	-35·4 (-53·5–-8·26)	-2·37 (-15·2–11·5)	3·68 (2·93–4·43)	2·18 (1·55–3·24)	2·10 (1·46–3·22)	-43·0 (-59·2–-11·1)	-3·70 (-17·1–9·78)

Turkmenistan	10·0 (9·33–10·7)	10·0 (8·25–12·2)	9·92 (7·84–12·8)	-1·00 (-23·1–29·3)	-0·963 (-11·0–11·4)	15·1 (13·9–16·3)	15·1 (12·5–18·4)	15·0 (11·9–19·2)	-0·916 (-23·2–28·1)	-0·832 (-10·7–11·3)	5·35 (4·94–5·83)	4·87 (3·95–6·06)	4·78 (3·70–6·32)	-10·7 (-32·8–19·8)	-1·88 (-12·9–11·4)
Uzbekistan	9·42 (8·99–9·84)	9·35 (8·68–9·99)	9·11 (7·91–10·5)	-3·28 (-16·7–11·7)	-2·59 (-12·6–8·52)	14·0 (13·2–14·8)	12·9 (11·9–14·0)	12·7 (11·0–14·6)	-9·46 (-22·2–6·41)	-2·09 (-13·1–10·5)	5·16 (4·89–5·44)	6·08 (5·62–6·56)	5·84 (5·01–6·82)	13·1 (-3·61–30·7)	-3·85 (-15·2–8·41)
Central Europe	16·8 (16·5–17·2)	10·8 (10·5–11·0)	10·1 (9·46–10·8)	-39·8 (-43·8–-36·0)	-5·66 (-11·5–-0·437)	27·2 (26·6–27·8)	18·5 (18·0–18·8)	17·4 (16·2–18·5)	-35·9 (-40·3–-31·9)	-5·51 (-12·0–-0·161)	7·52 (7·30–7·79)	3·55 (3·41–3·80)	3·33 (3·03–3·68)	-55·7 (-59·4–-51·5)	-6·18 (-13·5–-0·514)
Albania	4·89 (4·28–5·67)	3·77 (3·04–4·69)	3·24 (2·55–4·07)	-33·7 (-48·8–-15·1)	-14·1 (-24·9–-1·11)	6·09 (5·06–7·28)	4·88 (3·73–6·40)	4·34 (3·23–5·80)	-28·6 (-48·2–-0·550)	-10·9 (-23·9–3·49)	3·84 (3·15–4·62)	2·72 (2·00–3·55)	2·18 (1·59–2·94)	-43·2 (-58·1–-21·1)	-19·7 (-33·3–-4·02)
Bosnia and Herzegovina	14·0 (12·8–15·5)	8·90 (7·85–10·2)	7·68 (5·75–9·42)	-45·3 (-58·6–-32·3)	-13·6 (-31·9–1·35)	22·2 (20·0–24·7)	14·9 (12·9–17·2)	13·0 (9·42–16·2)	-41·5 (-56·6–-26·2)	-12·8 (-32·5–3·49)	6·25 (5·39–7·40)	3·30 (2·73–4·71)	2·77 (1·98–3·96)	-55·7 (-68·2–-38·9)	-16·1 (-33·4–-0·621)
Bulgaria	16·7 (16·0–17·6)	9·65 (9·01–10·4)	9·26 (7·96–10·6)	-44·7 (-52·8–-36·3)	-4·08 (-16·8–8·61)	24·9 (23·4–26·4)	15·6 (14·2–17·1)	14·9 (12·7–17·2)	-40·1 (-49·2–-30·8)	-4·19 (-16·9–8·00)	9·34 (8·78–9·94)	4·35 (4·06–4·67)	4·23 (3·60–4·92)	-54·7 (-62·5–-45·9)	-2·82 (-17·2–11·0)
Croatia	20·3 (19·4–21·2)	10·1 (9·44–10·9)	9·33 (8·18–10·4)	-54·0 (-60·1–-48·5)	-7·98 (-18·1–0·572)	32·6 (31·1–34·3)	16·7 (15·5–17·9)	15·3 (13·5–17·1)	-53·1 (-59·0–-47·2)	-8·23 (-18·1–0·317)	10·6 (9·98–11·4)	4·31 (3·95–4·72)	4·04 (3·44–4·59)	-62·1 (-67·8–-56·3)	-6·26 (-17·5–3·95)
Czechia	18·3 (17·6–19·0)	10·0 (9·54–10·5)	9·16 (8·09–10·2)	-50·0 (-56·1–-44·1)	-8·79 (-18·6–0·256)	29·4 (28·2–30·6)	16·8 (15·9–17·7)	15·4 (13·6–17·2)	-47·7 (-53·8–-41·3)	-8·56 (-18·1–0·493)	8·87 (8·52–9·30)	3·59 (3·38–3·81)	3·26 (2·82–3·68)	-63·3 (-68·1–-58·2)	-9·11 (-19·6–1·02)
Hungary	33·3 (31·9–34·7)	12·1 (11·5–12·7)	11·6 (10·4–12·8)	-65·0 (-68·9–-61·1)	-3·50 (-12·7–6·10)	52·9 (50·5–55·3)	19·9 (19·0–20·9)	19·1 (17·2–21·1)	-63·9 (-68·0–-59·8)	-4·13 (-13·3–5·10)	16·5 (15·9–17·2)	5·41 (5·14–5·70)	5·25 (4·61–5·91)	-68·1 (-72·0–-64·2)	-2·81 (-13·2–7·63)
Montenegro	15·2 (13·2–17·8)	13·6 (11·7–15·9)	12·1 (10·1–14·5)	-20·5 (-35·7–-0·147)	-10·8 (-18·6–-2·68)	24·4 (20·6–29·0)	21·9 (18·5–26·5)	19·7 (16·1–24·1)	-19·3 (-37·0–2·52)	-10·1 (-18·8–-0·355)	6·75 (5·29–9·06)	5·91 (4·65–7·59)	5·28 (4·12–6·87)	-21·8 (-42·2–4·40)	-10·7 (-19·4–-2·48)
North Macedonia	9·32 (8·25–10·6)	6·91 (6·01–8·07)	6·10 (4·78–7·41)	-34·5 (-48·6–-18·6)	-11·8 (-26·6–3·88)	12·6 (10·6–14·7)	10·4 (8·64–12·2)	8·89 (6·83–11·1)	-29·2 (-45·7–-8·58)	-14·2 (-28·7–-0·177)	6·21 (5·15–7·51)	3·67 (2·98–5·02)	3·42 (2·57–4·70)	-44·9 (-58·7–-26·5)	-6·68 (-22·9–12·0)
Poland	15·9 (15·6–16·2)	12·7 (12·5–13·0)	12·1 (11·2–13·1)	-23·8 (-30·3–-17·7)	-4·71 (-12·2–2·42)	27·6 (27·0–28·1)	22·8 (22·3–23·3)	21·8 (19·8–23·7)	-21·1 (-28·5–-13·9)	-4·51 (-13·0–3·59)	5·10 (4·99–5·20)	3·11 (3·03–3·18)	2·92 (2·59–3·24)	-42·7 (-49·2–-36·5)	-6·00 (-16·9–3·69)
Romania	9·27 (8·93–9·61)	8·22 (7·72–8·73)	7·91 (6·99–8·93)	-14·7 (-25·4–-4·16)	-3·70 (-13·0–5·80)	14·4 (13·8–15·0)	14·3 (13·3–15·3)	13·8 (12·2–15·6)	-3·70 (-16·0–8·23)	-3·48 (-12·5–5·73)	4·52 (4·32–4·72)	2·45 (2·31–2·59)	2·35 (2·06–2·66)	-48·0 (-54·6–-41·0)	-4·23 (-15·0–6·69)
Serbia	20·7 (18·3–22·9)	11·6 (10·7–13·0)	10·9 (9·10–12·8)	-47·5 (-56·7–-37·7)	-6·56 (-18·1–5·27)	31·7 (27·8–35·5)	18·5 (16·8–20·7)	17·2 (14·2–20·6)	-45·6 (-55·6–-35·2)	-6·89 (-18·4–4·93)	10·9 (9·06–13·3)	5·28 (4·66–7·00)	5·02 (3·95–6·62)	-53·8 (-64·1–-39·6)	-4·81 (-17·7–7·75)
Slovakia	16·4 (14·6–18·0)	9·98 (8·80–11·1)	9·31 (7·77–10·9)	-43·3 (-53·1–-31·1)	-6·68 (-17·8–3·33)	28·8 (25·5–32·6)	17·7 (15·5–20·0)	16·5 (13·6–19·3)	-42·6 (-52·7–-29·5)	-6·52 (-17·5–3·67)	5·27 (4·32–6·30)	2·89 (2·41–3·69)	2·66 (2·09–3·46)	-49·6 (-61·2–-31·1)	-8·04 (-20·7–4·16)
Slovenia	26·4 (25·3–27·7)	12·0 (11·2–12·8)	10·9 (9·56–12·2)	-58·8 (-63·9–-53·4)	-9·57 (-19·4–-0·0420)	43·2 (40·9–45·4)	20·6 (19·2–22·1)	18·7 (16·5–21·0)	-56·7 (-61·6–-51·0)	-9·17 (-19·1–0·172)	11·6 (10·8–12·4)	4·10 (3·69–4·54)	3·65 (3·05–4·22)	-68·5 (-73·8–-63·5)	-11·0 (-23·9–1·84)
Eastern Europe	24·9 (24·6–25·2)	19·9 (19·1–20·8)	19·2 (17·5–20·8)	-23·1 (-29·7–-16·6)	-3·87 (-12·9–4·77)	43·8 (43·3–44·4)	35·9 (34·1–37·7)	34·2 (30·9–37·5)	-21·9 (-29·3–-14·2)	-4·55 (-14·4–5·36)	9·29 (9·10–9·43)	6·23 (5·93–6·56)	6·11 (5·49–6·85)	-34·2 (-41·1–-26·4)	-1·91 (-13·6–10·8)
Belarus	20·0 (19·2–20·8)	15·6 (14·9–16·4)	16·1 (13·2–19·4)	-19·3 (-34·5–-3·10)	3·16 (-14·3–21·5)	34·5 (33·2–35·8)	28·5 (26·9–30·0)	29·4 (24·1–35·0)	-14·9 (-31·3–1·92)	2·89 (-14·0–20·6)	7·62 (7·25–8·04)	5·08 (4·71–5·46)	5·17 (4·12–6·38)	-32·1 (-46·1–-15·7)	1·67 (-17·9–22·8)
Estonia	24·3 (23·2–25·5)	11·0 (10·3–11·8)	11·2 (9·81–12·4)	-53·9 (-60·2–-48·3)	1·80 (-8·73–11·1)	40·8 (38·7–43·0)	19·6 (18·3–21·1)	20·1 (17·7–22·5)	-50·8 (-57·1–-44·4)	2·31 (-8·22–12·0)	10·6 (9·91–11·3)	3·60 (3·29–3·96)	3·45 (2·99–3·91)	-67·4 (-72·1–-62·4)	-4·12 (-14·7–8·55)

Latvia	25·1 (23·9–26·3)	14·2 (13·3–15·1)	14·3 (12·7–15·9)	-42·9 (-49·8–-36·1)	0·608 (-9·12–9·86)	42·9 (40·8–45·1)	26·3 (24·5–28·0)	26·6 (23·3–29·7)	-38·1 (-45·8–-30·0)	1·19 (-8·90–11·2)	10·4 (9·79–11·0)	3·99 (3·66–4·33)	3·75 (3·20–4·26)	-64·0 (-69·3–-58·7)	-6·13 (-17·8–5·54)
Lithuania	27·3 (26·3–28·4)	21·4 (20·4–22·5)	20·6 (18·3–22·7)	-24·4 (-33·2–-15·6)	-3·40 (-12·6–4·76)	47·1 (45·0–49·2)	39·7 (37·7–41·9)	38·5 (34·1–42·4)	-18·2 (-27·6–-8·40)	-3·14 (-12·5–5·13)	10·5 (9·94–11·1)	5·49 (5·08–5·95)	5·19 (4·44–5·91)	-50·6 (-58·0–-43·5)	-5·55 (-17·0–5·34)
Republic of Moldova	16·9 (16·2–17·6)	12·3 (11·2–13·5)	11·3 (10·1–12·7)	-33·2 (-40·6–-25·4)	-7·64 (-15·9–0·935)	28·1 (26·7–29·5)	22·5 (20·3–24·9)	20·5 (18·3–23·2)	-27·1 (-35·6–-17·8)	-8·70 (-17·1–0·245)	7·55 (7·23–7·93)	3·05 (2·76–3·35)	3·11 (2·77–3·50)	-58·9 (-64·0–-53·7)	1·93 (-9·04–13·2)
Russian Federation	27·5 (27·1–27·9)	20·8 (20·4–21·2)	19·9 (18·4–21·3)	-27·8 (-33·2–-22·0)	-4·46 (-11·4–2·11)	48·5 (47·8–49·3)	37·2 (36·5–37·9)	35·2 (31·9–38·0)	-27·4 (-34·1–-21·4)	-5·35 (-13·9–1·71)	10·2 (9·96–10·4)	6·63 (6·48–6·76)	6·55 (5·86–7·22)	-35·6 (-42·5–-28·6)	-1·10 (-11·7–8·73)
Ukraine	19·0 (18·3–19·7)	19·2 (15·4–23·2)	18·6 (13·5–24·4)	-2·25 (-29·9–28·4)	-3·03 (-35·1–31·3)	33·8 (32·4–35·1)	34·9 (26·9–43·6)	33·8 (23·3–45·6)	-0·0529 (-31·3–34·9)	-3·19 (-38·3–38·9)	7·12 (6·85–7·38)	5·66 (4·33–7·21)	5·35 (3·43–7·79)	-24·9 (-51·7–8·70)	-5·50 (-42·6–57·3)
**High-income**	**12·9 (12·6–13·6)**	**11·3 (10·9–11·5)**	**10·8 (10·4–11·0)**	**-15·9 (-23·5–-13·9)**	**-4·51 (-5·32–-3·62)**	**19·7 (19·4–20·9)**	**17·5 (16·9–17·8)**	**16·7 (16·1–17·1)**	**-15·3 (-22·9–-13·1)**	**-4·61 (-5·48–-3·60)**	**6·72 (6·51–7·21)**	**5·38 (5·14–5·51)**	**5·14 (4·88–5·29)**	**-23·5 (-31·2–-21·3)**	**-4·35 (-5·39–-3·22)**
Australasia	12·9 (12·5–13·2)	12·0 (11·4–12·5)	11·2 (10·6–11·7)	-13·3 (-18·0–-8·61)	-6·92 (-8·43–-5·23)	20·8 (20·2–21·4)	18·5 (17·5–19·4)	17·2 (16·1–18·1)	-17·4 (-22·4–-12·3)	-7·19 (-8·94–-5·21)	5·28 (5·08–5·49)	5·68 (5·43–5·90)	5·33 (5·09–5·55)	0·981 (-4·26–6·66)	-6·20 (-8·02–-4·45)
Australia	12·9 (12·5–13·2)	12·2 (11·6–12·7)	11·3 (10·7–11·9)	-11·9 (-16·8–-6·59)	-6·78 (-8·41–-4·88)	20·8 (20·1–21·4)	18·8 (17·8–19·8)	17·6 (16·4–18·6)	-15·4 (-20·8–-9·71)	-6·67 (-8·52–-4·55)	5·29 (5·07–5·52)	5·73 (5·46–5·97)	5·32 (5·05–5·56)	0·565 (-5·18–6·72)	-7·19 (-9·12–-5·31)
New Zealand	12·8 (12·4–13·3)	11·1 (10·6–11·6)	10·2 (9·68–10·8)	-20·3 (-25·4–-15·1)	-7·51 (-9·97–-5·06)	20·8 (20·0–21·7)	16·8 (16·0–17·8)	15·2 (14·3–16·1)	-27·1 (-32·0–-21·9)	-9·70 (-12·5–-6·64)	5·19 (4·93–5·49)	5·39 (5·12–5·66)	5·32 (5·00–5·61)	2·45 (-4·37–11·2)	-1·23 (-4·70–2·19)
High-income Asia Pacific	14·7 (14·1–18·5)	14·4 (12·3–14·8)	14·1 (12·2–14·6)	-4·13 (-33·7–1·57)	-1·70 (-3·01–-0·430)	19·5 (18·8–25·3)	20·6 (17·7–21·3)	20·1 (17·3–20·9)	3·09 (-29·9–9·54)	-2·53 (-4·01–-1·11)	10·3 (9·73–12·8)	8·22 (6·90–8·61)	8·24 (6·98–8·68)	-20·4 (-43·2–-14·6)	0·276 (-1·53–2·04)
Brunei Darussalam	4·93 (3·93–5·83)	3·76 (3·04–4·35)	3·65 (2·95–4·22)	-25·9 (-41·9–-3·62)	-2·90 (-13·1–5·53)	6·97 (5·37–8·35)	5·67 (4·59–6·75)	5·57 (4·48–6·49)	-20·1 (-38·6–4·52)	-1·74 (-13·8–9·26)	2·61 (1·91–3·30)	1·79 (1·40–2·19)	1·60 (1·19–2·03)	-38·7 (-54·2–-11·8)	-10·8 (-17·9–-3·41)
Japan	14·9 (14·5–15·2)	13·3 (13·0–13·5)	13·2 (12·9–13·5)	-11·5 (-13·3–-9·78)	-0·675 (-1·69–0·272)	19·7 (19·3–20·0)	19·2 (18·9–19·5)	18·9 (18·4–19·3)	-4·08 (-6·21–-1·94)	-1·85 (-3·00–-0·807)	10·5 (10·0–10·7)	7·39 (7·11–7·59)	7·56 (7·29–7·76)	-27·7 (-29·5–-26·0)	2·31 (0·511–3·98)
Republic of Korea	12·9 (11·0–30·4)	18·8 (11·3–20·2)	18·1 (11·1–19·7)	40·7 (-62·6–74·5)	-3·75 (-6·51–-0·691)	18·1 (15·5–45·7)	27·5 (16·1–29·7)	26·2 (15·5–28·8)	44·9 (-63·1–79·4)	-4·49 (-7·62–-1·14)	8·52 (7·07–20·0)	11·0 (6·25–12·2)	10·7 (6·17–12·1)	25·7 (-63·2–62·2)	-2·58 (-6·43–1·09)
Singapore	14·9 (14·3–15·5)	6·59 (6·22–6·94)	6·58 (6·18–6·98)	-55·9 (-59·3–-52·7)	-0·136 (-3·40–2·56)	18·2 (17·3–19·2)	8·74 (8·23–9·23)	8·81 (8·27–9·40)	-51·7 (-55·4–-47·7)	0·752 (-2·56–3·93)	11·8 (11·2–12·4)	4·47 (4·18–4·75)	4·40 (4·08–4·71)	-62·7 (-65·8–-59·8)	-1·55 (-5·01–1·77)
High-income North America	11·9 (11·8–12·1)	13·2 (12·9–13·4)	12·8 (12·4–13·2)	7·33 (3·97–10·9)	-2·74 (-4·50–-0·834)	19·9 (19·6–20·2)	20·8 (20·4–21·2)	20·3 (19·6–20·9)	1·73 (-1·68–5·12)	-2·63 (-4·57–-0·595)	4·77 (4·68–4·86)	5·87 (5·75–5·99)	5·69 (5·50–5·89)	19·3 (15·1–23·8)	-3·11 (-5·24–-0·896)
Canada	13·1 (12·8–13·5)	11·2 (10·8–11·7)	11·1 (10·5–11·6)	-15·7 (-20·3–-10·6)	-1·54 (-3·76–0·620)	21·1 (20·4–21·7)	17·3 (16·5–18·1)	17·1 (16·1–18·0)	-19·1 (-24·2–-13·7)	-1·37 (-3·85–1·19)	5·59 (5·40–5·79)	5·37 (5·15–5·62)	5·22 (4·98–5·50)	-6·64 (-12·1–-0·932)	-2·83 (-5·13–-0·349)
Greenland	95·1 (79·0–107)	57·5 (48·0–67·8)	53·5 (43·0–63·8)	-43·8 (-55·3–-28·7)	-7·04 (-14·1–0·0530)	131 (105–153)	85·5 (70·8–102)	78·8 (63·0–94·9)	-39·7 (-52·6–-20·4)	-7·80 (-14·9–-0·672)	53·7 (43·8–62·8)	27·4 (21·9–33·3)	26·3 (20·7–33·0)	-51·0 (-63·2–-35·5)	-3·87 (-11·5–4·16)
United States of America	11·8 (11·6–12·0)	13·4 (13·1–13·6)	13·0 (12·6–13·4)	10·5 (6·79–14·3)	-2·82 (-4·74–-0·813)	19·7 (19·4–20·0)	21·2 (20·7–21·6)	20·6 (19·9–21·3)	4·47 (0·835–8·08)	-2·70 (-4·83–-0·574)	4·67 (4·58–4·76)	5·94 (5·80–6·06)	5·75 (5·54–5·96)	23·1 (18·4–27·9)	-3·10 (-5·40–-0·701)
Southern Latin America	12·2 (11·8–12·6)	11·1 (10·7–11·5)	9·98 (9·51–10·5)	-18·2 (-23·0–-13·6)	-10·1 (-12·0–-7·97)	20·6 (20·0–21·4)	18·8 (18·0–19·6)	16·9 (16·0–17·7)	-18·3 (-23·1–-13·5)	-10·2 (-12·2–-7·99)	4·75 (4·59–4·91)	4·09 (3·88–4·31)	3·68 (3·46–3·91)	-22·5 (-27·6–-16·8)	-10·1 (-12·6–-7·63)

Argentina	8·74 (8·39–9·07)	11·0 (10·4–11·6)	9·63 (9·03–10·2)	10·2 (1·86–17·9)	-12·6 (-14·9–-9·83)	14·1 (13·4–14·7)	18·5 (17·4–19·6)	16·2 (15·0–17·2)	15·0 (5·54–24·2)	-12·8 (-15·2–-9·83)	4·22 (4·05–4·40)	4·17 (3·90–4·44)	3·67 (3·39–3·93)	-13·1 (-20·0–-5·50)	-12·0 (-15·0–-9·06)
Chile	21·1 (20·1–22·1)	9·82 (9·41–10·2)	9·23 (8·76–9·70)	-56·2 (-59·1–-53·1)	-6·01 (-8·28–-3·69)	37·7 (35·7–39·7)	16·6 (15·9–17·4)	15·6 (14·7–16·5)	-58·6 (-61·7–-55·2)	-6·17 (-8·64–-3·69)	5·96 (5·69–6·23)	3·46 (3·29–3·64)	3·23 (3·06–3·44)	-45·7 (-49·5–-41·4)	-6·51 (-9·47–-3·61)
Uruguay	11·3 (10·8–11·8)	18·6 (17·7–19·7)	18·2 (17·2–19·4)	60·6 (49·4–72·9)	-2·18 (-4·93–0·626)	18·5 (17·6–19·3)	32·5 (30·7–34·4)	31·9 (29·8–34·1)	72·5 (58·9–87·0)	-1·99 (-4·94–1·19)	5·22 (4·89–5·54)	6·34 (5·94–6·78)	6·13 (5·72–6·58)	17·5 (7·46–29·4)	-3·27 (-6·71–0·0533)
Western Europe	12·8 (12·5–13·0)	8·15 (7·91–8·37)	7·54 (7·29–7·77)	-40·8 (-42·5–-38·9)	-7·38 (-8·48–-6·31)	19·4 (19·1–19·8)	12·8 (12·4–13·2)	11·8 (11·4–12·2)	-39·1 (-41·1–-37·0)	-7·25 (-8·48–-6·14)	6·85 (6·64–6·98)	3·78 (3·64–3·89)	3·48 (3·34–3·59)	-49·3 (-50·8–-47·7)	-8·05 (-9·17–-6·90)
Andorra	10·4 (7·47–13·8)	6·85 (5·34–8·79)	5·71 (3·91–7·57)	-44·9 (-63·9–-16·2)	-16·6 (-32·5–-7·16)	16·0 (11·4–21·2)	10·7 (8·23–13·7)	8·99 (6·11–12·0)	-43·8 (-62·7–-13·5)	-16·2 (-32·6–-5·39)	4·11 (2·80–5·88)	2·85 (2·07–3·84)	2·30 (1·51–3·24)	-44·1 (-66·9–-7·31)	-19·3 (-34·4–-8·22)
Austria	19·4 (18·9–20·0)	11·1 (10·6–11·6)	9·99 (9·44–10·5)	-48·6 (-51·6–-45·9)	-10·3 (-12·4–-7·95)	30·2 (29·2–31·1)	17·8 (16·9–18·5)	16·1 (15·2–16·9)	-46·8 (-50·1–-43·4)	-9·60 (-11·9–-7·10)	10·3 (9·94–10·8)	5·02 (4·75–5·27)	4·35 (4·07–4·60)	-58·0 (-60·9–-55·0)	-13·3 (-16·0–-10·9)
Belgium	16·8 (16·3–17·3)	13·5 (12·9–14·2)	13·1 (12·3–13·8)	-22·2 (-27·0–-17·8)	-3·63 (-5·78–-1·37)	24·7 (23·9–25·6)	20·3 (19·3–21·3)	19·6 (18·4–20·7)	-20·7 (-26·1–-15·9)	-3·72 (-6·08–-1·10)	9·95 (9·61–10·3)	7·05 (6·66–7·42)	6·79 (6·39–7·18)	-31·8 (-36·2–-27·6)	-3·79 (-6·50–-1·27)
Cyprus	6·33 (5·58–7·16)	3·50 (3·05–4·02)	3·15 (2·67–3·68)	-50·2 (-58·3–-38·3)	-10·1 (-16·1–-3·87)	10·0 (8·84–11·7)	5·87 (5·03–6·82)	5·29 (4·42–6·20)	-47·2 (-56·9–-36·2)	-9·78 (-15·9–-3·40)	3·21 (2·46–3·89)	1·40 (1·13–1·84)	1·21 (0·962–1·59)	-62·2 (-70·8–-44·6)	-13·5 (-22·2–-1·88)
Denmark	22·4 (21·8–23·1)	8·47 (8·01–8·90)	7·80 (7·36–8·24)	-65·2 (-67·5–-63·1)	-7·85 (-10·5–-5·16)	30·7 (29·7–31·8)	12·6 (11·9–13·4)	11·6 (10·9–12·3)	-62·4 (-65·1–-59·9)	-8·56 (-11·4–-5·68)	14·6 (13·9–15·2)	4·49 (4·19–4·79)	4·24 (3·91–4·57)	-70·9 (-73·3–-68·2)	-5·75 (-9·10–-2·45)
Finland	26·8 (26·1–27·6)	13·0 (12·4–13·7)	12·0 (11·3–12·8)	-55·2 (-58·2–-52·0)	-7·50 (-10·2–-4·56)	43·7 (42·3–45·1)	19·8 (18·7–21·0)	18·5 (17·2–19·8)	-57·6 (-60·8–-54·3)	-6·57 (-9·45–-3·34)	11·1 (10·6–11·5)	6·38 (6·03–6·72)	5·70 (5·36–6·07)	-48·6 (-52·1–-44·9)	-10·7 (-14·4–-7·18)
France	20·3 (19·6–20·9)	10·8 (10·2–11·6)	10·1 (9·39–10·8)	-50·4 (-53·8–-46·4)	-7·20 (-9·43–-5·04)	31·5 (30·4–32·6)	17·8 (16·6–19·3)	16·6 (15·3–18·0)	-47·5 (-51·6–-42·8)	-6·99 (-9·47–-4·65)	10·7 (10·2–11·1)	4·50 (4·24–4·76)	4·12 (3·86–4·39)	-61·4 (-64·3–-59·1)	-8·38 (-10·5–-6·09)
Germany	14·5 (14·0–14·9)	8·79 (8·43–9·11)	8·25 (7·85–8·63)	-43·1 (-46·2–-40·2)	-6·16 (-7·98–-4·42)	22·2 (21·4–23·0)	13·8 (13·2–14·4)	12·9 (12·3–13·6)	-41·6 (-45·0–-38·1)	-6·14 (-8·24–-4·06)	7·98 (7·66–8·25)	3·99 (3·81–4·19)	3·72 (3·52–3·92)	-53·4 (-56·0–-50·7)	-6·83 (-8·92–-4·85)
Greece	3·21 (3·08–3·35)	3·72 (3·44–3·98)	3·59 (3·36–3·85)	11·8 (3·60–21·3)	-3·43 (-6·55–-0·521)	5·03 (4·78–5·28)	6·22 (5·67–6·70)	5·99 (5·54–6·47)	19·1 (9·07–30·0)	-3·58 (-7·27–-0·211)	1·51 (1·43–1·59)	1·37 (1·25–1·47)	1·34 (1·23–1·45)	-11·7 (-18·8–-2·73)	-2·15 (-5·75–-1·46)
Iceland	14·6 (13·8–15·4)	10·0 (9·18–11·0)	10·3 (9·42–11·3)	-29·0 (-35·8–-21·5)	2·90 (-2·42–8·31)	21·7 (20·3–23·1)	16·4 (15·0–17·9)	16·6 (15·2–18·2)	-23·2 (-31·2–-14·0)	1·55 (-4·20–7·56)	7·64 (7·10–8·22)	3·66 (3·31–4·04)	3·89 (3·44–4·31)	-49·2 (-55·0–-42·8)	6·14 (0·718–11·2)
Ireland	11·4 (11·0–11·8)	8·31 (7·67–9·09)	7·03 (6·45–7·73)	-38·1 (-43·8–-31·5)	-15·4 (-18·2–-12·5)	17·8 (17·0–18·5)	13·8 (12·6–15·3)	11·7 (10·6–13·0)	-34·0 (-41·0–-26·1)	-15·2 (-18·2–-12·1)	5·43 (5·15–5·73)	3·11 (2·90–3·32)	2·60 (2·40–2·79)	-52·2 (-56·4–-48·1)	-16·4 (-20·2–-13·0)
Israel	8·04 (7·72–8·37)	5·50 (5·21–5·79)	4·81 (4·49–5·11)	-40·2 (-44·3–-35·9)	-12·5 (-15·6–-9·57)	12·3 (11·7–12·8)	9·16 (8·64–9·65)	8·03 (7·49–8·55)	-34·6 (-39·4–-29·9)	-12·3 (-15·6–-9·07)	4·14 (3·93–4·35)	2·04 (1·90–2·18)	1·76 (1·63–1·89)	-57·5 (-60·9–-53·6)	-13·5 (-17·0–-9·60)
Italy	6·54 (6·35–6·69)	4·63 (4·43–4·81)	4·28 (4·10–4·46)	-34·5 (-37·3–-31·6)	-7·51 (-9·35–-5·98)	10·3 (9·98–10·5)	7·55 (7·21–7·88)	6·96 (6·66–7·26)	-32·3 (-35·2–-29·1)	-7·80 (-9·77–-6·13)	3·39 (3·27–3·49)	1·90 (1·81–1·98)	1·78 (1·68–1·87)	-47·6 (-50·2–-45·1)	-6·58 (-8·25–-4·84)
Luxembourg	16·6 (15·8–17·5)	7·37 (6·79–7·93)	6·51 (5·88–7·18)	-60·8 (-65·2–-56·1)	-11·7 (-17·4–-6·50)	25·1 (23·6–26·7)	11·4 (10·5–12·3)	9·82 (8·82–10·9)	-60·9 (-65·5–-56·0)	-14·0 (-20·5–-8·17)	8·87 (8·31–9·44)	3·57 (3·26–3·87)	3·39 (3·06–3·73)	-61·8 (-66·3–-57·3)	-4·94 (-9·92–0·166)
Malta	5·07 (4·74–5·39)	3·98 (3·62–4·31)	3·63 (3·24–3·99)	-28·5 (-36·6–-20·6)	-8·80 (-13·0–-4·52)	8·23 (7·66–8·79)	6·63 (6·01–7·21)	6·04 (5·38–6·66)	-26·7 (-35·7–-17·6)	-8·99 (-13·6–-4·29)	2·23 (2·06–2·40)	1·37 (1·23–1·53)	1·24 (1·10–1·39)	-44·3 (-51·1–-37·0)	-9·37 (-14·3–-4·93)

Monaco	12·5 (10·0–15·1)	11·9 (9·02–15·4)	11·7 (8·68–15·3)	-6·93 (-32·3–28·9)	-2·04 (-10·4–5·53)	16·3 (12·8–19·8)	17·0 (12·9–21·9)	16·7 (12·5–21·9)	2·52 (-27·6–45·9)	-1·88 (-10·6–6·40)	9·20 (6·68–11·8)	7·12 (5·24–9·65)	6·96 (4·97–9·71)	-24·3 (-47·7–9·17)	-2·28 (-12·2–6·77)
Netherlands	10·4 (10·1–10·7)	9·31 (8·93–9·69)	8·58 (8·13–8·98)	-17·5 (-21·8–-13·0)	-7·86 (-10·1–-5·81)	14·0 (13·5–14·4)	12·9 (12·3–13·5)	11·9 (11·3–12·6)	-14·7 (-19·9–-9·61)	-7·64 (-9·84–-5·55)	7·16 (6·89–7·41)	5·85 (5·57–6·13)	5·38 (5·04–5·68)	-24·9 (-29·8–-19·8)	-8·14 (-10·8–-5·58)
Norway	14·7 (14·3–15·2)	10·5 (10·2–10·9)	9·76 (9·34–10·2)	-33·6 (-36·9–-30·3)	-7·47 (-9·70–-5·02)	21·9 (21·2–22·6)	15·1 (14·5–15·7)	13·7 (13·0–14·3)	-37·5 (-40·7–-34·3)	-9·65 (-12·4–-6·59)	7·63 (7·36–7·92)	5·87 (5·62–6·13)	5·77 (5·50–6·06)	-24·3 (-28·4–-19·9)	-1·77 (-5·43–1·97)
Portugal	14·1 (13·5–14·5)	7·82 (7·45–8·24)	7·24 (6·82–7·65)	-48·5 (-51·5–-44·8)	-7·46 (-9·99–-5·08)	22·5 (21·6–23·4)	12·6 (12·0–13·3)	11·7 (11·0–12·4)	-48·0 (-51·4–-44·2)	-7·20 (-10·2–-4·45)	6·94 (6·67–7·22)	3·68 (3·45–3·91)	3·38 (3·14–3·60)	-51·3 (-54·9–-47·9)	-8·17 (-10·9–-5·47)
San Marino	10·7 (9·13–12·5)	8·05 (6·58–9·95)	5·39 (3·44–7·56)	-49·7 (-67·5–-25·3)	-33·0 (-50·5–-16·8)	17·6 (14·6–20·9)	13·4 (10·9–16·6)	8·86 (5·73–12·6)	-49·8 (-68·4–-24·1)	-34·0 (-51·2–-17·6)	4·33 (3·36–5·51)	3·19 (2·39–4·36)	2·20 (1·33–3·27)	-49·1 (-69·4–-19·4)	-31·0 (-49·3–-13·7)
Spain	7·14 (6·90–7·37)	5·70 (5·41–5·98)	5·35 (5·03–5·66)	-25·0 (-29·4–-20·3)	-6·00 (-8·14–-3·98)	11·2 (10·8–11·6)	8·91 (8·40–9·41)	8·39 (7·86–8·88)	-25·0 (-30·1–-19·8)	-5·84 (-8·33–-3·54)	3·63 (3·45–3·77)	2·72 (2·54–2·87)	2·54 (2·37–2·70)	-30·1 (-34·9–-24·3)	-6·55 (-9·02–-4·20)
Sweden	18·7 (18·2–19·3)	11·6 (11·2–12·1)	9·94 (9·00–11·0)	-46·9 (-52·0–-40·9)	-14·6 (-22·3–-6·26)	26·3 (25·5–27·1)	15·8 (15·1–16·4)	13·5 (11·9–15·3)	-48·5 (-54·7–-41·4)	-14·2 (-23·9–-3·42)	11·5 (11·1–11·9)	7·51 (7·18–7·84)	6·32 (5·49–7·20)	-45·1 (-52·2–-37·3)	-15·8 (-25·6–-4·97)
Switzerland	20·9 (20·3–21·6)	8·78 (8·30–9·20)	8·18 (7·66–8·62)	-60·9 (-63·6–-58·4)	-6·80 (-9·48–-4·17)	30·7 (29·6–31·7)	13·2 (12·4–13·9)	12·3 (11·5–13·1)	-59·7 (-62·7–-56·8)	-6·35 (-9·08–-3·67)	12·0 (11·6–12·6)	4·59 (4·29–4·89)	4·21 (3·92–4·51)	-65·0 (-67·5–-62·4)	-8·22 (-11·2–-4·96)
United Kingdom	9·54 (9·43–9·64)	7·89 (7·77–7·99)	7·23 (7·09–7·37)	-24·2 (-25·9–-22·5)	-8·28 (-9·79–-6·95)	14·6 (14·4–14·8)	12·4 (12·2–12·5)	11·4 (11·1–11·6)	-22·1 (-24·2–-20·2)	-7·92 (-9·66–-6·42)	4·73 (4·65–4·79)	3·59 (3·52–3·65)	3·26 (3·18–3·33)	-31·1 (-32·7–-29·5)	-9·17 (-11·0–-7·49)
**Latin America and Caribbean**	**6·27 (6·11–6·40)**	**6·87 (6·64–7·10)**	**6·71 (6·30–7·17)**	**7·02 (0·447–13·9)**	**-2·27 (-5·98–1·97)**	**10·0 (9·75–10·2)**	**11·3 (10·9–11·7)**	**11·0 (10·3–11·8)**	**10·3 (3·11–17·6)**	**-2·14 (-6·17–2·44)**	**2·79 (2·68–2·88)**	**2·78 (2·64–2·90)**	**2·71 (2·51–2·90)**	**-2·72 (-10·2–3·95)**	**-2·33 (-6·43–1·75)**
Andean Latin America	4·26 (3·81–4·75)	5·36 (4·48–6·08)	4·83 (3·89–5·68)	13·4 (-9·14–36·4)	-9·95 (-19·7–-0·181)	5·92 (5·23–6·62)	8·22 (6·81–9·37)	7·44 (5·96–8·79)	25·6 (-0·371–53·6)	-9·49 (-20·3–1·70)	2·67 (2·32–3·10)	2·60 (2·10–3·08)	2·32 (1·80–2·83)	-13·2 (-32·4–9·53)	-10·8 (-19·1–-1·59)
Bolivia (Plurinational State of)	6·77 (5·48–8·20)	5·82 (3·99–7·89)	5·74 (3·93–7·81)	-15·3 (-43·9–19·4)	-1·37 (-9·80–7·56)	9·01 (6·95–11·2)	8·22 (5·62–11·1)	8·16 (5·38–10·9)	-9·43 (-38·7–27·9)	-0·677 (-9·75–8·30)	4·74 (3·26–6·50)	3·53 (2·31–5·11)	3·44 (2·26–5·04)	-27·4 (-55·3–13·9)	-2·41 (-12·7–8·80)
Ecuador	5·11 (4·86–5·32)	9·54 (8·46–10·7)	8·40 (6·70–10·3)	64·2 (30·5–102)	-12·0 (-25·8–3·78)	7·08 (6·71–7·42)	15·3 (13·5–17·3)	13·6 (10·8–16·9)	91·6 (50·8–139)	-11·3 (-26·8–6·63)	3·22 (3·04–3·42)	4·01 (3·51–4·52)	3·46 (2·77–4·23)	7·38 (-15·0–33·3)	-13·8 (-29·2–2·63)
Peru	3·18 (2·60–3·75)	3·20 (2·33–3·84)	2·82 (2·01–3·58)	-11·0 (-37·9–24·1)	-11·9 (-29·1–7·11)	4·56 (3·61–5·49)	4·86 (3·44–5·84)	4·30 (2·99–5·47)	-5·62 (-36·5–32·2)	-11·4 (-28·6–8·90)	1·84 (1·46–2·33)	1·60 (1·00–2·05)	1·40 (0·861–1·89)	-24·0 (-48·4–9·81)	-12·7 (-29·2–6·57)
Caribbean	13·2 (12·4–13·8)	8·95 (8·07–9·85)	8·48 (7·46–9·59)	-35·8 (-43·0–-27·6)	-5·19 (-12·3–2·80)	18·6 (17·5–19·5)	14·4 (13·1–15·8)	13·8 (12·0–15·5)	-26·1 (-34·4–-16·9)	-4·77 (-12·6–3·02)	8·21 (7·56–8·68)	3·85 (3·20–4·44)	3·62 (2·99–4·27)	-55·9 (-62·2–-48·6)	-5·83 (-12·8–1·92)
Antigua and Barbuda	2·00 (1·83–2·17)	1·42 (1·26–1·59)	1·19 (1·10–1·29)	-40·4 (-46·7–-33·6)	-16·0 (-22·5–-8·17)	3·72 (3·37–4·07)	2·71 (2·40–3·06)	2·23 (2·06–2·43)	-40·0 (-46·7–-32·3)	-17·5 (-24·2–-9·40)	0·563 (0·505–0·620)	0·288 (0·255–0·331)	0·255 (0·233–0·277)	-54·7 (-60·5–-47·9)	-11·4 (-18·6–-3·38)
Bahamas	3·01 (2·74–3·29)	2·42 (1·97–2·92)	2·37 (1·89–2·95)	-21·2 (-37·9–1·46)	-1·97 (-9·80–5·95)	5·13 (4·61–5·65)	4·51 (3·70–5·45)	4·42 (3·53–5·48)	-13·9 (-32·4–11·1)	-2·08 (-10·3–6·30)	1·17 (1·06–1·27)	0·554 (0·440–0·684)	0·558 (0·434–0·714)	-52·1 (-63·6–-37·6)	0·720 (-7·68–10·3)
Barbados	4·82 (4·51–5·18)	3·95 (3·22–4·70)	3·54 (2·71–4·47)	-26·5 (-43·7–-6·55)	-10·4 (-20·7–-1·52)	8·34 (7·75–9·05)	7·15 (5·83–8·51)	6·37 (4·87–8·05)	-23·6 (-42·1–-2·29)	-10·9 (-21·6–-1·40)	1·90 (1·73–2·06)	1·13 (0·907–1·37)	1·04 (0·800–1·30)	-45·3 (-59·2–-30·3)	-7·90 (-17·2–-0·178)
Belize	5·59 (5·19–6·06)	6·08 (5·45–6·77)	5·46 (4·76–6·22)	-2·38 (-16·5–14·1)	-10·2 (-17·5–-2·54)	8·57 (7·85–9·44)	10·6 (9·44–12·0)	9·59 (8·29–11·0)	11·8 (-5·44–31·7)	-9·67 (-17·8–-0·699)	2·57 (2·34–2·80)	1·60 (1·42–1·78)	1·40 (1·23–1·58)	-45·6 (-53·7–-36·4)	-12·5 (-20·1–-4·90)

Bermuda	6·09 (5·70–6·52)	2·61 (2·22–3·08)	2·60 (2·20–3·21)	-57·3 (-64·5–-48·4)	-0·354 (-6·69–6·62)	9·96 (9·25–10·8)	4·70 (4·02–5·55)	4·67 (3·94–5·78)	-53·1 (-61·2–-43·2)	-0·546 (-7·21–6·93)	2·89 (2·60–3·21)	0·776 (0·652–0·955)	0·769 (0·633–0·951)	-73·4 (-78·8–-66·4)	-0·819 (-8·57–6·48)
Cuba	19·9 (19·3–20·3)	11·3 (10·8–11·9)	11·0 (9·47–12·4)	-44·7 (-52·1–-37·3)	-2·98 (-15·3–9·70)	24·6 (23·9–25·3)	18·3 (17·3–19·2)	18·0 (15·4–20·5)	-27·0 (-37·5–-16·7)	-1·79 (-14·9–11·7)	15·3 (14·8–15·8)	4·80 (4·54–5·09)	4·49 (3·81–5·13)	-70·7 (-75·2–-66·0)	-6·53 (-19·5–6·89)
Dominica	3·73 (3·27–4·17)	3·66 (2·94–4·40)	3·66 (2·87–4·48)	-1·84 (-22·6–22·0)	-0·128 (-6·87–6·01)	7·10 (6·13–8·06)	6·78 (5·43–8·28)	6·74 (5·27–8·26)	-5·05 (-25·1–18·0)	-0·665 (-7·59–6·30)	1·15 (0·869–1·35)	0·775 (0·585–1·06)	0·771 (0·579–1·05)	-32·9 (-50·6–-7·67)	-0·549 (-7·77–8·09)
Dominican Republic	5·57 (4·80–6·62)	6·24 (4·95–7·73)	5·34 (4·29–6·77)	-4·07 (-25·7–22·1)	-14·4 (-29·1–3·14)	8·79 (7·45–11·1)	10·3 (7·92–13·3)	8·80 (6·93–11·5)	0·194 (-22·9–31·3)	-14·1 (-29·0–4·10)	2·51 (2·03–3·05)	2·29 (1·68–3·11)	1·97 (1·45–2·67)	-21·5 (-43·2–6·15)	-13·9 (-28·9–5·21)
Grenada	6·23 (5·59–6·90)	4·20 (3·78–4·63)	4·13 (3·53–4·76)	-33·7 (-44·9–-20·7)	-1·65 (-11·9–5·01)	10·3 (9·16–11·5)	7·40 (6·68–8·13)	7·11 (6·13–8·12)	-31·1 (-43·4–-17·4)	-3·98 (-13·7–2·79)	2·72 (2·42–3·01)	1·66 (1·46–1·90)	1·61 (1·36–1·88)	-40·7 (-51·2–-28·0)	-3·12 (-11·3–4·51)
Guyana	28·7 (25·2–31·9)	32·3 (25·3–40·3)	31·5 (24·2–40·3)	9·76 (-19·6–41·9)	-2·53 (-14·3–10·0)	43·1 (37·4–48·5)	55·8 (44·3–69·6)	54·7 (42·0–69·8)	27·0 (-8·38–64·7)	-1·94 (-14·4–11·6)	15·1 (13·0–17·3)	10·5 (7·84–13·3)	10·1 (7·45–13·1)	-33·1 (-52·7–-10·5)	-3·67 (-16·8–9·74)
Haiti	11·5 (6·19–14·9)	8·81 (5·67–11·7)	8·64 (5·73–11·5)	-25·2 (-46·6–8·03)	-1·91 (-9·71–5·53)	16·8 (9·76–22·0)	13·3 (8·79–18·1)	13·2 (8·73–18·0)	-21·4 (-44·2–13·4)	-1·02 (-9·65–7·17)	6·78 (2·78–9·06)	4·66 (2·14–6·57)	4·51 (2·16–6·34)	-33·4 (-55·1–0·807)	-3·10 (-11·3–6·41)
Jamaica	1·16 (0·986–1·38)	1·30 (1·02–1·66)	1·30 (0·970–1·68)	11·9 (-25·6–60·5)	-0·202 (-19·7–21·5)	1·98 (1·64–2·44)	2·26 (1·72–2·94)	2·26 (1·68–2·92)	13·9 (-26·8–68·2)	-0·146 (-21·3–25·2)	0·421 (0·381–0·474)	0·398 (0·300–0·518)	0·393 (0·293–0·520)	-6·69 (-31·4–26·0)	-1·24 (-14·6–14·1)
Puerto Rico	10·5 (10·0–10·9)	5·39 (5·08–5·73)	5·21 (4·31–6·13)	-50·2 (-58·9–-40·8)	-3·31 (-18·7–13·4)	19·5 (18·7–20·5)	9·72 (9·14–10·4)	9·42 (7·78–11·1)	-51·8 (-60·1–-42·8)	-3·03 (-18·7–13·9)	2·48 (2·32–2·65)	1·56 (1·44–1·69)	1·48 (1·21–1·76)	-40·2 (-51·1–-27·9)	-4·95 (-20·1–12·8)
Saint Kitts and Nevis	4·98 (4·62–5·34)	2·42 (2·05–2·90)	2·42 (2·02–2·96)	-51·5 (-60·4–-39·9)	-0·242 (-6·36–7·52)	7·62 (6·95–8·26)	4·44 (3·78–5·27)	4·44 (3·72–5·40)	-41·8 (-52·8–-28·2)	-0·150 (-6·66–7·56)	2·87 (2·56–3·19)	0·629 (0·519–0·752)	0·625 (0·506–0·762)	-78·2 (-82·6–-73·2)	-0·596 (-6·97–6·10)
Saint Lucia	7·92 (7·47–8·49)	5·66 (4·82–6·61)	5·58 (4·63–6·70)	-29·6 (-42·1–-14·8)	-1·53 (-11·8–5·70)	14·2 (13·3–15·3)	10·7 (9·12–12·4)	10·4 (8·68–12·5)	-26·6 (-39·9–-11·0)	-2·41 (-13·0–4·97)	2·81 (2·57–3·05)	0·908 (0·753–1·07)	0·967 (0·778–1·17)	-65·5 (-72·8–-57·4)	6·47 (-4·25–15·7)
Saint Vincent and the Grenadines	7·04 (6·49–7·63)	5·49 (4·93–6·09)	5·45 (4·76–6·25)	-22·6 (-34·1–-9·69)	-0·764 (-7·17–5·78)	12·9 (11·8–14·1)	10·1 (9·03–11·1)	9·96 (8·68–11·4)	-22·7 (-34·2–-10·1)	-0·994 (-7·78–5·61)	1·92 (1·73–2·12)	0·882 (0·776–1·00)	0·882 (0·755–1·02)	-54·0 (-61·8–-44·2)	-0·0120 (-6·81–7·73)
Suriname	25·3 (20·9–27·8)	24·2 (21·6–27·3)	22·2 (17·8–26·9)	-12·4 (-31·9–9·86)	-8·35 (-22·5–6·10)	35·8 (30·5–40·0)	39·6 (34·6–44·7)	36·1 (28·2–44·3)	0·997 (-21·9–27·9)	-8·79 (-24·0–6·93)	15·3 (10·9–17·5)	10·2 (8·45–13·4)	9·52 (7·45–12·8)	-37·6 (-53·7–-7·29)	-6·40 (-18·2–6·27)
Trinidad and Tobago	16·5 (15·7–17·3)	12·1 (10·3–14·2)	11·9 (9·12–15·1)	-28·3 (-45·5–-8·20)	-2·32 (-20·0–19·0)	26·3 (24·8–27·6)	20·7 (17·7–24·0)	20·1 (15·5–25·5)	-23·5 (-41·6–-1·62)	-2·67 (-20·2–18·1)	7·39 (6·90–7·98)	3·79 (3·20–4·43)	3·81 (2·88–4·95)	-48·4 (-61·2–-33·1)	0·653 (-18·5–23·7)
United States Virgin Islands	7·66 (6·32–8·98)	8·03 (6·24–10·5)	6·51 (4·95–8·49)	-15·0 (-36·3–15·4)	-19·0 (-25·4–-12·4)	12·7 (10·5–15·2)	15·0 (11·7–19·6)	12·3 (9·35–16·0)	-3·38 (-27·3–33·1)	-18·4 (-25·0–-11·6)	3·40 (2·53–4·16)	1·68 (1·14–2·76)	1·26 (0·837–2·10)	-63·0 (-76·0–-37·1)	-25·0 (-32·4–-15·7)
Central Latin America	4·66 (4·55–4·76)	6·59 (6·36–6·84)	6·47 (5·85–7·14)	38·9 (25·5–53·0)	-1·89 (-9·04–6·60)	8·07 (7·86–8·28)	11·1 (10·7–11·6)	11·0 (9·85–12·2)	36·2 (21·9–50·6)	-1·37 (-9·16–7·64)	1·45 (1·41–1·51)	2·40 (2·29–2·51)	2·32 (2·04–2·60)	60·0 (40·1–79·8)	-3·29 (-12·8–6·03)
Colombia	4·31 (4·11–4·49)	6·55 (6·29–6·82)	6·19 (5·18–7·25)	43·9 (20·6–69·4)	-5·45 (-19·4–9·54)	7·28 (6·95–7·60)	10·8 (10·3–11·3)	10·3 (8·60–12·1)	41·1 (17·4–67·6)	-5·00 (-19·3–10·3)	1·50 (1·40–1·61)	2·58 (2·42–2·74)	2·43 (2·05–2·81)	61·8 (34·8–92·3)	-6·02 (-20·0–9·34)
Costa Rica	6·06 (5·70–6·44)	8·56 (8·12–9·02)	8·90 (7·97–9·80)	47·0 (30·4–64·6)	3·96 (-5·96–12·6)	10·8 (10·2–11·5)	15·2 (14·4–16·0)	15·9 (14·2–17·6)	46·8 (29·8–64·8)	4·31 (-5·60–13·4)	1·43 (1·33–1·54)	2·53 (2·34–2·72)	2·61 (2·30–2·89)	82·6 (57·0–106)	3·22 (-7·19–12·6)
El Salvador	10·2 (9·46–11·4)	8·21 (7·32–9·12)	8·25 (6·55–10·1)	-19·4 (-36·2–0·630)	0·396 (-16·3–19·5)	16·4 (15·0–18·4)	13·9 (12·0–15·8)	14·0 (11·0–17·1)	-15·1 (-34·1–6·34)	0·740 (-15·6–20·0)	4·72 (4·10–5·55)	3·83 (3·16–4·42)	3·78 (2·78–4·80)	-19·9 (-40·2–2·23)	-1·21 (-19·2–18·2)

Guatemala	6·68 (6·39–6·97)	6·37 (6·08–6·67)	5·50 (4·70–6·32)	-17·6 (-30·5–-5·07)	-13·6 (-25·0–-2·23)	11·7 (11·2–12·3)	10·4 (9·90–11·0)	8·99 (7·67–10·3)	-23·5 (-35·7–-11·5)	-13·8 (-25·5–-2·15)	1·91 (1·79–2·03)	2·79 (2·59–2·97)	2·44 (2·07–2·83)	27·3 (7·10–48·7)	-12·6 (-23·7–-1·03)
Honduras	5·92 (4·97–7·07)	4·75 (3·57–6·37)	4·68 (3·49–6·30)	-20·9 (-43·3–5·96)	-1·48 (-12·4–11·0)	10·2 (8·25–12·5)	8·37 (6·21–11·2)	8·29 (6·18–11·2)	-18·4 (-42·4–9·98)	-1·04 (-13·1–13·1)	2·13 (1·67–2·61)	1·54 (0·965–2·30)	1·53 (0·980–2·28)	-27·9 (-52·3–11·5)	-0·495 (-17·2–18·3)
Mexico	3·80 (3·71–3·90)	6·44 (6·27–6·60)	6·49 (5·80–7·23)	70·6 (52·8–90·7)	0·729 (-9·32–11·5)	6·75 (6·57–6·93)	10·9 (10·6–11·2)	11·0 (9·66–12·6)	63·4 (43·3–87·0)	1·34 (-11·5–14·1)	1·02 (0·984–1·05)	2·30 (2·23–2·37)	2·26 (1·94–2·61)	123 (90·5–158)	-1·72 (-15·6–12·9)
Nicaragua	5·37 (4·89–7·15)	6·59 (6·06–7·19)	5·80 (4·83–6·99)	7·90 (-22·2–31·9)	-12·1 (-24·2–3·50)	8·52 (7·63–11·1)	10·6 (9·70–11·6)	9·44 (7·65–11·5)	10·7 (-16·6–37·3)	-10·9 (-24·6–5·93)	2·59 (2·19–3·99)	2·99 (2·38–3·46)	2·53 (1·89–3·27)	-2·52 (-42·2–34·4)	-15·4 (-28·7–0·592)
Panama	4·20 (3·93–4·49)	5·26 (4·94–5·53)	5·14 (4·16–6·13)	22·4 (-2·87–48·7)	-2·25 (-20·2–14·9)	7·34 (6·82–7·91)	8·89 (8·32–9·39)	8·67 (7·01–10·4)	18·2 (-7·30–45·1)	-2·41 (-21·2–14·7)	1·02 (0·942–1·12)	1·68 (1·55–1·82)	1·66 (1·35–1·99)	62·0 (31·2–94·9)	-1·47 (-17·6–15·4)
Venezuela (Bolivarian Republic of)	6·34 (6·10–6·57)	7·90 (6·46–9·62)	7·97 (6·05–10·2)	25·7 (-5·36–61·2)	0·852 (-17·6–22·3)	11·0 (10·5–11·4)	14·0 (11·4–17·3)	14·3 (11·0–18·3)	30·8 (-1·75–68·9)	2·18 (-16·8–23·2)	1·96 (1·85–2·08)	2·27 (1·82–2·80)	2·33 (1·80–2·98)	18·7 (-9·87–54·5)	2·67 (-15·9–24·9)
Tropical Latin America	6·41 (6·24–6·59)	7·02 (6·84–7·18)	7·02 (6·79–7·25)	9·48 (5·46–13·8)	0·0279 (-2·51–2·61)	10·4 (10·1–10·7)	11·4 (11·1–11·8)	11·4 (11·0–11·8)	9·27 (4·74–14·5)	-0·510 (-3·57–2·63)	2·72 (2·64–2·79)	2·94 (2·85–3·02)	2·99 (2·85–3·11)	10·1 (5·01–14·8)	1·75 (-2·28–5·16)
Brazil	6·47 (6·29–6·65)	7·03 (6·85–7·20)	7·05 (6·82–7·28)	9·10 (5·00–13·3)	0·360 (-2·19–2·95)	10·5 (10·2–10·9)	11·5 (11·2–11·8)	11·5 (11·0–11·9)	8·80 (4·32–13·9)	-0·171 (-3·12–3·14)	2·72 (2·65–2·80)	2·93 (2·84–3·01)	2·99 (2·86–3·11)	10·1 (4·93–14·7)	2·04 (-2·01–5·41)
Paraguay	4·16 (3·58–4·94)	6·59 (5·10–7·88)	5·87 (4·45–7·46)	41·2 (6·28–85·1)	-10·9 (-30·3–14·4)	5·78 (4·77–7·11)	10·2 (7·77–12·2)	8·96 (6·77–11·5)	54·9 (12·9–109)	-11·7 (-32·9–14·6)	2·62 (2·11–3·37)	3·13 (2·29–3·85)	2·88 (2·04–3·81)	10·1 (-19·6–48·8)	-7·77 (-28·8–17·3)
**North Africa and Middle East**	**4·67 (3·74–5·24)**	**3·57 (2·98–4·03)**	**3·43 (2·81–3·94)**	**-26·6 (-35·6–-3·61)**	**-3·88 (-6·86–-0·824)**	**6·02 (4·92–6·83)**	**5·00 (4·20–5·76)**	**4·83 (3·98–5·56)**	**-19·8 (-31·3–4·17)**	**-3·42 (-6·88–-0·145)**	**3·26 (2·21–3·75)**	**2·02 (1·56–2·38)**	**1·92 (1·47–2·30)**	**-41·1 (-50·3–-13·1)**	**-4·96 (-8·49–-0·988)**
North Africa and Middle East	4·67 (3·74–5·24)	3·57 (2·98–4·03)	3·43 (2·81–3·94)	-26·6 (-35·6–-3·61)	-3·88 (-6·86–-0·824)	6·02 (4·92–6·83)	5·00 (4·20–5·76)	4·83 (3·98–5·56)	-19·8 (-31·3–4·17)	-3·42 (-6·88–-0·145)	3·26 (2·21–3·75)	2·02 (1·56–2·38)	1·92 (1·47–2·30)	-41·1 (-50·3–-13·1)	-4·96 (-8·49–-0·988)
Afghanistan	6·74 (4·94–9·15)	5·40 (4·07–7·52)	5·32 (3·98–7·40)	-21·1 (-43·0–17·2)	-1·52 (-8·38–5·59)	8·36 (5·60–11·6)	6·51 (4·75–9·02)	6·39 (4·69–8·86)	-23·6 (-48·3–19·5)	-1·84 (-9·24–5·66)	5·32 (3·92–7·52)	4·30 (3·10–6·37)	4·29 (3·07–6·44)	-19·4 (-44·6–21·6)	-0·424 (-7·79–8·30)
Algeria	4·54 (3·01–5·57)	2·88 (2·01–3·56)	2·85 (1·96–3·63)	-37·2 (-52·8–-15·7)	-0·900 (-8·05–7·18)	5·38 (4·16–6·57)	3·72 (2·72–4·71)	3·71 (2·59–4·78)	-31·1 (-48·5–-6·74)	-0·238 (-8·66–7·59)	3·70 (1·67–5·03)	2·00 (1·14–2·58)	1·96 (1·10–2·61)	-46·9 (-60·8–-17·9)	-2·07 (-10·3–7·37)
Bahrain	7·29 (6·52–8·24)	3·95 (3·35–4·74)	3·93 (3·28–4·80)	-46·2 (-56·7–-34·6)	-0·680 (-7·59–7·12)	9·85 (8·48–11·4)	5·55 (4·67–6·73)	5·53 (4·57–6·73)	-43·9 (-55·9–-30·2)	-0·538 (-7·84–7·46)	3·93 (3·05–4·64)	1·37 (1·12–2·11)	1·35 (1·09–2·03)	-65·7 (-74·5–-46·4)	-1·52 (-8·69–6·11)
Egypt	1·51 (1·12–1·70)	1·17 (1·05–1·28)	1·14 (0·906–1·39)	-24·1 (-39·5–0·375)	-2·03 (-17·6–15·2)	2·09 (1·61–2·44)	1·67 (1·46–1·85)	1·65 (1·30–2·01)	-21·2 (-38·1–3·13)	-1·63 (-16·7–15·7)	0·898 (0·561–1·11)	0·623 (0·524–0·744)	0·601 (0·452–0·777)	-33·1 (-50·5–8·76)	-3·65 (-19·9–14·6)
Iran (Islamic Republic of)	6·26 (5·26–6·90)	4·48 (4·02–4·91)	4·12 (3·72–4·58)	-34·2 (-41·2–-19·3)	-8·02 (-12·6–-3·08)	7·42 (6·35–8·39)	6·53 (5·57–7·13)	6·04 (5·14–6·67)	-18·6 (-29·2–-2·83)	-7·50 (-13·4–-1·85)	5·04 (3·47–5·71)	2·37 (2·10–3·06)	2·14 (1·85–2·74)	-57·6 (-64·8–-39·6)	-9·71 (-15·9–-3·12)
Iraq	7·37 (5·86–8·86)	5·13 (3·81–6·71)	4·89 (3·68–6·46)	-33·6 (-51·1–-6·49)	-4·72 (-11·7–3·05)	10·8 (8·37–13·2)	7·69 (5·63–10·2)	7·46 (5·47–9·88)	-31·0 (-49·5–-3·32)	-3·02 (-10·7–5·48)	3·80 (2·64–4·73)	2·43 (1·71–3·45)	2·22 (1·61–3·16)	-41·7 (-59·2–-10·5)	-8·63 (-17·6–1·54)
Jordan	2·96 (2·28–3·48)	1·10 (0·913–1·31)	1·10 (0·880–1·37)	-62·8 (-71·8–-47·7)	-0·0788 (-8·79–8·76)	3·64 (2·88–4·32)	1·62 (1·32–1·94)	1·61 (1·28–1·97)	-55·9 (-66·6–-40·2)	-0·519 (-10·3–9·90)	2·20 (1·31–2·86)	0·484 (0·371–0·675)	0·489 (0·362–0·683)	-77·8 (-84·8–-60·3)	1·02 (-9·70–12·8)
Kuwait	1·59 (1·47–1·74)	1·78 (1·67–1·89)	1·78 (1·49–2·13)	11·8 (-9·44–35·4)	0·0511 (-16·0–19·1)	1·87 (1·67–2·07)	2·69 (2·51–2·87)	2·71 (2·22–3·29)	45·3 (14·4–81·5)	0·806 (-16·9–22·7)	1·19 (1·09–1·32)	0·684 (0·630–0·749)	0·698 (0·604–0·806)	-41·4 (-50·9–-31·3)	2·05 (-8·11–14·1)

Lebanon	1·68 (0·859–2·19)	0·860 (0·724–1·01)	0·856 (0·700–1·04)	-49·0 (-63·2–-0·763)	-0·487 (-9·38–9·29)	1·92 (0·999–2·57)	1·10 (0·913–1·32)	1·09 (0·892–1·34)	-43·2 (-59·9–7·69)	-0·660 (-9·34–8·92)	1·46 (0·660–2·06)	0·636 (0·487–0·825)	0·637 (0·474–0·845)	-56·4 (-71·7–1·59)	0·165 (-9·53–11·5)
Libya	4·42 (3·03–5·59)	5·07 (3·31–6·92)	4·93 (3·20–6·78)	11·5 (-21·9–95·3)	-2·70 (-10·2–5·30)	5·39 (3·75–6·88)	6·92 (4·66–9·20)	6·79 (4·61–9·07)	25·9 (-11·2–101)	-1·81 (-9·83–6·18)	3·24 (1·63–4·34)	3·09 (1·62–4·75)	2·96 (1·58–4·64)	-8·70 (-42·2–110)	-4·38 (-13·3–5·72)
Morocco	5·17 (3·41–6·54)	3·63 (2·34–4·87)	3·59 (2·30–5·04)	-30·6 (-49·9–3·34)	-1·07 (-10·5–9·40)	5·44 (4·06–7·00)	4·23 (2·89–6·05)	4·23 (2·88–6·29)	-22·1 (-43·4–12·9)	-0·0132 (-8·83–10·9)	4·91 (2·16–6·59)	3·03 (1·53–4·43)	2·96 (1·52–4·54)	-39·8 (-59·7–3·10)	-2·46 (-12·8–9·46)
Oman	1·91 (1·40–2·58)	1·32 (1·04–1·66)	1·02 (0·798–1·28)	-46·3 (-62·5–-22·8)	-22·5 (-28·9–-15·7)	2·53 (1·83–3·49)	1·92 (1·50–2·43)	1·46 (1·12–1·83)	-42·5 (-60·0–-16·5)	-23·9 (-30·1–-16·8)	0·923 (0·574–1·29)	0·423 (0·323–0·537)	0·366 (0·271–0·489)	-60·4 (-74·3–-29·6)	-13·7 (-21·6–-4·03)
Palestine	1·08 (0·776–1·45)	0·913 (0·745–1·04)	0·893 (0·708–1·06)	-17·2 (-41·8–18·1)	-2·22 (-9·72–6·40)	1·67 (1·19–2·24)	1·48 (1·11–1·71)	1·45 (1·08–1·72)	-13·3 (-39·6–25·7)	-1·88 (-10·7–6·98)	0·553 (0·388–0·794)	0·369 (0·304–0·468)	0·353 (0·282–0·455)	-36·2 (-54·7–-6·39)	-4·32 (-12·7–4·65)
Qatar	7·74 (6·30–9·49)	4·13 (3·18–5·38)	3·41 (2·60–4·48)	-56·0 (-67·8–-38·8)	-17·5 (-25·4–-9·10)	9·87 (7·93–12·3)	5·29 (4·05–6·91)	4·57 (3·47–6·05)	-53·7 (-66·3–-35·5)	-13·6 (-22·4–-4·44)	3·53 (2·48–4·42)	1·12 (0·872–1·67)	0·912 (0·679–1·35)	-74·2 (-81·8–-59·2)	-18·9 (-26·0–-11·5)
Saudi Arabia	9·18 (6·62–11·8)	6·77 (5·24–8·68)	6·64 (5·04–8·57)	-27·6 (-51·0–11·3)	-1·80 (-9·82–5·56)	11·4 (8·33–15·1)	9·23 (7·11–12·1)	9·08 (6·73–11·8)	-20·4 (-46·8–22·2)	-1·70 (-10·6–6·97)	5·86 (3·68–7·67)	3·08 (2·26–4·16)	3·00 (2·17–4·18)	-48·7 (-66·7–-9·54)	-2·55 (-11·4–8·41)
Sudan	6·61 (3·83–8·79)	4·85 (2·84–7·13)	4·68 (2·78–6·77)	-29·2 (-54·0–10·9)	-3·43 (-12·9–5·21)	7·55 (4·83–10·2)	6·36 (3·84–9·02)	6·20 (3·81–8·89)	-17·9 (-43·9–25·8)	-2·56 (-11·9–6·21)	5·65 (2·08–8·41)	3·31 (1·45–5·42)	3·14 (1·37–5·29)	-44·4 (-67·3–4·04)	-5·19 (-17·3–7·17)
Syrian Arab Republic	1·73 (1·29–2·15)	0·884 (0·660–1·18)	0·870 (0·651–1·16)	-49·6 (-64·7–-22·9)	-1·68 (-14·6–11·8)	2·52 (1·88–3·21)	1·46 (1·08–1·96)	1·44 (1·05–1·96)	-42·8 (-60·5–-14·6)	-1·22 (-14·0–12·2)	0·895 (0·561–1·15)	0·385 (0·270–0·563)	0·377 (0·271–0·566)	-57·8 (-72·4–-25·5)	-1·98 (-15·6–12·7)
Tunisia	3·63 (2·47–5·15)	2·95 (1·94–4·25)	2·88 (1·89–4·16)	-20·6 (-43·3–8·02)	-2·23 (-10·4–7·80)	4·41 (3·32–6·89)	4·28 (2·77–6·37)	4·22 (2·74–6·32)	-4·28 (-30·7–32·1)	-1·53 (-10·9–9·42)	2·86 (1·38–4·22)	1·68 (0·917–2·53)	1·62 (0·924–2·40)	-43·3 (-63·7–-15·3)	-3·71 (-14·4–7·93)
Türkiye	4·27 (3·00–5·78)	3·68 (2·81–4·29)	3·58 (2·69–4·33)	-16·2 (-51·9–39·4)	-2·67 (-13·2–8·45)	6·73 (4·65–8·71)	5·75 (4·41–6·87)	5·63 (4·22–6·83)	-16·4 (-49·8–36·6)	-2·04 (-13·5–10·3)	1·84 (1·14–3·48)	1·63 (1·13–1·98)	1·56 (1·05–1·99)	-15·7 (-61·2–54·1)	-4·85 (-15·9–10·1)
United Arab Emirates	5·27 (3·91–7·17)	3·52 (2·78–4·61)	2·76 (2·21–3·61)	-47·6 (-59·3–-29·2)	-21·6 (-33·0–-8·98)	5·93 (4·28–8·67)	3·84 (2·98–5·35)	3·22 (2·53–4·42)	-45·8 (-59·5–-23·6)	-16·3 (-28·0–-3·44)	3·82 (2·76–5·01)	3·38 (2·54–4·83)	2·48 (1·87–3·58)	-35·0 (-53·8–13·3)	-26·5 (-39·7–-13·5)
Yemen	5·62 (2·83–7·99)	4·47 (2·28–6·59)	4·45 (2·19–6·53)	-20·8 (-44·9–18·4)	-0·392 (-7·87–7·83)	7·30 (4·31–10·2)	6·05 (3·42–8·80)	6·06 (3·30–8·90)	-17·0 (-42·2–25·2)	0·200 (-7·66–8·65)	3·98 (1·20–6·26)	2·92 (1·08–4·50)	2·88 (1·07–4·44)	-27·6 (-51·4–21·5)	-1·19 (-9·77–8·69)
**South Asia**	**16·8 (12·9–18·7)**	**11·6 (9·77–12·6)**	**11·4 (9·71–12·7)**	**-31·7 (-41·0–-9·17)**	**-1·23 (-10·1–8·52)**	**19·0 (14·6–21·6)**	**14·5 (12·5–16·2)**	**14·2 (12·3–16·2)**	**-25·0 (-36·6–-1·68)**	**-2·15 (-13·7–10·2)**	**14·3 (9·59–16·8)**	**8·64 (6·62–9·78)**	**8·69 (6·68–10·1)**	**-39·2 (-49·7–-8·55)**	**0·590 (-12·0–15·8)**
South Asia	16·8 (12·9–18·7)	11·6 (9·77–12·6)	11·4 (9·71–12·7)	-31·7 (-41·0–-9·17)	-1·23 (-10·1–8·52)	19·0 (14·6–21·6)	14·5 (12·5–16·2)	14·2 (12·3–16·2)	-25·0 (-36·6–-1·68)	-2·15 (-13·7–10·2)	14·3 (9·59–16·8)	8·64 (6·62–9·78)	8·69 (6·68–10·1)	-39·2 (-49·7–-8·55)	0·590 (-12·0–15·8)
Bangladesh	8·10 (6·60–9·49)	3·76 (2·93–5·14)	3·66 (2·82–5·05)	-54·9 (-67·1–-33·5)	-2·60 (-9·97–4·74)	10·5 (7·99–12·4)	5·33 (4·16–6·71)	5·27 (4·04–6·84)	-49·8 (-63·5–-28·6)	-1·13 (-9·61–6·68)	5·44 (4·38–7·01)	2·21 (1·57–4·35)	2·10 (1·46–4·40)	-61·3 (-74·2–-32·2)	-4·91 (-13·9–4·61)
Bhutan	8·30 (5·59–10·8)	6·01 (4·49–8·26)	5·94 (4·40–8·21)	-28·4 (-50·6–18·0)	-1·17 (-8·55–5·04)	10·5 (6·65–13·8)	7·85 (5·69–10·6)	7·79 (5·53–10·6)	-25·7 (-49·3–26·8)	-0·738 (-8·74–5·49)	6·05 (3·85–8·62)	4·02 (2·80–6·42)	3·96 (2·75–6·15)	-34·6 (-58·0–36·5)	-1·56 (-10·1–6·18)
India	18·9 (14·2–21·3)	13·1 (10·6–14·3)	13·0 (10·5–14·5)	-31·5 (-41·1–-7·67)	-0·931 (-10·7–9·87)	20·9 (15·8–24·0)	16·1 (13·2–17·9)	15·7 (13·0–18·1)	-24·9 (-37·2–-0·362)	-2·19 (-15·2–11·4)	16·8 (10·8–19·9)	10·1 (7·25–11·5)	10·3 (7·26–12·0)	-38·7 (-50·2–-7·45)	1·31 (-12·4–17·5)
Nepal	18·2 (11·9–23·1)	14·3 (10·9–18·2)	14·2 (10·7–18·5)	-22·0 (-44·9–23·7)	-0·637 (-8·47–7·47)	26·4 (16·6–33·8)	21·3 (16·7–27·2)	21·4 (16·6–27·3)	-18·8 (-41·4–24·5)	0·545 (-7·28–8·90)	10·1 (5·88–13·9)	8·26 (4·61–11·8)	8·12 (4·52–11·8)	-19·2 (-50·2–59·0)	-1·65 (-11·6–9·31)

Pakistan	6·32 (4·45–8·42)	6·52 (4·34–9·85)	6·37 (4·36–9·53)	0·794 (-22·9–32·4)	-2·27 (-26·2–30·7)	9·36 (6·37–13·0)	9·70 (6·00–15·7)	9·50 (5·92–15·4)	1·47 (-29·9–40·7)	-2·05 (-30·7–36·3)	2·85 (1·50–5·62)	3·22 (1·87–6·41)	3·15 (1·92–6·39)	10·5 (-25·8–54·9)	-2·26 (-33·7–41·4)
**Southeast Asia, East Asia, and Oceania**	**17·6 (12·3–19·9)**	**6·75 (5·94–8·01)**	**6·69 (5·77–7·94)**	**-62·0 (-69·0–-44·8)**	**-0·938 (-15·4–16·1)**	**18·9 (13·4–21·8)**	**8·89 (7·42–10·7)**	**8·82 (7·36–10·7)**	**-53·2 (-63·3–-31·1)**	**-0·807 (-20·2–23·1)**	**16·6 (10·0–20·1)**	**4·81 (3·83–6·27)**	**4·75 (3·85–6·25)**	**-71·4 (-78·5–-54·7)**	**-1·14 (-23·8–31·2)**
East Asia	21·1 (14·3–24·1)	7·28 (6·25–8·83)	7·23 (5·99–8·80)	-65·7 (-72·6–-47·5)	-0·703 (-18·5–21·0)	21·8 (14·6–25·6)	9·15 (7·26–11·6)	9·08 (7·21–11·5)	-58·4 (-69·2–-35·0)	-0·759 (-25·0–31·8)	20·9 (12·2–25·3)	5·64 (4·32–7·34)	5·61 (4·37–7·51)	-73·2 (-80·6–-56·2)	-0·600 (-26·4–37·6)
China	21·5 (14·4–24·6)	7·16 (6·10–8·69)	7·12 (5·83–8·76)	-66·8 (-73·9–-49·1)	-0·600 (-19·4–22·3)	22·2 (14·6–26·1)	9·00 (7·05–11·5)	8·94 (7·01–11·4)	-59·7 (-70·4–-36·7)	-0·651 (-26·0–33·8)	21·3 (12·3–26·0)	5·57 (4·23–7·24)	5·54 (4·26–7·48)	-74·0 (-81·5–-57·3)	-0·544 (-27·3–39·8)
Democratic People's Republic of Korea	12·3 (8·81–17·0)	9·24 (6·69–14·8)	9·11 (6·60–14·8)	-26·1 (-49·3–5·36)	-1·42 (-8·96–5·77)	14·8 (10·7–20·3)	11·3 (8·16–18·0)	11·1 (7·94–17·8)	-24·7 (-48·5–7·69)	-1·17 (-8·29–5·83)	10·8 (7·35–16·6)	7·69 (4·97–14·2)	7·52 (4·75–14·3)	-30·5 (-54·4–3·44)	-2·23 (-10·8–5·65)
Taiwan (Province of China)	9·16 (8·90–9·43)	11·9 (11·4–12·3)	11·3 (10·5–12·0)	23·7 (14·2–30·8)	-4·67 (-9·94–-1·23)	11·0 (10·6–11·4)	16·5 (15·7–17·3)	15·6 (14·5–16·6)	41·9 (30·1–51·9)	-5·48 (-10·8–-1·78)	7·17 (6·87–7·43)	7·47 (7·15–7·80)	7·29 (6·72–7·78)	1·71 (-7·29–9·46)	-2·46 (-9·09–2·11)
Oceania	7·18 (5·99–8·42)	5·56 (4·76–6·72)	5·36 (4·57–6·42)	-25·4 (-34·7–-6·11)	-3·60 (-7·67–0·374)	10·0 (8·23–11·8)	7·75 (6·48–9·02)	7·50 (6·37–8·76)	-25·3 (-34·9–-6·77)	-3·24 (-7·74–0·742)	4·15 (3·33–5·21)	3·29 (2·65–4·68)	3·15 (2·52–4·55)	-24·1 (-37·2–2·00)	-4·27 (-9·42–0·414)
American Samoa	7·47 (6·21–9·10)	8·38 (6·59–10·2)	8·41 (6·45–10·5)	12·7 (-16·2–48·5)	0·420 (-6·67–7·42)	11·5 (9·11–14·3)	12·6 (9·61–15·7)	12·7 (9·64–16·0)	9·86 (-19·9–46·7)	0·655 (-7·72–8·52)	3·29 (2·58–4·28)	4·07 (3·06–5·20)	4·02 (2·94–5·25)	22·2 (-16·4–69·7)	-1·03 (-9·42–7·39)
Cook Islands	16·3 (13·0–20·3)	11·1 (8·89–14·1)	10·8 (8·54–13·8)	-33·5 (-50·9–-7·81)	-2·46 (-8·66–3·84)	22·6 (17·8–28·5)	18·1 (14·6–23·2)	17·7 (13·9–22·8)	-21·5 (-43·3–11·2)	-2·14 (-9·49–5·82)	9·57 (6·96–12·5)	4·89 (3·65–6·57)	4·79 (3·57–6·48)	-50·0 (-64·6–-22·6)	-2·09 (-10·0–5·68)
Fiji	11·1 (9·43–13·0)	9·35 (7·23–11·8)	9·15 (6·93–11·8)	-17·6 (-38·1–12·6)	-2·15 (-11·6–7·76)	14·9 (12·0–18·1)	13·1 (9·75–16·7)	12·8 (9·56–16·4)	-13·7 (-36·7–20·1)	-1·90 (-12·2–9·70)	7·38 (5·60–9·48)	5·71 (4·03–7·59)	5·58 (3·98–7·53)	-24·4 (-47·6–11·6)	-2·31 (-15·5–13·9)
Guam	16·6 (15·0–19·3)	19·8 (17·7–21·7)	16·7 (14·6–18·6)	0·876 (-16·2–16·6)	-15·4 (-20·5–-10·6)	25·1 (22·6–29·6)	31·1 (27·8–34·4)	28·5 (24·6–31·9)	13·5 (-6·80–32·8)	-8·51 (-15·0–-2·02)	6·69 (5·66–8·42)	7·78 (6·17–8·87)	4·26 (3·30–4·99)	-36·2 (-52·7–-21·9)	-45·2 (-50·5–-39·1)
Kiribati	18·2 (14·4–22·1)	17·8 (13·1–22·6)	17·6 (12·8–22·6)	-3·01 (-29·7–30·4)	-1·05 (-7·73–5·45)	31·6 (24·1–38·9)	31·8 (23·0–41·2)	31·5 (22·5–40·7)	-0·410 (-28·0–37·3)	-0·954 (-7·89–5·82)	5·77 (4·34–7·41)	5·18 (3·67–7·03)	5·12 (3·58–6·94)	-11·2 (-35·7–29·2)	-1·17 (-9·32–7·37)
Marshall Islands	23·7 (14·5–29·9)	20·3 (12·1–26·7)	20·0 (12·1–26·6)	-15·5 (-35·3–8·48)	-1·46 (-6·93–3·91)	36·2 (19·6–48·0)	29·2 (17·4–39·6)	28·8 (17·0–39·5)	-20·4 (-39·5–3·21)	-1·24 (-7·43–5·52)	10·9 (6·06–13·7)	10·9 (5·46–15·1)	10·7 (5·35–14·8)	-1·84 (-31·3–32·6)	-1·90 (-8·86–6·20)
Micronesia (Federated States of)	25·0 (16·4–31·5)	20·3 (13·8–26·5)	20·1 (13·6–26·2)	-19·7 (-41·7–8·92)	-0·906 (-6·64–5·12)	37·2 (23·4–47·1)	31·0 (20·5–40·7)	30·8 (20·6–40·3)	-17·1 (-39·4–12·6)	-0·648 (-6·34–5·84)	12·5 (7·54–17·0)	9·31 (6·15–12·7)	9·13 (5·95–12·7)	-27·1 (-49·8–11·4)	-1·99 (-9·22–6·15)
Nauru	29·2 (16·1–37·6)	25·1 (13·8–32·6)	24·6 (13·7–32·6)	-15·8 (-38·3–13·6)	-1·83 (-7·40–3·75)	42·9 (22·8–56·1)	38·1 (20·1–50·2)	37·4 (19·9–49·3)	-12·8 (-38·1–18·7)	-1·72 (-7·95–4·32)	14·1 (7·33–19·3)	12·5 (6·75–17·6)	12·2 (6·52–17·4)	-13·6 (-42·1–31·6)	-2·00 (-9·92–6·70)
Niue	16·0 (11·7–21·2)	12·4 (8·86–17·4)	15·4 (12·3–19·3)	-3·66 (-29·5–35·5)	23·8 (8·55–46·3)	24·3 (16·8–32·8)	19·3 (12·8–26·9)	20·4 (14·8–27·3)	-16·2 (-40·9–20·8)	5·82 (-3·67–19·1)	7·71 (5·21–10·9)	5·71 (3·90–8·46)	10·7 (8·37–14·3)	39·0 (-2·71–94·0)	87·9 (40·5–143)
Northern Mariana Islands	13·9 (10·2–18·5)	15·4 (12·0–19·8)	15·3 (12·8–17·8)	10·7 (-19·0–54·7)	-0·228 (-14·1–13·4)	21·6 (15·6–29·3)	24·2 (18·9–31·4)	24·3 (20·2–28·2)	12·7 (-16·4–56·3)	0·461 (-13·6–15·1)	4·80 (3·40–7·15)	5·59 (3·85–7·41)	5·60 (4·00–6·78)	16·5 (-28·8–74·6)	0·0571 (-14·4–15·5)
Palau	17·5 (11·3–26·9)	15·4 (12·5–19·0)	15·3 (12·4–19·4)	-12·6 (-42·7–43·5)	-0·441 (-6·24–5·68)	21·9 (12·7–37·6)	15·7 (12·3–19·8)	15·5 (12·2–19·9)	-29·2 (-59·1–29·5)	-0·793 (-7·85–5·98)	12·7 (8·26–19·3)	14·2 (10·6–18·7)	14·0 (10·4–18·6)	10·0 (-30·3–97·1)	-1·81 (-9·90–6·35)
Papua New Guinea	3·00 (2·20–4·03)	2·85 (2·17–4·43)	2·76 (2·08–4·31)	-8·02 (-38·5–41·3)	-2·86 (-11·7–5·60)	3·81 (2·68–5·24)	3·61 (2·78–5·57)	3·52 (2·68–5·40)	-7·74 (-41·0–42·5)	-2·61 (-12·6–6·73)	2·13 (1·44–3·22)	2·02 (1·38–3·84)	1·96 (1·34–3·70)	-8·01 (-39·6–48·3)	-3·11 (-12·1–5·75)

Samoa	16·0 (11·9–20·4)	13·8 (10·2–17·5)	13·6 (9·94–17·5)	-14·9 (-39·0–18·9)	-1·59 (-8·99–6·61)	23·5 (17·2–30·7)	19·5 (14·2–25·3)	19·3 (13·9–25·3)	-17·8 (-41·0–20·6)	-1·09 (-8·90–6·93)	8·00 (5·45–10·9)	7·87 (5·42–10·5)	7·65 (5·21–10·6)	-4·32 (-38·3–39·1)	-2·70 (-11·5–5·90)
Solomon Islands	21·9 (10·0–30·1)	20·3 (13·5–27·2)	19·9 (13·5–26·8)	-8·97 (-37·4–57·1)	-1·74 (-8·18–4·46)	32·9 (15·3–45·6)	30·6 (20·0–40·5)	30·2 (20·5–39·7)	-8·39 (-36·1–55·6)	-1·42 (-8·09–5·05)	10·0 (3·81–15·1)	9·97 (5·95–14·7)	9·72 (5·83–14·4)	-3·08 (-39·0–79·9)	-2·47 (-10·5–6·74)
Tokelau	16·0 (11·1–21·5)	11·2 (7·83–15·9)	15·7 (12·6–19·1)	-1·31 (-25·1–37·7)	41·0 (16·8–74·3)	21·9 (15·0–30·2)	15·9 (11·2–22·8)	19·6 (15·8–25·1)	-10·4 (-32·5–23·3)	23·0 (5·49–47·4)	10·5 (6·57–14·5)	6·24 (3·74–9·38)	11·9 (8·39–15·8)	13·9 (-17·9–67·8)	90·8 (47·0–154)
Tonga	5·34 (4·41–6·43)	5·23 (4·00–6·88)	5·19 (3·91–6·93)	-2·93 (-29·8–36·9)	-0·831 (-7·06–6·19)	6·39 (5·14–7·61)	6·96 (5·14–9·27)	6·93 (5·12–9·33)	8·54 (-21·9–53·5)	-0·432 (-7·73–8·06)	4·38 (3·39–5·71)	3·62 (2·60–5·12)	3·57 (2·54–5·04)	-18·4 (-45·8–24·6)	-1·24 (-9·32–7·46)
Tuvalu	22·0 (14·7–27·7)	16·9 (12·7–21·2)	16·6 (12·6–20·8)	-24·5 (-42·1–0·132)	-2·06 (-8·56–4·79)	32·2 (20·9–41·6)	24·4 (17·6–31·7)	23·9 (17·3–30·8)	-25·7 (-47·2–3·81)	-1·93 (-8·39–5·48)	13·6 (7·79–17·9)	8·86 (6·05–12·3)	8·59 (5·78–12·0)	-36·8 (-56·5–-0·195)	-3·11 (-11·4–5·19)
Vanuatu	19·7 (12·5–25·4)	17·8 (12·8–22·1)	17·6 (12·8–22·5)	-10·3 (-35·9–24·3)	-0·955 (-7·94–5·86)	30·1 (18·7–39·3)	27·8 (19·5–34·4)	27·6 (19·4–34·7)	-8·44 (-32·9–27·5)	-0·743 (-7·93–6·00)	8·86 (5·18–12·5)	8·08 (5·57–11·1)	7·98 (5·49–11·1)	-9·92 (-38·7–32·1)	-1·24 (-9·06–6·91)
Southeast Asia	7·04 (6·17–7·71)	4·80 (4·20–5·39)	4·70 (3·97–5·41)	-33·2 (-42·3–-20·9)	-2·12 (-9·51–5·76)	10·3 (9·05–11·5)	7·40 (6·42–8·29)	7·26 (6·17–8·43)	-29·3 (-39·3–-16·1)	-1·88 (-10·0–7·25)	4·07 (3·13–4·63)	2·35 (1·98–2·96)	2·28 (1·86–2·89)	-44·0 (-53·0–-22·6)	-2·93 (-11·1–6·37)
Cambodia	6·75 (5·40–8·08)	4·93 (3·69–6·48)	4·87 (3·58–6·44)	-27·8 (-48·6–9·90)	-1·04 (-8·13–6·32)	9·46 (7·45–11·6)	7·41 (5·58–9·74)	7·34 (5·42–9·67)	-22·4 (-45·1–16·6)	-0·947 (-8·33–6·22)	4·70 (3·19–6·10)	2·86 (1·95–4·03)	2·81 (1·86–4·02)	-40·2 (-61·1–14·7)	-1·66 (-10·9–8·22)
Indonesia	2·21 (1·79–2·50)	1·79 (1·49–2·15)	1·75 (1·44–2·15)	-21·0 (-36·1–2·47)	-2·50 (-23·0–28·7)	2·87 (2·32–3·33)	2·43 (1·91–3·07)	2·38 (1·86–3·10)	-16·9 (-37·1–10·7)	-1·98 (-28·5–37·2)	1·59 (1·13–1·95)	1·17 (0·882–1·54)	1·13 (0·828–1·55)	-29·1 (-48·2–7·08)	-3·63 (-29·4–33·0)
Lao People's Democratic Republic	10·6 (6·54–14·0)	5·67 (4·19–7·72)	5·54 (4·09–7·42)	-47·6 (-64·6–-15·3)	-2·32 (-9·32–5·53)	15·8 (10·5–21·5)	8·42 (6·16–11·6)	8·25 (5·97–11·6)	-47·7 (-65·3–-14·6)	-1·96 (-9·29–5·51)	5·87 (2·46–8·40)	3·01 (1·97–4·32)	2·91 (1·90–4·18)	-50·4 (-69·5–9·41)	-3·26 (-13·3–6·54)
Malaysia	6·80 (6·11–7·52)	5·11 (4·65–5·79)	5·07 (4·61–5·82)	-25·5 (-35·6–-13·1)	-0·773 (-4·04–2·32)	9·95 (8·85–11·3)	7·91 (7·09–9·06)	7·87 (7·01–8·99)	-20·9 (-31·7–-6·32)	-0·518 (-4·49–3·09)	3·73 (2·75–4·47)	2·16 (1·84–2·99)	2·12 (1·79–2·95)	-43·2 (-56·4–-20·9)	-1·67 (-6·49–3·94)
Maldives	5·03 (2·84–6·20)	2·09 (1·76–2·60)	2·02 (1·59–2·51)	-59·9 (-69·8–-18·5)	-3·68 (-15·8–6·41)	7·53 (4·34–9·70)	3·18 (2·63–3·97)	3·04 (2·40–3·80)	-59·7 (-70·3–-21·7)	-4·58 (-16·8–5·47)	1·97 (0·738–2·77)	0·474 (0·379–0·620)	0·454 (0·344–0·596)	-76·9 (-85·0–-32·4)	-4·09 (-16·1–5·97)
Mauritius	16·4 (15·7–17·1)	9·70 (9·15–10·3)	9·32 (8·58–9·79)	-43·2 (-48·3–-39·4)	-3·87 (-11·1–0·631)	22·9 (21·7–24·1)	16·1 (15·1–17·1)	15·5 (14·3–16·3)	-32·3 (-38·7–-27·2)	-3·87 (-11·0–1·54)	10·7 (10·1–11·5)	3·58 (3·30–3·85)	3·45 (3·10–3·70)	-67·8 (-71·4–-64·8)	-3·77 (-12·1–2·26)
Myanmar	5·82 (3·42–7·76)	3·66 (2·47–4·71)	3·61 (2·45–4·63)	-38·0 (-56·4–-5·32)	-1·35 (-8·16–6·21)	9·22 (4·80–12·4)	6·45 (4·21–8·31)	6·39 (4·22–8·30)	-30·7 (-51·6–7·81)	-0·926 (-8·21–6·92)	2·69 (1·37–3·75)	1·27 (0·913–1·71)	1·23 (0·886–1·69)	-54·2 (-70·5–0·399)	-2·76 (-9·82–5·67)
Philippines	4·29 (3·84–4·72)	3·90 (3·42–4·30)	3·84 (3·16–4·58)	-10·4 (-25·5–8·10)	-1·55 (-18·2–19·8)	6·85 (5·99–7·74)	6·35 (5·52–7·08)	6·26 (4·88–7·72)	-8·62 (-26·9–13·1)	-1·46 (-21·1–24·3)	1·74 (1·43–2·05)	1·49 (1·19–1·72)	1·45 (1·09–1·88)	-16·3 (-35·5–8·70)	-2·21 (-23·5–24·3)
Seychelles	8·62 (7·69–9·55)	6·65 (5·73–7·55)	5·11 (4·34–5·83)	-40·7 (-50·6–-31·0)	-23·1 (-29·0–-17·4)	15·8 (13·9–17·8)	11·8 (10·1–13·5)	8·89 (7·44–10·2)	-43·6 (-53·7–-33·3)	-24·7 (-30·4–-18·9)	1·92 (1·47–2·25)	1·16 (0·938–1·46)	1·06 (0·868–1·31)	-44·8 (-56·0–-29·4)	-8·14 (-19·9–5·18)
Sri Lanka	40·0 (35·6–43·8)	15·7 (11·8–20·9)	15·1 (10·3–20·6)	-62·3 (-74·5–-46·7)	-3·83 (-28·4–20·2)	60·7 (53·8–67·3)	26·2 (19·5–35·5)	25·2 (17·0–35·1)	-58·5 (-71·8–-40·8)	-3·69 (-29·5–21·9)	19·2 (14·8–22·2)	6·71 (4·92–9·67)	6·38 (4·43–9·18)	-66·7 (-77·8–-50·3)	-4·87 (-27·4–19·6)
Thailand	11·1 (9·42–13·5)	10·1 (8·60–11·7)	10·1 (7·80–12·5)	-9·46 (-32·2–17·2)	0·273 (-17·2–20·9)	16·8 (13·6–20·8)	17·1 (14·2–20·3)	17·2 (13·3–21·4)	2·54 (-24·4–34·6)	0·843 (-17·1–22·4)	5·89 (4·35–7·74)	3·55 (2·89–4·45)	3·48 (2·57–4·80)	-40·8 (-59·2–-13·8)	-1·95 (-21·6–21·7)
Timor-Leste	6·33 (4·51–8·18)	4·70 (3·49–6·41)	4·71 (3·46–6·47)	-25·5 (-48·2–10·9)	0·366 (-5·84–6·79)	8·46 (5·89–11·3)	6·67 (4·73–9·57)	6·74 (4·77–9·72)	-20·3 (-43·6–15·0)	1·16 (-6·27–9·09)	4·13 (2·21–5·76)	2·74 (1·87–3·84)	2·72 (1·90–3·84)	-34·3 (-58·9–12·8)	-0·892 (-9·96–8·57)

Viet Nam	9·72 (7·28–12·2)	7·76 (6·00–9·71)	7·65 (5·83–9·71)	-21·2 (-43·2–11·6)	-1·31 (-7·55–5·39)	12·8 (9·28–16·3)	11·1 (8·39–14·2)	11·0 (8·29–13·8)	-14·4 (-39·5–25·6)	-1·46 (-8·15–6·03)	7·20 (5·02–9·45)	4·75 (3·52–6·21)	4·68 (3·38–6·13)	-35·0 (-54·7–7·88)	-1·49 (-9·13–6·12)
**Sub-Saharan Africa**	**14·0 (12·3–15·4)**	**11·7 (10·3–13·2)**	**11·5 (10·1–13·2)**	**-17·6 (-27·1–-0·781)**	**-1·47 (-6·64–4·26)**	**22·1 (19·1–24·9)**	**19·3 (17·1–22·0)**	**19·1 (16·6–22·0)**	**-13·5 (-24·2–5·09)**	**-1·09 (-7·10–5·66)**	**6·37 (5·17–7·31)**	**4·98 (3·91–5·98)**	**4·92 (3·77–5·92)**	**-22·7 (-34·9–-0·698)**	**-1·05 (-6·77–5·21)**
Central Sub-Saharan Africa	16·4 (13·2–19·7)	14·4 (11·0–19·1)	14·4 (11·0–19·1)	-11·9 (-29·7–16·5)	-0·0158 (-4·97–5·57)	27·0 (21·4–33·3)	24·4 (18·4–32·2)	24·4 (18·6–32·6)	-9·42 (-29·8–20·5)	0·322 (-4·62–5·89)	6·79 (4·94–8·57)	6·25 (4·34–8·99)	6·23 (4·29–8·88)	-8·29 (-33·5–33·4)	-0·261 (-6·13–6·44)
Angola	18·4 (13·9–23·2)	13·7 (10·7–17·4)	13·8 (10·3–17·3)	-25·2 (-46·1–7·52)	0·438 (-6·75–8·77)	30·4 (22·3–38·7)	24·1 (18·8–31·0)	24·4 (18·3–30·7)	-19·8 (-42·3–14·3)	1·09 (-6·14–9·44)	6·65 (4·76–8·88)	5·28 (3·61–7·21)	5·33 (3·57–7·39)	-19·9 (-46·2–30·5)	0·891 (-8·89–10·7)
Central African Republic	22·3 (17·4–27·8)	20·2 (14·4–26·7)	19·6 (14·0–26·2)	-11·9 (-32·9–14·6)	-2·69 (-8·22–3·26)	38·1 (28·9–50·1)	34·9 (24·9–46·8)	34·1 (24·7–45·9)	-10·4 (-30·9–15·7)	-2·18 (-7·77–3·59)	8·99 (5·73–11·6)	8·10 (5·03–11·3)	7·97 (4·97–11·3)	-11·4 (-35·0–23·9)	-1·68 (-8·66–5·71)
Congo	20·0 (15·9–24·5)	14·4 (10·9–18·4)	14·2 (10·9–18·2)	-28·8 (-47·2–-1·86)	-1·04 (-7·20–6·20)	33·9 (27·3–42·1)	22·2 (16·7–28·5)	22·2 (16·5–28·6)	-34·7 (-52·6–-9·33)	-0·302 (-6·45–6·96)	8·49 (5·56–11·3)	7·15 (4·69–9·49)	6·94 (4·53–9·34)	-18·2 (-44·3–30·5)	-2·93 (-10·8–4·96)
Democratic Republic of the Congo	15·1 (12·0–19·0)	14·4 (10·4–20·5)	14·4 (10·3–20·6)	-4·79 (-28·6–28·6)	0·281 (-6·19–7·71)	24·6 (18·8–32·0)	24·0 (17·1–33·4)	24·1 (17·6–34·7)	-1·85 (-28·4–36·7)	0·505 (-6·12–7·99)	6·56 (4·64–8·70)	6·44 (4·34–10·1)	6·44 (4·28–10·2)	-1·87 (-32·1–41·3)	-0·0637 (-7·95–8·27)
Equatorial Guinea	20·4 (15·3–25·6)	12·2 (8·35–17·8)	12·1 (8·45–17·3)	-40·8 (-60·0–-7·13)	-0·689 (-8·32–8·80)	36·1 (26·3–46·2)	21·1 (15·1–29·4)	20·9 (14·9–29·0)	-42·0 (-60·0–-10·8)	-0·636 (-7·87–8·56)	7·93 (5·27–10·6)	4·78 (2·57–7·93)	4·75 (2·66–8·04)	-40·1 (-65·2–21·8)	-0·530 (-11·5–11·6)
Gabon	16·9 (13·3–21·1)	14·1 (10·0–18·9)	13·7 (10·0–18·4)	-19·0 (-39·5–10·2)	-3·44 (-9·68–3·69)	29·7 (23·1–38·1)	25·3 (18·3–33·5)	24·5 (18·2–31·9)	-17·6 (-39·5–10·5)	-3·00 (-9·21–4·31)	5·88 (4·08–7·40)	4·70 (2·99–7·24)	4·54 (2·94–7·22)	-22·8 (-47·7–16·8)	-3·44 (-11·8–5·89)
Eastern Sub-Saharan Africa	16·6 (14·4–18·4)	12·4 (10·8–14·4)	12·2 (10·6–14·4)	-26·5 (-37·3–-9·56)	-1·35 (-7·44–4·51)	25·9 (22·0–29·6)	20·1 (17·4–23·8)	19·9 (17·3–23·9)	-23·1 (-35·6–-7·48)	-1·09 (-8·02–6·73)	7·65 (5·82–9·20)	5·39 (4·43–6·56)	5·35 (4·39–6·63)	-30·1 (-43·2–-5·21)	-0·688 (-8·38–7·79)
Burundi	21·7 (15·3–31·4)	14·0 (10·9–20·6)	13·8 (10·6–20·1)	-36·2 (-53·4–-11·0)	-1·13 (-8·12–5·48)	35·7 (23·9–57·0)	21·5 (16·3–32·1)	21·3 (15·8–31·4)	-40·5 (-56·9–-13·0)	-1·19 (-8·63–5·77)	9·88 (6·21–14·0)	6·23 (4·41–9·23)	6·16 (4·18–9·36)	-37·7 (-58·6–-0·802)	-1·10 (-8·73–7·01)
Comoros	10·6 (6·82–15·5)	8·13 (5·61–12·6)	8·26 (5·72–12·7)	-22·3 (-44·3–11·8)	1·58 (-5·94–8·67)	14·2 (8·52–23·2)	10·7 (6·82–18·9)	10·9 (6·73–19·3)	-23·3 (-47·1–10·8)	1·60 (-6·71–9·77)	7·33 (4·48–9·64)	5·82 (4·10–7·91)	5·93 (4·17–8·15)	-19·0 (-44·6–28·6)	1·85 (-6·05–9·98)
Djibouti	8·56 (5·62–13·7)	8·80 (5·34–14·8)	8·65 (5·36–14·5)	1·06 (-29·9–44·6)	-1·75 (-9·18–6·34)	11·7 (6·90–21·5)	12·0 (6·79–22·2)	11·8 (6·80–21·7)	1·14 (-29·9–46·0)	-1·65 (-9·01–6·83)	5·53 (3·87–7·77)	5·29 (3·34–7·91)	5·21 (3·35–7·80)	-5·77 (-39·5–47·8)	-1·53 (-11·2–8·26)
Eritrea	18·2 (14·5–22·6)	15·8 (11·8–20·7)	15·7 (11·6–21·1)	-13·8 (-34·5–13·8)	-0·604 (-5·43–5·30)	30·0 (22·0–40·5)	26·0 (18·5–35·2)	25·7 (18·5–35·1)	-14·3 (-34·2–11·9)	-1·35 (-6·56–5·09)	9·42 (6·32–12·2)	8·04 (5·67–10·8)	8·06 (5·77–10·8)	-14·4 (-37·5–24·0)	0·360 (-7·01–7·82)
Ethiopia	22·6 (15·5–27·0)	11·3 (9·62–13·2)	11·3 (9·43–13·5)	-49·8 (-62·1–-23·6)	-0·119 (-17·3–21·0)	34·5 (23·7–44·5)	17·4 (14·2–21·4)	17·4 (14·0–21·3)	-49·5 (-64·9–-25·5)	0·365 (-20·8–28·5)	10·3 (5·58–13·8)	5·07 (3·84–6·56)	5·11 (3·96–6·76)	-50·4 (-64·6–7·21)	0·834 (-21·9–33·3)
Kenya	11·4 (8·91–17·7)	11·9 (9·67–15·6)	11·7 (9·11–15·8)	2·08 (-19·1–29·9)	-2·34 (-21·3–20·8)	16·8 (12·4–28·7)	19·7 (15·0–26·7)	19·3 (14·4–27·6)	14·8 (-13·5–51·5)	-1·92 (-23·3–27·7)	6·16 (4·68–8·84)	5·43 (3·96–7·96)	5·29 (3·70–7·92)	-14·2 (-37·2–18·5)	-2·63 (-28·2–34·6)
Madagascar	13·4 (11·2–16·5)	11·1 (8·09–14·9)	11·0 (7·95–14·9)	-17·9 (-39·1–8·45)	-0·499 (-7·67–6·95)	19·8 (15·8–25·8)	16·7 (11·9–23·3)	16·7 (11·6–23·0)	-15·8 (-38·5–13·2)	-0·295 (-7·67–7·78)	7·02 (5·34–8·54)	5·94 (4·12–7·91)	5·94 (4·16–7·91)	-15·3 (-39·1–15·0)	-0·0743 (-7·35–8·08)
Malawi	15·3 (12·4–18·1)	15·2 (12·2–18·6)	14·8 (11·7–18·5)	-3·34 (-22·6–23·9)	-2·89 (-9·76–4·02)	24·9 (19·1–31·1)	27·4 (21·6–33·8)	26·6 (20·5–33·1)	6·68 (-18·0–41·0)	-2·96 (-9·81–4·29)	6·71 (5·02–8·57)	5·39 (3·84–7·69)	5·30 (3·62–7·92)	-20·9 (-47·0–27·5)	-1·62 (-10·5–7·69)
Mozambique	14·4 (11·8–17·4)	18·4 (13·6–23·7)	17·7 (12·8–22·9)	22·9 (-11·0–65·7)	-3·90 (-11·2–4·16)	24·1 (19·0–30·1)	34·7 (25·5–44·1)	33·3 (24·3–42·8)	38·1 (1·82–88·4)	-3·87 (-11·4–4·36)	6·02 (4·71–7·55)	5·98 (3·91–8·28)	5·85 (3·79–8·14)	-2·93 (-35·0–40·8)	-2·28 (-13·4–8·83)

Rwanda	23·6 (18·0–29·2)	11·9 (8·77–16·3)	11·9 (8·63–16·6)	-49·4 (-63·7–-27·9)	0·179 (-7·37–8·27)	36·8 (28·2–47·6)	20·3 (14·6–27·8)	20·4 (14·5–28·4)	-44·6 (-61·0–-23·4)	0·355 (-7·63–9·16)	12·9 (8·07–17·6)	5·82 (3·77–8·77)	5·81 (3·68–8·76)	-54·9 (-70·3–-25·9)	-0·0829 (-7·99–9·20)
Somalia	13·6 (8·37–22·2)	13·2 (8·37–22·0)	12·9 (8·39–21·9)	-4·95 (-29·5–32·9)	-1·99 (-8·75–4·60)	18·2 (9·14–33·8)	18·4 (9·98–35·3)	18·0 (9·61–34·5)	-1·17 (-28·1–36·2)	-2·21 (-9·14–4·60)	9·43 (6·01–13·6)	9·26 (5·91–15·5)	9·12 (5·74–15·3)	-3·22 (-32·8–37·5)	-1·46 (-9·17–5·64)
South Sudan	14·8 (10·3–20·0)	15·0 (11·0–20·4)	15·1 (11·1–20·3)	1·53 (-26·0–44·4)	0·643 (-6·66–7·77)	21·5 (14·5–30·1)	22·7 (16·0–32·1)	23·0 (16·1–32·3)	6·91 (-22·7–53·4)	1·18 (-6·54–8·58)	6·28 (4·37–8·67)	6·61 (4·66–9·16)	6·81 (4·95–9·55)	8·47 (-26·6–63·3)	3·04 (-4·79–11·4)
Uganda	13·1 (10·2–16·1)	11·8 (9·14–14·6)	11·6 (8·84–14·5)	-11·5 (-34·0–25·8)	-1·50 (-7·96–5·10)	22·9 (16·7–28·3)	22·1 (16·9–27·1)	21·8 (16·7–27·0)	-4·67 (-29·8–33·4)	-1·45 (-7·97–5·58)	4·15 (2·84–5·91)	3·82 (2·74–5·13)	3·82 (2·72–5·18)	-7·88 (-38·7–46·2)	0·187 (-8·29–8·91)
United Republic of Tanzania	13·0 (10·9–15·4)	9·81 (7·59–12·7)	9·77 (7·53–13·1)	-25·1 (-43·7–0·0711)	-0·392 (-8·07–7·50)	21·2 (17·2–25·2)	15·9 (12·4–20·7)	15·9 (12·2–20·8)	-25·0 (-44·1–2·78)	0·142 (-7·72–8·82)	5·66 (4·45–7·02)	4·48 (3·27–6·02)	4·42 (3·21–6·01)	-21·9 (-44·8–13·3)	-1·19 (-9·29–7·39)
Zambia	16·5 (13·9–19·6)	15·4 (11·7–19·7)	14·9 (11·2–19·4)	-9·95 (-35·0–21·2)	-3·60 (-10·4–3·37)	25·2 (20·8–30·0)	26·1 (19·8–33·2)	25·3 (19·0–32·1)	0·436 (-28·3–33·8)	-3·31 (-10·3–3·70)	7·26 (5·58–9·09)	5·53 (3·74–7·85)	5·41 (3·59–7·78)	-25·6 (-50·2–14·3)	-2·20 (-11·0–7·07)
Southern Sub-Saharan Africa	18·0 (14·3–21·2)	15·9 (14·0–18·1)	16·1 (14·0–18·3)	-10·5 (-22·1–2·39)	1·07 (-5·24–7·27)	28·3 (23·2–34·2)	26·5 (23·6–30·4)	26·7 (23·2–30·6)	-5·61 (-18·7–7·51)	0·874 (-6·10–8·58)	8·85 (6·15–10·4)	6·46 (5·09–7·76)	6·49 (5·09–8·05)	-26·6 (-41·3–-9·24)	0·531 (-7·37–8·82)
Botswana	16·3 (11·6–22·6)	10·7 (8·01–14·8)	10·5 (7·65–14·6)	-35·4 (-57·2–-1·33)	-1·61 (-13·5–8·88)	27·8 (19·3–38·7)	18·0 (13·0–24·9)	17·7 (12·5–24·9)	-36·3 (-57·1–-1·72)	-1·61 (-13·4–8·64)	6·73 (4·30–11·6)	4·39 (2·92–7·64)	4·23 (2·83–7·57)	-37·1 (-60·4–-1·63)	-3·67 (-19·4–10·9)
Eswatini	16·5 (12·8–20·8)	23·4 (16·1–30·9)	21·9 (15·2–29·3)	32·6 (-9·21–94·0)	-6·52 (-13·9–1·46)	28·4 (21·6–37·2)	42·3 (29·8–55·2)	39·1 (27·5–52·0)	37·3 (-3·17–96·2)	-7·66 (-14·4–-0·373)	6·93 (5·22–9·70)	7·71 (4·27–12·1)	7·40 (4·04–11·8)	6·76 (-40·2–74·4)	-3·97 (-14·7–8·73)
Lesotho	13·2 (9·44–22·3)	25·0 (17·5–33·6)	24·3 (17·2–32·9)	83·5 (21·0–159)	-2·78 (-10·3–4·42)	23·7 (16·7–39·0)	42·0 (31·2–55·7)	40·6 (29·3–53·9)	71·7 (15·2–137)	-3·32 (-10·3–3·25)	6·76 (3·32–13·5)	11·3 (5·93–17·0)	10·8 (5·62–16·6)	60·3 (-9·25–172)	-3·74 (-13·4–6·56)
Namibia	13·6 (10·9–16·4)	14·1 (10·2–18·8)	13·7 (9·83–18·6)	0·699 (-30·2–38·3)	-2·94 (-11·1–6·18)	22·9 (18·0–28·6)	25·9 (18·8–34·3)	25·0 (18·0–33·1)	9·40 (-23·6–48·6)	-3·33 (-10·8–5·40)	5·62 (4·18–7·68)	4·36 (2·58–7·33)	4·31 (2·64–7·14)	-23·3 (-50·4–21·1)	-1·03 (-12·0–11·5)
South Africa	19·2 (15·5–22·1)	14·3 (13·1–16·5)	14·7 (13·2–16·7)	-23·2 (-32·0–-9·65)	2·63 (-5·22–11·5)	29·8 (24·5–35·8)	24·2 (21·8–27·6)	24·8 (21·8–28·0)	-16·8 (-27·8–-4·47)	2·52 (-6·95–13·0)	9·55 (6·95–11·1)	5·29 (4·46–7·04)	5·36 (4·46–7·18)	-43·9 (-54·8–-23·5)	1·33 (-11·0–14·6)
Zimbabwe	14·6 (9·46–19·5)	24·3 (15·7–31·4)	23·8 (15·3–31·1)	63·4 (13·9–120)	-2·05 (-8·51–4·62)	22·3 (16·4–30·6)	38·7 (26·2–49·7)	38·1 (25·7–49·6)	70·9 (19·1–130)	-1·74 (-8·62–5·36)	7·53 (3·17–10·0)	12·4 (5·69–16·9)	12·1 (5·66–16·8)	60·9 (-9·25–138)	-1·94 (-9·73–6·51)
Western Sub-Saharan Africa	10·2 (8·13–11·9)	9·75 (7·65–11·4)	9·49 (7·54–11·0)	-6·81 (-24·6–24·6)	-2·66 (-14·5–10·7)	16·0 (12·3–19·2)	15·9 (12·9–18·9)	15·6 (12·6–18·5)	-2·26 (-22·7–32·9)	-1·99 (-15·8–14·5)	4·48 (3·58–5·57)	4·09 (2·84–5·09)	4·00 (2·73–5·07)	-10·6 (-32·0–19·3)	-2·00 (-14·0–12·1)
Benin	10·3 (8·58–12·4)	9·95 (7·32–14·0)	9·84 (7·14–14·0)	-4·35 (-31·8–36·3)	-1·03 (-8·39–6·42)	16·3 (13·1–19·7)	16·5 (12·4–23·2)	16·2 (12·1–23·4)	-0·171 (-29·1–42·7)	-1·27 (-8·44–6·11)	4·92 (3·71–6·10)	4·35 (2·43–6·05)	4·35 (2·49–6·23)	-11·4 (-38·7–32·6)	0·103 (-7·72–7·81)
Burkina Faso	14·1 (11·5–16·7)	12·9 (10·2–16·1)	12·6 (10·2–16·3)	-10·4 (-29·5–18·5)	-2·15 (-9·51–5·29)	22·2 (17·6–26·7)	21·5 (16·9–27·2)	21·0 (16·6–27·6)	-5·36 (-26·3–28·6)	-1·91 (-9·34–5·78)	6·86 (5·28–8·78)	5·36 (3·78–7·39)	5·26 (3·74–7·38)	-23·4 (-43·8–10·8)	-1·86 (-9·74–6·64)
Cabo Verde	14·4 (11·6–16·9)	16·1 (12·7–19·9)	15·9 (12·3–20·1)	9·86 (-15·7–44·8)	-1·61 (-8·20–5·14)	25·7 (20·6–30·7)	29·2 (22·6–36·7)	28·7 (22·0–36·1)	11·7 (-14·9–46·5)	-1·59 (-8·56–5·81)	6·19 (4·09–7·86)	4·54 (3·46–5·70)	4·44 (3·27–5·72)	-28·3 (-49·4–7·80)	-2·32 (-10·1–5·15)
Cameroon	11·4 (9·55–13·8)	12·7 (8·69–17·8)	12·4 (8·26–17·6)	8·36 (-24·7–53·2)	-3·03 (-10·3–4·84)	17·3 (14·2–21·5)	20·7 (14·4–28·6)	20·1 (13·2–28·6)	16·0 (-20·7–65·0)	-2·89 (-10·1–5·07)	5·84 (4·21–7·17)	5·33 (2·89–7·65)	5·19 (2·81–7·58)	-11·1 (-41·4–34·4)	-2·67 (-11·9–6·10)
Chad	8·25 (5·93–12·0)	11·7 (7·91–18·8)	11·7 (7·94–18·9)	42·5 (4·70–88·8)	0·472 (-7·08–7·49)	11·3 (7·47–18·7)	16·3 (10·7–28·7)	16·4 (10·9–29·4)	45·5 (4·57–101)	0·656 (-7·83–8·29)	5·54 (3·85–7·60)	6·76 (4·22–9·13)	6·79 (4·18–9·23)	22·7 (-12·6–72·2)	0·470 (-7·86–7·93)

Côte d'Ivoire	14·1 (11·7–17·2)	12·9 (9·50–17·5)	12·5 (9·15–17·5)	-11·3 (-31·7–20·1)	-3·12 (-9·00–3·16)	23·5 (19·3–28·7)	21·4 (15·9–29·2)	20·8 (15·5–29·0)	-11·8 (-31·7–16·9)	-3·17 (-9·62–3·74)	3·73 (2·48–4·92)	4·03 (2·25–5·88)	3·95 (2·22–5·94)	5·96 (-27·2–55·4)	-1·89 (-10·2–6·82)
Gambia	7·95 (5·34–12·1)	9·74 (6·72–14·6)	9·57 (6·37–14·2)	20·3 (-13·0–65·8)	-1·82 (-8·60–4·64)	10·3 (6·31–17·8)	14·1 (8·80–22·8)	13·9 (8·59–22·7)	34·3 (-7·64–89·1)	-1·37 (-8·87–6·76)	5·50 (4·02–7·43)	5·76 (4·13–7·77)	5·64 (3·99–7·66)	2·63 (-27·5–48·5)	-2·08 (-9·67–6·77)
Ghana	7·06 (5·62–8·92)	9·05 (7·04–11·4)	8·70 (6·72–11·1)	23·2 (-14·1–72·0)	-3·93 (-10·9–4·65)	12·3 (9·67–15·2)	15·9 (12·4–19·8)	15·4 (11·9–19·7)	25·3 (-9·40–69·6)	-2·70 (-9·75–5·83)	2·21 (1·48–5·06)	3·62 (2·67–5·03)	3·42 (2·51–4·82)	54·5 (-26·7–158)	-5·77 (-13·4–3·25)
Guinea	7·02 (5·09–9·84)	9·06 (6·06–13·4)	8·88 (5·85–13·4)	26·5 (-14·1–91·3)	-2·00 (-8·49–5·50)	8·75 (5·58–14·2)	12·5 (7·92–20·1)	12·3 (7·70–19·7)	40·1 (-2·55–111)	-1·93 (-8·57–5·55)	5·37 (3·68–7·08)	5·83 (3·49–8·00)	5·73 (3·47–7·96)	6·70 (-30·6–66·8)	-1·74 (-8·87–7·00)
Guinea-Bissau	14·8 (9·59–20·1)	15·5 (11·5–20·0)	15·1 (11·2–19·5)	1·87 (-25·9–47·8)	-2·98 (-9·34–3·71)	22·5 (13·4–32·3)	24·4 (17·2–32·8)	23·8 (17·0–32·1)	5·58 (-22·7–56·4)	-2·62 (-9·77–4·36)	7·76 (4·83–10·6)	8·06 (5·32–10·6)	7·84 (5·12–10·2)	1·07 (-27·6–53·7)	-2·74 (-9·89–4·73)
Liberia	9·69 (7·44–12·0)	11·2 (7·55–15·4)	11·3 (7·59–16·0)	16·7 (-16·0–78·8)	1·45 (-5·22–8·60)	13·0 (9·98–16·6)	15·3 (10·8–20·3)	15·6 (11·1–21·7)	19·4 (-14·2–85·8)	1·99 (-4·91–9·47)	6·02 (3·84–8·11)	6·83 (3·58–11·4)	6·88 (3·61–11·8)	14·2 (-22·6–78·6)	0·634 (-7·65–9·07)
Mali	8·12 (5·48–11·6)	8·22 (5·78–11·7)	8·10 (5·64–11·8)	-0·351 (-24·6–33·9)	-1·48 (-8·21–6·09)	9·87 (6·25–16·3)	10·7 (7·23–17·0)	10·6 (7·00–16·9)	7·51 (-20·9–52·2)	-1·03 (-8·63–7·83)	6·43 (3·82–8·81)	5·63 (3·51–7·93)	5·54 (3·41–7·91)	-13·8 (-39·4–25·6)	-1·55 (-9·55–7·16)
Mauritania	6·72 (4·79–10·1)	5·75 (3·79–9·51)	5·88 (3·87–9·83)	-12·5 (-35·9–27·8)	2·17 (-4·43–10·1)	9·29 (6·03–16·0)	8·01 (4·91–15·2)	8·25 (5·01–15·4)	-11·2 (-36·1–29·7)	2·95 (-4·11–11·2)	4·44 (2·80–6·66)	3·53 (2·00–5·14)	3·57 (2·08–5·23)	-19·7 (-44·2–26·6)	1·03 (-6·77–9·70)
Niger	7·64 (5·49–11·2)	7·98 (5·36–12·1)	7·88 (5·20–12·1)	3·11 (-26·2–51·5)	-1·27 (-8·11–5·34)	9·86 (6·23–16·3)	10·8 (6·74–18·0)	10·7 (6·38–18·3)	8·08 (-23·7–66·5)	-1·07 (-8·66–6·05)	5·41 (3·52–6·89)	5·33 (3·30–7·24)	5·28 (3·19–7·45)	-2·45 (-32·5–49·8)	-0·860 (-7·78–8·10)
Nigeria	10·4 (6·83–13·4)	8·60 (6·06–11·4)	8·31 (5·93–10·6)	-20·0 (-42·1–14·8)	-3·37 (-27·8–30·4)	16·9 (10·0–22·3)	14·7 (9·35–20·3)	14·4 (9·34–19·3)	-15·0 (-41·5–29·4)	-2·32 (-31·2–40·4)	3·88 (2·69–5·53)	3·06 (1·89–4·72)	3·02 (1·89–4·80)	-22·2 (-51·0–23·1)	-1·36 (-30·7–43·1)
Sao Tome and Principe	1·50 (1·01–1·84)	1·56 (1·14–2·05)	1·54 (1·13–2·07)	2·86 (-25·5–45·1)	-0·980 (-7·78–6·80)	2·39 (1·80–2·97)	2·42 (1·78–3·11)	2·40 (1·75–3·23)	0·333 (-28·1–44·0)	-0·655 (-8·40–7·43)	0·760 (0·404–0·981)	0·745 (0·447–1·06)	0·729 (0·426–1·04)	-4·14 (-33·7–38·3)	-2·20 (-9·12–5·64)
Senegal	13·3 (10·6–16·1)	12·9 (10·3–16·6)	12·5 (9·68–16·4)	-5·96 (-29·0–38·0)	-2·72 (-9·07–3·60)	21·1 (16·7–25·8)	21·0 (16·7–27·1)	20·6 (15·9–27·0)	-2·38 (-27·1–46·6)	-2·07 (-8·73–4·98)	5·67 (4·36–7·18)	5·35 (3·95–7·41)	5·18 (3·76–7·08)	-8·71 (-36·4–37·5)	-3·15 (-10·7–4·29)
Sierra Leone	7·14 (4·87–10·1)	8·61 (5·78–12·6)	8·51 (5·66–12·6)	19·1 (-16·1–73·0)	-1·25 (-9·11–6·72)	10·1 (6·69–15·8)	11·7 (7·67–18·9)	11·5 (7·35–18·5)	13·7 (-21·4–72·0)	-1·24 (-9·54–7·72)	4·09 (2·64–5·41)	5·62 (3·14–7·63)	5·54 (3·03–7·60)	35·3 (-5·64–94·3)	-1·49 (-9·78–6·33)
Togo	10·2 (8·12–12·8)	12·8 (8·67–16·9)	12·5 (8·80–17·1)	22·5 (-12·8–68·3)	-2·18 (-9·16–5·41)	15·8 (12·4–20·0)	22·2 (15·5–29·5)	21·7 (15·0–29·8)	37·6 (-2·41–89·7)	-2·28 (-9·59–5·14)	5·33 (3·77–6·73)	5·54 (3·18–8·01)	5·46 (3·12–7·93)	2·60 (-29·5–47·0)	-1·43 (-9·30–7·70)

Appendix Table S6: Mean age at the time of death due to suicide, for males, females, and both sexes combined in 1990 and 2021. Estimates provided at the global, super-region, regional, and national level.

	Males		Female		Both	
**Location**	**1990**	**2021**	**1990**	**2021**	**1990**	**2021**
**Global**	**43·0 (38·0–45·8)**	**47·0 (43·5–50·6)**	**41·9 (30·9–46·7)**	**46·9 (41·2–52·8)**	**42·6 (36·8–45·1)**	**47·0 (43·3–50·3)**
Low SDI	44·4 (36·7–51·2)	43·6 (37·8–51·4)	37·7 (28·7–46·8)	39·9 (32·6–47·9)	42·3 (35·9–47·6)	42·5 (37·3–49·1)
Low-middle SDI	37·1 (29·3–41·6)	40·8 (34·2–45·4)	31·2 (21·2–36·3)	37·2 (28·9–42·8)	34·6 (27·1–38·3)	39·5 (32·0–43·4)
Middle SDI	41·6 (31·4–47·3)	47·7 (42·5–53·9)	41·1 (23·8–49·3)	49·8 (39·3–59·7)	41·4 (29·3–45·9)	48·4 (42·2–54·1)
High-middle SDI	45·4 (40·6–48·7)	50·7 (46·0–56·4)	47·9 (37·6–54·4)	57·0 (48·8–70·5)	46·3 (40·6–49·4)	52·5 (47·9–58·5)
High SDI	46·4 (45·5–49·2)	51·2 (48·8–52·7)	51·6 (49·0–55·2)	52·5 (48·2–55·4)	47·9 (46·5–50·5)	51·5 (48·7–53·2)
**Central Europe, Eastern Europe, and Central Asia**	**44·4 (43·9–44·9)**	**47·7 (44·5–51·0)**	**52·8 (51·3–53·8)**	**53·6 (49·5–58·0)**	**46·3 (45·7–46·8)**	**48·8 (46·0–51·7)**
Central Asia	39·0 (37·4–40·8)	41·1 (37·2–45·1)	42·1 (40·2–44·3)	39·7 (35·4–44·1)	39·8 (38·3–41·5)	40·7 (36·7–44·8)
Armenia	41·0 (39·1–43·3)	51·0 (44·6–58·8)	43·7 (40·4–47·1)	61·0 (53·2–71·3)	41·9 (40·1–43·6)	53·3 (46·6–61·6)
Azerbaijan	38·7 (33·9–44·6)	42·1 (30·7–57·9)	40·6 (33·8–48·9)	47·8 (34·4–65·2)	39·2 (34·9–44·2)	43·4 (32·0–58·0)
Georgia	44·5 (41·5–47·9)	49·2 (43·7–55·2)	49·4 (45·7–53·1)	58·5 (51·5–65·8)	45·9 (42·9–49·1)	51·0 (45·5–57·0)
Kazakhstan	39·9 (36·8–43·1)	43·3 (38·3–48·9)	45·9 (42·3–49·2)	45·2 (38·9–51·5)	41·3 (38·5–44·3)	43·7 (38·4–49·4)
Kyrgyzstan	39·7 (36·5–43·2)	39·4 (33·4–46·0)	41·5 (38·0–44·9)	42·2 (35·5–49·4)	40·1 (37·1–43·4)	40·0 (34·0–46·6)
Mongolia	34·7 (25·9–45·5)	39·3 (31·1–49·0)	34·9 (24·1–50·2)	41·6 (31·6–54·3)	34·8 (26·8–44·8)	39·6 (32·5–48·4)
Tajikistan	38·3 (31·7–45·4)	38·6 (27·1–55·9)	35·9 (28·6–43·6)	37·8 (26·3–56·9)	37·5 (32·0–42·9)	38·4 (29·2–53·5)
Turkmenistan	35·8 (32·9–38·6)	38·0 (29·9–49·3)	36·2 (33·7–39·3)	39·6 (30·6–52·3)	35·9 (33·5–38·4)	38·4 (30·1–49·8)
Uzbekistan	37·2 (35·2–39·4)	37·9 (32·4–44·4)	37·6 (35·6–39·5)	33·4 (28·6–39·0)	37·3 (35·6–39·0)	36·5 (31·6–42·7)
Central Europe	47·1 (46·0–48·1)	52·6 (48·8–56·2)	52·8 (50·9–54·6)	57·9 (52·5–63·6)	48·4 (47·4–49·5)	53·6 (49·7–57·3)

Albania	39·6 (32·9–47·2)	52·7 (39·7–69·0)	41·0 (33·6–49·4)	56·1 (39·4–74·2)	40·2 (35·2–46·5)	53·9 (42·3–66·7)
Bosnia and Herzegovina	41·2 (36·8–46·3)	54·7 (38·9–69·1)	48·2 (41·5–57·7)	60·1 (43·6–85·4)	42·8 (38·8–47·4)	55·8 (41·6–68·2)
Bulgaria	52·3 (49·2–55·4)	56·6 (48·3–65·3)	55·3 (52·0–58·8)	59·4 (51·3–67·7)	53·3 (50·7–55·7)	57·3 (49·5–65·3)
Croatia	51·6 (49·1–54·4)	58·5 (51·3–65·7)	56·6 (52·8–60·6)	62·4 (54·0–69·9)	53·0 (50·7–55·7)	59·4 (52·3–66·1)
Czechia	49·7 (47·8–51·7)	54·7 (47·9–61·7)	56·0 (53·4–58·8)	58·6 (51·3–65·8)	51·4 (49·7–53·2)	55·5 (48·9–61·9)
Hungary	50·2 (47·9–52·6)	56·1 (50·3–62·1)	56·5 (54·3–58·7)	60·0 (52·7–66·2)	52·0 (50·0–54·0)	57·1 (50·9–63·0)
Montenegro	44·5 (37·6–53·1)	53·0 (42·8–65·0)	48·2 (37·2–65·8)	58·3 (45·3–76·2)	45·3 (39·0–53·2)	54·2 (45·4–65·0)
North Macedonia	47·0 (39·9–54·7)	53·7 (40·7–67·5)	47·7 (38·9–57·9)	57·4 (42·9–76·0)	47·3 (41·9–53·3)	54·8 (42·5–67·7)
Poland	43·1 (42·3–43·9)	48·6 (43·5–53·5)	48·4 (47·1–49·4)	53·0 (46·9–58·7)	44·0 (43·2–44·8)	49·1 (44·6–53·7)
Romania	46·6 (44·6–48·9)	53·2 (46·7–59·9)	49·9 (47·7–52·1)	57·3 (50·6–64·5)	47·5 (45·8–49·2)	53·9 (47·6–60·6)
Serbia	48·8 (42·1–55·5)	57·9 (47·5–70·0)	52·3 (42·3–66·5)	61·7 (48·8–79·9)	49·7 (43·6–55·9)	58·8 (49·5–70·0)
Slovakia	46·0 (40·8–52·0)	51·7 (42·6–61·4)	50·5 (41·8–59·8)	56·9 (45·1–75·6)	46·7 (41·7–51·2)	52·5 (44·0–61·6)
Slovenia	47·5 (44·9–50·0)	59·8 (52·1–67·8)	54·0 (49·9–58·3)	64·1 (53·1–74·8)	49·1 (46·8–51·5)	60·6 (52·8–68·4)
Eastern Europe	44·0 (43·5–44·6)	47·3 (42·8–52·1)	54·1 (52·6–55·1)	55·5 (50·0–61·9)	46·2 (45·6–46·8)	48·9 (44·8–52·8)
Belarus	44·7 (42·9–46·4)	49·6 (40·2–59·2)	52·8 (49·8–55·9)	56·6 (45·8–68·4)	46·5 (44·8–48·3)	50·9 (41·7–61·0)
Estonia	44·9 (42·7–47·2)	53·7 (47·3–60·8)	55·0 (51·6–58·7)	65·0 (55·9–73·1)	47·5 (45·5–49·7)	56·0 (49·4–62·9)
Latvia	46·1 (43·8–48·3)	52·7 (46·2–59·9)	55·3 (52·0–58·4)	63·8 (55·7–71·3)	48·3 (46·1–50·3)	54·6 (48·1–61·4)
Lithuania	45·8 (43·9–47·8)	52·0 (46·1–57·9)	52·6 (49·6–55·5)	63·9 (55·8–71·9)	47·3 (45·6–49·1)	54·1 (48·0–60·0)
Republic of Moldova	44·1 (41·8–46·4)	49·2 (43·2–56·2)	49·3 (47·2–51·7)	55·8 (49·6–62·6)	45·3 (43·5–47·4)	50·2 (44·6–56·8)
Russian Federation	43·3 (42·7–43·9)	47·0 (42·5–51·0)	53·8 (52·2–54·9)	55·1 (49·6–60·5)	45·5 (44·8–46·1)	48·5 (44·8–52·1)

Ukraine	46·7 (44·7–48·5)	47·3 (31·9–65·1)	56·1 (53·7–58·5)	55·9 (37·9–79·6)	48·8 (46·9–50·6)	48·8 (35·3–64·2)
**High-income**	**46·9 (45·9–49·4)**	**51·3 (48·5–53·0)**	**51·9 (49·2–55·3)**	**52·3 (47·8–54·9)**	**48·3 (46·9–50·9)**	**51·6 (48·6–53·3)**
Australasia	39·9 (38·7–41·1)	45·3 (42·7–47·8)	43·3 (41·5–45·0)	45·7 (42·8–48·2)	40·6 (39·4–41·8)	45·4 (42·8–47·7)
Australia	40·2 (39·1–41·5)	45·5 (42·8–48·1)	43·9 (41·9–45·9)	46·4 (43·2–48·9)	41·0 (39·8–42·2)	45·7 (43·0–48·1)
New Zealand	38·0 (36·4–39·7)	44·2 (41·5–47·0)	40·3 (38·0–42·6)	42·2 (39·3–44·7)	38·5 (36·9–40·1)	43·7 (41·0–46·3)
High-income Asia Pacific	48·2 (46·4–61·4)	54·6 (45·1–57·4)	53·8 (49·9–64·3)	55·8 (46·2–61·0)	50·3 (47·8–60·9)	54·9 (46·1–58·0)
Brunei Darussalam	35·4 (27·7–42·7)	41·6 (32·3–48·8)	38·1 (27·7–48·6)	46·3 (33·4–58·7)	36·0 (28·8–42·7)	42·5 (33·7–49·4)
Japan	50·5 (49·4–51·4)	54·4 (52·0–56·3)	57·0 (52·9–59·1)	56·7 (49·4–60·9)	53·0 (50·6–54·2)	55·1 (51·4–57·7)
Republic of Korea	39·5 (33·6–105)	55·2 (30·0–61·6)	40·3 (33·3–97·8)	54·3 (30·5–63·4)	39·7 (33·8–97·4)	54·9 (31·2–60·6)
Singapore	41·3 (39·1–43·5)	49·2 (45·9–52·5)	43·5 (41·4–45·8)	52·0 (47·1–56·8)	42·2 (40·4–44·0)	50·1 (46·6–53·3)
High-income North America	43·7 (42·8–44·4)	47·8 (46·0–49·3)	45·0 (43·6–46·0)	46·9 (44·9–48·7)	44·0 (43·0–44·7)	47·6 (45·8–49·1)
Canada	40·7 (39·5–42·0)	46·0 (43·4–48·8)	43·1 (41·5–44·6)	45·2 (42·7–47·6)	41·2 (40·1–42·3)	45·8 (43·4–48·4)
Greenland	36·3 (29·1–42·5)	43·4 (36·0–54·4)	34·3 (28·4–40·3)	41·5 (32·5–51·7)	35·8 (30·1–40·8)	43·0 (35·7–52·4)
United States of America	44·1 (43·2–44·8)	47·9 (46·1–49·6)	45·3 (43·8–46·4)	47·1 (44·9–48·9)	44·3 (43·3–45·1)	47·8 (45·9–49·3)
Southern Latin America	44·9 (43·4–46·5)	43·9 (41·6–46·2)	44·6 (43·1–46·0)	43·2 (40·7–45·9)	44·9 (43·4–46·3)	43·8 (41·5–45·9)
Argentina	48·9 (46·8–51·1)	42·5 (39·5–45·5)	46·3 (44·3–48·2)	42·1 (39·1–45·4)	48·3 (46·4–50·2)	42·4 (39·7–45·2)
Chile	40·6 (38·5–42·7)	45·6 (42·9–48·1)	41·1 (39·3–42·8)	44·2 (41·5–46·9)	40·7 (38·8–42·6)	45·3 (42·9–47·6)
Uruguay	49·4 (46·8–52·0)	48·0 (44·6–51·4)	46·5 (43·7–49·3)	48·6 (44·7–52·3)	48·7 (46·4–51·1)	48·1 (44·9–51·4)
Western Europe	49·2 (48·0–50·1)	55·2 (52·0–58·1)	54·5 (51·5–56·1)	56·7 (51·5–59·7)	50·8 (49·3–51·8)	55·6 (52·1–58·2)
Andorra	45·9 (32·6–61·3)	52·7 (35·2–71·1)	46·6 (32·0–67·1)	53·9 (35·5–74·7)	46·0 (32·7–61·3)	53·0 (35·5–71·4)

Austria	49·2 (47·5–50·8)	56·9 (52·7–60·5)	55·6 (52·3–58·6)	58·4 (52·3–63·3)	51·1 (49·2–52·7)	57·2 (53·0–60·7)
Belgium	50·2 (48·3–52·0)	53·4 (49·5–57·1)	53·4 (50·5–55·5)	55·8 (50·5–59·6)	51·2 (49·3–52·8)	54·1 (50·0–57·4)
Cyprus	49·7 (43·6–56·5)	52·0 (43·3–61·4)	53·5 (40·1–65·2)	56·2 (44·6–70·9)	50·7 (44·1–56·4)	52·9 (45·2–61·5)
Denmark	50·1 (48·3–51·8)	58·2 (53·9–62·6)	55·3 (52·2–58·1)	59·4 (53·3–64·5)	51·9 (50·1–53·8)	58·5 (53·9–62·5)
Finland	44·1 (42·5–45·6)	53·6 (48·8–58·3)	50·7 (48·1–53·0)	55·4 (50·1–60·2)	45·6 (44·1–47·0)	54·0 (49·5–58·1)
France	50·7 (48·6–52·6)	56·3 (50·8–62·5)	55·4 (52·0–57·9)	58·4 (53·0–63·0)	52·0 (49·9–53·7)	56·8 (51·6–62·3)
Germany	50·2 (48·4–52·1)	58·1 (54·1–61·8)	57·6 (54·2–59·9)	60·0 (53·5–64·7)	52·5 (50·4–54·2)	58·5 (54·1–62·1)
Greece	49·6 (47·0–52·4)	56·8 (51·8–61·7)	53·5 (50·2–56·0)	57·4 (51·8–62·4)	50·6 (48·3–52·8)	56·9 (52·1–61·5)
Iceland	44·1 (41·3–47·1)	48·9 (44·2–54·1)	50·9 (47·3–54·9)	56·1 (49·2–62·5)	45·9 (43·3–48·7)	50·4 (45·4–55·6)
Ireland	45·7 (43·7–47·7)	50·4 (45·3–56·2)	50·4 (47·6–53·1)	52·4 (47·7–56·6)	46·8 (45·1–48·8)	50·8 (46·3–55·9)
Israel	46·6 (44·5–48·6)	50·6 (47·1–54·0)	50·5 (47·8–53·1)	53·4 (48·1–58·2)	47·7 (45·7–49·6)	51·2 (47·4–54·4)
Italy	52·8 (51·0–54·2)	58·1 (54·3–61·3)	54·7 (51·5–56·6)	58·8 (52·2–62·9)	53·3 (51·2–54·7)	58·3 (54·2–61·5)
Luxembourg	46·6 (43·8–49·5)	56·4 (50·3–62·7)	52·7 (49·1–56·2)	56·4 (50·1–62·2)	48·4 (46·1–50·9)	56·4 (50·6–62·4)
Malta	47·6 (44·3–50·9)	54·6 (48·4–60·7)	51·0 (47·2–54·9)	58·0 (50·5–65·2)	48·4 (45·4–51·3)	55·2 (49·1–61·2)
Monaco	52·4 (41·2–63·5)	54·9 (43·8–67·8)	55·8 (38·2–72·6)	57·5 (40·9–78·7)	53·8 (41·9–63·4)	55·8 (44·3–69·0)
Netherlands	45·9 (44·3–47·4)	52·9 (49·2–56·1)	49·9 (47·2–52·0)	54·7 (50·1–58·7)	47·4 (45·7–48·9)	53·5 (49·9–56·7)
Norway	44·2 (42·7–45·7)	48·5 (46·0–51·1)	47·9 (46·0–50·0)	48·6 (45·7–51·5)	45·2 (43·7–46·8)	48·6 (46·1–51·0)
Portugal	49·6 (47·3–51·8)	60·2 (56·0–64·7)	52·2 (49·7–54·7)	61·3 (54·5–66·1)	50·3 (48·2–52·2)	60·5 (56·0–64·7)
San Marino	50·4 (41·3–59·3)	56·5 (37·1–80·3)	53·7 (40·1–69·2)	57·4 (35·1–85·3)	51·1 (43·1–59·3)	56·7 (37·8–78·6)
Spain	50·8 (49·0–52·7)	56·3 (51·7–60·7)	55·0 (51·2–57·4)	57·4 (51·6–62·2)	51·9 (49·7–53·8)	56·6 (52·2–60·9)

Sweden	49·2 (47·5–50·9)	52·0 (45·2–59·8)	51·0 (48·4–53·2)	52·2 (45·1–59·4)	49·8 (48·2–51·4)	52·0 (46·6–58·7)
Switzerland	48·2 (46·4–49·9)	57·3 (52·7–61·5)	52·6 (49·3–55·3)	56·8 (50·7–61·8)	49·6 (47·6–51·2)	57·2 (52·3–61·0)
United Kingdom	43·1 (42·5–43·7)	47·1 (45·9–48·3)	49·8 (48·2–50·8)	49·3 (47·1–50·9)	44·9 (44·1–45·5)	47·7 (46·4–48·8)
**Latin America and Caribbean**	**39·1 (38·1–39·9)**	**41·8 (39·0–44·7)**	**35·7 (34·4–36·9)**	**39·3 (36·6–41·9)**	**38·3 (37·3–39·1)**	**41·3 (38·7–44·1)**
Andean Latin America	36·2 (31·9–40·5)	40·2 (31·9–48·2)	29·1 (25·1–33·8)	34·8 (26·9–42·5)	33·7 (30·1–37·4)	38·9 (31·1–46·1)
Bolivia (Plurinational State of)	38·2 (29·4–47·8)	41·9 (27·4–55·9)	29·7 (20·5–40·8)	35·7 (23·2–51·7)	34·8 (27·9–42·3)	40·0 (27·2–54·7)
Ecuador	34·6 (32·7–36·3)	39·1 (30·8–49·1)	27·8 (26·2–29·5)	33·7 (26·6–41·7)	32·2 (30·7–33·5)	38·0 (29·9–47·5)
Peru	36·3 (28·7–43·8)	41·0 (28·3–52·2)	29·7 (23·5–37·8)	35·6 (21·7–48·2)	34·2 (28·0–40·4)	39·7 (27·8–50·8)
Caribbean	45·7 (43·1–48·1)	50·9 (44·5–57·2)	39·2 (36·5–41·4)	47·5 (41·4–54·9)	43·6 (41·1–45·5)	50·2 (44·3–55·9)
Antigua and Barbuda	48·9 (44·5–53·0)	51·7 (47·4–56·0)	42·7 (38·5–46·9)	49·8 (45·3–54·0)	47·9 (44·1–51·6)	51·5 (47·4–55·5)
Bahamas	40·6 (36·4–44·7)	44·4 (34·9–55·4)	38·1 (34·4–41·6)	42·9 (33·8–54·1)	40·0 (36·5–43·8)	44·3 (34·6–55·3)
Barbados	47·7 (44·7–51·5)	54·8 (41·8–68·4)	45·3 (41·5–49·4)	52·9 (41·3–65·2)	47·2 (44·5–50·6)	54·5 (41·9–68·0)
Belize	42·2 (38·8–46·5)	42·0 (36·2–48·5)	35·9 (32·8–39·0)	39·6 (34·7–45·0)	40·7 (37·8–44·0)	41·7 (36·2–47·7)
Bermuda	48·6 (45·1–52·6)	59·9 (51·4–73·1)	47·9 (43·3–52·9)	58·1 (48·1–72·4)	48·4 (45·2–51·8)	59·6 (51·1–73·4)
Cuba	48·3 (46·7–49·8)	58·3 (49·7–66·7)	40·6 (39·2–41·9)	58·8 (50·1–67·4)	45·3 (44·0–46·4)	58·4 (50·0–66·2)
Dominica	48·5 (42·0–54·5)	50·2 (39·2–62·0)	46·0 (34·0–53·2)	47·4 (36·8–64·7)	48·0 (42·1–53·2)	49·9 (39·4–61·2)
Dominican Republic	40·1 (33·8–50·8)	44·4 (34·6–57·7)	34·5 (27·7–42·0)	42·2 (30·7–57·3)	38·7 (33·3–46·1)	44·0 (35·1–56·1)
Grenada	44·9 (40·3–50·0)	49·0 (41·7–56·4)	40·9 (36·8–45·0)	46·4 (39·9–53·3)	44·0 (39·9–48·6)	48·5 (41·7–55·7)
Guyana	39·5 (34·3–44·5)	41·8 (31·8–53·8)	36·5 (31·5–42·0)	39·7 (29·5–51·2)	38·7 (34·0–43·0)	41·5 (31·6–53·4)
Haiti	44·0 (25·1–58·0)	43·4 (28·5–59·5)	34·0 (14·1–45·5)	35·1 (17·0–49·7)	40·5 (21·6–52·2)	40·9 (26·8–55·0)

Jamaica	50·2 (42·8–59·9)	50·5 (37·4–65·4)	43·5 (39·5–48·1)	46·0 (34·8–60·3)	48·8 (42·7–56·8)	49·8 (37·3–64·2)
Puerto Rico	46·5 (44·2–48·7)	54·7 (44·8–64·6)	45·1 (42·1–48·2)	55·1 (45·5–64·9)	46·3 (44·2–48·4)	54·8 (45·0–64·4)
Saint Kitts and Nevis	49·2 (44·8–53·3)	50·4 (41·0–60·7)	43·2 (39·0–47·7)	50·0 (40·5–60·3)	47·3 (43·9–50·5)	50·4 (41·4–60·5)
Saint Lucia	44·2 (41·2–48·0)	48·9 (40·0–59·3)	40·2 (37·0–43·6)	48·5 (39·3–58·6)	43·4 (40·9–46·7)	48·9 (40·1–59·2)
Saint Vincent and the Grenadines	43·3 (39·8–47·3)	50·4 (44·0–57·9)	37·9 (34·0–42·0)	44·5 (38·2–51·5)	42·5 (39·2–45·9)	50·0 (43·5–57·5)
Suriname	40·8 (35·0–45·7)	45·6 (35·0–57·3)	37·3 (27·9–42·6)	41·7 (32·0–57·7)	39·7 (34·1–43·5)	44·7 (35·0–55·3)
Trinidad and Tobago	39·9 (37·6–42·0)	47·8 (36·2–61·8)	39·0 (36·4–42·0)	46·8 (35·7–59·8)	39·7 (37·7–41·7)	47·7 (36·2–61·4)
United States Virgin Islands	43·3 (35·6–51·5)	47·8 (36·9–60·9)	42·6 (31·4–51·9)	53·8 (38·2–84·2)	43·1 (35·6–50·5)	48·5 (38·0–60·9)
Central Latin America	35·8 (34·9–36·7)	39·0 (34·9–43·6)	29·8 (28·9–31·0)	34·6 (30·3–38·7)	34·7 (33·9–35·5)	38·2 (34·4–42·5)
Colombia	34·7 (33·1–36·2)	40·6 (33·6–47·9)	29·7 (27·7–31·7)	34·8 (29·1–40·5)	33·7 (32·2–35·1)	39·5 (32·7–46·2)
Costa Rica	38·1 (35·8–40·5)	43·6 (38·4–49·0)	31·6 (29·4–34·1)	38·3 (33·7–42·5)	37·2 (35·1–39·7)	42·8 (37·8–47·9)
El Salvador	32·9 (30·0–36·7)	40·0 (31·1–48·9)	26·5 (23·2–31·8)	35·4 (25·5–44·8)	31·1 (28·8–34·7)	38·8 (31·3–48·1)
Guatemala	36·0 (34·3–37·8)	36·3 (30·8–41·8)	29·8 (28·0–31·6)	29·8 (25·2–34·6)	34·9 (33·4–36·5)	34·7 (29·5–39·9)
Honduras	36·1 (29·2–44·9)	44·4 (33·4–58·3)	27·3 (21·3–33·7)	35·8 (23·1–54·1)	34·2 (28·5–41·1)	42·7 (32·2–56·7)
Mexico	36·4 (35·4–37·3)	37·6 (32·4–43·3)	31·2 (30·2–32·2)	34·1 (28·9–39·8)	35·6 (34·7–36·5)	37·0 (32·7–41·9)
Nicaragua	34·4 (30·7–44·7)	38·7 (31·3–47·5)	26·1 (22·0–40·2)	32·9 (24·6–42·7)	31·9 (29·0–42·4)	37·4 (31·1–45·1)
Panama	36·4 (33·9–39·2)	43·2 (34·4–51·9)	28·6 (26·3–31·0)	35·2 (28·6–42·4)	35·3 (33·0–37·9)	41·9 (33·5–50·3)
Venezuela (Bolivarian Republic of)	36·6 (34·9–38·1)	40·8 (30·6–52·2)	30·6 (29·0–32·5)	38·4 (29·4–49·7)	35·5 (34·2–36·8)	40·4 (30·5–51·7)
Tropical Latin America	39·4 (38·3–40·6)	42·5 (40·8–44·4)	38·6 (37·5–39·7)	42·1 (39·8–43·9)	39·2 (38·2–40·3)	42·4 (40·8–44·0)
Brazil	39·4 (38·2–40·6)	42·6 (40·8–44·4)	38·7 (37·6–39·8)	42·2 (40·0–44·1)	39·2 (38·2–40·3)	42·5 (40·8–44·0)

Paraguay	40·5 (33·4–50·1)	41·0 (31·0–53·1)	34·3 (27·6–44·4)	37·6 (27·2–50·4)	38·4 (33·0–45·8)	40·2 (31·1–51·3)
**North Africa and Middle East**	**34·9 (28·4–39·5)**	**38·4 (31·6–44·5)**	**30·8 (21·0–35·5)**	**36·1 (27·2–43·1)**	**33·4 (26·8–37·5)**	**37·8 (30·7–43·3)**
North Africa and Middle East	34·9 (28·4–39·5)	38·4 (31·6–44·5)	30·8 (21·0–35·5)	36·1 (27·2–43·1)	33·4 (26·8–37·5)	37·8 (30·7–43·3)
Afghanistan	44·5 (30·9–59·6)	34·9 (25·4–49·7)	41·4 (30·1–56·5)	36·8 (26·0–56·8)	43·2 (31·7–55·7)	35·7 (26·3–51·4)
Algeria	31·9 (24·8–38·8)	38·5 (27·0–49·9)	29·4 (13·3–40·4)	36·4 (20·5–49·2)	30·9 (20·5–37·9)	37·8 (26·2–48·0)
Bahrain	35·7 (30·9–41·8)	37·0 (30·7–45·3)	31·2 (24·6–36·9)	36·5 (28·9–56·0)	34·6 (30·9–39·3)	36·9 (30·7–45·5)
Egypt	35·3 (27·2–41·1)	37·3 (29·3–45·9)	33·0 (20·5–40·7)	36·9 (27·6–48·0)	34·6 (25·8–39·0)	37·2 (29·9–45·5)
Iran (Islamic Republic of)	31·9 (27·4–36·1)	37·5 (32·1–41·3)	27·8 (19·4–31·5)	35·8 (31·0–48·1)	30·2 (25·6–33·3)	37·1 (33·5–41·4)
Iraq	36·6 (28·3–44·6)	40·0 (29·0–53·9)	31·2 (21·8–38·8)	35·5 (25·2–50·5)	35·1 (27·9–42·2)	38·9 (28·7–52·2)
Jordan	32·8 (26·1–39·0)	36·5 (29·1–45·1)	30·5 (18·2–39·6)	35·7 (26·2–49·3)	31·9 (24·7–37·7)	36·3 (28·8–45·6)
Kuwait	34·5 (30·5–38·2)	39·5 (32·3–48·3)	32·0 (29·1–36·8)	36·6 (31·8–42·2)	33·8 (30·9–36·7)	39·0 (32·4–47·0)
Lebanon	38·5 (19·7–50·9)	42·0 (34·4–50·7)	34·2 (15·6–47·8)	41·0 (30·5–54·0)	36·5 (18·8–47·1)	41·6 (34·4–50·1)
Libya	33·3 (23·0–42·4)	37·6 (25·6–51·7)	30·0 (15·1–40·2)	36·0 (19·1–57·6)	32·1 (22·0–40·6)	37·2 (24·2–52·0)
Morocco	35·3 (26·7–45·8)	42·3 (28·1–61·0)	30·5 (13·3–41·0)	38·7 (20·0–56·7)	32·9 (21·6–41·6)	40·8 (26·1–56·6)
Oman	35·0 (25·2–47·8)	37·0 (28·0–47·5)	32·7 (20·2–45·7)	35·1 (25·2–47·9)	34·6 (25·4–46·6)	36·8 (28·1–46·6)
Palestine	34·2 (24·5–45·9)	35·1 (26·1–41·8)	33·1 (23·3–47·5)	34·6 (27·8–45·1)	33·9 (24·4–45·4)	35·0 (27·9–41·7)
Qatar	34·8 (27·8–44·5)	36·0 (26·5–47·7)	32·5 (22·9–42·2)	35·5 (25·4–54·7)	34·5 (28·0–42·8)	35·9 (26·5–47·3)
Saudi Arabia	37·3 (27·2–49·8)	38·5 (28·0–51·3)	31·8 (20·0–41·9)	36·8 (26·2–50·4)	35·7 (25·7–46·2)	38·2 (28·2–50·3)
Sudan	34·6 (22·2–46·8)	33·7 (20·8–48·2)	28·5 (10·5–42·4)	29·3 (12·5–49·6)	31·8 (18·5–42·1)	32·1 (19·0–46·5)
Syrian Arab Republic	33·1 (24·7–42·2)	41·8 (30·3–57·6)	30·2 (18·9–38·9)	36·9 (25·9–54·4)	32·3 (24·1–40·4)	40·7 (30·4–55·4)

Tunisia	35·9 (27·3–56·3)	42·1 (26·8–64·2)	32·7 (15·7–48·9)	41·3 (21·6–64·3)	34·6 (23·4–50·1)	41·9 (26·6–62·5)
Türkiye	34·8 (23·9–45·6)	40·9 (31·1–50·6)	35·1 (22·3–63·8)	40·4 (28·5–51·4)	34·9 (24·4–47·0)	40·8 (31·3–49·6)
United Arab Emirates	34·9 (25·2–52·4)	38·5 (28·8–53·2)	31·9 (22·9–43·0)	37·6 (28·2–54·9)	34·3 (25·2–48·2)	38·3 (29·3–51·7)
Yemen	37·5 (22·1–52·5)	37·1 (20·3–54·8)	33·7 (10·1–53·1)	33·5 (12·5–52·2)	36·1 (18·3–51·3)	35·9 (17·8–52·9)
**South Asia**	**36·2 (27·6–41·2)**	**40·1 (34·4–46·0)**	**30·2 (20·1–35·6)**	**36·1 (26·6–42·2)**	**33·6 (25·6–37·6)**	**38·6 (31·8–43·1)**
South Asia	36·2 (27·6–41·2)	40·1 (34·4–46·0)	30·2 (20·1–35·6)	36·1 (26·6–42·2)	33·6 (25·6–37·6)	38·6 (31·8–43·1)
Bangladesh	30·5 (23·3–36·2)	38·8 (29·8–50·0)	25·8 (20·7–33·5)	32·7 (22·9–68·0)	28·8 (23·4–33·8)	36·9 (28·3–51·3)
Bhutan	37·6 (23·9–49·6)	45·5 (32·9–60·4)	37·3 (23·6–53·5)	46·5 (32·5–66·8)	37·5 (25·0–49·0)	45·8 (34·5–60·4)
India	36·6 (27·2–42·1)	40·6 (33·1–47·0)	30·4 (19·4–36·1)	36·5 (25·0–43·0)	33·8 (25·3–38·1)	39·0 (30·8–43·8)
Nepal	37·5 (23·3–49·2)	41·6 (32·1–53·5)	29·9 (17·5–41·2)	35·4 (19·4–52·1)	35·1 (22·8–44·8)	39·6 (30·0–51·5)
Pakistan	34·4 (23·5–47·9)	34·8 (21·5–57·0)	31·4 (16·9–61·0)	30·8 (18·8–61·4)	33·7 (23·8–45·2)	33·8 (23·1–50·8)
**Southeast Asia, East Asia, and Oceania**	**44·4 (31·5–51·8)**	**54·5 (44·7–67·2)**	**44·2 (26·3–53·6)**	**58·6 (46·1–76·7)**	**44·3 (31·0–50·1)**	**56·0 (47·8–66·7)**
East Asia	45·6 (30·6–54·2)	57·8 (45·6–73·6)	44·7 (25·9–54·6)	60·3 (46·2–80·8)	45·1 (30·9–51·7)	58·8 (48·8–71·9)
China	45·6 (30·1–54·4)	58·1 (45·3–74·7)	44·6 (25·6–54·7)	60·6 (45·7–81·2)	45·1 (30·4–51·8)	59·1 (48·7–72·7)
Democratic People's Republic of Korea	44·2 (31·1–60·7)	49·1 (34·8–78·8)	46·4 (31·3–72·9)	53·5 (33·9–104)	45·3 (32·2–62·0)	51·0 (37·2–82·5)
Taiwan (Province of China)	47·9 (46·2–49·7)	54·7 (50·2–58·7)	48·0 (46·0–49·9)	54·5 (49·6–59·0)	47·9 (46·5–49·4)	54·6 (50·3–58·1)
Oceania	33·5 (27·3–39·6)	35·8 (30·4–41·9)	31·9 (25·5–40·1)	34·3 (27·4–48·0)	33·0 (27·3–38·9)	35·3 (30·2–42·0)
American Samoa	32·1 (25·3–39·6)	36·9 (28·1–45·8)	30·6 (23·8–40·4)	38·0 (28·2–48·4)	31·8 (26·3–38·9)	37·2 (28·7–45·9)
Cook Islands	35·6 (27·9–44·4)	43·8 (35·4–54·4)	33·6 (24·7–43·8)	45·5 (33·7–61·3)	35·0 (27·9–43·3)	44·2 (35·2–54·4)
Fiji	32·6 (26·1–39·6)	36·7 (26·5–47·5)	29·3 (21·9–37·9)	35·3 (25·2–47·5)	31·5 (26·5–36·9)	36·2 (26·9–47·0)

Guam	31·6 (28·2–37·2)	37·0 (32·0–41·7)	34·1 (28·6–44·5)	45·9 (35·1–54·6)	32·0 (29·0–37·1)	38·2 (33·6–42·5)
Kiribati	30·7 (23·4–37·7)	31·9 (22·6–41·4)	29·3 (22·0–37·5)	30·8 (21·7–42·3)	30·4 (24·1–37·1)	31·7 (23·0–41·0)
Marshall Islands	29·6 (16·1–39·5)	33·5 (19·6–46·2)	29·2 (16·1–36·8)	31·5 (15·3–43·8)	29·5 (18·0–37·5)	33·0 (19·6–44·0)
Micronesia (Federated States of)	31·7 (19·8–40·1)	33·9 (22·5–44·7)	30·1 (18·2–40·9)	33·7 (22·2–46·4)	31·3 (20·5–39·7)	33·8 (22·9–44·5)
Nauru	32·9 (17·3–43·4)	32·0 (16·7–42·5)	30·1 (15·2–42·1)	31·2 (16·6–45·1)	32·2 (17·5–41·7)	31·8 (17·2–42·3)
Niue	36·1 (25·7–47·5)	38·0 (27·3–50·2)	41·5 (28·1–56·0)	33·3 (25·7–42·7)	37·5 (28·0–48·1)	36·5 (28·4–46·1)
Northern Mariana Islands	33·9 (23·0–46·9)	41·3 (34·5–48·3)	31·3 (21·7–46·8)	42·9 (30·4–51·4)	33·5 (23·4–46·5)	41·5 (34·5–47·6)
Palau	33·3 (19·2–56·7)	38·6 (30·0–49·0)	31·8 (20·6–48·7)	34·5 (25·6–46·0)	32·8 (21·1–51·0)	37·1 (29·2–46·9)
Papua New Guinea	38·6 (27·1–53·0)	38·6 (29·3–56·0)	35·6 (24·0–53·3)	34·7 (23·9–62·0)	37·5 (27·3–49·8)	37·2 (28·2–55·2)
Samoa	31·5 (22·9–41·0)	34·5 (24·9–44·6)	31·6 (21·6–42·8)	34·7 (23·8–47·0)	31·5 (23·5–40·2)	34·6 (25·8–44·2)
Solomon Islands	31·6 (14·6–43·9)	32·6 (21·9–43·1)	29·6 (10·8–45·0)	31·4 (18·7–46·8)	31·1 (14·0–43·2)	32·3 (21·6–43·6)
Tokelau	35·7 (24·5–47·9)	33·9 (26·9–44·6)	35·6 (22·2–50·4)	30·4 (20·9–40·3)	35·7 (25·5–47·6)	32·6 (25·5–40·9)
Tonga	35·6 (28·7–42·2)	36·4 (27·3–48·6)	35·0 (27·4–45·7)	38·7 (27·9–53·2)	35·3 (29·1–42·5)	37·2 (28·4–49·2)
Tuvalu	33·8 (21·9–43·9)	34·5 (25·1–44·6)	34·6 (20·0–45·3)	36·4 (24·6–51·4)	34·1 (23·0–43·1)	35·0 (26·6–43·9)
Vanuatu	32·1 (19·9–42·1)	32·7 (22·9–40·9)	29·8 (17·2–43·2)	31·6 (21·5–44·3)	31·6 (20·0–41·1)	32·5 (23·4–41·2)
Southeast Asia	38·1 (33·5–42·5)	44·1 (37·3–51·8)	37·4 (28·6–42·6)	47·3 (38·0–58·4)	37·9 (33·1–41·7)	44·9 (38·0–51·7)
Cambodia	38·5 (30·2–47·8)	41·7 (30·4–55·2)	35·7 (24·0–46·5)	43·7 (28·9–61·2)	37·4 (29·9–44·9)	42·3 (31·0–55·8)
Indonesia	37·5 (30·6–43·7)	42·6 (32·8–56·4)	38·0 (27·1–46·5)	48·0 (34·7–63·7)	37·7 (30·6–42·7)	44·3 (36·2–54·4)
Lao People's Democratic Republic	36·5 (24·2–49·2)	37·9 (27·3–54·0)	32·8 (13·7–46·8)	34·2 (22·3–49·0)	35·4 (22·1–46·4)	36·9 (27·1–50·1)
Malaysia	40·9 (36·3–46·2)	45·5 (40·6–51·9)	40·6 (29·6–48·7)	47·5 (40·4–61·7)	40·8 (36·3–45·1)	45·9 (41·5–51·6)

Maldives	38·3 (22·1–49·6)	39·4 (30·0–50·4)	32·9 (11·8–46·6)	42·1 (31·7–55·4)	37·2 (20·9–46·2)	39·6 (30·8–50·3)
Mauritius	40·3 (38·2–42·5)	45·5 (41·7–48·3)	36·4 (34·2–39·0)	47·0 (42·7–50·1)	39·0 (37·3–40·7)	45·8 (42·3–48·0)
Myanmar	37·4 (19·5–50·4)	41·4 (26·9–53·7)	34·7 (17·9–48·4)	42·6 (29·9–57·8)	36·7 (21·3–48·8)	41·6 (27·6–53·4)
Philippines	32·6 (28·6–36·9)	37·4 (29·0–46·4)	30·9 (25·5–36·7)	37·0 (28·1–48·7)	32·3 (28·8–35·8)	37·4 (30·6–44·6)
Seychelles	42·3 (37·3–47·7)	47·5 (39·5–54·7)	38·1 (29·6–44·9)	45·5 (37·0–56·6)	41·8 (37·3–46·5)	47·3 (40·1–54·0)
Sri Lanka	39·4 (34·8–44·0)	46·8 (30·1–66·7)	32·6 (25·8–37·6)	42·5 (28·0–66·1)	37·6 (33·7–41·3)	45·9 (29·9–64·4)
Thailand	37·0 (30·0–45·6)	45·7 (34·2–58·3)	38·2 (28·4–49·7)	50·4 (37·0–66·8)	37·3 (31·8–44·8)	46·6 (35·6–58·6)
Timor-Leste	34·0 (23·5–45·8)	37·8 (26·7–54·6)	31·6 (16·7–44·3)	34·8 (24·3–48·8)	33·2 (23·7–43·4)	36·9 (27·2–50·5)
Viet Nam	41·5 (30·1–52·8)	47·0 (35·0–59·1)	42·3 (29·7–54·6)	51·3 (36·2–66·7)	41·8 (31·7–52·5)	48·4 (36·0–61·3)
**Sub-Saharan Africa**	**46·2 (40·0–52·3)**	**44·8 (38·6–52·1)**	**44·0 (35·6–50·4)**	**45·6 (34·9–54·9)**	**45·7 (40·3–50·5)**	**45·0 (39·3–51·8)**
Central Sub-Saharan Africa	47·3 (37·0–58·4)	45·6 (34·6–61·3)	45·8 (33·0–57·7)	46·3 (32·0–65·8)	47·0 (37·8–56·9)	45·8 (34·8–61·3)
Angola	46·8 (34·2–60·6)	45·6 (34·3–58·8)	44·1 (31·2–58·7)	44·9 (29·8–62·8)	46·3 (35·3–59·3)	45·4 (33·7–57·9)
Central African Republic	46·3 (34·8–61·7)	44·7 (31·5–61·7)	46·3 (29·7–60·6)	44·5 (27·0–63·8)	46·3 (36·0–58·1)	44·6 (31·2–60·3)
Congo	46·4 (36·7–58·1)	46·5 (34·1–60·9)	48·9 (32·1–64·1)	46·7 (30·0–63·3)	47·0 (37·1–57·9)	46·5 (35·2–60·4)
Democratic Republic of the Congo	47·5 (35·9–61·8)	45·7 (32·8–67·4)	45·8 (32·6–61·3)	46·7 (31·0–73·9)	47·1 (37·3–59·5)	45·9 (32·8–65·6)
Equatorial Guinea	48·2 (34·8–62·0)	40·7 (28·8–58·4)	47·4 (31·2–63·9)	44·9 (24·5–78·0)	48·0 (35·7–60·7)	41·5 (28·2–60·8)
Gabon	50·9 (39·4–65·5)	48·5 (35·1–63·7)	52·6 (36·3–66·1)	50·9 (32·9–80·4)	51·2 (40·8–63·8)	48·9 (35·7–65·9)
Eastern Sub-Saharan Africa	47·9 (40·7–55·2)	45·8 (39·5–55·5)	44·8 (33·7–54·0)	45·5 (37·4–56·6)	47·1 (40·8–52·4)	45·8 (39·6–54·1)
Burundi	47·6 (31·9–76·7)	46·4 (34·2–70·0)	46·5 (29·4–66·0)	42·2 (28·5–64·6)	47·3 (33·2–68·7)	45·4 (34·6–67·2)
Comoros	48·0 (28·5–79·1)	50·4 (30·8–89·4)	44·2 (26·8–58·2)	49·6 (34·8–67·6)	46·6 (29·6–68·5)	50·1 (34·4–77·1)

Djibouti	43·9 (25·3–83·2)	47·3 (26·2–87·7)	44·7 (30·6–63·5)	49·1 (31·2–75·2)	44·1 (28·1–73·0)	47·8 (28·9–81·2)
Eritrea	42·4 (31·0–58·8)	42·7 (29·7–60·6)	46·0 (29·7–60·1)	47·8 (33·8–64·9)	43·4 (34·1–55·0)	44·0 (31·9–61·1)
Ethiopia	47·7 (32·6–62·4)	47·5 (38·0–58·2)	43·4 (23·2–58·6)	45·1 (34·8–59·6)	46·7 (32·0–56·4)	47·0 (39·0–56·0)
Kenya	49·1 (36·1–84·2)	48·2 (35·9–69·3)	47·4 (36·0–68·0)	49·6 (34·7–74·8)	48·6 (37·9–75·9)	48·5 (37·9–65·5)
Madagascar	47·7 (37·9–62·4)	44·1 (30·1–62·0)	43·8 (33·2–53·2)	41·6 (28·5–55·5)	46·6 (38·6–57·4)	43·3 (30·9–59·0)
Malawi	46·3 (35·3–57·7)	43·7 (33·4–55·1)	44·0 (32·8–56·3)	44·9 (30·5–67·8)	45·8 (37·2–54·7)	44·0 (34·6–55·2)
Mozambique	50·1 (39·3–63·0)	44·0 (31·4–58·1)	45·8 (35·6–57·1)	44·3 (28·4–62·5)	49·1 (40·0–59·5)	44·0 (31·3–57·5)
Rwanda	45·6 (34·6–58·9)	46·6 (32·9–66·0)	44·7 (28·0–61·2)	48·6 (30·7–73·4)	45·3 (34·4–56·0)	47·1 (33·8–65·8)
Somalia	44·8 (22·1–84·7)	42·4 (22·3–84·1)	44·2 (27·5–63·6)	44·1 (27·4–74·0)	44·6 (26·8–73·6)	43·0 (27·3–74·0)
South Sudan	52·0 (34·7–73·6)	47·7 (33·1–67·9)	46·0 (31·8–63·4)	43·2 (31·1–60·7)	50·8 (35·1–68·9)	46·6 (33·8–63·4)
Uganda	50·1 (36·8–62·4)	44·0 (33·2–55·2)	48·9 (33·3–69·5)	45·4 (31·8–61·8)	49·9 (38·5–61·5)	44·3 (33·3–55·5)
United Republic of Tanzania	47·7 (38·3–57·5)	47·0 (35·6–62·3)	46·1 (36·2–57·0)	47·1 (33·9–64·2)	47·4 (39·4–56·4)	47·1 (35·9–63·0)
Zambia	47·3 (39·1–56·5)	43·6 (32·3–56·5)	41·1 (31·2–51·4)	43·2 (28·1–62·9)	45·8 (38·5–54·3)	43·6 (32·3–57·6)
Southern Sub-Saharan Africa	37·0 (30·3–44·6)	38·4 (33·5–44·2)	36·6 (25·4–43·4)	43·0 (34·9–52·4)	36·9 (29·4–43·5)	39·3 (34·3–44·7)
Botswana	38·1 (26·2–53·3)	40·5 (28·4–57·2)	39·4 (25·3–68·0)	42·2 (28·5–73·9)	38·3 (27·2–53·2)	40·8 (29·2–56·1)
Eswatini	39·3 (29·8–51·7)	37·5 (25·9–50·2)	37·6 (28·1–52·8)	39·9 (22·1–64·1)	38·9 (29·9–49·2)	37·9 (26·0–50·7)
Lesotho	43·2 (30·6–69·5)	39·0 (28·0–52·1)	45·9 (22·5–89·4)	43·0 (22·2–66·2)	44·0 (31·7–72·2)	39·9 (28·0–54·0)
Namibia	40·2 (31·6–50·6)	40·4 (28·7–54·1)	40·0 (29·6–54·5)	43·0 (26·6–70·1)	40·2 (32·0–48·5)	40·8 (28·8–55·0)
South Africa	36·0 (29·4–42·9)	38·1 (33·5–43·0)	35·7 (26·1–41·6)	45·2 (37·8–61·9)	35·9 (29·1–41·4)	39·4 (35·4–45·6)
Zimbabwe	42·4 (31·0–58·8)	39·0 (26·2–51·3)	40·5 (17·0–54·0)	38·5 (17·8–53·5)	41·9 (27·2–56·4)	38·9 (24·8–51·1)

Western Sub-Saharan Africa	49·5 (38·1–59·7)	46·7 (37·8–55·3)	48·1 (38·5–60·0)	46·6 (31·6–59·5)	49·2 (39·2–57·7)	46·7 (37·2–54·4)
Benin	48·6 (39·6–59·1)	45·3 (32·9–66·2)	47·9 (36·2–59·3)	46·0 (26·3–66·5)	48·4 (40·6–58·1)	45·5 (32·7–65·3)
Burkina Faso	51·4 (40·2–62·6)	49·3 (38·3–65·1)	48·9 (37·6–62·7)	46·4 (32·9–65·8)	50·8 (41·2–61·0)	48·6 (38·8–63·2)
Cabo Verde	43·7 (35·7–52·6)	47·1 (35·7–59·8)	46·7 (32·9–60·3)	54·2 (41·3–70·6)	44·4 (36·6–52·0)	48·1 (37·3–60·4)
Cameroon	47·2 (38·5–58·9)	44·1 (28·4–62·9)	46·7 (33·5–57·4)	45·3 (24·4–66·3)	47·1 (39·2–57·1)	44·3 (29·3–63·4)
Chad	50·0 (33·1–82·4)	45·6 (29·9–81·9)	50·8 (35·2–69·7)	44·7 (27·4–60·7)	50·3 (36·2–73·1)	45·3 (30·5–73·0)
Côte d'Ivoire	45·6 (37·1–57·2)	46·2 (33·5–65·6)	42·4 (27·9–56·5)	43·9 (24·1–67·7)	45·2 (37·0–56·2)	45·8 (32·7–64·7)
Gambia	44·7 (26·9–77·5)	43·8 (27·0–71·8)	46·7 (34·2–63·4)	47·9 (33·8–65·1)	45·4 (30·4–70·2)	45·0 (29·9–67·0)
Ghana	44·7 (35·0–55·6)	46·1 (35·4–58·6)	41·5 (27·6–95·9)	47·1 (34·5–66·5)	44·2 (35·2–56·0)	46·3 (35·4–59·2)
Guinea	51·1 (32·6–82·3)	46·8 (29·1–75·0)	49·8 (34·3–65·7)	45·2 (27·3–62·9)	50·6 (36·8–71·3)	46·2 (30·4–69·7)
Guinea-Bissau	45·4 (26·8–65·2)	41·7 (29·4–57·1)	43·5 (27·0–59·9)	43·4 (28·1–56·9)	44·8 (28·9–61·0)	42·2 (30·9–55·7)
Liberia	48·3 (36·8–62·1)	43·8 (30·4–61·6)	48·6 (30·8–65·2)	44·4 (23·1–75·7)	48·4 (36·8–60·3)	44·0 (28·6–64·1)
Mali	49·1 (30·8–81·0)	46·7 (30·4–74·9)	45·8 (27·0–63·3)	42·9 (26·5–61·2)	47·7 (32·1–68·6)	45·3 (31·5–66·2)
Mauritania	47·8 (30·9–82·6)	49·4 (30·0–92·3)	49·9 (31·9–75·2)	49·3 (28·6–72·6)	48·5 (34·7–72·8)	49·3 (32·4–82·6)
Niger	46·0 (28·7–76·5)	46·0 (27·1–79·9)	43·7 (28·1–56·0)	44·9 (27·0–63·8)	45·2 (32·1–66·8)	45·6 (29·7–71·1)
Nigeria	51·1 (30·1–68·0)	47·4 (30·8–64·3)	50·5 (35·4–71·8)	48·5 (29·9–78·7)	51·0 (33·3–65·8)	47·6 (34·0–61·8)
Sao Tome and Principe	45·5 (34·1–56·6)	42·6 (30·6–58·3)	48·9 (27·9–62·5)	47·6 (27·4–67·7)	46·4 (32·7–56·3)	43·8 (32·1–59·8)
Senegal	49·3 (38·7–60·8)	48·9 (37·7–64·4)	45·5 (34·9–57·8)	49·6 (36·2–67·9)	48·4 (38·6–58·8)	49·1 (37·8–64·3)
Sierra Leone	50·1 (32·7–77·2)	45·6 (28·7–73·5)	49·3 (32·1–65·4)	45·2 (24·5–62·2)	49·9 (34·1–70·3)	45·4 (30·1–67·7)
Togo	44·6 (34·8–57·4)	45·3 (30·7–62·8)	43·4 (30·6–55·0)	47·7 (27·1–69·4)	44·3 (35·2–56·2)	45·9 (31·9–63·1)

Appendix Table S7: Number of deaths, incidence of suicide, and percent of suicide deaths using firearms, for males and females in 2021. Estimates provided at the global, super-region, regional, and national level.

	Male				Female			
Location	Deaths	Incidence	Incidence Ratio	Percent Firearm	Deaths	Incidence	Incidence Ratio	Percent Firearm
**Global**	**519000 (485000–556000)**	**2210000 (1860000–2590000)**	**4·27 (3·54–5·16)**	**9·70 (7·88–12·0)**	**227000 (200000–255000)**	**3270000 (2730000–3910000)**	**14·5 (11·3–18·1)**	**2·89 (2·13–3·70)**
Low SDI	52900 (45700–62800)	167000 (136000–201000)	3·19 (2·40–4·09)	7·28 (3·06–13·0)	20900 (17300–25500)	253000 (202000–317000)	12·2 (8·64–16·2)	4·10 (1·57–7·39)
Low-middle SDI	120000 (101000–133000)	409000 (329000–497000)	3·44 (2·71–4·47)	8·14 (4·17–13·7)	63900 (50800–73600)	874000 (699000–1080000)	13·8 (10·4–18·6)	1·82 (0·738–2·98)
Middle SDI	137000 (124000–153000)	524000 (432000–621000)	3·84 (3·10–4·73)	3·91 (2·57–5·58)	68800 (56200–81300)	840000 (693000–1010000)	12·3 (9·21–16·1)	0·876 (0·552–1·31)
High-middle SDI	95500 (87100–106000)	499000 (424000–577000)	5·23 (4·36–6·19)	4·94 (4·22–5·55)	36500 (31600–45200)	522000 (442000–612000)	14·4 (10·9–17·7)	0·774 (0·532–0·948)
High SDI	114000 (109000–117000)	613000 (528000–704000)	5·40 (4·63–6·26)	23·5 (23·0–24·1)	37000 (34800–38600)	785000 (678000–905000)	21·2 (18·2–24·7)	9·86 (9·35–10·4)
**Central Europe, Eastern Europe, and Central Asia**	**59500 (55300–63400)**	**301000 (257000–348000)**	**5·07 (4·27–5·98)**	**4·00 (3·70–4·31)**	**14100 (13000–15300)**	**211000 (182000–244000)**	**15·0 (12·6–17·8)**	**0·466 (0·402–0·566)**
Central Asia	7090 (6430–7770)	38100 (34800–41600)	5·39 (4·68–6·13)	2·52 (2·33–2·77)	2130 (1890–2390)	36700 (32700–41100)	17·2 (14·5–20·2)	0·434 (0·393–0·497)
Armenia	128 (114–145)	841 (762–924)	6·61 (5·66–7·74)	10·1 (9·91–10·3)	37·8 (33·0–44·0)	730 (630–840)	19·4 (15·7–23·6)	3·46 (3·27–3·68)
Azerbaijan	206 (150–286)	1810 (1530–2090)	9·01 (6·12–12·7)	2·14 (0·779–3·75)	56·6 (40·9–80·6)	2070 (1620–2600)	37·6 (24·8–53·8)	0·853 (0·398–1·46)
Georgia	252 (222–284)	920 (830–1020)	3·66 (3·14–4·28)	20·8 (19·6–22·2)	59·7 (52·3–67·4)	600 (509–694)	10·1 (8·21–12·4)	2·86 (2·55–3·25)
Kazakhstan	2850 (2520–3170)	14300 (13200–15500)	5·05 (4·33–5·83)	1·59 (1·55–1·64)	604 (526–681)	10000 (9110–11000)	16·7 (14·2–19·5)	0·300 (0·274–0·327)
Kyrgyzstan	498 (425–580)	2260 (2050–2490)	4·56 (3·83–5·43)	3·09 (2·72–3·53)	136 (115–159)	1900 (1640–2190)	14·1 (11·1–17·6)	0·414 (0·374–0·458)
Mongolia	423 (336–521)	1950 (1690–2230)	4·67 (3·48–5·99)	4·30 (1·78–7·30)	63·7 (48·6–81·7)	1210 (1040–1400)	19·4 (14·1–26·3)	1·42 (0·708–2·67)
Tajikistan	253 (176–373)	1600 (1380–1810)	6·56 (4·19–9·38)	0·235 (0·0717–0·441)	89·8 (60·7–145)	2110 (1800–2490)	24·7 (14·0–35·6)	0·0215 (0·00676–0·0411)
Turkmenistan	384 (304–494)	1680 (1480–1900)	4·45 (3·30–5·78)	2·40 (2·21–2·93)	117 (90·0–156)	1610 (1380–1860)	14·0 (10·1–18·2)	0·585 (0·471–0·988)

Uzbekistan	2100 (1810–2430)	12700 (11600–14000)	6·11 (5·04–7·33)	0·944 (0·894–0·995)	968 (827–1130)	16400 (14500–18500)	17·0 (13·8–20·6)	0·183 (0·158–0·212)
Central Europe	12800 (11900–13600)	58800 (52300–65500)	4·60 (4·01–5·28)	5·99 (4·74–7·43)	2880 (2610–3170)	41900 (36900–47300)	14·6 (12·4–17·0)	1·07 (0·786–1·52)
Albania	70·9 (53·2–94·1)	821 (703–949)	11·9 (8·73–16·4)	24·5 (11·7–37·6)	38·6 (28·1–51·4)	1000 (872–1150)	26·6 (18·9–37·3)	7·94 (3·60–14·4)
Bosnia and Herzegovina	281 (200–354)	1220 (1090–1340)	4·41 (3·33–6·08)	11·1 (5·03–18·6)	72·5 (52·1–104)	1000 (875–1140)	14·2 (9·54–19·9)	2·52 (1·16–4·73)
Bulgaria	681 (579–787)	2450 (2230–2690)	3·62 (3·00–4·38)	9·56 (9·37–9·77)	222 (190–254)	2270 (2060–2510)	10·3 (8·38–12·3)	1·13 (1·01–1·27)
Croatia	456 (399–510)	1930 (1760–2100)	4·26 (3·68–4·99)	17·9 (16·3–19·7)	147 (126–166)	1630 (1470–1790)	11·2 (9·40–13·3)	1·90 (1·72–2·10)
Czechia	1080 (943–1210)	5470 (5050–5910)	5·11 (4·43–5·92)	14·1 (13·9–14·4)	260 (228–294)	4110 (3690–4580)	15·9 (13·3–18·5)	1·41 (1·26–1·56)
Hungary	1210 (1080–1330)	5120 (4780–5500)	4·25 (3·73–4·86)	4·57 (4·38–4·78)	415 (366–462)	4840 (4390–5260)	11·7 (10·1–13·6)	0·274 (0·240–0·312)
Montenegro	72·5 (58·7–89·4)	349 (312–388)	4·87 (3·81–6·20)	20·5 (11·0–33·3)	22·9 (17·9–29·9)	338 (303–372)	15·0 (10·9–19·5)	6·74 (3·55–11·3)
North Macedonia	122 (92·0–153)	649 (573–720)	5·40 (4·01–7·38)	10·6 (4·55–17·4)	49·8 (37·2–67·3)	654 (571–748)	13·4 (9·55–18·1)	1·68 (0·839–3·19)
Poland	5050 (4550–5520)	25000 (20400–30000)	4·97 (3·97–6·16)	1·33 (1·30–1·37)	747 (659–831)	13500 (11100–16200)	18·1 (14·5–22·9)	0·139 (0·129–0·148)
Romania	1710 (1500–1930)	6920 (6380–7490)	4·07 (3·49–4·70)	0·844 (0·745–0·958)	331 (292–372)	5160 (4440–5980)	15·7 (12·9–18·9)	0·205 (0·179–0·236)
Serbia	1060 (866–1270)	4110 (3690–4510)	3·92 (3·10–4·86)	15·4 (7·11–24·8)	361 (285–470)	4060 (3670–4500)	11·4 (8·40–14·6)	2·53 (1·33–4·69)
Slovakia	565 (462–666)	2490 (2260–2730)	4·46 (3·61–5·53)	8·39 (3·56–14·9)	105 (83·0–139)	1720 (1510–1930)	16·6 (11·9–21·5)	1·50 (0·717–2·73)
Slovenia	288 (252–325)	1430 (1310–1540)	4·98 (4·27–5·78)	11·9 (11·4–12·4)	66·4 (55·4–76·9)	976 (883–1070)	14·8 (12·2–17·8)	0·877 (0·766–1·01)
Eastern Europe	39600 (35700–43500)	204000 (169000–243000)	5·18 (4·18–6·19)	3·62 (3·53–3·73)	9100 (8180–10200)	133000 (111000–157000)	14·6 (11·8–18·0)	0·282 (0·270–0·297)
Belarus	1550 (1260–1850)	7890 (7120–8660)	5·14 (4·12–6·37)	1·91 (1·63–2·25)	357 (287–436)	4850 (4400–5380)	13·8 (10·7–17·2)	0·00969 (0·00869–0·0109)
Estonia	159 (140–179)	803 (735–877)	5·08 (4·26–5·87)	7·88 (7·29–8·54)	40·4 (35·1–45·6)	519 (467–572)	12·9 (10·7–15·5)	0·680 (0·604–0·764)
Latvia	299 (262–337)	1250 (1140–1360)	4·21 (3·58–4·92)	5·37 (4·90–5·90)	64·5 (55·9–72·3)	781 (701–869)	12·2 (10·2–14·4)	0·476 (0·415–0·543)
Lithuania	628 (557–694)	2670 (2480–2870)	4·26 (3·70–4·90)	3·03 (2·81–3·27)	133 (116–151)	1650 (1530–1790)	12·4 (10·6–14·4)	0·281 (0·253–0·319)

Republic of Moldova	453 (402–513)	1910 (1760–2090)	4·24 (3·60–4·91)	2·02 (1·82–2·24)	83·1 (74·0–93·3)	1100 (965–1250)	13·3 (11·1–15·8)	0·408 (0·364–0·458)
Russian Federation	28200 (25600–30500)	136000 (111000–164000)	4·84 (3·90–5·94)	3·52 (3·46–3·59)	6700 (6020–7370)	95200 (78800–113000)	14·2 (11·6–17·5)	0·278 (0·269–0·286)
Ukraine	8260 (5630–11200)	53600 (43300–64600)	6·69 (4·42–9·87)	4·27 (3·97–4·62)	1720 (1130–2470)	28700 (23800–34000)	17·5 (11·1–26·1)	0·332 (0·291–0·381)
**High-income**	**112000 (107000–115000)**	**592000 (513000–676000)**	**5·28 (4·56–6·06)**	**25·1 (24·6–26·0)**	**36200 (33900–37500)**	**780000 (676000–898000)**	**21·6 (18·6–24·9)**	**10·4 (9·92–10·9)**
Australasia	2960 (2780–3120)	14200 (12800–15600)	4·81 (4·31–5·37)	7·22 (6·92–7·52)	940 (892–982)	18700 (16700–20800)	19·9 (17·6–22·3)	0·710 (0·655–0·780)
Australia	2520 (2370–2670)	11900 (10900–13000)	4·73 (4·25–5·26)	7·02 (6·66–7·38)	793 (751–831)	14900 (13400–16400)	18·8 (16·7–20·9)	0·706 (0·647–0·783)
New Zealand	438 (412–465)	2300 (1850–2810)	5·26 (4·21–6·49)	8·39 (7·95–8·86)	147 (138–155)	3760 (3140–4590)	25·7 (21·3–31·4)	0·727 (0·674–0·783)
High-income Asia Pacific	25600 (21500–26800)	127000 (108000–148000)	4·99 (4·17–6·20)	0·0797 (0·0610–0·122)	10500 (8810–11300)	178000 (156000–201000)	16·9 (14·5–20·4)	0·0181 (0·0157–0·0218)
Brunei Darussalam	14·7 (11·7–17·2)	98·3 (84·1–113)	6·77 (5·33–8·86)	0·161 (0·0561–0·290)	3·58 (2·69–4·57)	135 (113–160)	38·4 (28·2–52·3)	0·0374 (0·0138–0·0632)
Japan	15700 (15100–16100)	85900 (70900–103000)	5·48 (4·49–6·60)	0·0714 (0·0704–0·0725)	6630 (6020–7000)	115000 (95400–136000)	17·3 (14·1–21·1)	0·0215 (0·0190–0·0232)
Republic of Korea	9570 (5460–10600)	39700 (35300–44700)	4·24 (3·50–7·15)	0·0902 (0·0394–0·211)	3740 (2110–4270)	59800 (55800–64700)	16·4 (13·8–28·2)	0·0114 (0·00439–0·0212)
Singapore	297 (279–316)	1560 (1430–1690)	5·25 (4·72–5·87)	0·187 (0·175–0·199)	147 (135–158)	2850 (2540–3140)	19·5 (17·1–22·1)	0·0286 (0·0264–0·0313)
High-income North America	43100 (41700–44400)	270000 (224000–319000)	6·28 (5·18–7·52)	52·3 (51·8–52·8)	12400 (11900–12800)	380000 (315000–449000)	30·7 (25·2–36·5)	28·2 (27·5–28·9)
Canada	3590 (3400–3800)	27800 (25500–30400)	7·74 (6·89–8·50)	18·2 (17·4–19·2)	1100 (1040–1160)	38300 (34700–41900)	35·0 (31·1–38·9)	3·74 (3·34–4·16)
Greenland	25·6 (20·6–31·2)	107 (95·1–118)	4·21 (3·32–5·20)	29·7 (16·8–44·4)	7·83 (6·16–9·75)	149 (135–163)	19·4 (14·8–25·3)	8·08 (3·77–11·7)
United States of America	39400 (38100–40700)	242000 (198000–291000)	6·15 (4·98–7·45)	55·4 (54·9–56·0)	11300 (10800–11700)	341000 (279000–408000)	30·3 (24·6–36·5)	30·6 (29·8–31·3)
Southern Latin America	6080 (5780–6390)	34800 (31400–38300)	5·73 (5·15–6·40)	17·0 (16·3–17·6)	1410 (1330–1500)	33700 (29700–37800)	23·9 (20·8–27·5)	7·23 (6·61–8·00)
Argentina	3830 (3560–4070)	23500 (21200–26100)	6·15 (5·46–7·02)	21·1 (20·1–22·1)	930 (863–998)	21700 (19100–24600)	23·4 (20·3–27·2)	8·67 (7·79–9·75)
Chile	1640 (1550–1740)	9000 (8140–9890)	5·47 (4·90–6·13)	4·98 (4·74–5·23)	350 (330–371)	9670 (8350–11100)	27·7 (23·7–32·5)	1·81 (1·68–1·97)
Uruguay	598 (558–638)	2240 (2020–2460)	3·75 (3·32–4·19)	23·6 (22·5–24·7)	128 (119–138)	2250 (2030–2490)	17·6 (15·4–20·0)	11·6 (10·7–12·6)

Western Europe	34400 (32800–35900)	145000 (132000–158000)	4·22 (3·83–4·66)	12·5 (12·1–12·9)	10900 (10200–11400)	170000 (151000–189000)	15·6 (13·8–17·7)	1·56 (1·48–1·64)
Andorra	5·38 (3·60–7·24)	27·7 (24·4–31·4)	5·33 (3·70–7·94)	8·34 (4·55–25·2)	1·38 (0·889–1·93)	39·1 (34·3–44·2)	29·5 (19·7–44·4)	0·650 (0·289–2·76)
Austria	990 (928–1050)	4160 (3870–4460)	4·21 (3·85–4·61)	21·1 (20·3–21·9)	298 (273–319)	4180 (3820–4590)	14·1 (12·4–16·0)	2·44 (2·22–2·68)
Belgium	1440 (1350–1530)	6560 (6020–7050)	4·56 (4·13–5·04)	10·2 (9·80–10·5)	545 (504–579)	8480 (7750–9290)	15·6 (13·9–17·5)	1·95 (1·78–2·14)
Cyprus	43·4 (36·6–51·0)	331 (290–374)	7·69 (6·07–9·54)	20·9 (10·5–34·9)	11·5 (9·20–14·9)	370 (302–444)	32·7 (22·8–44·0)	0·166 (0·0682–0·345)
Denmark	474 (442–505)	1850 (1690–2010)	3·91 (3·48–4·37)	13·3 (12·6–14·1)	186 (169–201)	2350 (2080–2670)	12·7 (11·0–14·8)	0·560 (0·503–0·625)
Finland	655 (603–706)	2880 (2620–3140)	4·41 (3·90–4·98)	22·0 (20·9–23·1)	212 (196–228)	3350 (3020–3680)	15·8 (13·9–17·9)	1·67 (1·51–1·84)
France	7090 (6500–7790)	30300 (27600–32800)	4·28 (3·75–4·84)	20·9 (19·9–21·9)	2030 (1870–2170)	33800 (30400–37500)	16·7 (14·8–19·2)	2·90 (2·66–3·19)
Germany	8040 (7550–8500)	32100 (29600–34700)	3·99 (3·62–4·42)	9·76 (9·34–10·2)	2470 (2250–2640)	35400 (32100–38800)	14·3 (12·7–16·3)	1·17 (1·06–1·29)
Greece	428 (393–463)	1570 (1400–1740)	3·69 (3·21–4·18)	21·4 (20·2–22·6)	103 (94·5–112)	2490 (2080–2980)	24·1 (19·5–29·2)	3·42 (3·10–3·80)
Iceland	34·9 (31·7–38·3)	152 (140–165)	4·37 (3·81–4·97)	13·0 (12·2–13·8)	8·87 (7·87–9·84)	123 (108–142)	14·0 (11·6–17·0)	0·710 (0·646–0·801)
Ireland	335 (303–373)	2040 (1850–2210)	6·12 (5·29–7·04)	5·76 (5·49–6·04)	81·0 (74·4–87·0)	1880 (1640–2120)	23·2 (19·6–27·2)	0·912 (0·838–0·996)
Israel	400 (373–426)	2260 (2040–2480)	5·65 (5·02–6·42)	31·1 (30·1–32·2)	94·7 (86·8–102)	2420 (2010–2900)	25·6 (21·0–31·1)	9·06 (8·34–10·0)
Italy	3020 (2860–3170)	7390 (6140–8790)	2·45 (2·01–2·89)	14·2 (13·9–14·4)	836 (761–887)	8550 (7290–9950)	10·2 (8·57–12·3)	2·41 (2·29–2·55)
Luxembourg	42·9 (38·4–47·7)	245 (221–269)	5·72 (4·97–6·61)	12·6 (12·0–13·3)	15·2 (13·6–16·7)	265 (236–297)	17·6 (14·9–20·7)	1·69 (1·53–1·86)
Malta	17·8 (16·0–19·7)	92·3 (83·0–102)	5·19 (4·43–6·10)	11·1 (10·5–11·7)	4·09 (3·58–4·59)	90·2 (73·7–108)	22·2 (17·4–27·5)	2·06 (1·87–2·29)
Monaco	3·92 (3·11–4·92)	14·3 (12·5–15·9)	3·69 (2·77–4·82)	15·1 (7·33–25·9)	1·92 (1·37–2·62)	20·0 (17·8–22·4)	10·7 (7·23–14·9)	14·7 (8·54–22·4)
Netherlands	1300 (1220–1370)	5950 (5450–6420)	4·57 (4·13–5·07)	3·11 (3·00–3·23)	631 (587–672)	9510 (8590–10400)	15·1 (13·4–17·0)	0·272 (0·247–0·299)
Norway	449 (428–470)	2240 (1760–2770)	4·98 (3·87–6·21)	15·9 (15·5–16·2)	184 (174–194)	3180 (2560–3920)	17·3 (13·7–21·2)	1·45 (1·37–1·53)
Portugal	919 (863–981)	2790 (2540–3030)	3·04 (2·72–3·39)	10·5 (10·2–10·8)	314 (284–336)	3560 (3170–4010)	11·4 (9·80–13·3)	1·42 (1·27–1·60)

San Marino	1·93 (1·28–2·74)	12·4 (10·9–13·9)	6·67 (4·41–9·91)	34·1 (18·6–55·0)	0·554 (0·338–0·827)	13·9 (12·3–15·9)	26·5 (17·2–42·6)	1·50 (0·800–2·86)
Spain	2640 (2440–2820)	11300 (10200–12300)	4·27 (3·80–4·80)	7·00 (6·47–7·56)	881 (811–948)	12400 (10800–14000)	14·1 (12·0–16·4)	0·466 (0·426–0·507)
Sweden	875 (765–999)	5110 (4170–6160)	5·86 (4·70–7·25)	11·3 (10·9–11·8)	403 (351–458)	7250 (5980–8670)	18·1 (14·4–22·4)	0·630 (0·583–0·675)
Switzerland	776 (723–823)	3570 (3300–3850)	4·61 (4·16–5·11)	27·0 (25·9–28·1)	270 (246–292)	4880 (4450–5380)	18·1 (16·0–20·7)	2·96 (2·73–3·24)
United Kingdom	4390 (4290–4480)	22200 (18500–26300)	5·06 (4·20–5·95)	1·88 (1·84–1·90)	1360 (1310–1390)	25500 (21200–30300)	18·7 (15·6–22·1)	0·236 (0·228–0·244)
**Latin America and Caribbean**	**33600 (31500–35900)**	**61800 (50500–73800)**	**1·84 (1·50–2·23)**	**10·7 (10·0–11·4)**	**8730 (8080–9320)**	**67500 (55800–80100)**	**7·75 (6·33–9·31)**	**3·86 (3·48–4·15)**
Andean Latin America	2460 (1980–2890)	6130 (5170–7100)	2·52 (1·96–3·26)	5·34 (3·73–7·25)	783 (608–956)	8590 (7370–9950)	11·1 (8·30–14·8)	1·35 (0·762–1·94)
Bolivia (Plurinational State of)	449 (299–603)	912 (762–1080)	2·10 (1·41–3·08)	7·61 (3·25–12·2)	203 (132–299)	1570 (1290–1910)	8·09 (5·00–13·0)	2·34 (1·07–3·56)
Ecuador	1210 (964–1510)	2850 (2380–3330)	2·37 (1·75–3·14)	5·06 (4·68–5·43)	322 (258–394)	3520 (3110–4020)	11·1 (8·45–14·4)	0·468 (0·420–0·518)
Peru	794 (554–1010)	2370 (1990–2780)	3·06 (2·23–4·34)	4·48 (1·81–7·63)	258 (159–350)	3500 (2840–4220)	14·0 (9·41–22·0)	1·67 (0·827–3·04)
Caribbean	3420 (2990–3860)	6110 (5430–6750)	1·79 (1·51–2·12)	5·80 (4·05–8·12)	951 (794–1120)	7850 (6780–9060)	8·32 (6·54–10·4)	2·51 (1·52–3·43)
Antigua and Barbuda	1·09 (1·01–1·19)	6·32 (5·36–7·37)	5·79 (4·84–6·87)	1·87 (1·76–2·01)	0·136 (0·124–0·147)	11·4 (8·90–14·3)	84·3 (64·8–107)	1·35 (1·23–1·47)
Bahamas	8·89 (7·08–11·0)	23·0 (19·8–26·6)	2·62 (1·99–3·46)	5·90 (5·48–6·35)	1·24 (0·964–1·59)	47·0 (36·9–58·0)	38·4 (27·2–52·2)	0·847 (0·755–0·947)
Barbados	11·9 (9·01–15·0)	24·2 (21·3–26·9)	2·08 (1·57–2·76)	4·57 (4·30–4·83)	2·11 (1·63–2·63)	36·3 (29·8–43·6)	17·5 (12·5–24·0)	6·79 (5·86–7·71)
Belize	18·9 (16·3–21·7)	52·3 (45·6–59·5)	2·78 (2·28–3·41)	8·89 (8·40–9·39)	2·90 (2·54–3·29)	49·5 (39·9–61·2)	17·2 (13·4–22·0)	5·85 (5·19–6·54)
Bermuda	2·17 (1·84–2·66)	6·46 (5·78–7·11)	3·01 (2·38–3·66)	0·755 (0·714–0·804)	0·366 (0·306–0·455)	9·77 (8·00–11·8)	27·1 (19·4–35·6)	0·715 (0·645–0·801)
Cuba	1400 (1200–1600)	2440 (2230–2640)	1·75 (1·48–2·06)	1·96 (1·85–2·07)	380 (323–435)	2400 (2160–2640)	6·34 (5·33–7·58)	1·58 (1·43–1·75)
Dominica	2·54 (1·98–3·16)	5·83 (5·00–6·71)	2·33 (1·73–3·10)	2·58 (0·970–4·21)	0·293 (0·222–0·400)	6·88 (5·58–8·36)	24·1 (15·4–33·2)	0·442 (0·167–0·703)
Dominican Republic	483 (378–633)	1140 (960–1350)	2·41 (1·72–3·18)	13·9 (6·37–22·6)	110 (81·0–150)	1200 (999–1450)	11·2 (7·55–16·1)	7·60 (3·05–10·8)
Grenada	3·85 (3·28–4·45)	8·13 (7·07–9·13)	2·12 (1·74–2·58)	0·213 (0·200–0·226)	0·899 (0·761–1·05)	11·0 (8·90–13·3)	12·3 (9·41–16·1)	0·0425 (0·0388–0·0464)

Guyana	199 (153–254)	262 (227–301)	1·34 (0·996–1·81)	1·28 (1·20–1·37)	40·0 (29·5–52·1)	254 (218–294)	6·49 (4·63–8·77)	0·274 (0·245–0·306)
Haiti	647 (423–894)	861 (694–1040)	1·38 (0·909–2·10)	6·01 (2·17–12·2)	276 (127–390)	2290 (1880–2750)	8·95 (5·46–18·6)	1·85 (0·481–4·80)
Jamaica	34·0 (25·4–44·0)	166 (140–192)	4·99 (3·45–6·89)	25·8 (24·7–26·9)	6·19 (4·61–8·19)	337 (260–424)	55·8 (36·8–80·5)	27·6 (25·5–29·7)
Puerto Rico	198 (162–235)	471 (423–520)	2·39 (1·94–2·96)	17·9 (17·1–18·9)	35·5 (29·4–41·9)	473 (388–567)	13·5 (10·1–17·4)	1·97 (1·84–2·12)
Saint Kitts and Nevis	1·46 (1·20–1·78)	4·76 (4·02–5·48)	3·31 (2·52–4·23)	0·941 (0·875–1·01)	0·217 (0·173–0·265)	7·59 (5·90–9·39)	35·4 (25·2–47·0)	1·67 (1·50–1·84)
Saint Lucia	11·0 (9·08–13·3)	22·5 (19·8–25·3)	2·07 (1·61–2·60)	2·46 (2·32–2·60)	1·04 (0·837–1·26)	19·8 (16·0–24·2)	19·3 (14·0–25·4)	1·58 (1·41–1·76)
Saint Vincent and the Grenadines	6·52 (5·67–7·51)	11·9 (10·4–13·4)	1·83 (1·50–2·18)	1·65 (1·56–1·74)	0·544 (0·467–0·630)	11·2 (8·83–13·7)	20·8 (15·5–26·6)	0·652 (0·586–0·726)
Suriname	107 (83·4–132)	157 (137–177)	1·49 (1·14–1·99)	4·12 (1·87–7·40)	29·7 (23·2–40·4)	191 (169–217)	6·55 (4·42–8·64)	1·58 (0·676–2·50)
Trinidad and Tobago	162 (123–207)	232 (201–261)	1·46 (1·06–1·95)	1·72 (1·61–1·83)	30·6 (23·2–39·4)	218 (191–249)	7·26 (5·15–9·70)	0·501 (0·443–0·556)
United States Virgin Islands	5·32 (4·11–6·79)	10·3 (8·96–11·7)	1·97 (1·44–2·59)	30·4 (16·9–46·5)	0·708 (0·488–1·14)	9·96 (8·44–11·9)	14·8 (8·37–21·7)	8·19 (4·53–18·5)
Central Latin America	14000 (12600–15600)	29800 (24300–36100)	2·14 (1·67–2·67)	13·5 (12·6–14·4)	3170 (2780–3540)	31200 (25200–37900)	9·89 (7·67–12·3)	4·92 (4·46–5·32)
Colombia	2670 (2240–3150)	5580 (4850–6330)	2·11 (1·68–2·62)	12·1 (11·3–12·8)	642 (543–745)	5830 (4980–6890)	9·16 (7·14–11·7)	3·41 (3·07–3·81)
Costa Rica	398 (356–441)	787 (698–877)	1·98 (1·69–2·32)	11·7 (10·8–12·6)	69·5 (61·0–77·3)	557 (467–659)	8·05 (6·53–9·96)	3·78 (3·39–4·22)
El Salvador	402 (316–498)	804 (670–951)	2·03 (1·50–2·67)	2·42 (0·894–3·81)	134 (98·9–170)	983 (850–1150)	7·46 (5·37–10·4)	0·917 (0·444–1·51)
Guatemala	645 (552–740)	1180 (1000–1360)	1·83 (1·46–2·26)	8·40 (7·94–8·93)	210 (179–244)	1990 (1650–2430)	9·51 (7·39–12·3)	2·75 (2·49–3·05)
Honduras	318 (225–439)	440 (331–562)	1·43 (0·871–2·14)	18·4 (8·07–29·2)	77·0 (47·0–116)	711 (517–959)	9·81 (5·48–16·6)	7·19 (3·17–12·3)
Mexico	7260 (6360–8280)	17000 (12800–22000)	2·35 (1·71–3·18)	8·77 (8·44–9·14)	1580 (1350–1820)	16700 (12600–21200)	10·7 (7·64–14·1)	4·18 (3·93–4·44)
Nicaragua	293 (237–357)	644 (550–746)	2·22 (1·73–2·80)	5·88 (2·72–11·0)	87·9 (65·5–114)	861 (730–1020)	9·98 (7·25–13·6)	1·42 (0·846–2·81)
Panama	191 (154–228)	393 (343–448)	2·08 (1·61–2·65)	4·68 (4·34–5·02)	36·1 (29·5–43·2)	388 (314–473)	10·9 (8·12–14·4)	1·33 (1·18–1·50)
Venezuela (Bolivarian Republic of)	1830 (1390–2340)	3020 (2600–3510)	1·69 (1·22–2·27)	40·1 (38·3–42·0)	331 (256–425)	3140 (2670–3730)	9·68 (6·89–13·2)	15·4 (13·1–17·5)

Tropical Latin America	13700 (13200–14300)	19700 (15500–24300)	1·44 (1·13–1·79)	9·98 (9·61–10·4)	3830 (3660–3980)	19900 (16000–23900)	5·21 (4·19–6·32)	3·83 (3·61–4·03)
Brazil	13400 (12900–13900)	19100 (15000–23700)	1·43 (1·11–1·78)	9·80 (9·49–10·1)	3720 (3560–3880)	19200 (15400–23000)	5·17 (4·11–6·27)	3·67 (3·50–3·84)
Paraguay	312 (232–401)	599 (498–707)	1·96 (1·40–2·71)	18·0 (9·81–31·8)	103 (73·1–136)	705 (596–833)	6·98 (4·89–9·84)	9·76 (5·94–17·6)
**North Africa and Middle East**	**15500 (12800–17900)**	**103000 (85600–122000)**	**6·70 (5·26–8·60)**	**9·91 (4·72–15·7)**	**5710 (4370–6870)**	**134000 (110000–163000)**	**23·8 (17·9–32·2)**	**4·86 (2·31–7·27)**
North Africa and Middle East	15500 (12800–17900)	103000 (85600–122000)	6·70 (5·26–8·60)	9·91 (4·72–15·7)	5710 (4370–6870)	134000 (110000–163000)	23·8 (17·9–32·2)	4·86 (2·31–7·27)
Afghanistan	664 (479–970)	3050 (2430–3760)	4·75 (2·83–6·90)	6·82 (2·55–13·9)	460 (315–844)	6810 (5600–8270)	15·7 (7·90–22·7)	4·14 (1·03–9·62)
Algeria	808 (568–1040)	5950 (4870–7190)	7·52 (5·38–10·8)	4·58 (1·72–9·02)	413 (236–558)	9960 (8340–11800)	25·1 (16·6–42·0)	2·25 (0·839–3·93)
Bahrain	57·9 (48·0–70·2)	466 (404–529)	8·12 (6·35–10·2)	1·54 (0·556–3·03)	7·52 (5·95–11·2)	278 (230–328)	37·9 (23·1–49·7)	0·721 (0·270–1·24)
Egypt	808 (636–992)	13100 (10500–16100)	16·5 (11·9–22·9)	9·42 (4·00–15·8)	256 (189–337)	19000 (15300–23000)	75·7 (52·9–107)	3·92 (1·81–6·47)
Iran (Islamic Republic of)	2800 (2400–3090)	19900 (15600–24700)	7·13 (5·55–9·25)	4·25 (1·46–5·98)	906 (780–1180)	21000 (16500–26100)	23·4 (16·0–30·7)	4·06 (1·57–5·64)
Iraq	1400 (1040–1890)	8330 (6930–9800)	6·11 (4·10–8·47)	20·8 (10·6–33·1)	420 (305–607)	9730 (8010–11700)	23·9 (15·1–34·0)	11·9 (7·39–18·4)
Jordan	106 (84·2–132)	1520 (1270–1810)	14·6 (10·8–19·4)	5·01 (1·81–8·00)	26·5 (19·6–37·5)	2150 (1610–2770)	83·5 (51·9–122)	3·85 (1·51–6·01)
Kuwait	74·9 (61·0–91·4)	780 (687–883)	10·5 (8·31–13·5)	1·15 (1·06–1·26)	16·5 (14·3–19·1)	953 (742–1190)	58·1 (43·3–76·5)	1·67 (1·49–1·87)
Lebanon	32·7 (26·7–40·1)	990 (814–1190)	30·6 (23·1–39·7)	28·4 (14·2–44·3)	19·2 (14·4–25·4)	1270 (1070–1500)	67·8 (48·7–92·0)	18·9 (10·5–28·4)
Libya	269 (184–356)	1320 (1090–1560)	5·04 (3·53–7·54)	4·52 (1·92–7·93)	110 (59·1–168)	1820 (1510–2180)	17·6 (10·4–31·8)	2·44 (0·968–4·89)
Morocco	800 (541–1200)	7230 (6040–8650)	9·40 (5·74–14·1)	4·10 (1·56–7·58)	567 (291–873)	10700 (8920–12600)	20·0 (12·0–39·1)	2·37 (0·795–4·49)
Oman	43·0 (32·7–55·6)	781 (643–935)	18·5 (13·0–25·3)	0·867 (0·221–1·43)	6·05 (4·40–8·19)	657 (506–829)	111 (74·1–164)	0·498 (0·122–0·865)
Palestine	32·4 (24·1–39·0)	677 (569–817)	21·2 (16·4–29·3)	3·76 (1·26–6·60)	7·86 (6·24–10·4)	880 (663–1140)	114 (73·0–157)	3·05 (0·856–4·85)
Qatar	107 (79·4–141)	867 (734–1010)	8·31 (5·78–11·6)	0·315 (0·0935–0·681)	6·76 (4·81–10·3)	382 (304–460)	58·9 (35·6–84·3)	0·205 (0·0707–0·457)
Saudi Arabia	2410 (1730–3300)	8850 (7350–10300)	3·78 (2·58–5·24)	5·48 (2·26–8·69)	556 (395–765)	6510 (5130–7820)	12·1 (7·63–17·3)	2·92 (1·23–4·96)

Sudan	1190 (718–1710)	7370 (5920–9060)	6·48 (3·93–10·5)	6·16 (2·45–11·1)	664 (276–1140)	11700 (9310–14400)	19·8 (9·79–43·5)	4·32 (1·36–9·51)
Syrian Arab Republic	96·9 (70·5–134)	1490 (1240–1800)	15·8 (10·5–22·6)	8·07 (3·10–13·5)	28·3 (20·1–42·9)	2640 (2000–3380)	96·7 (58·5–143)	2·51 (1·04–4·70)
Tunisia	260 (169–390)	1700 (1420–1990)	6·83 (4·18–10·4)	3·51 (1·35–6·34)	103 (58·2–154)	2390 (1960–2840)	24·7 (14·9–44·3)	2·15 (0·825–4·03)
Türkiye	2520 (1890–3080)	11200 (9740–12800)	4·51 (3·52–5·93)	25·0 (12·3–37·9)	686 (468–878)	16400 (13400–20100)	24·5 (17·3–34·7)	10·0 (4·87–14·7)
United Arab Emirates	194 (148–267)	2820 (2310–3320)	14·9 (9·82–20·2)	1·38 (0·473–2·47)	35·0 (26·0–51·2)	1260 (1020–1510)	37·1 (23·0–52·0)	0·651 (0·266–1·12)
Yemen	786 (401–1190)	4510 (3670–5480)	6·13 (3·47–10·7)	5·84 (2·09–12·6)	410 (140–649)	7680 (6130–9460)	21·3 (11·0–55·4)	3·21 (0·854–8·37)
**South Asia**	**131000 (112000–149000)**	**491000 (388000–603000)**	**3·78 (2·92–4·96)**	**8·44 (3·58–15·5)**	**81600 (63200–94900)**	**1230000 (975000–1530000)**	**15·2 (11·4–20·7)**	**1·41 (0·475–2·64)**
South Asia	131000 (112000–149000)	491000 (388000–603000)	3·78 (2·92–4·96)	8·44 (3·58–15·5)	81600 (63200–94900)	1230000 (975000–1530000)	15·2 (11·4–20·7)	1·41 (0·475–2·64)
Bangladesh	4200 (3210–5460)	26000 (22100–31000)	6·33 (4·56–8·53)	8·50 (2·27–23·2)	1850 (1280–3900)	42400 (35000–51100)	24·6 (10·3–35·1)	1·20 (0·370–2·75)
Bhutan	29·7 (20·9–41·7)	137 (110–167)	4·76 (3·06–6·71)	12·6 (4·99–25·2)	13·8 (9·41–22·5)	208 (168–259)	15·8 (8·78–23·3)	2·15 (0·795–4·71)
India	114000 (94300–131000)	397000 (311000–489000)	3·52 (2·72–4·71)	8·16 (3·57–14·7)	74900 (53200–87700)	1110000 (879000–1380000)	15·0 (11·0–21·9)	1·40 (0·471–2·63)
Nepal	2700 (2070–3570)	9160 (7740–10700)	3·46 (2·48–4·70)	10·7 (3·83–23·7)	1370 (768–1970)	8340 (6830–10300)	6·44 (3·92–11·3)	1·65 (0·505–3·76)
Pakistan	10200 (6420–16500)	58900 (45400–74100)	6·19 (3·18–9·77)	11·1 (4·69–22·0)	3510 (2040–7320)	70700 (54600–89300)	21·8 (9·49–36·9)	1·62 (0·559–3·78)
**Southeast Asia, East Asia, and Oceania**	**108000 (89000–131000)**	**502000 (418000–595000)**	**4·71 (3·67–6·17)**	**0·774 (0·351–1·36)**	**63900 (51300–83300)**	**697000 (586000–824000)**	**11·1 (7·81–14·5)**	**0·0684 (0·0360–0·136)**
East Asia	81700 (64400–104000)	364000 (299000–435000)	4·53 (3·34–6·17)	0·0873 (0·0458–0·146)	55500 (43200–74400)	571000 (475000–676000)	10·5 (7·21–13·9)	0·0150 (0·00823–0·0296)
China	77500 (60200–99800)	349000 (286000–419000)	4·58 (3·33–6·29)	0·0669 (0·0252–0·117)	53000 (40700–71600)	549000 (455000–653000)	10·6 (7·16–14·1)	0·0145 (0·00794–0·0292)
Democratic People's Republic of Korea	1610 (1120–2540)	5470 (4650–6340)	3·54 (2·16–5·02)	0·346 (0·154–1·03)	1260 (801–2430)	9240 (7910–10600)	7·79 (3·73–11·5)	0·0344 (0·0113–0·0966)
Taiwan (Province of China)	2560 (2370–2740)	10000 (9180–10900)	3·93 (3·49–4·40)	0·535 (0·500–0·576)	1230 (1120–1320)	12200 (11200–13300)	9·97 (8·94–11·2)	0·0180 (0·0165–0·0197)
Oceania	471 (398–561)	2920 (2320–3580)	6·26 (4·48–7·99)	4·63 (1·60–7·08)	187 (148–282)	2400 (1970–2950)	13·1 (7·99–17·2)	0·0498 (0·0201–0·131)
American Samoa	3·05 (2·31–3·84)	13·3 (11·1–15·5)	4·43 (3·32–6·16)	10·4 (5·09–18·9)	0·955 (0·702–1·25)	12·4 (10·3–14·6)	13·3 (9·17–18·6)	0·00466 (0·00215–0·0105)

Cook Islands	1·56 (1·24–1·96)	7·91 (6·89–9·07)	5·14 (3·81–6·64)	3·49 (0·864–6·66)	0·485 (0·361–0·649)	7·17 (6·10–8·36)	15·1 (10·4–20·7)	0·0502 (0·0173–0·135)
Fiji	57·4 (42·2–73·8)	263 (219–306)	4·68 (3·41–6·59)	0·0844 (0·0245–0·124)	24·8 (17·5–33·9)	302 (253–364)	12·5 (8·39–17·7)	0·0156 (0·00257–0·0244)
Guam	23·5 (20·3–26·3)	117 (103–132)	5·00 (4·19–6·02)	9·15 (4·80–15·8)	3·70 (2·88–4·34)	83·7 (72·9–96·0)	22·9 (18·1–30·2)	0·0626 (0·0250–0·107)
Kiribati	17·8 (12·7–23·1)	76·3 (64·1–89·4)	4·39 (3·06–6·35)	0·490 (0·126–0·772)	3·11 (2·16–4·29)	45·6 (37·5–55·0)	15·1 (10·2–22·2)	0·129 (0·0313–0·246)
Marshall Islands	8·35 (4·87–11·5)	29·9 (25·3–35·2)	3·75 (2·49–6·18)	2·92 (0·757–7·17)	2·93 (1·46–4·08)	29·6 (24·7–35·8)	10·8 (6·72–21·3)	0·0203 (0·00645–0·0608)
Micronesia (Federated States of)	16·1 (10·6–21·0)	60·0 (50·3–71·3)	3·85 (2·65–5·80)	4·03 (1·18–7·62)	4·63 (2·97–6·46)	52·3 (43·5–62·9)	11·7 (7·66–18·0)	0·0403 (0·0153–0·0903)
Nauru	1·95 (1·02–2·60)	5·97 (4·94–7·16)	3·22 (2·15–5·76)	2·85 (0·779–4·93)	0·645 (0·344–0·924)	5·68 (4·70–6·94)	9·39 (5·80–16·0)	0·0265 (0·00951–0·0506)
Niue	0·173 (0·127–0·230)	0·650 (0·542–0·776)	3·85 (2·64–5·45)	3·73 (1·08–6·19)	0·0873 (0·0683–0·115)	0·535 (0·442–0·655)	6·23 (4·48–8·57)	0·0361 (0·00758–0·0945)
Northern Mariana Islands	6·28 (5·23–7·32)	31·6 (27·7–35·9)	5·06 (4·05–6·28)	8·85 (4·49–16·8)	1·28 (0·921–1·54)	14·7 (12·5–17·1)	11·7 (9·08–17·1)	0·0287 (0·0139–0·105)
Palau	1·71 (1·33–2·17)	7·50 (6·42–8·63)	4·47 (3·28–5·90)	5·82 (2·27–9·50)	1·01 (0·744–1·34)	6·16 (5·29–7·18)	6·26 (4·42–8·41)	0·256 (0·0390–0·424)
Papua New Guinea	154 (114–261)	1510 (1130–1980)	10·2 (5·62–14·4)	6·10 (1·75–11·4)	84·3 (55·6–172)	1350 (1070–1700)	17·3 (7·17–26·0)	0·0585 (0·0155–0·185)
Samoa	18·8 (13·5–24·7)	85·1 (70·9–101)	4·64 (3·28–6·72)	4·23 (1·29–6·58)	7·21 (4·89–10·1)	81·4 (67·2–98·2)	11·7 (7·63–17·2)	0·0474 (0·0200–0·106)
Solomon Islands	95·0 (64·2–126)	407 (332–481)	4·42 (2·97–6·73)	4·80 (1·52–8·42)	29·9 (18·0–44·4)	142 (116–174)	4·98 (3·10–8·20)	0·0482 (0·0178–0·117)
Tokelau	0·137 (0·110–0·174)	0·416 (0·347–0·495)	3·09 (2·22–4·03)	3·89 (1·20–6·28)	0·0804 (0·0567–0·107)	0·503 (0·417–0·606)	6·44 (4·46–9·40)	0·0348 (0·00726–0·0982)
Tonga	3·16 (2·33–4·24)	19·4 (15·7–23·7)	6·28 (4·19–8·78)	4·32 (1·08–7·31)	1·77 (1·24–2·51)	23·7 (19·4–28·7)	13·8 (8·87–20·0)	0·0517 (0·0167–0·152)
Tuvalu	1·53 (1·10–1·99)	5·36 (4·44–6·34)	3·59 (2·51–5·14)	3·11 (0·799–6·58)	0·507 (0·339–0·707)	5·03 (4·13–6·04)	10·3 (6·66–15·2)	0·0311 (0·00962–0·0708)
Vanuatu	39·5 (27·8–49·7)	146 (121–173)	3·79 (2·72–5·44)	4·46 (1·37–7·86)	11·5 (7·93–16·1)	127 (105–153)	11·4 (7·44–16·8)	0·0437 (0·0170–0·108)
Southeast Asia	25400 (21400–29500)	134000 (113000–157000)	5·34 (4·21–6·70)	2·89 (1·38–5·41)	8200 (6670–10400)	124000 (103000–148000)	15·3 (11·2–19·6)	0·425 (0·237–0·866)
Cambodia	540 (387–723)	2680 (2270–3170)	5·10 (3·43–7·37)	2·05 (1·15–4·97)	234 (156–340)	2940 (2410–3560)	13·1 (8·25–19·9)	0·342 (0·153–0·894)
Indonesia	3120 (2410–4200)	31200 (24100–38800)	10·2 (6·65–14·0)	1·89 (0·975–5·11)	1450 (1060–2050)	30200 (23000–37700)	21·3 (13·9–29·8)	0·280 (0·142–0·648)

Lao People's Democratic Republic	281 (200–387)	1450 (1190–1730)	5·31 (3·47–7·49)	1·98 (1·02–4·57)	105 (67·7–151)	1600 (1300–1930)	16·1 (9·62–24·6)	0·367 (0·130–0·940)
Malaysia	1320 (1170–1510)	6850 (5650–8250)	5·24 (4·11–6·60)	2·52 (0·918–3·80)	327 (272–463)	5460 (4390–6840)	16·9 (11·4–22·2)	0·183 (0·0588–0·261)
Maldives	10·6 (8·13–13·8)	121 (103–141)	11·6 (8·58–15·5)	1·66 (0·537–3·52)	0·878 (0·651–1·18)	65·1 (49·2–81·5)	75·7 (48·6–106)	0·296 (0·111–0·533)
Mauritius	113 (104–119)	454 (410–505)	4·03 (3·56–4·61)	1·52 (1·43–1·63)	26·4 (23·8–28·1)	387 (338–438)	14·7 (12·4–17·2)	0·819 (0·742–0·908)
Myanmar	1650 (1090–2160)	7840 (6570–9230)	4·90 (3·38–7·22)	2·50 (1·26–5·92)	367 (263–505)	7670 (6030–9660)	21·5 (14·1–31·3)	0·392 (0·148–1·06)
Philippines	3470 (2710–4280)	17600 (13600–22200)	5·15 (3·63–7·00)	2·35 (0·852–3·57)	803 (600–1030)	15100 (11400–19300)	19·2 (13·3–28·6)	0·472 (0·135–0·595)
Seychelles	5·51 (4·61–6·35)	32·8 (28·4–36·8)	6·01 (4·98–7·56)	1·33 (0·654–3·10)	0·565 (0·461–0·699)	14·8 (11·7–18·2)	26·6 (19·1–35·8)	0·216 (0·104–0·519)
Sri Lanka	2840 (1890–3980)	12200 (10600–13800)	4·45 (2·96–6·38)	0·482 (0·165–0·796)	782 (535–1160)	12600 (10900–14600)	16·8 (10·6–24·5)	0·167 (0·0745–0·269)
Thailand	6470 (4950–8140)	23500 (20800–26400)	3·71 (2·70–4·87)	6·01 (2·74–10·2)	1530 (1120–2050)	15500 (13500–18100)	10·4 (7·23–15·1)	1·00 (0·481–1·61)
Timor-Leste	39·8 (27·9–56·8)	236 (191–287)	6·13 (3·85–8·85)	2·33 (1·20–6·67)	17·2 (12·0–24·5)	264 (211–331)	15·9 (9·88–23·6)	0·380 (0·154–1·03)
Viet Nam	5490 (4060–7040)	30000 (25900–34600)	5·60 (4·07–7·74)	1·77 (0·839–4·92)	2550 (1830–3350)	32100 (27600–37100)	12·9 (9·22–17·6)	0·268 (0·146–0·861)
**Sub-Saharan Africa**	**60000 (51600–70300)**	**162000 (134000–193000)**	**2·73 (2·09–3·46)**	**4·68 (1·61–8·00)**	**17100 (12800–21100)**	**155000 (126000–189000)**	**9·16 (6·78–12·7)**	**5·31 (2·32–8·70)**
Central Sub-Saharan Africa	8540 (6460–11800)	20200 (16900–23600)	2·42 (1·65–3·35)	3·82 (1·41–7·73)	2480 (1700–3640)	16700 (13700–20400)	6·96 (4·28–9·90)	6·21 (2·45–12·4)
Angola	1790 (1330–2340)	4630 (3880–5460)	2·64 (1·87–3·71)	2·81 (1·06–4·53)	483 (317–696)	3780 (3070–4640)	8·17 (5·05–12·1)	5·21 (2·05–7·97)
Central African Republic	504 (346–707)	965 (796–1140)	1·98 (1·26–2·88)	4·38 (1·53–10·2)	135 (83·7–197)	812 (680–961)	6·30 (3·89–10·1)	7·61 (2·60–14·6)
Congo	388 (281–517)	873 (731–1030)	2·31 (1·56–3·29)	2·69 (1·05–4·20)	128 (81·8–173)	852 (711–1020)	6·90 (4·61–10·6)	4·31 (1·49–6·99)
Democratic Republic of the Congo	5630 (3970–8320)	13100 (10900–15300)	2·40 (1·50–3·45)	4·22 (1·49–9·23)	1680 (1120–2730)	10800 (8820–13200)	6·70 (3·80–9·73)	6·59 (2·39–16·1)
Equatorial Guinea	84·5 (57·8–124)	287 (237–347)	3·55 (2·24–5·25)	2·46 (0·820–4·30)	19·7 (10·4–36·3)	189 (152–233)	10·6 (5·05–18·8)	4·59 (1·51–8·56)
Gabon	146 (103–194)	389 (330–453)	2·74 (1·88–3·87)	2·19 (0·681–3·63)	29·4 (18·6–50·4)	251 (202–306)	9·02 (4·89–14·1)	4·38 (1·67–7·87)
Eastern Sub-Saharan Africa	22000 (18900–27000)	53400 (44100–63500)	2·44 (1·83–3·09)	4·56 (1·67–8·51)	6540 (5220–8150)	53600 (43700–65600)	8·32 (6·01–11·2)	7·24 (3·01–12·6)

Burundi	724 (533–1140)	1710 (1420–2020)	2·44 (1·44–3·42)	4·78 (1·37–11·0)	218 (143–333)	1870 (1540–2230)	8·92 (5·35–13·7)	6·30 (1·60–17·8)
Comoros	29·1 (17·6–51·1)	84·8 (70·4–100)	3·20 (1·56–4·96)	4·39 (1·38–9·43)	17·9 (12·5–24·6)	120 (101–141)	6·90 (4·63–9·90)	5·24 (1·68–10·1)
Djibouti	52·0 (29·0–96·6)	181 (149–214)	3·93 (1·78–6·58)	3·91 (1·38–7·50)	18·8 (11·5–29·2)	150 (124–182)	8·43 (4·91–13·5)	6·01 (2·52–8·95)
Eritrea	476 (321–687)	1030 (846–1210)	2·25 (1·41–3·33)	5·02 (2·09–8·32)	162 (113–220)	958 (799–1140)	6·10 (4·04–8·86)	7·64 (2·93–11·8)
Ethiopia	5160 (4140–6320)	12100 (9440–15100)	2·37 (1·69–3·15)	4·47 (1·38–9·56)	1560 (1170–2080)	14400 (11300–17800)	9·47 (6·22–13·4)	7·73 (3·04–13·8)
Kenya	2620 (1950–3830)	6780 (5450–8250)	2·66 (1·70–3·68)	3·27 (1·13–6·24)	797 (561–1240)	6510 (5190–7990)	8·50 (5·02–12·6)	5·11 (2·02–8·99)
Madagascar	1270 (875–1790)	3180 (2620–3780)	2·58 (1·60–3·76)	5·24 (2·04–9·25)	526 (360–705)	3980 (3330–4800)	7·80 (5·33–11·7)	8·16 (3·56–14·4)
Malawi	1310 (991–1670)	2840 (2400–3330)	2·21 (1·56–3·01)	5·79 (2·05–12·1)	304 (197–474)	2130 (1690–2690)	7·34 (4·21–11·6)	6·66 (2·21–16·7)
Mozambique	2370 (1670–3150)	4710 (4000–5530)	2·05 (1·43–2·95)	5·08 (2·01–8·71)	511 (318–745)	3740 (3050–4630)	7·65 (4·91–12·0)	8·52 (3·40–14·7)
Rwanda	719 (503–1040)	1670 (1380–1960)	2·40 (1·54–3·43)	3·52 (1·01–7·47)	258 (160–395)	1840 (1540–2230)	7·54 (4·50–11·6)	4·96 (1·55–10·1)
Somalia	785 (409–1550)	2370 (1900–2940)	3·49 (1·47–6·17)	8·93 (2·80–20·3)	463 (285–759)	2650 (2150–3260)	6·05 (3·26–9·58)	10·6 (2·95–30·3)
South Sudan	591 (406–843)	918 (745–1110)	1·61 (1·06–2·39)	5·79 (2·26–9·13)	185 (129–263)	996 (782–1250)	5·57 (3·42–8·50)	10·9 (4·21–17·3)
Uganda	2090 (1560–2630)	5400 (4500–6460)	2·64 (1·89–3·63)	3·04 (0·986–6·27)	441 (301–598)	4520 (3570–5740)	10·6 (6·90–15·9)	5·56 (1·76–11·0)
United Republic of Tanzania	2530 (1870–3330)	7140 (6040–8380)	2·89 (2·02–4·00)	4·54 (1·70–8·30)	778 (546–1070)	7130 (5810–8780)	9·46 (6·23–13·5)	6·39 (2·71–10·4)
Zambia	1300 (943–1720)	3240 (2760–3770)	2·56 (1·77–3·56)	4·27 (1·73–7·05)	294 (186–447)	2580 (2110–3160)	9·19 (5·51–14·4)	7·02 (3·17–11·4)
Southern Sub-Saharan Africa	10000 (8630–11500)	30400 (23600–37600)	3·06 (2·30–4·14)	3·55 (1·27–6·02)	2550 (1970–3170)	22600 (17900–27800)	8·96 (6·27–12·2)	2·58 (0·932–4·51)
Botswana	186 (129–272)	1070 (884–1260)	5·94 (3·79–8·75)	5·01 (1·93–7·71)	46·5 (30·3–87·7)	781 (650–930)	17·9 (8·85–26·5)	4·82 (1·74–9·10)
Eswatini	177 (121–238)	538 (443–635)	3·14 (2·19–4·67)	4·69 (1·66–8·13)	36·2 (18·9–58·8)	341 (276–414)	10·2 (5·45–18·4)	5·25 (1·88–9·46)
Lesotho	296 (210–401)	1030 (852–1220)	3·56 (2·42–5·20)	5·09 (1·88–9·24)	89·5 (47·0–136)	744 (604–898)	9·06 (5·01–16·2)	4·91 (1·10–9·57)
Namibia	236 (162–324)	855 (703–1020)	3·74 (2·48–5·32)	4·70 (1·82–7·37)	45·2 (27·1–77·8)	586 (480–710)	14·0 (6·94–22·6)	4·37 (1·84–7·96)

South Africa	7120 (6240–8010)	22100 (16500–28400)	3·12 (2·26–4·24)	3·22 (1·01–5·65)	1580 (1310–2130)	14300 (10800–18200)	9·13 (5·97–12·5)	2·63 (0·862–5·75)
Zimbabwe	1990 (1340–2660)	4840 (4020–5690)	2·51 (1·69–3·84)	4·20 (1·82–8·16)	754 (351–1070)	5850 (4880–6980)	8·28 (5·27–16·6)	1·89 (0·574–4·30)
Western Sub-Saharan Africa	19500 (15600–23100)	58400 (48100–69400)	3·04 (2·33–4·04)	5·75 (1·85–9·41)	5560 (3720–7220)	61700 (50200–75500)	11·4 (8·19–17·3)	3·91 (2·08–5·60)
Benin	553 (391–838)	1780 (1480–2090)	3·33 (2·05–4·67)	6·27 (1·95–10·3)	165 (92·5–248)	1730 (1430–2110)	11·1 (6·52–19·5)	3·73 (2·13–5·76)
Burkina Faso	1150 (883–1520)	3000 (2510–3550)	2·66 (1·87–3·64)	6·48 (2·05–10·9)	344 (237–492)	3130 (2580–3780)	9·43 (6·02–13·8)	4·47 (2·18–6·49)
Cabo Verde	72·4 (54·5–93·8)	238 (209–269)	3·35 (2·46–4·49)	6·32 (2·77–10·9)	11·9 (8·69–15·4)	116 (99·7–135)	9·98 (7·08–13·9)	3·45 (2·48–5·54)
Cameroon	1800 (1160–2580)	5530 (4660–6430)	3·19 (2·05–4·82)	5·06 (1·67–7·94)	473 (247–694)	4770 (3990–5700)	10·8 (6·38–19·8)	2·80 (1·45–4·44)
Chad	696 (453–1210)	2040 (1700–2440)	3·11 (1·66–4·67)	7·40 (2·45–12·2)	268 (166–370)	2110 (1730–2570)	8·26 (5·28–13·7)	4·15 (1·91–6·71)
Côte d'Ivoire	1660 (1190–2390)	4790 (4070–5600)	2·98 (1·93–4·31)	5·90 (1·96–9·01)	307 (156–494)	3360 (2790–4110)	11·7 (6·39–23·0)	3·38 (1·86–4·96)
Gambia	95·6 (59·0–158)	308 (251–371)	3·51 (1·83–5·58)	6·38 (1·96–11·5)	38·0 (26·0–52·5)	308 (255–376)	8·40 (5·55–11·9)	3·31 (1·38–7·04)
Ghana	1530 (1180–1990)	5780 (4950–6710)	3·85 (2·75–5·12)	1·83 (0·659–3·08)	411 (296–599)	3750 (2960–4740)	9·46 (5·82–13·8)	7·58 (3·86–13·1)
Guinea	449 (276–738)	1610 (1330–1920)	3·86 (2·04–5·95)	7·50 (2·47–12·0)	225 (133–321)	2000 (1680–2400)	9·34 (6·09–15·2)	4·02 (2·05–5·85)
Guinea-Bissau	128 (90·5–180)	313 (260–376)	2·53 (1·65–3·76)	7·34 (2·43–11·3)	46·9 (30·8–62·4)	349 (291–420)	7·68 (5·28–12·0)	3·35 (1·48–4·87)
Liberia	259 (176–379)	663 (551–797)	2·66 (1·62–4·01)	8·30 (2·30–17·6)	104 (54·0–173)	857 (713–1030)	9·02 (4·68–17·0)	3·38 (1·58–5·80)
Mali	628 (407–1030)	2270 (1850–2720)	3·90 (2·12–5·90)	9·83 (2·72–19·5)	359 (225–499)	3040 (2520–3700)	8·88 (5·67–14·2)	3·96 (1·79–5·81)
Mauritania	108 (65·4–201)	439 (359–535)	4·48 (2·17–7·12)	6·24 (1·99–11·0)	48·3 (28·2–71·2)	532 (433–660)	11·6 (7·37–18·5)	4·17 (2·13–6·21)
Niger	573 (336–1010)	2230 (1810–2700)	4·27 (2·11–6·93)	10·5 (3·07–20·7)	318 (185–472)	2760 (2250–3410)	9·16 (5·44–15·4)	4·21 (1·88–6·78)
Nigeria	7990 (5340–10900)	22400 (17900–27600)	2·90 (1·95–4·30)	5·31 (1·67–9·82)	1880 (1130–3110)	28200 (22300–34900)	16·0 (8·77–25·8)	3·38 (1·89–4·82)
Sao Tome and Principe	1·93 (1·33–2·74)	18·9 (15·1–23·2)	10·2 (6·58–15·7)	6·76 (1·80–13·7)	0·561 (0·315–0·825)	23·5 (17·7–30·3)	44·1 (26·6–73·6)	2·47 (1·33–4·48)
Senegal	945 (731–1280)	2510 (2120–2930)	2·71 (1·87–3·56)	6·49 (2·21–11·2)	257 (184–364)	2060 (1690–2510)	8·28 (5·28–11·8)	4·39 (2·04–6·12)

Sierra Leone	304<br>(187–503)	1100<br>(924–1290)	3·90<br>(2·06–6·12)	7·00<br>(2·18–12·8)	147<br>(76·3–208)	1390<br>(1160–1660)	9·99<br>(6·20–18·5)	3·40<br>(1·64–5·40)
Togo	509<br>(345–718)	1360<br>(1150–1580)	2·77<br>(1·89–4·02)	5·86<br>(1·84–10·7)	151<br>(84·8–225)	1190<br>(993–1410)	8·31<br>(5·04–14·4)	3·12<br>(1·46–5·81)

Appendix Table S8: The rate and rank of suicides by age groups 10-29, 30-49, 50-69, and 70+ for males and females. Estimates provided at the global, super-region, regional, and national level.

	Male 10 to 29		Female: 10 to 29		Male: 30 to 49		Female: 30 to 49		Male: 50 to 69		Female: 50 to 69		Male: 70+		Female: 70+	
**Location**	**Mortality Rate**	**Rank**	**Mortality Rate**	**Rank**	**Mortality Rate**	**Rank**	**Mortality Rate**	**Rank**	**Mortality Rate**	**Rank**	**Mortality Rate**	**Rank**	**Mortality Rate**	**Rank**	**Mortality Rate**	**Rank**
**Global**	**9·49 (8·81–10·2)**	**3**	**5·31 (4·62–6·04)**	**3**	**17·1 (15·9–18·2)**	**7**	**6·01 (5·12–6·69)**	**11**	**20·8 (19·1–22·3)**	**17**	**8·35 (7·05–9·42)**	**25**	**37·9 (33·4–41·4)**	**28**	**15·6 (12·7–18·0)**	**47**
Low SDI	7·17 (6·04–8·67)	7	3·93 (3·18–5·11)	8	15·4 (12·9–18·6)	11	4·73 (3·76–5·93)	19	29·2 (24·4–34·6)	15	10·0 (7·69–12·1)	30	63·1 (53·8–72·3)	25	20·4 (15·8–24·2)	39
Low-middle SDI	10·8 (9·26–12·2)	3	8·19 (6·59–9·57)	3	19·5 (15·8–21·9)	8	8·20 (5·77–9·75)	10	20·3 (16·3–23·0)	19	8·84 (6·34–10·4)	28	30·4 (23·9–34·1)	32	13·3 (9·95–15·6)	46
Middle SDI	8·22 (7·55–8·90)	3	4·70 (4·01–5·46)	3	12·8 (11·6–14·1)	8	4·91 (4·13–5·80)	10	15·3 (13·1–17·7)	18	7·84 (6·02–9·46)	24	38·5 (31·0–45·5)	26	20·6 (14·9–25·2)	32
High-middle SDI	8·83 (8·23–9·46)	2	2·86 (2·55–3·44)	2	17·5 (16·4–18·8)	4	4·70 (4·11–5·74)	10	20·0 (18·1–22·2)	16	7·52 (6·37–9·33)	21	37·8 (33·4–43·0)	24	16·5 (13·6–21·7)	38
High SDI	14·2 (13·7–14·6)	1	4·51 (4·36–4·67)	1	23·6 (22·8–24·3)	1	7·75 (7·31–8·01)	3	28·5 (27·1–29·5)	8	9·25 (8·71–9·64)	14	37·1 (33·4–39·4)	30	10·5 (8·50–11·7)	55
**Central Europe, Eastern Europe, and Central Asia**	**18·4 (17·4–19·4)**	**1**	**4·67 (4·43–4·95)**	**1**	**42·6 (40·6–44·8)**	**2**	**6·75 (6·39–7·15)**	**9**	**42·8 (40·7–45·1)**	**10**	**7·90 (7·48–8·37)**	**21**	**54·3 (50·7–57·6)**	**22**	**14·5 (13·2–15·5)**	**40**
Central Asia	12·7 (11·8–13·7)	2	6·41 (5·83–7·07)	1	25·4 (23·5–27·3)	4	4·82 (4·38–5·33)	9	23·1 (21·1–25·2)	16	4·73 (4·32–5·20)	30	30·6 (27·9–33·4)	25	11·7 (10·5–12·7)	37
Armenia	4·82 (4·28–5·36)	3	1·37 (1·21–1·54)	4	9·98 (8·94–11·1)	6	1·52 (1·34–1·72)	18	14·1 (12·2–16·3)	23	3·35 (2·88–3·92)	34	25·4 (21·5–29·7)	32	10·3 (8·78–12·0)	42
Azerbaijan	3·64 (2·55–5·16)	7	1·08 (0·758–1·64)	14	5·42 (3·78–7·92)	13	0·956 (0·650–1·49)	30	6·06 (4·33–8·28)	33	1·66 (1·12–2·37)	42	9·42 (6·59–12·7)	38	4·44 (2·66–6·19)	48
Georgia	9·26 (7·91–10·8)	2	2·11 (1·82–2·43)	2	19·4 (16·7–22·4)	5	2·27 (1·95–2·62)	18	21·6 (18·7–24·7)	22	3·90 (3·39–4·47)	36	31·1 (26·5–36·2)	34	8·99 (7·51–10·5)	47
Kazakhstan	21·4 (19·3–23·6)	1	6·72 (6·08–7·40)	1	55·4 (49·6–61·4)	1	8·31 (7·35–9·30)	7	51·4 (44·7–58·6)	10	7·23 (6·12–8·36)	25	64·6 (55·6–74·6)	19	18·6 (15·8–21·3)	28
Kyrgyzstan	11·6 (9·73–13·6)	2	4·10 (3·42–4·85)	1	27·2 (23·3–31·6)	5	4·86 (3·97–5·89)	8	23·2 (19·2–27·9)	15	6·09 (5·19–7·11)	23	31·1 (25·9–37·4)	22	13·5 (11·1–16·1)	32
Mongolia	24·8 (18·3–31·1)	1	4·73 (3·13–6·52)	1	47·1 (35·6–60·7)	3	4·95 (3·33–7·10)	14	42·6 (31·1–57·5)	13	6·25 (4·19–9·14)	29	43·1 (30·4–59·9)	22	12·2 (8·11–18·0)	41

Tajikistan	4·36 (2·73–7·31)	7	2·33 (1·35–4·25)	6	9·41 (6·09–14·8)	11	2·18 (1·30–3·91)	19	8·44 (5·63–12·6)	21	3·00 (1·89–4·74)	32	11·1 (6·57–16·0)	32	6·38 (3·68–9·65)	37
Turkmenistan	14·9 (12·1–18·3)	1	6·18 (4·86–7·82)	2	21·8 (17·7–26·9)	5	4·71 (3·60–6·14)	14	19·6 (15·2–25·4)	17	6·40 (5·00–8·24)	26	26·8 (21·5–33·0)	23	11·3 (9·01–13·9)	27
Uzbekistan	13·5 (11·9–15·2)	2	10·5 (9·24–11·8)	1	19·5 (17·0–22·2)	4	5·35 (4·55–6·28)	8	16·4 (13·8–19·5)	14	4·42 (3·76–5·23)	29	20·8 (17·4–24·7)	26	10·2 (8·53–11·9)	29
Central Europe	12·0 (11·5–12·6)	2	2·25 (2·10–2·42)	2	25·5 (24·3–26·6)	2	4·32 (4·04–4·70)	8	34·2 (32·3–36·1)	14	7·03 (6·53–7·66)	24	43·5 (40·0–46·5)	25	9·84 (8·81–10·8)	46
Albania	3·10 (1·99–4·77)	3	2·14 (1·30–3·26)	2	6·94 (4·64–10·1)	8	3·09 (1·97–4·66)	7	7·03 (4·71–10·1)	25	3·75 (2·39–5·59)	25	13·5 (9·36–19·1)	35	8·24 (5·02–12·3)	40
Bosnia and Herzegovina	10·9 (8·22–13·9)	1	1·91 (1·34–2·68)	1	17·2 (12·7–22·2)	3	3·61 (2·53–5·15)	9	25·4 (18·7–33·8)	13	6·17 (4·32–9·07)	25	44·6 (33·0–59·2)	23	11·3 (8·15–15·9)	42
Bulgaria	9·61 (8·31–11·0)	2	3·13 (2·59–3·72)	2	18·4 (15·7–21·3)	5	5·73 (4·78–6·83)	10	29·5 (25·4–33·8)	16	6·99 (5·93–8·17)	23	49·8 (42·7–57·6)	21	13·3 (11·4–15·4)	35
Croatia	8·81 (7·55–10·1)	2	1·78 (1·52–2·05)	2	19·0 (16·4–21·8)	2	5·30 (4·49–6·15)	3	34·5 (30·2–39·3)	7	9·58 (8·03–11·2)	18	56·5 (48·2–65·7)	25	15·4 (13·0–17·9)	43
Czechia	11·7 (10·6–12·9)	1	2·21 (1·90–2·52)	1	22·6 (20·1–25·2)	1	4·43 (3·78–5·12)	4	29·9 (26·0–33·9)	11	7·11 (6·15–8·16)	23	43·1 (37·2–49·3)	25	9·34 (7·87–10·8)	52
Hungary	10·9 (9·68–12·2)	1	2·70 (2·30–3·14)	1	24·3 (21·5–27·2)	2	6·41 (5·57–7·33)	6	42·6 (38·0–47·5)	11	12·4 (10·7–14·1)	16	67·6 (59·1–76·2)	21	17·2 (14·7–19·8)	40
Montenegro	12·1 (8·93–16·1)	1	3·08 (2·17–4·29)	1	28·6 (21·3–37·4)	2	6·98 (4·94–9·69)	6	37·6 (27·2–51·0)	10	11·7 (8·03–16·6)	14	66·4 (48·0–88·6)	22	21·2 (15·0–29·1)	30
North Macedonia	5·95 (4·22–8·18)	2	1·99 (1·35–2·99)	3	10·8 (7·93–14·6)	5	3·71 (2·56–5·35)	8	18·0 (12·9–24·4)	18	7·38 (5·04–10·6)	22	35·2 (25·3–46·9)	27	14·2 (10·0–19·7)	38
Poland	17·8 (16·9–18·7)	1	2·56 (2·39–2·72)	2	34·6 (32·8–36·4)	1	3·77 (3·50–4·04)	6	39·6 (37·0–42·2)	9	5·66 (5·24–6·07)	30	32·3 (29·2–35·0)	32	5·23 (4·59–5·66)	58
Romania	8·26 (7·17–9·45)	2	1·54 (1·29–1·84)	3	20·3 (17·8–23·0)	3	3·35 (2·85–3·91)	12	28·9 (25·1–32·8)	13	4·82 (4·14–5·55)	27	31·8 (27·5–36·4)	25	5·88 (5·00–6·81)	51
Serbia	7·61 (5·89–9·45)	2	2·15 (1·58–3·00)	2	22·0 (17·2–27·6)	2	6·11 (4·49–8·34)	6	37·0 (28·7–48·2)	10	11·7 (8·57–16·2)	20	70·7 (53·3–90·3)	20	21·7 (16·1–28·5)	35
Slovakia	11·0 (8·31–14·0)	1	1·86 (1·28–2·57)	2	24·1 (18·3–30·7)	3	3·54 (2·46–4·82)	9	34·3 (25·0–45·1)	9	5·71 (3·93–8·10)	28	42·7 (31·6–56·3)	25	7·65 (5·41–10·7)	47

Slovenia	10·2 (8·92–11·5)	1	1·68 (1·38–1·98)	1	21·4 (18·7–24·3)	1	4·38 (3·61–5·22)	3	40·7 (35·0–46·8)	7	9·47 (7·83–11·3)	14	81·7 (69·2–95·7)	21	16·8 (13·5–20·0)	44
Eastern Europe	25·7 (24·0–27·5)	1	4·88 (4·57–5·22)	2	59·2 (55·5–63·3)	2	8·78 (8·17–9·47)	8	53·9 (50·3–58·1)	9	9·17 (8·55–9·87)	22	68·4 (62·9–73·9)	19	17·9 (16·0–19·2)	34
Belarus	18·3 (15·8–20·9)	1	3·39 (2·81–4·04)	1	41·6 (35·3–48·6)	3	7·14 (5·89–8·57)	8	50·9 (42·4–59·6)	9	7·92 (6·53–9·58)	20	73·8 (62·1–86·1)	13	16·2 (13·6–19·1)	29
Estonia	13·6 (11·5–15·5)	1	2·45 (2·04–2·90)	1	24·7 (21·0–28·4)	1	3·50 (2·89–4·17)	8	41·1 (34·7–48·0)	12	6·52 (5·39–7·74)	24	58·3 (48·8–68·8)	22	15·6 (12·6–18·6)	40
Latvia	16·2 (14·1–18·3)	1	2·09 (1·72–2·50)	3	39·4 (34·0–44·9)	2	5·01 (4·14–5·94)	8	53·7 (46·0–62·0)	8	7·89 (6·59–9·34)	22	63·3 (53·9–74·0)	21	15·0 (12·5–17·6)	39
Lithuania	24·9 (22·3–27·5)	1	2·74 (2·30–3·21)	2	63·5 (56·5–70·6)	1	6·91 (5·85–8·12)	5	73·5 (64·0–83·4)	6	12·5 (10·7–14·6)	15	87·2 (74·8–101)	16	20·7 (17·2–24·2)	31
Republic of Moldova	14·9 (13·0–17·0)	1	2·04 (1·67–2·45)	3	31·6 (27·5–36·0)	3	3·78 (3·15–4·50)	9	45·1 (38·8–52·3)	11	6·57 (5·53–7·76)	24	35·5 (29·8–41·9)	21	8·71 (7·22–10·3)	36
Russian Federation	26·4 (24·9–27·7)	1	5·25 (5·00–5·48)	2	63·2 (60·0–66·3)	2	9·62 (9·09–10·1)	8	55·4 (52·4–58·3)	10	9·46 (8·94–9·97)	21	68·2 (63·4–72·3)	19	19·1 (17·2–20·3)	35
Ukraine	27·1 (21·6–33·3)	1	4·52 (3·34–5·96)	2	53·8 (40·9–69·4)	3	7·16 (5·02–9·90)	8	49·7 (36·6–65·5)	9	8·65 (6·49–11·4)	18	69·5 (54·4–87·7)	13	15·2 (12·0–19·0)	23
**High-income**	**14·6 (14·2–15·0)**	**1**	**4·52 (4·36–4·64)**	**1**	**24·4 (23·5–25·0)**	**1**	**7·70 (7·26–7·92)**	**3**	**28·0 (26·5–29·0)**	**9**	**8·97 (8·45–9·31)**	**15**	**35·9 (32·1–38·2)**	**30**	**9·39 (7·61–10·5)**	**58**
Australasia	18·0 (16·5–19·3)	1	5·87 (5·40–6·33)	1	27·5 (25·0–29·9)	1	8·13 (7·35–8·93)	2	23·5 (21·2–25·9)	6	7·62 (6·77–8·58)	14	21·2 (18·3–23·9)	40	6·14 (4·93–7·14)	62
Australia	17·9 (16·2–19·5)	1	5·53 (4·98–6·07)	1	28·3 (25·4–31·1)	1	8·42 (7·50–9·38)	2	24·0 (21·4–26·9)	6	7·90 (6·89–9·05)	13	21·5 (18·3–24·5)	40	6·25 (4·96–7·38)	61
New Zealand	18·1 (16·3–19·9)	1	7·46 (6·83–8·09)	1	23·3 (21·0–25·8)	1	6·57 (5·83–7·35)	2	21·0 (18·4–23·7)	7	6·24 (5·39–7·14)	17	19·4 (16·3–22·4)	41	5·56 (4·46–6·60)	60
High-income Asia Pacific	15·4 (14·4–16·0)	1	7·80 (6·79–8·16)	1	29·8 (25·7–31·5)	1	11·8 (9·51–12·7)	1	37·5 (30·3–40·9)	7	12·3 (10·2–13·5)	9	43·8 (34·7–48·5)	24	17·1 (13·1–20·0)	34
Brunei Darussalam	4·25 (2·90–5·98)	2	1·05 (0·661–1·58)	5	8·86 (5·93–12·4)	5	2·39 (1·53–3·47)	15	10·1 (6·72–14·3)	21	3·25 (2·00–4·72)	41	9·90 (6·87–13·6)	53	5·29 (3·31–7·55)	69
Japan	16·4 (16·0–16·7)	1	7·24 (7·10–7·40)	1	28·1 (27·4–28·7)	1	10·3 (9·97–10·5)	1	32·3 (31·2–33·5)	8	11·9 (11·1–12·5)	9	34·0 (30·5–36·1)	30	14·1 (10·7–16·0)	40

Republic of Korea	14·1 (10·9–15·9)	1	9·40 (6·21–10·5)	1	36·3 (22·8–41·7)	1	16·2 (8·63–18·9)	1	51·3 (27·6–62·2)	4	13·5 (6·78–16·9)	4	99·5 (41·7–125)	12	33·0 (18·3–43·8)	22
Singapore	10·2 (9·11–11·3)	1	4·48 (4·02–4·94)	1	8·56 (7·46–9·70)	2	4·33 (3·76–4·92)	2	14·5 (12·6–16·6)	8	7·58 (6·46–8·82)	10	24·6 (20·7–28·9)	26	13·6 (10·9–16·1)	29
High-income North America	19·8 (19·1–20·5)	1	5·34 (5·15–5·55)	3	29·3 (28·3–30·3)	2	9·15 (8·83–9·50)	4	30·7 (29·7–31·7)	10	9·66 (9·22–10·0)	19	35·0 (31·6–37·1)	31	5·22 (4·47–5·65)	66
Canada	18·1 (16·2–20·0)	1	6·62 (5·92–7·34)	1	25·9 (23·2–28·9)	2	7·21 (6·39–8·08)	3	23·4 (20·9–26·2)	7	7·39 (6·54–8·32)	16	21·3 (18·3–24·3)	39	4·74 (3·90–5·53)	67
Greenland	84·2 (61·3–101)	1	28·0 (18·6–35·7)	1	129 (96·3–161)	1	46·3 (31·5–62·3)	1	116 (84·1–166)	3	41·0 (27·6–58·2)	7	110 (76·0–195)	13	13·6 (8·94–23·3)	45
United States of America	19·9 (19·2–20·6)	1	5·21 (5·01–5·42)	3	29·7 (28·7–30·7)	2	9·37 (9·02–9·74)	4	31·6 (30·5–32·6)	11	9·92 (9·48–10·3)	19	36·8 (33·1–39·0)	30	5·27 (4·51–5·71)	65
Southern Latin America	19·2 (17·4–21·1)	1	4·73 (4·25–5·23)	1	22·6 (20·6–24·7)	1	4·96 (4·44–5·51)	6	24·8 (22·5–27·3)	15	5·38 (4·85–5·99)	30	38·7 (34·3–43·2)	30	5·63 (4·83–6·31)	66
Argentina	20·4 (17·9–23·0)	1	5·05 (4·40–5·75)	1	21·0 (18·3–23·8)	2	4·77 (4·10–5·49)	10	23·1 (19·9–26·5)	16	5·37 (4·63–6·20)	31	37·3 (31·8–43·3)	29	5·57 (4·63–6·47)	64
Chile	14·5 (12·8–16·2)	1	3·63 (3·15–4·12)	2	23·5 (21·1–26·1)	1	4·78 (4·15–5·46)	5	23·9 (21·0–27·0)	10	4·50 (3·87–5·19)	28	34·1 (29·2–39·2)	28	4·73 (3·87–5·56)	67
Uruguay	28·6 (25·0–32·2)	1	5·98 (5·16–6·84)	1	40·3 (35·7–45·2)	1	8·52 (7·25–9·86)	4	49·1 (42·4–56·3)	7	10·3 (8·65–12·1)	16	73·1 (62·2–85·2)	22	9·40 (7·67–11·1)	56
Western Europe	7·97 (7·60–8·31)	1	2·32 (2·23–2·41)	1	18·0 (17·3–18·8)	1	5·13 (4·93–5·32)	2	22·2 (20·9–23·6)	9	7·49 (7·05–7·89)	14	33·1 (29·5–35·7)	33	8·42 (6·96–9·30)	62
Andorra	7·04 (4·75–9·87)	2	1·56 (0·907–2·43)	2	15·2 (9·68–22·0)	1	3·88 (2·33–6·02)	3	16·0 (9·66–24·2)	13	5·30 (3·22–8·26)	20	27·9 (17·6–40·4)	31	6·39 (3·83–9·81)	54
Austria	11·4 (10·3–12·6)	1	2·88 (2·52–3·25)	1	21·9 (19·8–24·1)	1	6·44 (5·65–7·27)	2	30·1 (26·5–33·8)	7	10·0 (8·69–11·5)	11	65·4 (55·7–74·7)	24	14·0 (11·3–16·4)	45
Belgium	11·6 (10·4–12·8)	1	3·28 (2·91–3·69)	1	31·1 (28·2–34·2)	1	10·1 (8·97–11·3)	2	37·0 (32·7–41·9)	6	16·0 (13·9–18·2)	10	44·4 (37·3–51·4)	30	14·6 (11·6–17·1)	55
Cyprus	4·01 (2·75–5·49)	2	0·700 (0·449–1·11)	4	7·11 (5·00–9·80)	3	1·59 (1·05–2·34)	10	9·10 (6·27–12·7)	21	2·88 (1·89–4·17)	32	17·4 (12·1–24·0)	42	4·45 (2·85–6·57)	71
Denmark	6·87 (6·01–7·80)	1	2·44 (2·11–2·79)	1	16·1 (14·3–18·2)	1	5·31 (4·60–6·11)	3	24·4 (21·1–27·9)	10	10·6 (9·07–12·2)	11	39·8 (33·5–46·4)	27	13·2 (10·8–15·5)	49

Finland	17·5 (15·6–19·5)	1	5·64 (4·99–6·34)	1	26·7 (23·9–29·8)	1	7·75 (6·81–8·83)	2	29·6 (25·5–34·0)	8	10·6 (9·07–12·3)	10	43·0 (35·9–50·2)	27	11·6 (9·12–13·8)	49
France	8·02 (6·96–9·16)	2	2·17 (1·88–2·47)	2	27·8 (24·8–31·4)	1	6·31 (5·49–7·20)	2	32·4 (27·4–38·6)	6	10·0 (8·62–11·5)	10	49·5 (41·3–58·1)	29	10·8 (8·56–12·9)	57
Germany	8·52 (7·67–9·36)	1	2·55 (2·27–2·84)	1	17·8 (16·0–19·8)	1	4·96 (4·38–5·57)	2	25·4 (22·4–28·6)	9	8·23 (7·24–9·29)	14	44·9 (38·2–51·5)	28	11·5 (9·24–13·3)	55
Greece	3·55 (2·97–4·20)	2	0·786 (0·649–0·941)	5	8·64 (7·44–9·94)	4	1·89 (1·59–2·22)	12	12·2 (10·3–14·2)	20	2·74 (2·30–3·24)	29	14·6 (12·2–17·0)	46	2·87 (2·31–3·40)	71
Iceland	12·3 (10·6–14·1)	1	2·05 (1·70–2·42)	2	25·2 (21·9–28·8)	1	5·19 (4·34–6·16)	4	22·0 (18·3–26·1)	5	7·27 (6·00–8·67)	11	45·0 (36·6–53·8)	25	12·0 (9·47–14·5)	47
Ireland	9·67 (7·88–11·8)	1	1·87 (1·58–2·19)	1	19·1 (16·6–21·9)	1	4·22 (3·57–4·97)	3	19·7 (16·7–23·3)	10	5·31 (4·43–6·31)	19	30·2 (24·5–36·5)	30	6·49 (5·16–7·75)	60
Israel	5·45 (4·70–6·23)	2	1·11 (0·930–1·31)	2	11·7 (10·2–13·2)	1	2·60 (2·21–3·03)	3	15·9 (13·7–18·3)	13	3·82 (3·23–4·47)	24	23·7 (19·5–27·9)	37	5·40 (4·27–6·44)	68
Italy	4·79 (4·46–5·13)	2	1·11 (1·04–1·19)	2	10·0 (9·51–10·6)	2	2·68 (2·53–2·84)	5	13·6 (12·8–14·4)	13	3·70 (3·43–3·93)	26	22·0 (19·4–23·8)	43	4·52 (3·56–5·08)	68
Luxembourg	5·58 (4·65–6·57)	2	1·89 (1·59–2·23)	2	12·7 (10·7–14·9)	1	4·21 (3·53–4·95)	2	22·4 (18·6–26·7)	9	8·16 (6·80–9·68)	12	40·8 (33·9–48·3)	28	12·0 (9·76–14·2)	56
Malta	4·63 (3·80–5·54)	1	0·845 (0·677–1·04)	3	10·3 (8·69–12·2)	2	2·02 (1·66–2·45)	10	9·70 (8·04–11·7)	20	2·39 (1·94–2·87)	34	16·3 (13·3–19·7)	37	2·90 (2·27–3·51)	67
Monaco	13·7 (9·20–19·7)	1	5·60 (3·65–8·23)	1	28·5 (17·4–42·1)	1	10·4 (6·24–16·1)	3	20·3 (13·6–29·2)	10	11·6 (7·42–17·6)	12	36·3 (24·5–50·6)	33	15·5 (9·44–23·7)	44
Netherlands	9·02 (8·17–9·89)	1	3·94 (3·56–4·35)	1	19·2 (17·4–21·1)	1	7·90 (6·99–8·89)	2	22·0 (19·5–24·8)	9	11·4 (9·87–13·1)	10	25·9 (21·7–30·1)	39	11·2 (9·09–13·1)	55
Norway	13·8 (12·9–14·6)	1	5·02 (4·77–5·28)	1	21·3 (20·2–22·5)	1	8·94 (8·42–9·49)	1	22·7 (21·2–24·4)	6	10·7 (9·88–11·5)	9	22·1 (19·7–24·1)	36	6·97 (5·94–7·72)	62
Portugal	5·79 (4·92–6·71)	2	1·75 (1·48–2·02)	2	15·6 (13·7–17·7)	2	4·63 (3·98–5·31)	3	25·8 (22·2–29·6)	10	7·78 (6·65–9·00)	14	46·4 (39·2–54·2)	25	11·7 (9·43–13·8)	52
San Marino	8·07 (5·62–11·1)	2	1·76 (1·08–2·70)	2	16·9 (11·0–24·1)	1	4·09 (2·50–6·30)	6	17·5 (11·1–26·0)	10	5·50 (3·38–8·60)	17	38·4 (25·7–54·5)	26	7·18 (4·26–11·0)	54
Spain	4·76 (4·11–5·43)	2	1·40 (1·19–1·61)	2	12·3 (10·8–13·8)	2	3·70 (3·23–4·21)	3	15·9 (13·8–18·3)	11	5·84 (5·06–6·74)	15	27·1 (22·9–31·3)	40	6·34 (4·94–7·54)	62

Sweden	13·3 (12·0–14·7)	1	6·07 (5·57–6·60)	1	19·6 (17·7–21·7)	1	9·65 (8·68–10·7)	1	26·4 (23·5–29·5)	4	12·2 (10·7–13·7)	9	29·3 (25·1–33·4)	32	10·5 (8·64–12·2)	56
Switzerland	9·17 (8·09–10·2)	1	2·88 (2·54–3·23)	1	14·9 (13·3–16·6)	1	5·76 (5·06–6·54)	2	25·9 (22·6–29·4)	3	10·1 (8·74–11·7)	8	45·0 (37·7–52·1)	25	11·1 (8·63–13·2)	53
United Kingdom	9·33 (8·93–9·68)	1	2·46 (2·38–2·54)	1	20·5 (19·9–21·1)	1	5·74 (5·58–5·91)	5	17·0 (16·5–17·5)	12	5·54 (5·33–5·72)	22	12·8 (11·8–13·4)	50	4·10 (3·56–4·38)	67
**Latin America and Caribbean**	**11·0 (10·4–11·6)**	**3**	**3·49 (3·23–3·74)**	**4**	**15·1 (14·4–15·8)**	**7**	**3·29 (3·11–3·48)**	**16**	**15·5 (14·7–16·3)**	**19**	**3·30 (3·11–3·48)**	**44**	**20·2 (18·5–21·4)**	**42**	**3·55 (3·16–3·82)**	**74**
Andean Latin America	8·46 (6·98–10·1)	3	3·96 (3·09–4·99)	4	9·49 (7·60–11·6)	9	2·41 (1·82–3·02)	19	11·3 (8·79–13·8)	19	2·32 (1·75–2·95)	43	14·1 (11·0–17·0)	44	3·37 (2·54–4·26)	70
Bolivia (Plurinational State of)	6·90 (3·98–11·0)	4	5·05 (2·75–8·47)	3	9·36 (5·23–15·3)	11	3·46 (1·89–5·60)	16	15·0 (8·50–23·3)	19	4·16 (2·18–6·96)	42	19·5 (11·5–29·1)	41	5·99 (3·31–9·72)	67
Ecuador	17·0 (14·2–20·2)	2	6·30 (5·24–7·50)	2	17·8 (14·6–21·5)	6	3·70 (2·93–4·61)	14	19·5 (15·4–24·4)	14	3·21 (2·51–4·00)	36	23·7 (19·1–28·9)	32	4·62 (3·73–5·60)	68
Peru	4·68 (3·06–6·64)	5	2·34 (1·41–3·42)	6	5·61 (3·68–7·91)	13	1·48 (0·848–2·17)	23	6·21 (4·02–8·79)	26	1·34 (0·782–2·00)	51	8·33 (5·16–11·7)	49	2·08 (1·18–3·06)	73
Caribbean	7·97 (6·42–9·74)	3	3·46 (2·37–4·77)	6	18·0 (15·3–21·2)	8	4·00 (3·14–5·01)	16	26·0 (22·8–29·7)	16	6·59 (5·69–7·62)	26	48·5 (42·6–54·6)	22	9·56 (8·28–10·9)	55
Antigua and Barbuda	1·88 (1·44–2·39)	11	0·223 (0·178–0·277)	39	2·86 (2·27–3·57)	21	0·267 (0·216–0·329)	63	4·16 (3·32–5·12)	45	0·514 (0·409–0·631)	82	8·99 (7·30–11·0)	58	0·810 (0·669–0·969)	94
Bahamas	3·21 (2·35–4·29)	8	0·498 (0·366–0·666)	29	6·82 (5·22–8·76)	18	0·762 (0·563–1·01)	52	6·43 (4·79–8·25)	38	0·774 (0·586–0·985)	82	10·7 (8·44–13·5)	59	1·14 (0·911–1·40)	92
Barbados	4·42 (3·29–5·83)	4	1·08 (0·808–1·42)	13	8·70 (6·54–11·3)	9	1·15 (0·846–1·50)	34	11·2 (8·23–14·6)	23	1·89 (1·43–2·41)	57	23·0 (17·5–29·2)	41	2·89 (2·28–3·58)	78
Belize	7·58 (6·06–9·41)	5	1·40 (1·12–1·75)	10	14·4 (11·7–17·5)	8	1·84 (1·47–2·26)	28	15·1 (12·2–18·8)	19	2·59 (2·12–3·12)	45	26·1 (21·4–31·3)	30	2·73 (2·25–3·27)	73
Bermuda	2·50 (1·97–3·21)	5	0·714 (0·516–0·931)	13	5·55 (4·36–7·01)	9	0·809 (0·610–1·03)	26	8·26 (6·54–10·4)	28	1·26 (0·981–1·62)	49	19·9 (16·2–24·3)	39	2·31 (1·80–2·90)	69
Cuba	8·31 (7·10–9·59)	3	2·27 (1·88–2·71)	3	20·5 (17·8–23·6)	3	5·04 (4·26–5·94)	7	36·3 (31·2–41·9)	9	10·4 (8·83–12·2)	17	77·3 (65·4–89·8)	17	17·0 (14·4–19·9)	36
Dominica	3·90 (2·46–5·77)	5	0·753 (0·428–1·22)	21	8·22 (5·34–12·1)	12	0·827 (0·515–1·26)	38	11·9 (7·86–17·2)	25	1·22 (0·785–1·88)	68	23·4 (16·1–32·3)	41	2·02 (1·35–2·99)	85

Dominican Republic	8·51 (4·93–12·7)	3	2·36 (1·46–3·68)	8	13·0 (8·37–19·3)	8	2·77 (1·70–4·20)	18	15·0 (9·93–22·1)	19	3·11 (1·89–4·73)	35	24·5 (16·6–35·6)	27	3·99 (2·51–6·06)	65
Grenada	3·95 (3·15–4·88)	5	1·49 (1·16–1·91)	12	9·11 (7·40–11·1)	12	1·80 (1·41–2·27)	34	12·0 (9·69–14·8)	24	2·61 (2·13–3·19)	50	26·0 (21·3–31·4)	41	3·69 (3·05–4·38)	70
Guyana	40·8 (30·9–53·0)	1	11·7 (8·30–15·8)	2	90·6 (67·4–118)	1	12·7 (8·59–17·4)	10	80·7 (57·9–110)	8	16·3 (11·8–21·7)	18	113 (83·6–147)	14	16·4 (12·6–20·8)	37
Haiti	6·24 (3·42–10·2)	5	5·56 (2·22–9·37)	8	17·9 (9·91–27·8)	8	4·89 (2·01–8·01)	19	24·6 (14·2–38·4)	21	6·85 (3·26–11·0)	37	41·9 (25·7–63·3)	28	7·78 (4·10–12·2)	65
Jamaica	1·01 (0·684–1·42)	10	0·307 (0·216–0·423)	29	3·01 (2·07–4·22)	17	0·516 (0·354–0·714)	51	4·12 (2·82–5·73)	42	0·675 (0·496–0·898)	78	8·19 (6·02–10·8)	59	0·871 (0·667–1·10)	90
Puerto Rico	5·30 (4·36–6·41)	3	1·15 (0·919–1·41)	7	16·3 (13·9–19·0)	6	1·81 (1·46–2·19)	20	17·9 (15·1–20·9)	18	2·46 (2·02–2·96)	37	22·3 (18·4–26·4)	35	3·07 (2·46–3·67)	67
Saint Kitts and Nevis	2·47 (1·62–3·77)	7	0·540 (0·396–0·712)	21	4·54 (3·07–6·53)	16	0·552 (0·387–0·746)	42	8·49 (6·38–10·8)	32	1·22 (0·936–1·54)	66	18·2 (13·9–23·0)	54	2·11 (1·73–2·55)	82
Saint Lucia	7·87 (6·09–9·93)	4	0·749 (0·574–0·960)	17	15·0 (12·0–18·6)	8	1·31 (1·00–1·69)	35	15·6 (12·1–19·9)	20	1·49 (1·15–1·88)	60	31·1 (24·9–38·1)	35	1·97 (1·57–2·41)	82
Saint Vincent and the Grenadines	5·98 (4·76–7·39)	5	0·952 (0·742–1·20)	21	14·2 (11·4–17·1)	10	1·12 (0·889–1·40)	41	16·3 (13·2–19·8)	22	1·28 (1·03–1·57)	67	30·4 (25·4–36·3)	37	1·64 (1·35–1·96)	85
Suriname	25·3 (18·3–33·2)	1	11·5 (7·76–15·9)	1	56·3 (41·1–74·0)	1	12·1 (7·86–17·7)	7	63·1 (44·0–87·2)	7	14·1 (9·18–21·7)	16	89·8 (61·8–126)	15	16·1 (10·5–25·9)	35
Trinidad and Tobago	16·8 (13·2–21·0)	3	3·74 (2·90–4·74)	4	29·1 (23·1–36·0)	4	5·23 (4·02–6·72)	12	29·9 (23·3–38·0)	11	5·75 (4·50–7·21)	28	47·8 (37·9–59·0)	21	6·68 (5·33–8·21)	54
United States Virgin Islands	19·9 (12·5–31·9)	3	1·65 (0·896–3·05)	6	17·3 (11·1–26·0)	7	2·24 (1·27–4·10)	22	11·4 (7·00–16·8)	22	2·04 (1·22–3·42)	40	19·4 (12·4–28·3)	32	2·72 (1·73–4·29)	66
Central Latin America	12·5 (11·6–13·4)	3	3·68 (3·40–3·96)	3	15·4 (14·4–16·5)	9	2·75 (2·53–2·98)	18	13·0 (11·9–14·1)	16	2·01 (1·84–2·18)	50	15·8 (14·3–17·1)	43	2·03 (1·80–2·21)	80
Colombia	12·6 (10·8–14·6)	3	4·15 (3·55–4·80)	3	13·9 (12·0–16·0)	6	2·62 (2·24–3·05)	17	13·5 (11·5–15·8)	16	1·96 (1·65–2·30)	46	15·9 (13·4–18·6)	44	2·09 (1·71–2·49)	77
Costa Rica	13·7 (11·8–15·7)	3	3·53 (2·97–4·12)	2	21·4 (18·6–24·4)	3	3·42 (2·84–4·06)	13	22·0 (18·8–25·5)	13	2·44 (2·01–2·91)	43	29·5 (24·5–34·8)	31	2·81 (2·28–3·34)	73
El Salvador	12·3 (8·44–17·3)	3	5·73 (4·00–7·69)	2	22·9 (16·6–30·7)	7	4·61 (3·20–6·30)	14	20·4 (14·8–27·3)	16	3·95 (2·60–5·51)	33	20·2 (14·6–26·8)	35	3·73 (2·35–5·13)	59

Guatemala	10·4 (8·70–12·2)	4	4·36 (3·68–5·11)	4	14·0 (11·9–16·2)	11	2·83 (2·37–3·35)	17	10·8 (9·07–12·9)	21	2·08 (1·73–2·48)	52	13·8 (11·5–16·4)	40	1·97 (1·65–2·33)	77
Honduras	4·45 (2·44–7·48)	3	1·70 (0·762–3·35)	10	8·79 (4·64–14·9)	8	1·94 (1·01–3·34)	33	18·1 (11·4–27·0)	22	2·38 (1·28–3·76)	60	25·8 (16·2–37·4)	40	2·05 (1·22–3·24)	86
Mexico	12·6 (11·7–13·5)	3	3·57 (3·29–3·86)	4	15·7 (14·5–16·9)	8	2·73 (2·49–3·00)	17	11·3 (10·3–12·4)	20	1·79 (1·63–1·97)	54	12·9 (11·7–14·1)	46	1·76 (1·59–1·92)	79
Nicaragua	10·0 (7·40–12·8)	3	4·43 (3·09–5·97)	1	14·7 (11·0–19·2)	8	3·17 (2·20–4·38)	11	14·7 (10·7–19·6)	16	2·78 (1·83–3·93)	34	18·3 (13·0–24·9)	30	2·48 (1·55–3·60)	66
Panama	8·49 (6·96–10·1)	4	2·56 (2·10–3·07)	5	11·4 (9·41–13·5)	6	1·98 (1·61–2·41)	18	12·9 (10·5–15·5)	16	1·48 (1·21–1·80)	52	20·1 (16·3–24·0)	36	1·64 (1·31–1·97)	80
Venezuela (Bolivarian Republic of)	17·7 (13·4–22·8)	3	3·24 (2·49–4·17)	4	17·2 (13·3–21·9)	6	2·76 (2·09–3·59)	19	15·4 (11·6–20·1)	17	2·39 (1·83–3·06)	44	22·0 (17·1–27·7)	40	2·43 (1·91–3·00)	72
Tropical Latin America	10·7 (10·1–11·4)	3	3·10 (2·91–3·29)	3	15·7 (14·9–16·5)	7	3·94 (3·75–4·15)	13	16·7 (15·9–17·6)	21	4·13 (3·87–4·35)	36	19·3 (17·5–20·6)	46	3·67 (3·16–3·98)	72
Brazil	10·8 (10·2–11·5)	3	3·07 (2·90–3·27)	3	15·9 (15·1–16·7)	7	3·96 (3·76–4·17)	13	16·8 (15·9–17·7)	21	4·13 (3·87–4·35)	36	19·2 (17·5–20·5)	46	3·65 (3·14–3·96)	72
Paraguay	9·47 (5·93–13·1)	3	3·65 (2·27–5·04)	3	10·4 (7·35–14·1)	8	3·48 (2·29–4·97)	13	15·3 (10·6–21·1)	20	4·20 (2·75–5·96)	32	22·6 (15·9–30·8)	41	4·67 (3·10–6·56)	68
**North Africa and Middle East**	**4·93 (4·01–5·82)**	**5**	**2·47 (1·86–3·08)**	**6**	**7·34 (6·08–8·73)**	**7**	**2·72 (2·03–3·35)**	**16**	**6·19 (4·87–7·38)**	**28**	**1·93 (1·35–2·35)**	**44**	**7·66 (5·90–9·15)**	**49**	**2·92 (1·99–3·61)**	**72**
North Africa and Middle East	4·93 (4·01–5·82)	5	2·47 (1·86–3·08)	6	7·34 (6·08–8·73)	7	2·72 (2·03–3·35)	16	6·19 (4·87–7·38)	28	1·93 (1·35–2·35)	44	7·66 (5·90–9·15)	49	2·92 (1·99–3·61)	72
Afghanistan	4·25 (2·41–7·28)	11	2·99 (1·54–7·77)	17	9·69 (5·87–16·5)	13	6·69 (3·50–12·5)	23	8·77 (5·19–13·9)	29	7·15 (3·73–11·0)	33	17·3 (9·28–25·2)	39	7·98 (3·31–12·1)	54
Algeria	4·16 (2·51–6·39)	2	2·47 (1·28–3·88)	4	5·29 (3·42–7·70)	5	2·86 (1·47–4·45)	13	4·03 (2·54–6·14)	25	1·54 (0·770–2·42)	41	5·75 (3·66–8·74)	51	2·78 (1·29–4·58)	65
Bahrain	6·80 (4·43–9·46)	2	1·67 (1·13–2·53)	5	7·16 (5·04–9·72)	5	1·64 (1·11–2·58)	17	5·60 (3·90–8·09)	24	1·31 (0·856–2·28)	53	14·2 (10·2–19·9)	47	3·17 (2·18–5·07)	73
Egypt	1·71 (1·12–2·43)	16	0·610 (0·398–0·899)	17	2·20 (1·51–3·07)	22	0·675 (0·455–0·948)	33	2·24 (1·63–3·08)	39	0·754 (0·518–1·06)	61	3·04 (2·16–4·08)	61	1·59 (1·06–2·24)	80
Iran (Islamic Republic of)	8·39 (6·94–9·42)	2	3·64 (3·04–4·47)	2	9·08 (7·71–10·3)	5	2·61 (2·20–3·68)	9	6·43 (5·32–7·37)	25	1·70 (1·44–2·52)	37	7·25 (5·99–8·40)	50	3·18 (2·58–4·34)	63

Iraq	5·77 (3·58–8·84)	6	2·67 (1·61–4·25)	5	10·9 (6·88–16·7)	7	3·69 (2·24–5·75)	14	13·2 (7·72–19·8)	20	2·62 (1·56–4·07)	36	13·9 (8·59–19·9)	34	2·73 (1·73–4·07)	65
Jordan	1·63 (1·08–2·36)	10	0·533 (0·348–0·808)	15	2·44 (1·68–3·38)	16	0·615 (0·406–0·913)	25	1·92 (1·29–2·71)	37	0·534 (0·352–0·813)	61	2·58 (1·78–3·56)	59	0·980 (0·668–1·41)	78
Kuwait	3·19 (2·58–3·90)	3	0·869 (0·705–1·06)	5	3·61 (2·97–4·34)	7	0·839 (0·688–1·01)	13	3·36 (2·71–4·13)	22	0·715 (0·580–0·868)	45	5·82 (4·65–7·06)	47	1·48 (1·12–1·81)	67
Lebanon	1·14 (0·726–1·68)	14	0·843 (0·519–1·27)	9	1·58 (1·05–2·27)	21	0·857 (0·552–1·30)	27	1·36 (0·921–1·94)	58	0·609 (0·395–0·908)	68	2·31 (1·56–3·23)	82	1·20 (0·761–1·80)	79
Libya	7·53 (4·44–11·5)	5	3·87 (1·96–6·23)	5	10·8 (6·63–16·4)	6	4·55 (2·34–7·51)	13	7·87 (4·48–13·7)	30	2·89 (1·41–5·70)	41	8·33 (5·00–15·6)	47	3·63 (1·49–8·69)	64
Morocco	3·87 (2·18–6·44)	2	3·47 (1·73–6·01)	3	5·58 (3·19–9·30)	6	4·42 (2·17–7·31)	13	6·59 (3·80–10·3)	21	3·60 (1·62–6·01)	29	8·44 (5·14–12·6)	43	3·73 (1·74–5·92)	56
Oman	1·99 (1·27–3·02)	9	0·499 (0·294–0·784)	17	2·15 (1·37–3·30)	14	0·479 (0·286–0·739)	27	2·23 (1·44–3·40)	42	0·503 (0·312–0·771)	71	3·87 (2·56–5·50)	65	0·742 (0·474–1·12)	81
Palestine	1·48 (0·949–2·15)	17	0·395 (0·247–0·608)	20	1·79 (1·21–2·56)	17	0·435 (0·280–0·654)	33	1·94 (1·29–2·72)	44	0·427 (0·283–0·634)	67	2·83 (1·90–3·89)	65	0·838 (0·556–1·21)	89
Qatar	6·96 (4·74–10·0)	2	1·18 (0·726–1·88)	3	6·59 (4·47–9·52)	4	1·02 (0·618–1·74)	11	5·36 (3·36–8·55)	19	1·20 (0·730–1·99)	47	10·4 (6·57–16·1)	43	3·01 (1·88–4·61)	68
Saudi Arabia	8·49 (5·14–13·3)	3	2·97 (1·75–4·80)	5	15·1 (9·09–23·6)	6	5·66 (3·41–8·74)	10	13·9 (9·08–20·5)	14	3·70 (2·33–5·75)	33	13·0 (9·02–18·5)	38	3·02 (1·92–4·95)	63
Sudan	6·23 (3·19–10·3)	3	4·93 (1·93–9·30)	5	10·2 (5·38–16·3)	7	4·18 (1·59–7·64)	14	7·18 (4·21–11·4)	29	2·57 (1·20–4·42)	41	7·52 (4·66–11·2)	49	3·08 (1·48–4·97)	67
Syrian Arab Republic	1·21 (0·710–1·90)	17	0·438 (0·274–0·701)	27	1·98 (1·26–2·95)	23	0·479 (0·288–0·769)	40	2·03 (1·27–3·08)	40	0·397 (0·240–0·615)	67	2·35 (1·56–3·34)	65	0·616 (0·404–0·930)	90
Tunisia	4·33 (2·46–7·30)	3	2·05 (1·05–3·42)	2	6·37 (3·70–10·1)	7	2·30 (1·15–3·88)	11	4·90 (2·68–8·55)	29	1·66 (0·745–3·13)	41	7·26 (4·08–14·4)	50	2·93 (1·24–5·66)	60
Türkiye	6·46 (4·49–8·65)	2	2·20 (1·34–3·01)	2	7·36 (5·33–9·83)	6	1·75 (1·14–2·46)	15	7·04 (4·96–9·69)	26	1·60 (1·02–2·24)	41	9·64 (6·83–13·1)	48	2·95 (1·95–4·17)	72
United Arab Emirates	6·75 (4·40–10·2)	2	2·63 (1·58–4·29)	3	3·03 (1·95–4·67)	6	1·48 (0·910–2·38)	14	3·13 (1·97–4·92)	29	1·53 (0·942–2·34)	44	6·32 (4·21–9·50)	54	23·5 (14·5–36·7)	66
Yemen	3·74 (1·31–7·27)	5	2·84 (0·750–5·33)	6	9·85 (4·98–16·2)	7	4·11 (1·39–7·28)	12	9·18 (5·03–14·9)	29	3·36 (1·49–5·68)	37	9·35 (5·50–14·2)	46	3·83 (1·76–6·41)	62

**South Asia**	**12·5 (10·8–14·2)**	**2**	**11·6 (9·49–13·6)**	**1**	**21·4 (17·8–24·3)**	**6**	**10·4 (7·15–12·3)**	**6**	**20·3 (15·9–23·3)**	**18**	**10·5 (6·76–12·3)**	**26**	**28·3 (21·8–32·5)**	**30**	**14·8 (9·98–17·4)**	**40**
South Asia	12·5 (10·8–14·2)	2	11·6 (9·49–13·6)	1	21·4 (17·8–24·3)	6	10·4 (7·15–12·3)	6	20·3 (15·9–23·3)	18	10·5 (6·76–12·3)	26	28·3 (21·8–32·5)	30	14·8 (9·98–17·4)	40
Bangladesh	5·99 (3·78–8·99)	2	3·63 (2·08–7·23)	6	6·62 (4·33–9·69)	8	2·22 (1·29–4·38)	15	6·14 (3·99–9·18)	29	2·00 (1·19–4·10)	46	10·0 (6·64–14·6)	47	2·62 (1·59–5·14)	77
Bhutan	5·54 (3·13–10·7)	4	2·83 (1·25–7·22)	6	8·83 (5·05–14·4)	7	4·07 (2·34–7·59)	12	13·2 (8·05–20·0)	21	7·39 (3·87–12·0)	27	29·3 (18·4–41·5)	33	14·9 (7·10–23·3)	43
India	13·5 (11·4–15·3)	2	13·8 (10·7–16·2)	1	23·8 (19·1–27·3)	6	12·4 (7·98–14·8)	3	22·8 (17·2–26·4)	18	12·2 (7·53–14·5)	19	31·5 (23·5–36·5)	28	16·9 (11·0–19·9)	38
Nepal	17·0 (11·0–25·5)	1	11·1 (6·28–17·1)	1	25·9 (16·2–39·4)	4	9·55 (4·97–15·5)	8	28·4 (17·6–41·9)	11	9·38 (4·39–16·5)	25	55·4 (26·4–83·2)	22	13·6 (6·33–24·3)	49
Pakistan	10·2 (5·85–16·6)	2	4·82 (2·34–10·5)	7	14·0 (7·90–24·1)	7	3·60 (1·99–6·48)	16	10·7 (5·96–19·1)	29	2·77 (1·70–5·00)	57	15·4 (9·14–26·4)	42	4·11 (2·71–7·93)	77
**Southeast Asia, East Asia, and Oceania**	**4·99 (4·33–5·78)**	**3**	**2·46 (2·02–3·49)**	**2**	**8·78 (7·42–10·5)**	**7**	**4·22 (3·36–5·92)**	**8**	**13·7 (10·9–17·0)**	**19**	**8·71 (6·73–11·3)**	**20**	**42·9 (34·5–52·2)**	**24**	**26·1 (19·9–33·5)**	**26**
East Asia	4·61 (3·76–5·66)	3	2·83 (2·20–4·06)	2	8·44 (6·73–10·7)	8	5·00 (3·80–7·14)	6	14·4 (11·0–18·6)	15	10·3 (7·83–13·7)	16	48·2 (38·2–59·5)	20	30·9 (23·4–40·1)	21
China	4·48 (3·60–5·55)	3	2·75 (2·11–3·97)	2	8·14 (6·39–10·4)	8	4·81 (3·58–6·91)	6	14·0 (10·5–18·3)	15	10·2 (7·66–13·7)	16	48·2 (37·9–59·8)	19	31·2 (23·4–40·5)	21
Democratic People's Republic of Korea	7·02 (3·97–11·9)	4	4·74 (2·47–8·33)	3	12·1 (6·75–20·8)	7	9·46 (5·03–17·8)	7	21·4 (12·6–35·2)	16	14·2 (7·71–26·6)	16	40·2 (26·2–68·7)	23	26·1 (15·0–52·6)	23
Taiwan (Province of China)	9·31 (8·11–10·5)	2	4·73 (4·21–5·31)	1	22·8 (20·1–25·6)	2	11·4 (10·1–12·8)	2	31·2 (27·3–35·5)	11	12·2 (10·5–14·0)	11	51·1 (43·8–59·0)	23	23·1 (19·0–27·0)	29
Oceania	7·57 (5·96–10·1)	6	4·11 (2·92–7·04)	9	11·2 (8·67–14·1)	14	3·03 (2·22–4·31)	22	9·38 (7·07–11·8)	27	4·53 (3·34–6·07)	32	12·3 (9·42–15·2)	42	6·07 (4·71–7·98)	57
American Samoa	14·5 (9·42–20·5)	1	5·39 (3·29–8·26)	1	17·6 (11·1–25·7)	8	4·02 (2·46–6·02)	20	10·8 (7·34–15·3)	24	4·43 (2·74–6·54)	33	14·7 (10·1–20·2)	44	7·45 (4·64–10·7)	51
Cook Islands	19·1 (13·2–26·9)	2	6·41 (4·12–9·33)	2	26·2 (16·6–39·3)	4	4·80 (2·95–7·53)	9	18·4 (12·4–26·2)	13	5·31 (3·34–8·16)	18	27·4 (19·1–38·2)	27	11·7 (7·33–17·5)	29
Fiji	16·0 (10·2–23·4)	1	8·79 (5·36–13·4)	1	16·0 (10·1–23·9)	6	4·79 (2·85–7·55)	14	13·9 (8·47–21·2)	21	6·26 (3·76–9·74)	24	20·0 (13·1–28·5)	39	8·68 (5·37–13·0)	50

Guam	40·8 (33·2–48·6)	1	9·70 (7·07–12·2)	1	47·7 (36·2–59·5)	2	7·22 (4·99–9·65)	8	24·1 (17·6–31·9)	12	6·74 (4·58–9·19)	20	22·1 (16·0–29·7)	26	7·41 (4·75–10·3)	38
Kiribati	41·7 (26·6–59·7)	1	8·61 (5·14–13·5)	3	46·6 (27·7–69·1)	6	4·46 (2·56–7·20)	19	26·2 (16·3–38·7)	19	4·70 (2·80–7·36)	31	29·1 (18·5–42·4)	33	6·93 (4·25–10·3)	51
Marshall Islands	35·9 (19·3–53·0)	1	16·6 (7·48–25·7)	2	44·0 (21·9–68·7)	4	11·0 (4·54–18·1)	12	26·2 (14·2–40·8)	17	10·4 (4·54–16·7)	23	27·0 (15·9–40·7)	29	13·1 (6·27–20·3)	42
Micronesia (Federated States of)	37·1 (22·0–52·8)	1	13·6 (7·62–20·8)	1	46·9 (26·1–71·6)	3	9·16 (5·08–14·5)	13	27·5 (16·4–42·0)	17	9·72 (5·49–15·3)	24	32·0 (20·3–46·6)	31	14·4 (8·49–21·8)	42
Nauru	45·4 (22·8–66·7)	2	18·6 (8·97–28·9)	1	58·1 (26·5–88·0)	4	13·0 (5·72–21·7)	12	36·4 (17·2–56·5)	14	12·5 (6·03–20·2)	23	36·3 (19·6–53·3)	31	15·9 (7·81–24·9)	38
Niue	25·6 (15·4–38·1)	1	14·3 (8·75–22·1)	1	24·5 (13·5–40·9)	4	5·41 (3·10–9·01)	9	18·4 (11·0–27·5)	15	6·03 (3·42–9·52)	26	28·9 (18·1–41·3)	34	11·8 (7·26–17·7)	43
Northern Mariana Islands	22·6 (16·3–31·0)	1	6·47 (4·00–9·70)	2	31·6 (20·9–46·5)	2	5·79 (3·37–8·72)	11	28·0 (19·2–40·0)	14	7·33 (4·36–11·1)	27	39·0 (27·1–54·4)	26	12·1 (7·36–17·9)	47
Palau	20·2 (13·2–28·9)	2	29·9 (19·7–45·4)	1	25·3 (16·5–37·4)	7	6·62 (4·06–10·3)	12	12·3 (7·93–18·4)	21	7·32 (4·46–11·3)	21	12·3 (8·14–17·4)	37	20·5 (13·1–31·3)	35
Papua New Guinea	2·55 (1·43–6·31)	19	2·29 (1·16–6·03)	18	5·14 (3·17–8·36)	23	1·89 (1·14–3·38)	26	5·55 (3·44–8·25)	32	3·26 (1·99–5·23)	38	7·66 (5·02–11·1)	51	3·56 (2·27–5·93)	64
Samoa	22·7 (14·6–32·8)	1	10·6 (6·27–16·6)	1	27·5 (16·0–43·0)	4	7·89 (4·56–12·4)	13	18·1 (11·7–26·0)	16	8·78 (5·26–13·4)	26	26·3 (17·7–37·1)	34	13·5 (8·25–20·5)	44
Solomon Islands	34·9 (21·4–50·7)	1	13·8 (7·57–21·9)	2	48·2 (27·4–73·0)	6	10·5 (5·38–16·9)	13	26·9 (15·9–42·1)	15	10·7 (5·68–17·5)	23	27·9 (17·9–40·3)	28	13·6 (7·67–21·1)	38
Tokelau	24·1 (15·7–34·1)	1	15·1 (8·87–24·0)	1	21·3 (12·4–35·7)	4	6·81 (3·56–11·2)	11	14·9 (9·08–23·6)	17	6·87 (3·54–11·6)	26	24·0 (15·4–35·3)	36	11·0 (6·10–17·6)	44
Tonga	7·52 (4·45–11·6)	3	4·37 (2·52–7·02)	3	9·35 (5·58–14·6)	11	3·68 (2·16–5·83)	14	7·05 (4·44–10·5)	31	4·45 (2·70–6·84)	31	11·7 (7·88–16·8)	50	8·47 (5·22–12·8)	48
Tuvalu	30·3 (18·7–43·7)	2	12·6 (7·38–19·8)	1	36·0 (21·9–54·3)	4	8·86 (4·94–14·4)	13	22·1 (13·5–34·2)	17	9·76 (5·54–16·1)	23	26·7 (17·5–38·9)	32	13·7 (8·17–21·6)	42
Vanuatu	33·5 (21·2–47·1)	1	11·9 (7·03–18·3)	1	41·4 (24·6–61·8)	5	7·84 (4·49–12·4)	15	24·5 (15·4–35·8)	18	8·42 (4·91–13·4)	25	27·2 (17·9–39·3)	29	11·8 (6·95–17·9)	41
Southeast Asia	5·53 (4·67–6·44)	4	1·87 (1·53–2·60)	9	9·53 (7·85–11·5)	10	2·50 (2·00–3·36)	20	11·4 (9·24–13·6)	21	3·78 (2·68–4·81)	34	20·6 (17·0–24·1)	37	7·93 (5·83–9·96)	52

Cambodia	5·07 (2·93–8·10)	9	2·42 (1·29–4·45)	10	9·56 (5·77–15·2)	10	3·15 (1·88–5·05)	20	12·8 (7·96–19·4)	23	5·19 (2·72–8·08)	34	20·5 (13·6–29·4)	38	7·25 (4·28–10·9)	50
Indonesia	2·45 (1·73–3·38)	11	0·849 (0·568–1·60)	18	1·92 (1·36–3·27)	26	1·04 (0·744–1·72)	33	3·37 (2·47–4·39)	40	1·67 (1·13–2·26)	48	8·68 (5·76–10·7)	47	5·37 (2·09–7·02)	55
Lao People's Democratic Republic	8·07 (4·82–12·8)	3	4·16 (2·23–6·95)	8	10·7 (6·37–17·3)	9	3·16 (1·74–5·19)	23	12·0 (7·39–18·3)	22	3·51 (1·96–5·70)	38	18·7 (12·0–29·5)	33	5·20 (3·09–8·39)	58
Malaysia	4·72 (3·12–6·90)	2	1·36 (0·865–2·35)	5	9·96 (6·88–13·8)	8	2·43 (1·62–3·60)	16	14·7 (10·4–19·8)	17	3·92 (2·64–5·56)	40	21·6 (15·4–29·5)	37	7·06 (4·66–10·2)	58
Maldives	3·32 (2·21–4·85)	4	0·453 (0·280–0·697)	17	3·91 (2·56–5·85)	8	0·515 (0·320–0·807)	31	3·69 (2·44–5·38)	23	0·643 (0·411–0·963)	59	11·8 (7·98–16·5)	42	1·59 (1·02–2·33)	75
Mauritius	14·8 (12·4–17·4)	2	4·20 (3·50–4·96)	2	22·5 (19·1–26·1)	8	4·44 (3·67–5·31)	11	19·1 (16·0–22·5)	15	4·33 (3·62–5·16)	25	31·2 (26·0–37·2)	27	7·05 (5·75–8·39)	51
Myanmar	5·44 (3·32–8·21)	9	1·32 (0·753–2·19)	19	8·56 (5·13–13·0)	14	1·42 (0·851–2·27)	28	9·07 (5·32–13·2)	25	1·92 (0·990–3·06)	49	16·5 (9·27–23·8)	35	2·85 (1·66–4·29)	69
Philippines	6·19 (4·48–7·14)	5	1·73 (1·21–2·06)	13	8·86 (7·53–10·1)	13	1·78 (1·43–2·18)	23	8·22 (6·91–9·69)	25	1·78 (1·41–2·18)	45	10·9 (9·40–13·3)	47	2·61 (2·15–3·50)	67
Seychelles	9·99 (6·44–14·0)	3	1·25 (0·835–1·81)	13	14·4 (10·2–19·7)	8	1·52 (1·04–2·16)	27	17·0 (11·9–22·8)	24	1·35 (0·923–1·93)	49	28·3 (20·1–37·7)	38	2·73 (1·91–3·82)	68
Sri Lanka	17·9 (11·3–25·6)	1	8·66 (5·82–12·2)	1	33·4 (21·7–47·6)	2	6·78 (4·19–10·6)	5	38·0 (23·8–57·5)	9	6·26 (3·69–11·4)	21	71·9 (45·6–105)	15	15·0 (9·59–24·9)	29
Thailand	14·9 (10·9–20·1)	2	2·83 (1·93–4·65)	3	28·6 (20·5–37·8)	6	5·30 (3·62–7·27)	12	19·8 (14·0–26·7)	18	5·25 (3·38–7·19)	25	25·5 (18·3–34·3)	34	7·40 (5·05–10·8)	52
Timor-Leste	6·06 (3·64–9·43)	4	3·32 (1·85–5·47)	6	7·96 (4·60–13·1)	10	2·52 (1·43–4·02)	19	9·67 (5·78–15·7)	21	4·11 (2·43–6·46)	37	16·1 (9·90–26·7)	32	5·79 (3·60–8·95)	54
Viet Nam	6·41 (3·87–9·82)	4	3·26 (2·00–5·07)	2	14·1 (8·54–22·8)	6	4·73 (2·93–7·45)	9	20·5 (12·5–30·7)	17	9·03 (4·91–14·1)	20	35·9 (24·1–50·4)	25	18·1 (11·0–27·4)	30
**Sub-Saharan Africa**	**7·25 (6·09–8·81)**	**7**	**2·22 (1·55–2·89)**	**13**	**18·1 (15·2–21·6)**	**11**	**3·69 (2·78–4·66)**	**20**	**37·7 (31·7–44·3)**	**15**	**10·7 (8·26–12·9)**	**27**	**91·5 (75·9–103)**	**18**	**24·8 (19·5–29·1)**	**37**
Central Sub-Saharan Africa	7·38 (4·88–11·5)	9	2·53 (1·52–4·26)	13	20·5 (14·0–29·8)	10	4·22 (2·73–6·57)	20	51·9 (36·0–73·5)	15	15·1 (9·38–22·7)	24	122 (85·9–172)	16	29·7 (19·2–44·1)	35
Angola	7·12 (4·22–11·3)	5	2·25 (1·19–4·08)	12	19·9 (12·4–30·2)	10	3·63 (2·07–5·98)	18	50·5 (32·4–75·9)	15	12·0 (6·99–19·1)	25	124 (81·8–176)	17	25·4 (15·3–38·8)	36

Central African Republic	10·5 (5·86–17·4)	10	3·39 (1·80–5·89)	14	33·3 (18·4–54·8)	12	6·26 (3·26–10·6)	20	79·1 (48·1–121)	15	19·3 (10·1–30·9)	26	145 (95·3–214)	18	34·5 (19·0–55·1)	35
Congo	8·09 (4·69–13·3)	5	2·90 (1·61–4·81)	12	20·0 (12·0–32·5)	10	5·75 (2·97–9·47)	18	44·3 (27·9–65·7)	16	16·6 (8·84–25·7)	24	107 (72·3–151)	18	31·2 (18·9–46·7)	35
Democratic Republic of the Congo	7·20 (4·19–12·4)	8	2·58 (1·36–4·76)	12	19·9 (11·9–32·1)	8	4·21 (2·38–7·37)	20	51·3 (31·8–81·2)	14	15·9 (8·87–26·3)	23	121 (76·6–194)	15	30·8 (17·9–50·7)	33
Equatorial Guinea	7·56 (3·99–12·9)	4	1·99 (0·751–4·58)	11	17·1 (9·90–28·0)	9	3·43 (1·53–6·91)	17	41·1 (24·6–65·7)	15	10·5 (5·04–19·3)	26	108 (70·9–160)	19	23·4 (12·3–39·3)	38
Gabon	9·10 (4·93–14·9)	5	1·65 (0·752–3·53)	14	22·5 (12·9–37·0)	9	3·33 (1·75–6·12)	20	50·9 (31·9–76·6)	16	10·4 (5·64–17·1)	28	124 (82·2–184)	19	25·7 (14·9–39·3)	42
Eastern Sub-Saharan Africa	6·75 (5·48–8·49)	9	2·35 (1·72–3·03)	14	16·3 (13·2–20·3)	12	3·44 (2·65–4·37)	20	40·2 (33·4–48·6)	14	11·6 (9·20–14·3)	28	108 (92·3–125)	18	29·2 (23·6–36·0)	36
Burundi	7·06 (4·03–12·8)	7	3·19 (1·67–5·48)	10	16·5 (9·83–29·0)	11	3·74 (2·06–6·08)	19	43·3 (26·9–69·5)	14	12·9 (7·39–21·6)	24	117 (78·2–174)	19	32·5 (19·6–52·4)	30
Comoros	3·89 (1·89–7·63)	11	3·17 (1·74–5·12)	11	8·84 (4·55–17·0)	14	3·99 (2·32–6·26)	18	20·8 (10·7–39·6)	21	12·1 (7·32–19·3)	28	60·6 (33·0–111)	27	30·1 (18·4–47·2)	36
Djibouti	4·37 (1·98–9·09)	10	1·91 (0·820–3·67)	12	9·51 (4·23–19·9)	15	3·23 (1·63–5·68)	19	23·4 (11·0–47·0)	23	11·3 (5·92–19·0)	27	61·7 (32·0–120)	26	30·1 (17·5–46·9)	36
Eritrea	9·62 (5·31–16·1)	6	2·97 (1·58–4·94)	11	23·0 (12·7–38·7)	11	5·72 (3·19–9·46)	18	51·9 (31·5–79·8)	12	17·9 (10·4–27·4)	22	123 (81·3–179)	18	41·9 (26·5–62·5)	30
Ethiopia	5·87 (4·38–7·64)	6	2·27 (1·51–3·26)	12	13·1 (9·90–17·1)	11	3·06 (2·20–4·23)	20	33·3 (24·7–43·7)	15	10·8 (7·80–14·8)	28	103 (79·4–130)	18	28·6 (20·9–38·2)	33
Kenya	5·68 (4·04–8·70)	6	1·84 (1·18–2·84)	15	13·9 (10·0–20·3)	9	3·52 (2·35–5·20)	20	40·4 (29·2–56·9)	13	11·6 (8·00–17·1)	27	111 (82·5–151)	17	31·9 (23·0–46·2)	33
Madagascar	6·51 (3·65–10·9)	7	3·26 (1·73–5·35)	13	13·4 (7·87–21·9)	11	4·29 (2·44–6·84)	20	32·8 (19·8–51·0)	14	12·2 (7·04–19·1)	26	85·1 (53·4–127)	20	27·4 (16·8–41·2)	35
Malawi	9·56 (5·79–15·1)	6	2·44 (1·25–4·40)	14	23·3 (14·6–35·4)	11	3·45 (1·98–5·70)	22	56·2 (36·7–81·7)	15	11·6 (7·02–18·6)	25	132 (91·0–186)	19	28·6 (18·0–43·6)	32
Mozambique	11·2 (6·30–18·3)	6	2·69 (1·38–4·64)	11	30·8 (17·9–48·3)	8	3·95 (2·19–6·65)	16	69·8 (44·1–102)	16	12·5 (7·26–19·5)	25	169 (114–243)	17	31·7 (19·3–48·4)	37
Rwanda	6·68 (3·87–10·9)	5	2·59 (1·32–4·78)	11	15·3 (8·98–25·4)	8	3·58 (1·96–6·26)	19	40·2 (24·7–61·5)	13	12·3 (6·83–21·1)	28	114 (74·7–169)	17	32·9 (18·4–54·5)	34

Somalia	4·88 (1·95–11·2)	17	3·53 (1·80–6·61)	15	14·5 (6·24–32·0)	16	5·98 (2·99–10·7)	19	42·7 (20·2–87·1)	23	22·9 (12·5–40·3)	26	89·7 (45·9–176)	27	45·4 (26·5–82·5)	31
South Sudan	6·59 (3·62–10·7)	11	3·03 (1·53–5·35)	16	18·6 (10·9–29·7)	12	4·26 (2·30–7·05)	19	47·6 (28·0–75·5)	15	14·2 (8·41–22·7)	26	121 (75·3–187)	19	33·1 (19·8–50·4)	34
Uganda	7·08 (4·23–10·7)	6	1·67 (0·849–2·84)	15	17·6 (11·0–26·4)	10	2·20 (1·26–3·41)	22	44·0 (28·1–64·6)	16	7·68 (4·63–12·0)	31	120 (82·2–168)	19	23·1 (14·7–35·3)	42
United Republic of Tanzania	5·58 (3·35–8·61)	7	1·93 (1·04–3·36)	16	13·6 (8·37–20·6)	11	2·72 (1·61–4·44)	21	30·4 (19·3–46·0)	15	9·57 (5·91–14·8)	30	84·0 (57·2–121)	19	25·4 (16·1–38·1)	40
Zambia	9·46 (5·65–15·0)	6	2·57 (1·38–4·36)	14	23·4 (14·1–35·7)	11	3·89 (2·19–6·48)	21	51·2 (33·3–74·2)	17	11·5 (6·71–18·1)	27	128 (88·3–178)	20	27·9 (17·4–42·3)	40
Southern Sub-Saharan Africa	22·2 (18·3–27·5)	4	4·59 (2·90–6·34)	8	40·5 (33·3–49·3)	7	8·89 (6·62–11·7)	16	39·0 (33·2–45·7)	18	10·3 (8·23–12·7)	27	42·5 (36·6–49·1)	30	16·9 (13·5–21·0)	44
Botswana	12·0 (6·97–21·1)	4	3·51 (1·79–7·56)	9	24·5 (14·5–40·1)	5	4·89 (2·65–9·42)	14	31·6 (19·7–48·1)	17	7·18 (4·28–11·9)	27	45·6 (29·5–66·2)	28	14·4 (8·83–22·4)	45
Eswatini	26·9 (15·3–44·1)	4	5·66 (2·33–10·7)	9	63·7 (36·2–99·9)	6	9·34 (4·20–17·3)	15	73·5 (44·8–112)	16	13·4 (6·61–23·2)	26	71·0 (45·4–104)	27	20·5 (11·6–32·3)	41
Lesotho	24·3 (13·6–42·4)	5	7·18 (3·40–12·9)	9	58·9 (35·2–94·2)	7	13·1 (5·89–24·1)	13	80·5 (50·8–121)	17	20·9 (9·66–37·2)	22	81·3 (53·9–119)	24	32·6 (15·4–55·7)	37
Namibia	15·2 (8·59–25·8)	4	2·92 (1·33–6·74)	9	35·3 (19·9–57·9)	7	4·84 (2·41–10·0)	17	48·2 (29·9–72·4)	15	7·80 (4·20–13·3)	27	58·6 (35·2–84·0)	24	14·5 (8·28–23·8)	47
South Africa	23·2 (19·1–27·9)	4	3·09 (2·15–4·25)	9	37·9 (31·7–45·9)	6	7·99 (6·13–11·2)	17	33·1 (28·1–40·5)	18	8·57 (7·05–12·0)	28	33·7 (29·3–40·1)	32	14·3 (11·5–19·3)	49
Zimbabwe	21·0 (12·0–35·4)	5	9·21 (4·18–15·2)	5	54·0 (30·8–85·6)	5	14·0 (5·86–23·6)	9	69·1 (40·8–102)	14	20·1 (8·23–32·7)	25	97·5 (57·0–138)	27	36·6 (15·9–57·0)	41
Western Sub-Saharan Africa	5·30 (4·03–6·70)	8	1·67 (1·02–2·25)	18	13·5 (10·5–16·5)	12	2·56 (1·68–3·38)	21	31·0 (23·3–38·2)	16	8·79 (5·95–11·4)	26	85·3 (61·6–102)	18	22·7 (16·0–28·0)	37
Benin	5·67 (3·16–9·78)	8	1·91 (0·888–3·54)	15	14·7 (8·70–24·3)	11	2·83 (1·46–4·88)	20	33·5 (20·8–53·4)	16	9·82 (5·22–15·8)	25	82·1 (53·5–121)	21	22·9 (12·9–34·9)	38
Burkina Faso	5·83 (3·43–9·15)	9	2·44 (1·28–4·25)	17	16·7 (10·5–25·5)	12	3·32 (1·96–5·41)	21	46·2 (30·1–69·0)	15	11·8 (7·19–18·9)	24	118 (78·7–170)	21	28·9 (18·1–43·6)	33
Cabo Verde	13·4 (8·31–19·6)	2	1·61 (0·896–2·71)	6	33·9 (21·0–50·4)	2	4·30 (2·07–6·85)	10	60·5 (38·2–87·6)	8	10·3 (5·97–15·9)	19	102 (61·6–164)	21	21·7 (13·5–35·6)	36

Cameroon	7·94 (4·60–13·1)	5	2·33 (1·07–4·15)	17	19·9 (11·7–31·6)	9	3·64 (1·79–6·15)	20	41·4 (24·6–65·3)	15	11·8 (6·00–19·4)	21	92·9 (58·9–138)	21	26·9 (14·4–43·1)	36
Chad	5·62 (3·00–10·1)	12	2·36 (1·16–4·16)	14	14·6 (8·16–25·0)	12	4·23 (2·28–6·92)	19	34·7 (19·3–63·2)	17	16·6 (9·05–26·1)	22	77·0 (44·4–147)	23	34·5 (17·6–56·7)	31
Côte d'Ivoire	7·31 (4·28–12·0)	5	2·04 (0·923–3·74)	16	17·9 (10·8–27·9)	11	2·73 (1·33–4·73)	19	43·6 (26·9–68·1)	16	8·74 (4·48–14·3)	23	112 (73·2–162)	17	20·1 (10·9–31·5)	38
Gambia	5·99 (2·97–11·4)	8	2·22 (1·05–4·02)	14	12·9 (6·66–24·4)	13	3·17 (1·83–5·21)	21	27·1 (14·1–49·9)	18	12·9 (7·84–20·1)	24	63·8 (34·9–113)	26	33·2 (20·2–51·7)	32
Ghana	5·94 (3·57–9·49)	9	1·87 (1·03–3·23)	18	13·9 (8·60–20·8)	11	2·65 (1·59–4·23)	23	31·7 (20·5–46·8)	16	7·67 (4·70–11·9)	31	78·8 (51·2–115)	22	17·9 (11·3–27·4)	43
Guinea	4·65 (2·33–8·61)	11	2·67 (1·33–4·62)	16	11·6 (6·11–21·6)	13	3·84 (2·06–6·26)	21	26·0 (13·9–46·8)	21	13·6 (7·53–21·7)	23	58·7 (32·9–100)	29	28·2 (16·2–43·8)	33
Guinea-Bissau	9·61 (5·50–16·1)	6	3·50 (1·80–5·79)	14	24·4 (14·2–39·2)	11	6·42 (3·53–10·3)	21	50·5 (30·2–77·9)	16	18·3 (10·3–28·3)	23	96·9 (59·6–141)	21	34·7 (20·8–51·9)	34
Liberia	6·02 (3·40–10·6)	5	3·14 (1·41–6·01)	13	15·0 (8·91–24·6)	11	4·36 (2·02–8·32)	19	29·2 (17·3–45·5)	16	16·0 (7·52–29·4)	22	73·0 (43·5–107)	23	34·3 (15·8–65·5)	30
Mali	3·48 (1·78–6·65)	17	2·71 (1·44–4·55)	19	9·58 (5·09–17·5)	16	4·00 (2·11–6·41)	23	21·6 (11·8–38·7)	21	13·0 (6·99–21·4)	25	54·8 (29·6–96·5)	31	24·7 (13·3–42·5)	36
Mauritania	2·93 (1·43–5·76)	6	1·34 (0·666–2·41)	16	6·74 (3·45–13·1)	14	2·15 (1·10–3·62)	21	15·4 (7·61–31·9)	21	8·64 (4·44–14·5)	26	43·3 (23·3–80·8)	29	18·0 (9·16–30·4)	41
Niger	3·17 (1·45–6·90)	12	2·11 (1·04–3·89)	16	8·93 (4·61–17·1)	12	3·24 (1·68–5·44)	20	22·4 (11·9–42·1)	21	12·7 (7·07–20·9)	24	54·8 (31·0–94·6)	27	26·5 (15·1–41·3)	32
Nigeria	4·72 (3·01–6·56)	8	1·04 (0·546–1·75)	17	11·7 (7·61–16·1)	12	1·67 (0·942–2·75)	23	26·7 (15·8–38·6)	16	6·27 (3·71–10·2)	29	87·5 (53·2–117)	17	19·6 (12·9–28·0)	37
Sao Tome and Principe	1·31 (0·642–2·27)	20	0·250 (0·111–0·507)	48	2·53 (1·32–4·31)	24	0·762 (0·320–1·28)	40	4·88 (3·14–7·14)	38	1·86 (0·957–2·90)	54	8·27 (5·52–12·3)	53	2·56 (1·62–3·87)	69
Senegal	7·07 (4·32–11·0)	3	2·20 (1·20–3·87)	15	16·4 (10·4–25·3)	10	3·24 (1·93–5·35)	19	41·3 (26·8–60·7)	14	11·6 (7·04–18·0)	23	115 (76·1–168)	17	29·3 (17·9–44·3)	31
Sierra Leone	4·50 (2·18–8·65)	8	2·57 (1·18–4·46)	15	10·7 (5·77–19·7)	11	3·78 (1·85–6·32)	21	23·5 (12·7–42·2)	20	13·0 (6·98–20·7)	24	54·6 (32·2–91·2)	28	27·2 (15·6–41·6)	34
Togo	7·41 (4·21–12·0)	7	2·44 (1·21–4·16)	15	21·1 (12·4–33·7)	11	3·88 (1·97–6·62)	20	46·4 (28·3–69·5)	15	12·4 (6·61–20·2)	24	99·7 (63·0–147)	18	26·8 (14·9–42·3)	35

Appendix Table S9: Suicides by firearm. Age-standardised mortality rate per 100k for males, females, and both sexes combined. Estimates provided at the global, super-region, regional, and national level, 2021.

Location	Male	Female	Both
**Global**	**1·25 (1·00–1·55)**	**0·157 (0·119–0·196)**	**0·687 (0·561–0·824)**
Low SDI	0·942 (0·359–1·59)	0·236 (0·0960–0·420)	0·584 (0·264–0·909)
Low-middle SDI	1·02 (0·506–1·71)	0·122 (0·0505–0·200)	0·568 (0·303–0·913)
Middle SDI	0·415 (0·270–0·617)	0·0479 (0·0307–0·0685)	0·230 (0·151–0·323)
High-middle SDI	0·612 (0·528–0·687)	0·0412 (0·0319–0·0499)	0·317 (0·276–0·355)
High SDI	3·81 (3·69–3·94)	0·556 (0·530–0·583)	2·16 (2·10–2·23)
**Central Europe, Eastern Europe, and Central Asia**	**0·993 (0·896–1·09)**	**0·0271 (0·0232–0·0340)**	**0·471 (0·427–0·514)**
Central Asia	0·413 (0·371–0·463)	0·0200 (0·0177–0·0231)	0·201 (0·181–0·225)
Armenia	0·771 (0·687–0·875)	0·0565 (0·0492–0·0649)	0·387 (0·345–0·438)
Azerbaijan	0·0809 (0·0291–0·138)	0·00889 (0·00405–0·0170)	0·0430 (0·0181–0·0716)
Georgia	2·54 (2·19–2·93)	0·0613 (0·0512–0·0730)	1·21 (1·04–1·40)
Kazakhstan	0·515 (0·454–0·583)	0·0182 (0·0155–0·0210)	0·242 (0·213–0·273)
Kyrgyzstan	0·521 (0·417–0·648)	0·0179 (0·0147–0·0216)	0·251 (0·203–0·308)
Mongolia	1·23 (0·500–2·11)	0·0609 (0·0288–0·113)	0·608 (0·273–1·02)
Tajikistan	0·0136 (0·00391–0·0263)	0·000483 (0·000137–0·000949)	0·00699 (0·00213–0·0134)
Turkmenistan	0·366 (0·287–0·483)	0·0286 (0·0203–0·0478)	0·196 (0·153–0·259)
Uzbekistan	0·127 (0·109–0·147)	0·0110 (0·00904–0·0133)	0·0659 (0·0564–0·0765)
Central Europe	1·01 (0·804–1·27)	0·0482 (0·0353–0·0724)	0·505 (0·403–0·632)
Albania	1·06 (0·492–1·80)	0·218 (0·0945–0·421)	0·632 (0·328–0·989)
Bosnia and Herzegovina	1·48 (0·667–2·71)	0·102 (0·0461–0·187)	0·763 (0·385–1·35)
Bulgaria	1·44 (1·23–1·67)	0·0745 (0·0601–0·0907)	0·732 (0·623–0·847)
Croatia	2·76 (2·37–3·14)	0·113 (0·0938–0·131)	1·36 (1·16–1·55)
Czechia	1·99 (1·74–2·24)	0·0643 (0·0526–0·0761)	0·982 (0·858–1·11)
Hungary	0·864 (0·770–0·960)	0·0208 (0·0176–0·0243)	0·413 (0·368–0·458)
Montenegro	4·08 (2·10–6·63)	0·466 (0·246–0·863)	2·18 (1·23–3·41)

North Macedonia	0·951 (0·378–1·51)	0·0734 (0·0379–0·157)	0·505 (0·220–0·801)
Poland	0·299 (0·270–0·328)	0·00330 (0·00286–0·00372)	0·147 (0·133–0·161)
Romania	0·122 (0·103–0·142)	0·00668 (0·00556–0·00793)	0·0628 (0·0527–0·0732)
Serbia	2·70 (1·26–4·67)	0·177 (0·105–0·339)	1·39 (0·684–2·34)
Slovakia	1·41 (0·611–2·57)	0·0546 (0·0281–0·100)	0·704 (0·317–1·28)
Slovenia	2·27 (1·99–2·57)	0·0475 (0·0372–0·0603)	1·12 (0·977–1·27)
Eastern Europe	1·20 (1·08–1·33)	0·0213 (0·0187–0·0244)	0·559 (0·502–0·621)
Belarus	0·529 (0·417–0·670)	0·000599 (0·000454–0·000770)	0·239 (0·187–0·303)
Estonia	1·49 (1·27–1·71)	0·0346 (0·0289–0·0412)	0·701 (0·595–0·803)
Latvia	1·39 (1·16–1·61)	0·0250 (0·0203–0·0312)	0·644 (0·539–0·746)
Lithuania	1·11 (0·952–1·26)	0·0207 (0·0171–0·0247)	0·515 (0·443–0·587)
Republic of Moldova	0·408 (0·351–0·477)	0·0152 (0·0129–0·0179)	0·198 (0·170–0·231)
Russian Federation	1·19 (1·08–1·29)	0·0221 (0·0196–0·0246)	0·553 (0·506–0·597)
Ukraine	1·42 (0·966–1·93)	0·0233 (0·0142–0·0356)	0·670 (0·458–0·905)
**High-income**	**4·08 (3·96–4·21)**	**0·575 (0·548–0·603)**	**2·29 (2·22–2·36)**
Australasia	1·07 (0·998–1·16)	0·0405 (0·0362–0·0459)	0·543 (0·505–0·587)
Australia	1·05 (0·970–1·14)	0·0406 (0·0361–0·0465)	0·532 (0·491–0·579)
New Zealand	1·17 (1·09–1·24)	0·0395 (0·0356–0·0440)	0·595 (0·556–0·632)
High-income Asia Pacific	0·0162 (0·0116–0·0248)	0·00128 (0·00101–0·00166)	0·00880 (0·00637–0·0131)
Brunei Darussalam	0·00861 (0·00278–0·0148)	0·000580 (0·000194–0·000944)	0·00478 (0·00175–0·00805)
Japan	0·0123 (0·0119–0·0126)	0·00124 (0·00116–0·00131)	0·00673 (0·00650–0·00693)
Republic of Korea	0·0245 (0·00950–0·0529)	0·00140 (0·000449–0·00257)	0·0131 (0·00512–0·0272)
Singapore	0·0155 (0·0140–0·0171)	0·00129 (0·00116–0·00144)	0·00849 (0·00774–0·00933)
High-income North America	10·1 (9·74–10·5)	1·56 (1·48–1·64)	5·71 (5·50–5·91)
Canada	2·78 (2·60–3·00)	0·184 (0·162–0·208)	1·45 (1·36–1·56)
Greenland	22·8 (13·0–35·6)	2·13 (0·928–3·30)	12·9 (7·70–19·4)
United States of America	10·9 (10·5–11·3)	1·72 (1·63–1·80)	6·19 (5·96–6·42)

Southern Latin America	2·83 (2·63–3·03)	0·263 (0·231–0·297)	1·46 (1·36–1·56)
Argentina	3·39 (3·12–3·65)	0·314 (0·272–0·361)	1·74 (1·61–1·87)
Chile	0·770 (0·705–0·838)	0·0575 (0·0521–0·0629)	0·392 (0·361–0·425)
Uruguay	7·08 (6·53–7·61)	0·713 (0·640–0·799)	3·61 (3·34–3·87)
Western Europe	1·28 (1·21–1·34)	0·0623 (0·0589–0·0663)	0·639 (0·607–0·672)
Andorra	0·711 (0·323–2·31)	0·0174 (0·00657–0·0798)	0·370 (0·173–1·19)
Austria	3·02 (2·82–3·21)	0·125 (0·111–0·141)	1·48 (1·38–1·57)
Belgium	1·73 (1·61–1·85)	0·151 (0·135–0·168)	0·907 (0·846–0·970)
Cyprus	1·08 (0·514–1·86)	0·00218 (0·000924–0·00424)	0·512 (0·245–0·881)
Denmark	1·40 (1·28–1·51)	0·0286 (0·0246–0·0328)	0·689 (0·633–0·747)
Finland	3·48 (3·16–3·80)	0·112 (0·0992–0·127)	1·75 (1·59–1·90)
France	2·99 (2·69–3·31)	0·134 (0·116–0·153)	1·46 (1·32–1·61)
Germany	1·04 (0·964–1·11)	0·0518 (0·0468–0·0576)	0·518 (0·483–0·552)
Greece	1·16 (1·03–1·30)	0·0556 (0·0484–0·0633)	0·586 (0·523–0·651)
Iceland	2·05 (1·80–2·27)	0·0316 (0·0269–0·0369)	1·04 (0·919–1·16)
Ireland	0·621 (0·552–0·708)	0·0257 (0·0225–0·0293)	0·313 (0·278–0·357)
Israel	2·49 (2·30–2·68)	0·176 (0·157–0·197)	1·29 (1·20–1·39)
Italy	0·849 (0·802–0·895)	0·0573 (0·0531–0·0620)	0·431 (0·408–0·455)
Luxembourg	1·21 (1·07–1·34)	0·0659 (0·0565–0·0759)	0·617 (0·550–0·685)
Malta	0·637 (0·558–0·717)	0·0312 (0·0268–0·0366)	0·330 (0·289–0·372)
Monaco	2·27 (1·11–3·80)	1·31 (0·800–2·20)	1·76 (1·10–2·74)
Netherlands	0·320 (0·299–0·342)	0·0164 (0·0142–0·0186)	0·162 (0·152–0·173)
Norway	2·01 (1·90–2·12)	0·106 (0·0977–0·116)	1·06 (1·01–1·12)
Portugal	1·15 (1·08–1·23)	0·0653 (0·0565–0·0751)	0·563 (0·529–0·601)
San Marino	2·83 (1·36–4·87)	0·0413 (0·0192–0·0888)	1·38 (0·673–2·36)
Spain	0·566 (0·513–0·622)	0·0149 (0·0130–0·0169)	0·281 (0·255–0·308)
Sweden	1·31 (1·13–1·51)	0·0473 (0·0403–0·0550)	0·676 (0·583–0·774)

Switzerland	3·04<br>(2·81–3·27)	0·143<br>(0·127–0·160)	1·54<br>(1·43–1·65)
United Kingdom	0·195<br>(0·189–0·200)	0·00838<br>(0·00804–0·00872)	0·0991<br>(0·0963–0·102)
**Latin America and Caribbean**	**1·18<br>(1·07–1·31)**	**0·105<br>(0·0925–0·118)**	**0·623<br>(0·564–0·689)**
Andean Latin America	0·403<br>(0·267–0·546)	0·0314<br>(0·0160–0·0466)	0·214<br>(0·144–0·283)
Bolivia (Plurinational State of)	0·628<br>(0·210–0·972)	0·0817<br>(0·0338–0·140)	0·349<br>(0·133–0·529)
Ecuador	0·698<br>(0·544–0·887)	0·0165<br>(0·0128–0·0214)	0·349<br>(0·272–0·443)
Peru	0·195<br>(0·0651–0·352)	0·0232<br>(0·00910–0·0402)	0·108<br>(0·0429–0·185)
Caribbean	0·803<br>(0·524–1·15)	0·0919<br>(0·0560–0·125)	0·439<br>(0·304–0·604)
Antigua and Barbuda	0·0412<br>(0·0369–0·0465)	0·00364<br>(0·00318–0·00412)	0·0218<br>(0·0196–0·0244)
Bahamas	0·257<br>(0·201–0·320)	0·00497<br>(0·00375–0·00651)	0·125<br>(0·0975–0·155)
Barbados	0·302<br>(0·233–0·385)	0·0735<br>(0·0556–0·0957)	0·182<br>(0·140–0·232)
Belize	0·816<br>(0·697–0·951)	0·0808<br>(0·0682–0·0959)	0·445<br>(0·384–0·517)
Bermuda	0·0382<br>(0·0318–0·0469)	0·00526<br>(0·00414–0·00670)	0·0211<br>(0·0176–0·0258)
Cuba	0·375<br>(0·318–0·438)	0·0747<br>(0·0632–0·0879)	0·223<br>(0·191–0·257)
Dominica	0·172<br>(0·0608–0·279)	0·00338<br>(0·00141–0·00584)	0·0875<br>(0·0316–0·141)
Dominican Republic	1·19<br>(0·521–2·00)	0·147<br>(0·0581–0·237)	0·668<br>(0·332–1·09)
Grenada	0·0144<br>(0·0122–0·0168)	0·000710<br>(0·000582–0·000843)	0·00746<br>(0·00633–0·00873)
Guyana	0·685<br>(0·529–0·885)	0·0281<br>(0·0198–0·0374)	0·345<br>(0·267–0·447)
Haiti	0·723<br>(0·251–1·50)	0·0793<br>(0·0184–0·199)	0·387<br>(0·148–0·740)
Jamaica	0·576<br>(0·432–0·766)	0·108<br>(0·0791–0·146)	0·337<br>(0·252–0·444)
Puerto Rico	1·81<br>(1·49–2·13)	0·0281<br>(0·0230–0·0338)	0·873<br>(0·719–1·03)
Saint Kitts and Nevis	0·0404<br>(0·0329–0·0502)	0·0116<br>(0·00935–0·0141)	0·0258<br>(0·0213–0·0321)
Saint Lucia	0·257<br>(0·209–0·312)	0·0157<br>(0·0125–0·0195)	0·135<br>(0·111–0·164)
Saint Vincent and the Grenadines	0·165<br>(0·142–0·193)	0·00589<br>(0·00477–0·00715)	0·0867<br>(0·0747–0·101)
Suriname	1·47<br>(0·604–2·41)	0·154<br>(0·0781–0·251)	0·793<br>(0·378–1·25)
Trinidad and Tobago	0·351<br>(0·267–0·447)	0·0199<br>(0·0148–0·0263)	0·185<br>(0·140–0·235)
United States Virgin Islands	3·96<br>(2·03–6·43)	0·114<br>(0·0571–0·290)	1·97<br>(1·05–3·15)

Central Latin America	1·50 (1·30–1·72)	0·114 (0·0956–0·133)	0·774 (0·674–0·892)
Colombia	1·25 (1·02–1·50)	0·0811 (0·0655–0·0985)	0·640 (0·526–0·773)
Costa Rica	1·86 (1·64–2·11)	0·0985 (0·0829–0·115)	0·929 (0·820–1·06)
El Salvador	0·336 (0·123–0·516)	0·0343 (0·0170–0·0545)	0·168 (0·0720–0·248)
Guatemala	0·792 (0·669–0·930)	0·0678 (0·0562–0·0814)	0·407 (0·344–0·476)
Honduras	1·55 (0·615–2·46)	0·109 (0·0393–0·208)	0·783 (0·329–1·22)
Mexico	0·995 (0·862–1·15)	0·0941 (0·0786–0·111)	0·523 (0·458–0·596)
Nicaragua	0·576 (0·261–1·05)	0·0355 (0·0187–0·0712)	0·291 (0·138–0·517)
Panama	0·407 (0·325–0·495)	0·0220 (0·0171–0·0269)	0·213 (0·170–0·258)
Venezuela (Bolivarian Republic of)	6·06 (4·63–7·70)	0·369 (0·263–0·505)	3·04 (2·31–3·88)
Tropical Latin America	1·15 (1·09–1·20)	0·118 (0·109–0·127)	0·609 (0·578–0·636)
Brazil	1·13 (1·08–1·18)	0·112 (0·105–0·121)	0·599 (0·573–0·623)
Paraguay	1·64 (0·796–2·75)	0·275 (0·148–0·511)	0·942 (0·543–1·54)
**North Africa and Middle East**	**0·484 (0·213–0·730)**	**0·0937 (0·0411–0·140)**	**0·296 (0·152–0·425)**
North Africa and Middle East	0·484 (0·213–0·730)	0·0937 (0·0411–0·140)	0·296 (0·152–0·425)
Afghanistan	0·402 (0·135–0·903)	0·159 (0·0426–0·410)	0·278 (0·120–0·560)
Algeria	0·169 (0·0607–0·311)	0·0454 (0·0132–0·0798)	0·108 (0·0452–0·183)
Bahrain	0·0814 (0·0290–0·154)	0·00961 (0·00421–0·0170)	0·0546 (0·0218–0·101)
Egypt	0·153 (0·0643–0·237)	0·0220 (0·00996–0·0361)	0·0904 (0·0417–0·135)
Iran (Islamic Republic of)	0·265 (0·0888–0·373)	0·0904 (0·0348–0·129)	0·179 (0·0751–0·242)
Iraq	1·55 (0·688–2·45)	0·253 (0·147–0·440)	0·915 (0·489–1·42)
Jordan	0·0783 (0·0268–0·126)	0·0172 (0·00710–0·0314)	0·0506 (0·0206–0·0804)
Kuwait	0·0302 (0·0239–0·0369)	0·0124 (0·0102–0·0148)	0·0220 (0·0180–0·0263)
Lebanon	0·308 (0·157–0·486)	0·124 (0·0687–0·187)	0·214 (0·135–0·312)
Libya	0·306 (0·118–0·514)	0·0740 (0·0187–0·169)	0·193 (0·0842–0·334)
Morocco	0·175 (0·0531–0·375)	0·0719 (0·0164–0·152)	0·123 (0·0490–0·232)
Oman	0·0121 (0·00274–0·0214)	0·00177 (0·000403–0·00305)	0·00809 (0·00205–0·0140)

Palestine	0·0519<br>(0·0172–0·0875)	0·00963<br>(0·00274–0·0151)	0·0306<br>(0·0123–0·0478)
Qatar	0·0141<br>(0·00387–0·0297)	0·00178<br>(0·000713–0·00362)	0·0103<br>(0·00339–0·0210)
Saudi Arabia	0·480<br>(0·196–0·834)	0·0899<br>(0·0409–0·160)	0·324<br>(0·145–0·562)
Sudan	0·369<br>(0·124–0·650)	0·128<br>(0·0223–0·313)	0·249<br>(0·101–0·444)
Syrian Arab Republic	0·116<br>(0·0460–0·210)	0·00876<br>(0·00389–0·0174)	0·0579<br>(0·0257–0·100)
Tunisia	0·152<br>(0·0459–0·335)	0·0368<br>(0·0101–0·0794)	0·0930<br>(0·0357–0·191)
Türkiye	1·40<br>(0·639–2·06)	0·163<br>(0·0698–0·246)	0·781<br>(0·397–1·11)
United Arab Emirates	0·0452<br>(0·0161–0·0828)	0·0161<br>(0·00625–0·0297)	0·0335<br>(0·0137–0·0560)
Yemen	0·346<br>(0·0953–0·886)	0·0883<br>(0·00986–0·255)	0·216<br>(0·0682–0·492)
**South Asia**	**1·13<br>(0·449–2·10)**	**0·119<br>(0·0375–0·227)**	**0·627<br>(0·285–1·11)**
South Asia	1·13<br>(0·449–2·10)	0·119<br>(0·0375–0·227)	0·627<br>(0·285–1·11)
Bangladesh	0·430<br>(0·123–1·16)	0·0237<br>(0·00885–0·0563)	0·219<br>(0·0713–0·566)
Bhutan	0·875<br>(0·331–1·83)	0·0802<br>(0·0265–0·192)	0·495<br>(0·226–1·01)
India	1·21<br>(0·494–2·18)	0·141<br>(0·0413–0·275)	0·683<br>(0·309–1·18)
Nepal	1·97<br>(0·676–4·65)	0·131<br>(0·0305–0·348)	0·974<br>(0·354–2·20)
Pakistan	0·942<br>(0·356–2·21)	0·0457<br>(0·0194–0·103)	0·501<br>(0·198–1·14)
**Southeast Asia, East Asia, and Oceania**	**0·0675<br>(0·0308–0·123)**	**0·00355<br>(0·00203–0·00733)**	**0·0356<br>(0·0172–0·0631)**
East Asia	0·00911<br>(0·00475–0·0163)	0·000917<br>(0·000546–0·00208)	0·00501<br>(0·00286–0·00855)
China	0·00687<br>(0·00258–0·0127)	0·000877<br>(0·000504–0·00199)	0·00385<br>(0·00176–0·00670)
Democratic People's Republic of Korea	0·0376<br>(0·0165–0·115)	0·00262<br>(0·000939–0·00771)	0·0198<br>(0·00929–0·0563)
Taiwan (Province of China)	0·101<br>(0·0908–0·111)	0·00136<br>(0·00121–0·00150)	0·0511<br>(0·0460–0·0568)
Oceania	0·367<br>(0·121–0·575)	0·00193<br>(0·000819–0·00461)	0·189<br>(0·0627–0·295)
American Samoa	1·30<br>(0·603–2·17)	0·000213<br>(0·0000906–0·000421)	0·658<br>(0·305–1·09)
Cook Islands	0·583<br>(0·136–1·14)	0·00212<br>(0·000693–0·00602)	0·275<br>(0·0647–0·537)
Fiji	0·0112<br>(0·00297–0·0181)	0·000889<br>(0·000158–0·00157)	0·00587<br>(0·00163–0·00935)
Guam	2·52<br>(1·29–4·25)	0·00182<br>(0·000904–0·00372)	1·29<br>(0·655–2·17)
Kiribati	0·166<br>(0·0373–0·283)	0·00715<br>(0·00191–0·0123)	0·0813<br>(0·0209–0·135)

Marshall Islands	0·857 (0·199–1·73)	0·00273 (0·000682–0·00649)	0·439 (0·103–0·886)
Micronesia (Federated States of)	1·27 (0·357–2·14)	0·00463 (0·00156–0·0105)	0·636 (0·181–1·06)
Nauru	1·13 (0·299–2·10)	0·00421 (0·00126–0·00890)	0·553 (0·147–1·03)
Niue	0·736 (0·191–1·38)	0·00347 (0·000718–0·00934)	0·364 (0·0966–0·686)
Northern Mariana Islands	2·07 (0·996–3·79)	0·00182 (0·000913–0·00671)	1·07 (0·523–2·00)
Palau	0·868 (0·309–1·44)	0·0373 (0·00623–0·0691)	0·520 (0·197–0·848)
Papua New Guinea	0·223 (0·0603–0·422)	0·00142 (0·000353–0·00414)	0·117 (0·0321–0·221)
Samoa	0·855 (0·243–1·39)	0·00438 (0·00169–0·00982)	0·436 (0·125–0·706)
Solomon Islands	1·52 (0·433–2·74)	0·00632 (0·00185–0·0168)	0·767 (0·219–1·38)
Tokelau	0·775 (0·213–1·35)	0·00398 (0·000809–0·00957)	0·393 (0·110–0·683)
Tonga	0·310 (0·0751–0·547)	0·00194 (0·000660–0·00495)	0·149 (0·0370–0·263)
Tuvalu	0·773 (0·171–1·70)	0·00315 (0·000848–0·00834)	0·399 (0·0932–0·882)
Vanuatu	1·29 (0·356–2·15)	0·00472 (0·00159–0·0105)	0·637 (0·181–1·07)
Southeast Asia	0·209 (0·0932–0·395)	0·00968 (0·00528–0·0193)	0·106 (0·0499–0·195)
Cambodia	0·152 (0·0760–0·349)	0·00975 (0·00387–0·0237)	0·0745 (0·0372–0·163)
Indonesia	0·0451 (0·0212–0·125)	0·00338 (0·00166–0·00713)	0·0237 (0·0125–0·0638)
Lao People's Democratic Republic	0·169 (0·0806–0·438)	0·0110 (0·00352–0·0278)	0·0890 (0·0438–0·224)
Malaysia	0·195 (0·0713–0·297)	0·00374 (0·00140–0·00566)	0·102 (0·0374–0·154)
Maldives	0·0484 (0·0156–0·0944)	0·00129 (0·000478–0·00212)	0·0300 (0·00975–0·0596)
Mauritius	0·228 (0·209–0·246)	0·0282 (0·0243–0·0323)	0·126 (0·115–0·135)
Myanmar	0·161 (0·0705–0·379)	0·00489 (0·00174–0·0136)	0·0768 (0·0372–0·177)
Philippines	0·153 (0·0508–0·225)	0·00705 (0·00177–0·0100)	0·0789 (0·0279–0·115)
Seychelles	0·118 (0·0527–0·300)	0·00228 (0·00105–0·00571)	0·0620 (0·0289–0·158)
Sri Lanka	0·120 (0·0395–0·208)	0·0104 (0·00489–0·0184)	0·0620 (0·0232–0·102)
Thailand	1·03 (0·446–1·58)	0·0375 (0·0183–0·0645)	0·515 (0·229–0·771)
Timor-Leste	0·164 (0·0767–0·486)	0·0110 (0·00416–0·0304)	0·0874 (0·0424–0·248)
Viet Nam	0·192 (0·0782–0·522)	0·0126 (0·00588–0·0386)	0·0965 (0·0445–0·248)

**Sub-Saharan Africa**	**0·822 (0·261–1·41)**	**0·277 (0·125–0·448)**	**0·539 (0·239–0·831)**
Central Sub-Saharan Africa	0·879 (0·252–2·07)	0·367 (0·127–0·747)	0·604 (0·266–1·15)
Angola	0·644 (0·199–1·21)	0·261 (0·0953–0·443)	0·433 (0·197–0·703)
Central African Republic	1·39 (0·446–3·25)	0·571 (0·183–1·16)	0·942 (0·409–1·72)
Congo	0·564 (0·197–1·00)	0·293 (0·0923–0·521)	0·425 (0·194–0·655)
Democratic Republic of the Congo	0·964 (0·251–2·63)	0·402 (0·123–0·981)	0·663 (0·282–1·40)
Equatorial Guinea	0·485 (0·137–0·970)	0·207 (0·0499–0·481)	0·333 (0·139–0·586)
Gabon	0·516 (0·149–0·938)	0·192 (0·0587–0·370)	0·344 (0·151–0·563)
Eastern Sub-Saharan Africa	0·799 (0·266–1·50)	0·341 (0·124–0·610)	0·560 (0·247–0·904)
Burundi	0·900 (0·213–2·61)	0·331 (0·0655–1·05)	0·623 (0·182–1·57)
Comoros	0·456 (0·108–1·09)	0·299 (0·0894–0·638)	0·374 (0·139–0·728)
Djibouti	0·414 (0·110–0·962)	0·288 (0·101–0·500)	0·358 (0·147–0·687)
Eritrea	1·11 (0·410–2·11)	0·569 (0·211–0·896)	0·819 (0·404–1·27)
Ethiopia	0·678 (0·189–1·54)	0·339 (0·116–0·660)	0·510 (0·213–0·937)
Kenya	0·569 (0·180–1·22)	0·249 (0·102–0·461)	0·400 (0·196–0·722)
Madagascar	0·759 (0·252–1·54)	0·429 (0·164–0·744)	0·588 (0·254–0·959)
Malawi	1·38 (0·449–2·94)	0·314 (0·0902–0·896)	0·802 (0·317–1·51)
Mozambique	1·49 (0·438–3·17)	0·445 (0·159–0·825)	0·911 (0·378–1·66)
Rwanda	0·623 (0·178–1·45)	0·269 (0·0684–0·625)	0·425 (0·160–0·841)
Somalia	1·36 (0·329–4·31)	0·824 (0·192–2·27)	1·06 (0·375–2·62)
South Sudan	1·22 (0·454–2·22)	0·641 (0·229–1·12)	0·934 (0·439–1·54)
Uganda	0·602 (0·148–1·55)	0·203 (0·0588–0·483)	0·381 (0·136–0·758)
United Republic of Tanzania	0·645 (0·209–1·21)	0·257 (0·101–0·419)	0·441 (0·203–0·758)
Zambia	0·973 (0·404–1·64)	0·330 (0·134–0·593)	0·640 (0·310–1·02)
Southern Sub-Saharan Africa	0·980 (0·346–1·65)	0·154 (0·0548–0·280)	0·536 (0·238–0·863)
Botswana	0·878 (0·336–1·50)	0·171 (0·0575–0·403)	0·495 (0·215–0·797)
Eswatini	1·84 (0·549–3·38)	0·313 (0·0786–0·657)	0·998 (0·421–1·75)

Lesotho	2·04 (0·619–4·04)	0·444 (0·102–1·05)	1·15 (0·474–2·02)
Namibia	1·21 (0·477–2·15)	0·155 (0·0586–0·322)	0·625 (0·276–1·09)
South Africa	0·818 (0·253–1·42)	0·133 (0·0454–0·293)	0·454 (0·188–0·743)
Zimbabwe	1·72 (0·684–3·47)	0·192 (0·0460–0·458)	0·864 (0·393–1·65)
Western Sub-Saharan Africa	0·802 (0·212–1·37)	0·244 (0·118–0·341)	0·512 (0·206–0·801)
Benin	0·897 (0·217–1·61)	0·229 (0·0993–0·347)	0·558 (0·202–0·943)
Burkina Faso	1·19 (0·328–2·19)	0·345 (0·176–0·523)	0·755 (0·307–1·26)
Cabo Verde	1·75 (0·766–2·82)	0·151 (0·0987–0·238)	0·926 (0·451–1·46)
Cameroon	0·919 (0·268–1·71)	0·231 (0·0946–0·361)	0·579 (0·212–0·990)
Chad	1·05 (0·284–2·13)	0·394 (0·169–0·667)	0·734 (0·283–1·29)
Côte d'Ivoire	1·09 (0·298–1·86)	0·206 (0·0871–0·316)	0·678 (0·250–1·10)
Gambia	0·805 (0·193–1·74)	0·289 (0·132–0·588)	0·560 (0·231–0·989)
Ghana	0·247 (0·0731–0·440)	0·332 (0·183–0·573)	0·307 (0·173–0·482)
Guinea	0·850 (0·204–1·67)	0·306 (0·136–0·448)	0·573 (0·223–0·997)
Guinea-Bissau	1·53 (0·451–2·42)	0·396 (0·175–0·603)	0·934 (0·380–1·38)
Liberia	1·23 (0·277–2·79)	0·349 (0·133–0·709)	0·805 (0·257–1·60)
Mali	0·964 (0·213–2·12)	0·327 (0·135–0·558)	0·643 (0·219–1·26)
Mauritania	0·473 (0·112–1·03)	0·211 (0·0908–0·388)	0·336 (0·123–0·620)
Niger	0·992 (0·204–2·33)	0·327 (0·130–0·553)	0·648 (0·232–1·29)
Nigeria	0·684 (0·173–1·22)	0·183 (0·0935–0·271)	0·414 (0·167–0·687)
Sao Tome and Principe	0·158 (0·0387–0·328)	0·0235 (0·0118–0·0418)	0·0903 (0·0304–0·174)
Senegal	1·16 (0·379–2·01)	0·326 (0·173–0·496)	0·741 (0·327–1·18)
Sierra Leone	0·743 (0·167–1·74)	0·268 (0·115–0·447)	0·512 (0·172–1·00)
Togo	1·14 (0·290–2·49)	0·266 (0·106–0·517)	0·679 (0·253–1·27)
