# Supplementary material for: Global, regional, and national burden of suicide, 1990–2021: a systematic analysis for the Global Burden of Disease Study 2021
Source: Lancet Public Health. 2025 Feb 19;10(3):e189–202. doi: 10.1016/S2468-2667(25)00006-4 (PMC11876099; doi:10.1016/S2468-2667(25)00006-4)
Supplement: Supplementary appendix 2 [file mmc2.pdf]

THE LANCET
Public Health

## Supplementary appendix 2

This appendix formed part of the original submission and has been peer reviewed. We post it as supplied by the authors.

Supplement to: GBD 2021 Suicide Collaborators. Global, regional, and national burden of suicide, 1990–2021: a systematic analysis for the Global Burden of Disease Study 2021. *Lancet Public Health* 2025; published online Feb 19. https://doi.org/10.1016/S2468-2667(25)00006-4.

# Appendix 2: Authorship appendix to "Global, regional, and national burden of suicide 1990 to 2021: a systematic analysis for the Global Burden of Disease Study 2021"

This appendix provides further authorship detail for "Global, regional, and national burden of suicide 1990 to 2021: a systematic analysis for the Global Burden of Disease Study 2021"

## Table of Contents

Appendix 2: Authorship appendix to "Global, regional, and national burden of suicide 1990 to 2021: a systematic analysis for the Global Burden of Disease Study 2021" .................................. 1

**GBD 2021 Suicide Collaborators ..................................................... 2**

**Affiliations ..................................................... 3**

**Authors' Contributions ..................................................... 14**

Managing the overall research enterprise ..................................................... 14

Writing the first draft of the manuscript ..................................................... 14

Primary responsibility for applying analytical methods to produce estimates ..................................................... 14

Primary responsibility for seeking, cataloguing, extracting, or cleaning data; designing or coding figures and tables ..................................................... 14

Providing data or critical feedback on data sources ..................................................... 14

Developing methods or computational machinery ..................................................... 15

Providing critical feedback on methods or results ..................................................... 16

Drafting the work or revising it critically for important intellectual content ..................................................... 17

Managing the estimation or publications process ..................................................... 18

## GBD 2021 Suicide Collaborators

Nicole Davis Weaver*, Gregory J Bertolacci*, Emily Rosenblad, Sama Ghoba, Matthew Cunningham, Kevin S Ikuta, Madeline E Moberg, Vincent Mougin, Chieh Han, Eve E Wool, Yohannes Habtegiorgis Abate, Habeeb Omoponle Adewuyi, Qorinah Estiningtyas Sakilah Adnani, Leticia Akua Adzigbli, Aanuoluwapo Adeyimika Afolabi, Suneth Buddhika Agampodi, Bright Opoku Ahinkorah, Aqeel Ahmad, Danish Ahmad, Sajjad Ahmad, Ayman Ahmed, Haroon Ahmed, Hanadi Al Hamad, Yazan Al-Ajlouni, Rasmieh Mustafa Al-amer, Mohammed Albashtawy, Wafa A Aldhaleei, Syed Shujait Ali, Waad Ali, Mahmoud A Alomari, Mohammed A Alsabri, Nelson Alvis-Guzman, Yaser Mohammed Al-Worafi, Alireza Amindarolzarbi, Sohrab Amiri, Tudorel Andrei, Saeid Anvari, Jalal Arabloo, Demelash Areda, Anton A Artamonov, Tahira Ashraf, Seyyed Shamsadin Athari, Maha Moh'd Wahbi Atout, Ahmed Y. Azzam, Ashish D Badiye, Nayereh Baghcheghi, Saeed Bahramian, Maciej Banach, Suzanne Lyn Barker-Collo, Till Winfried Bärnighausen, Amadou Barrow, Azadeh Bashiri, Hameed Akande Bashiru, Mohammad-Mahdi Bastan, Kavita Batra, Ravi Batra, Mohsen Bayati, Corina Benjet, Habib Benzian, Paola Bertuccio, Akshaya Srikanth Bhagavathula, Priyadarshini Bhattacharjee, Corey B Bills, Sri Harsha Boppana, Guilherme Borges, Hamed Borhany, Yasser Bustanji, Florentino Luciano Caetano dos Santos, Giulio Castelpietra, Arthur Caye, Muthia Cenderadewi, Rama Mohan Chandika, Eeshwar K Chandrasekar, Periklis Charalampous, Yifan Chen, Ritesh Chimoriya, Hitesh Chopra, Sonali Gajanan Choudhari, Dinh-Toi Chu, Isaac Sunday Chukwu, Muhammad Chutiyami, Richard G Cowden, Berihun Assefa Dachew, Omid Dadras, Xiaochen Dai, Koustuv Dalal, Lalit Dandona, Rakhi Dandona, Samuel Demissie Darcho, Reza Darvishi Cheshmeh Soltani, Claudio Alberto Dávila-Cervantes, Alejandro de la Torre-Luque, Shayom Debopadhaya, Louisa Degenhardt, Ivan Delgado-Enciso, Emina Dervišević, Michael J Diaz, Deepa Dongarwar, Ojas Prakashbhai Doshi, Haneil Larson Dsouza, Samuel C Dumith, Senbagam Duraisamy, Ejemai Eboreime, Ferry Efendi, Michael Ekholuenetale, Rabie Adel El Arab, Muhammed Elhadi, Gihan ELNahas, Chadi Eltaha, Syed Emdadul Emdadul Haque, Sharareh Eskandarieh, Ayesha Fahim, Andre Faro, Ali Fatehizadeh, Patrick Fazeli, Alireza Feizkhah, Ginenus Fekadu, Nuno Ferreira, Florian Fischer, Richard Charles Franklin, Ni Kadek Yuni Fridayani, Márió Gajdács, Aravind P Gandhi, Balasankar Ganesan, Miglas Welay Gebregergis, Mesfin Gebrehiwot, Teferi Gebru Gebremeskel, Molla Getie, Delaram J Ghadimi, Khalid Yaser Ghailan, Ahmad Ghashghaee, Ali Gholamrezanezhad, Pouya Goleij, Ayman Grada, Michal Grivna, Shi-Yang Guan, Snigdha Gulati, Sapna Gupta, Reyna Alma Gutiérrez, Roberth Steven Gutiérrez-Murillo, Erin B Hamilton, Nasrin Hanifi, Ikramul Hasan, Mahgol Sadat Hassan Zadeh Tabatabaei, Simon I Hay, Mohammad Heidari, Mehdi Hemmati, Nguyen Quoc Hoan, Mehdi Hosseinzadeh, Sorin Hostiuc, Junjie Huang, Hong-Han Huynh, Segun Emmanuel Ibitoye, Olayinka Stephen Ilesanmi, Irena M Ilic, Milena D Ilic, Mustapha Immurana, Arit Inok, Chidozie Declan Iwu, Haitham Jahrami, Sanobar Jaka, Reza Jalilzadeh Yengejeh, Zixiang Ji, Shuai Jin, Nitin Joseph, Charity Ehimwenma Joshua, Jacek Jerzy Jozwiak, Zubair Kabir, Vidya Kadashetti, Kehinde Kazeem Kanmodi, Rami S Kantar, Neeti Kapoor, Ibraheem M Karaye, Shilpi Karmakar, Harkiran Kaur, Jessica A Kerr, Himanshu Khajuria, Ajmal Khan, Khaled Khatab, Khalid A Kheirallah, Kwanghyun Kim, Min Seo Kim, Shivakumar KM KM Shivakumar, Ali-Asghar Kolahi, Hamid Reza Koohestani, Varun Krishna, Nuworza Kugbey, Mukhtar Kulimbet, G Anil Kumar, Manasi Kumar, Satyajit Kundu, Ville Kytö, Iván Landires, Nhi Huu Hanh Le, Doo Woong Lee, Wei-Chen Lee, Yo Han Lee, Stephen S Lim, Jialing Lin, Richard T Liu, José Francisco López-Gil, Giancarlo Lucchetti, Zheng Feei Ma, Venkatesh Maled, Kashish Malhotra, Ahmad Azam Malik, Agustina Maria Marconi, Ramon Martinez-Piedra, Roy Rillera Marzo, Yasith Mathangasinghe, Pallab K Maulik, Hadush Negash Meles, Ritesh G Menezes, Tuomo J Meretoja, Tomislav Mestrovic, Irmina Maria Michalek, Ted R Miller, Moonis Mirza, Awoke Misganaw, Chaitanya Mittal, Abdalla Z Mohamed, Nouh Saad Mohamed, Abdollah

Mohammadian-Hafshejani, Ali H Mokdad, Sabrina Molinaro, Lorenzo Monasta, AmirAli Moodi Ghalibaf, Shane Douglas Morrison, Rohith Motappa, Faraz Mughal, Francesk Mulita, Yanjinlkham Munkhsaikhan, Christopher J L Murray, Sathish Muthu, Woojae Myung, Ayoub Nafei, Pirouz Naghavi, Ganesh R Naik, Gurudatta Naik, Zuhair S Natto, Muhammad Naveed, Shadan Navid, Biswa Prakash Nayak, Athare Nazri-Panjaki, Henok Biresaw Netsere, Sudan Prasad Neupane, Hoang Anh Hoang Nguyen, Nhien Ngoc Y Nguyen, Phat Tuan Nguyen, Phuong The Nguyen, Van Thanh Nguyen, Ali Nikoobar, Isabel Noguer, Shuhei Nomura, Chisom Adaobi Nri-Ezedi, Virginia Nuñez-Samudio, Ogochukwu Janet Nzoputam, Bogdan Oancea, Michael Safo Oduro, In-Hwan Oh, Sylvester Reuben Okeke, Yinka Doris Oluwafemi, Sok King Ong, Michal Ordak, Heather M Orpana, Esteban Ortiz-Prado, Uchechukwu Levi Osuagwu, Alicia Padron-Monedero, Jagadish Rao Padubidri, Raul Felipe Felipe Palma-Alvarez, Anamika Pandey, Ashok Pandey, Ioannis Pantazopoulos, Seoyeon Park, Sungchul Park, Ava Pashaei, Jay Patel, Shrikant Pawar, Prince Peprah, Mario F P Peres, Ionela-Roxana Petcu, Anil K Philip, Michael R Phillips, Zahra Zahid Piracha, Jalandhar Pradhan, Elton Junio Sady Prates, Dimas Ria Angga Pribadi, Jagadeesh Puvvula, Ibrahim Qattea, Gangzhen Qian, Venkatraman Radhakrishnan, Pankaja Raghav, Sarvenaz Rahimibarghani, Afarin Rahimi-Movaghar, Vafa Rahimi-Movaghar, Md. Mosfequr Rahman, Mosiur Rahman, Muhammad Aziz Rahman, Mohammad Rahmanian, Pushp Lata Rajpoot, Mahmoud Mohammed Ramadan, Shakthi Kumaran Ramasamy, Smitha Rani, Mithun Rao, Sowmya J Rao, Mohammad-Mahdi Rashidi, Prateek Rastogi, Devarajan Rathish, David Laith Rawaf, Lennart Reifels, Mohsen Rezaeian, Taeho Gregory Rhee, Jennifer Rickard, Leonardo Roever, Moustaq Karim Khan Rony, Chandan S N, Basema Ahmad Saddik, Farideh Sadeghian, Mohammad Reza Saeb, Umar Saeed, Sahar Saeedi Moghaddam, Mehdi Safari, Dominic Sagoe, Narjes Saheb Sharif-Askari, Pragyan Monalisa Sahoo, Soumya Swaroop Sahoo, Payman Salamati, Dauda Salihu, Sohrab Salimi, Giovanni A Salum, Sonia Sameen, Abdallah M Samy, Milena M Santric-Milicevic, Chinmoy Sarkar, Gargi Sachin Sarode, Sachin C Sarode, Brijesh Sathian, Austin E Schumacher, Mario Šekerija, Mohammad H Semreen, Sadaf G Sepanlou, Mahan Shafie, Samiah Shahid, Ahmed Shaikh, Masood Ali Shaikh, Amin Sharifan, Javad Sharifi Rad, Anupam Sharma, Vishal Sharma, Rahim Ali Sheikhi, Mahabalesh Shetty, Pavanchand H Shetty, Premalatha K Shetty, Velizar Shivarov, Sina Shool, Paramdeep Singh, Puneetpal Singh, Surjit Singh, Bogdan Socea, Dan J Stein, Murray B Stein, Jing Sun, Chandan Kumar Swain, Lukasz Szarpak, Sree Sudha T Y, Seyyed Mohammad Tabatabaei, Celine Tabche, Minale Tareke, Mohamad-Hani Temsah, Chern Choong Thum, Tenaw Yimer Tiruye, Marcos Roberto Tovani-Palone, Nghia Minh Tran, Thang Huu Tran, Nguyen Tran Minh Duc, Samuel Joseph Tromans, Thien Tan Tri Tai Truyen, Guesh Mebrahtom Tsegay, Munkhtuya Tumurkhuu, Sanaz Vahdati, Asokan Govindaraj Vaithinathan, Pascual R Valdez, Tommi Juhani Vasankari, Massimiliano Veroux, Georgios-Ioannis Verras, Manish Vinayak, Theo Vos, Mandaras Tariku Walde, Yanzhong Wang, Joseph L L Ward, Nuwan Darshana Wickramasinghe, Marcin W Wojewodzic, Renjulal Yesodharan, Arzu Yiğit, Dehui Yin, Paul Yip, Dong Keon Yon, Naohiro Yonemoto, Chuanhua Yu, Iman Zare, Mohammed G M Zeariya, Haijun Zhang, Claire Chenwen Zhong, Bin Zhu, Abzal Zhumagaliuly, and Mohsen Naghavi†.

*Co-first authors

†Senior author

## Affiliations

Institute for Health Metrics and Evaluation (N Davis Weaver MPH, G J Bertolacci MS, E Rosenblad MPH, S Ghoba MS, M Cunningham MSc, K S Ikuta MD, M E Moberg MS, V Mougin BA, C Han BA, E E Wool MPH,

X Dai PhD, Prof L Dandona MD, Prof R Dandona PhD, Prof L Degenhardt PhD, E B Hamilton MPH, Prof S I Hay FMedSci, Prof S S Lim PhD, T Mestrovic PhD, Prof A H Mokdad PhD, Prof C J L Murray DPhil, A E Schumacher PhD, Prof T Vos PhD, Prof M Naghavi PhD), Department of Health Metrics Sciences, School of Medicine (X Dai PhD, Prof R Dandona PhD, Prof S I Hay FMedSci, Prof S S Lim PhD, A Misganaw PhD, Prof A H Mokdad PhD, Prof C J L Murray DPhil, Prof T Vos PhD, Prof M Naghavi PhD), School of Health Systems and Public Health (C Iwu MPH), University of Washington, Seattle, WA, USA; Division of Infectious Diseases (K S Ikuta MD), Veterans Affairs Greater Los Angeles, Los Angeles, CA, USA; Department of Clinical Governance and Quality Improvement (Y H Abate MSc), Aleta Wondo General Hospital, Aleta Wondo, Ethiopia; Department of Educational Counselling and Developmental Psychology (H O Adewuyi PhD), Department of Health Promotion and Education (S Ibitoye PhD), University of Ibadan, Ibadan, Nigeria; Department of Educational Psychology (H O Adewuyi PhD), University of Johannesburg, Johannesburg, South Africa; Department of Public Health (Q Adnani PhD), Padjadjaran University, Bandung, Indonesia; Department of Epidemiology and Biostatistics (L A Adzigbli BSc), Institute of Health Research (M Immurana PhD), University of Health and Allied Sciences, Ho, Ghana; Technical Services Directorate (A A Afolabi MPH), MSI Nigeria Reproductive Choices, Abuja, Nigeria; Department of New Initiatives (Prof S B Agampodi MD), International Vaccine Institute, Seoul, South Korea; School of Public Health (B O Ahinkorah MPhil), School of Nursing and Midwifery (M Chutiyami PhD), University of Technology Sydney, Sydney, NSW, Australia; College of Medicine (A Ahmad PhD), Shaqra University, Shaqra, Saudi Arabia; School of Medicine and Psychology (D Ahmad PhD), Australian National University, Canberra, ACT, Australia; Public Health Foundation of India, Gandhinagar, India (D Ahmad PhD); Department of Health and Biological Sciences (S Ahmad PhD), Abasyn University, Peshawar, Pakistan; Department of Natural Sciences (S Ahmad PhD), Gilbert and Rose-Marie Chagoury School of Medicine (Prof L Roever PhD), Lebanese American University, Beirut, Lebanon; Institute of Endemic Diseases (A Ahmed MSc), University of Khartoum, Khartoum, Sudan; Swiss Tropical and Public Health Institute (A Ahmed MSc), University of Basel, Basel, Switzerland; Department of Biosciences (H Ahmed PhD), COMSATS Institute of Information Technology, Islamabad, Pakistan; Department of Geriatric and Long Term Care (H Al Hamad MD, B Sathian PhD), Rumailah Hospital (H Al Hamad MD), Hamad Medical Corporation, Doha, Qatar; School of Medicine (Y Al-Ajlouni MD), New York Medical College, Valhalla, NY, USA; Department of Epidemiology (Y Al-Ajlouni MD), Departments of Psychiatry and Epidemiology (Prof M R Phillips MD), Columbia University, New York, NY, USA; School of Nursing (R M Al-amer PhD), Yarmouk University, Irbid, Jordan; School of Nursing and Midwifery (R M Al-amer PhD), Department of Engineering (G R Naik PhD), Western Sydney University, Sydney, NSW, Australia; Department of Community and Mental Health (Prof M Albashtawy PhD), Al al-Bayt University, Mafraq, Jordan; Division of Gastroenterology and Hepatology (W A Aldhaleei MD, A S Bhagavathula PhD), Mayo Clinic, Jacksonville, FL, USA; Center for Biotechnology and Microbiology (S S Ali PhD), University of Swat, Swat, Pakistan; Department of Geography (W Ali PhD), Sultan Qaboos University, Muscat, Oman; Department of Physical Therapy and Rehabilitation Sciences (Prof M A Alomari PhD), Department of Rehabilitation Sciences and Physical Therapy (Prof M A Alomari PhD), Department of Public Health (Prof K A Kheirallah PhD), Jordan University of Science and Technology, Irbid, Jordan; Department of Emergency Medicine (M A Alsabri MD), Sana'a University, Sanaa, Yemen; Pediatric Emergency Medicine Department (M A Alsabri MD), Drexel University, Philadelphia, PA, USA; Research Group in Health Economics (Prof N Alvis-Guzman PhD), Universidad de Cartagena (University of Cartagena), Cartagena, Colombia; Research Group in Hospital Management and Health Policies (Prof N Alvis-Guzman PhD), Universidad de la Costa (University of the Coast), Barranquilla, Colombia; Department of Medical

Sciences (Prof Y M Al-Worafi PhD), Azal University for Human Development, Sana'a, Yemen; Department of Clinical Sciences (Prof Y M Al-Worafi PhD), University of Science and Technology of Fujairah, Fujairah, United Arab Emirates; Department of Radiology and Radiological Science (A Amindarolzarbi MD), Department of Anesthesia and Critical Care Medicine (S Boppana MD), Department of International Health (H Zhang MS), Johns Hopkins University, Baltimore, MD, USA; Spiritual Health Research Center (S Amiri PhD), Baqiyatallah University of Medical Sciences, Tehran, Iran; Department of Statistics and Econometrics (Prof T Andrei PhD, I Petcu PhD), Bucharest University of Economic Studies, Bucharest, Romania; Regenerative Medicine, Organ Procurement and Transplantation Multi-disciplinary Center (S Anvari MD), Department of Social Medicine and Epidemiology (A Feizkhah MD), Guilan University of Medical Sciences, Rasht, Iran; Health Management and Economics Research Center (J Arabloo PhD), School of Medicine (M Bastan MD), Center for Technology and Innovation in Cardiovascular Informatics (S Shool MD), Iran University of Medical Sciences, Tehran, Iran; College of Art and Science (D Areda PhD), Ottawa University, Surprise, AZ, USA; School of Life Sciences (D Areda PhD), Arizona State University, Tempe, AZ, USA; Institute for Biomedical Problems (A A Artamonov PhD), Russian Academy of Sciences, Moscow, Russia; Pioneer Journal of Biostatistics and Medical Research (PJBMR), Pakistan, Pakistan (T Ashraf PhD); Department of Immunology (S Athari PhD), Department of Critical Care and Emergency Nursing (N Hanifi PhD), Zanjan University of Medical Sciences, Zanjan, Iran; Faculty of Nursing (M M W Atout PhD), Philadelphia University, Amman, Jordan; ASIDE Healthcare, Lewes, DE, USA (A Y Azzam MD); Faculty of Medicine (A Y Azzam MD), October 6 University, 6th of October City, Egypt; Department of Forensic Science (A D Badiye PhD, N Kapoor PhD), Government Institute of Forensic Science Nagpur, Nagpur, India; Rashtrasant Tukadoji Maharaj Nagpur University, Nagpur, India (A D Badiye PhD); Department of Nursing (N Baghcheghi PhD), Social Determinants of Health Research Center (H Koohestani PhD), Saveh University of Medical Sciences, Saveh, Iran; School of Medicine (S Bahramian MD), Isfahan University of Medical Sciences, Isfahan, Iran; Department of Hypertension (Prof M Banach PhD), Medical University of Lodz, Lodz, Poland; Polish Mothers' Memorial Hospital Research Institute, Lodz, Poland (Prof M Banach PhD); School of Psychology (Prof S L Barker-Collo PhD), University of Auckland, Auckland, New Zealand; Heidelberg Institute of Global Health (HIGH) (Prof T W Bärnighausen MD), Heidelberg University, Heidelberg, Germany; T.H. Chan School of Public Health (Prof T W Bärnighausen MD), Harvard Business School (F Caetano dos Santos PhD), Harvard Medical School (D Lee PhD), Department of Health Policy and Oral Epidemiology (Z S Natto DrPH), Harvard University, Boston, MA, USA; Department of Public and Environmental Health (A Barrow MPH), University of The Gambia, Banjul, The Gambia; Department of Epidemiology (A Barrow MPH), College of Medicine (M J Diaz BS), University of Florida, Gainesville, FL, USA; Health Information Management (A Bashiri PhD), Health Human Resources Research Center (M Bayati PhD), Non-communicable Disease Research Center (S G Sepanlou MD), Shiraz University of Medical Sciences, Shiraz, Iran; Department of Animal Sciences (H A Bashiru PhD), Obafemi Awolowo University, Ile-Ife, Nigeria; Non-communicable Diseases Research Center (M Bastan MD, M Rashidi MD), Iranian Research Center for HIV/AIDS (IRCHA) (O Dadras PhD), Multiple Sclerosis Research Center (S Eskandarieh PhD), Sina Trauma and Surgery Research Center (M Hassan Zadeh Tabatabaei MD, Prof V Rahimi-Movaghar MD, F Sadeghian PhD, Prof P Salamati MD, S Shool MD), Department of Physical Medicine and Rehabilitation (S Rahimibarghani MD), Iranian National Center for Addiction Studies (Prof A Rahimi-Movaghar MD), Non-Communicable Diseases Research Center (S Saeedi Moghaddam MSc), Digestive Diseases Research Institute (S G Sepanlou MD), Department of Neurology (M Shafie MD), Sina Hospital (A Sharifan PharmD), Tehran University of Medical Sciences, Tehran, Iran; Department of Medical Education (K Batra PhD), School of Public Health

(R Batra MS), University of Nevada Las Vegas, Las Vegas, NV, USA; IT Department (R Batra MS), Coforge, Georgia, GA, USA; Department of Epidemiology and Psychosocial Reseach (C Benjet PhD), Department of Epidemiology and Psychosocial Research (Prof G Borges DSc, R A Gutiérrez PhD), Ramón de la Fuente Muñiz National Institute of Psychiatry, Mexico City, Mexico; Department of Epidemiology and Health Promotion (Prof H Benzian PhD), Department of Population Health (S Jaka MD), Institute for Excellence in Health Equity (M Kumar PhD), New York University, New York, NY, USA; Department of Public Health, Experimental and Forensic Medicine (P Bertuccio PhD), University of Pavia, Pavia, Italy; Department of Public Health (A S Bhagavathula PhD), North Dakota State University, Fargo, ND, USA; The Translational and Clinical Institute (P Bhattacharjee MD), Newcastle University, Newcastle upon Tyne, UK; Department of Clinical Medicine (P Bhattacharjee MD), Cambridge University Hospitals NHS Foundation Trust, Cambridge, UK; Department of Emergency Medicine (C B Bills MD), University of Colorado, Aurora, CO, USA; Department of Emergency Medicine (C B Bills MD), University of California San Francisco, San Francisco, CA, USA; Internal Medicine Department (H Borhany MD), School of Medicine (D J Ghadimi MD), Social Determinants of Health Research Center (A Kolahi MD, A Nikoobar BSc, M Rashidi MD), Student Research Committee (M Rahmanian MD), Department of Health (M Safari PhD), Department of Anesthesiology (S Salimi MD), Shahid Beheshti University of Medical Sciences, Tehran, Iran; School of Pharmacy (Prof Y Bustanji PhD), The University of Jordan, Amman, Jordan; Department of Basic Biomedical Sciences (Prof Y Bustanji PhD), Department of Clinical Sciences (Prof M M Ramadan PhD), College of Medicine (Prof B A Saddik PhD), Clinical Sciences Department (N Saheb Sharif-Askari PhD), College of Pharmacy (Prof M H Semreen PhD), Research Institute of Medical & Health Sciences (Prof M H Semreen PhD), University of Sharjah, Sharjah, United Arab Emirates; Division of Country Health Policies and Systems (CPS) (G Castelpietra PhD), World Health Organisation, -, Italy; Mental Health Flagship (G Castelpietra PhD), World Health Organization (WHO), Copenhagen, Denmark; Department of Psychiatry (A Caye PhD, Prof M F P Peres MD), University of São Paulo, São Paulo, Brazil; Department of Psychiatry (A Caye PhD), Department of Psychiatry and Legal Medicine (Prof G A Salum PhD), Federal University of Rio Grande do Sul, Porto Alegre, Brazil; College of Public Health, Medical, and Veterinary Sciences (M Cenderadewi MPHTM), College of Medicine, Dentistry and Public Health (Prof R C Franklin PhD), James Cook University, Townsville, QLD, Australia; Department of Public Health (M Cenderadewi MPHTM), University of Mataram, Mataram, Indonesia; Clinical Nutrition Department (R M Chandika PhD), Department of Public Health (K Y Ghailan PhD, P Rajpoot PhD), Jazan University, Jazan, Saudi Arabia; Department of Anesthesiology and Perioperative Medicine (E K Chandrasekar MD), University of Rochester, Rochester, NY, USA; Department of Public Health (P Charalampous PhD), Erasmus University Medical Center, Rotterdam, Netherlands; Centre for Health Management and Policy Research (Y Chen MPH), National Health Commission of China (NHC) Key Laboratory of Health Economics and Policy Research (Y Chen MPH), Shandong University, Jinan, China; Concord Institute of Academic Surgery (R Chimoriya PhD), Sydney Local Health District, Sydney, NSW, Australia; Concord Clinical School (R Chimoriya PhD), University of Sydney, Sydney, NSW, Australia (S R Okeke PhD); Centre for Research Impact & Outcome (H Chopra PhD), Chitkara University, Rajpura, India; Department of Community Medicine (Prof S G Choudhari MD), Jawaharlal Nehru Medical College, Wardha, India; The Interdisciplinary Research Group on Biomedicine and Health (D Chu PhD), Faculty of Applied Sciences (D Chu PhD), VNU International School (VNUIS), Hanoi, Viet Nam; Department of Paediatric Surgery (I S Chukwu B.Med.Sc.), Federal Medical Centre, Umuahia, Nigeria; Department of Psychology (R G Cowden PhD), University of the Free State, Park West, South Africa; School of Public Health (B A Dachew PhD, T R Miller PhD), Curtin University, Perth, WA, Australia; Department of Epidemiology (B A Dachew PhD),

School of Nursing (H B Netsere MSc), University of Gondar, Gondar, Ethiopia; Research Center for Child Psychiatry (O Dadras PhD), University of Turku, Turku, Finland; Institute for Health Sciences (Prof K Dalal PhD), Mid Sweden University, Sundsvall, Sweden; Public Health Foundation of India, Gurugram, India (Prof L Dandona MD, Prof R Dandona PhD, G Kumar PhD, A Pandey PhD); Department of Public Health (S D Darcho MPH), Department of Psychiatry (M T Walde MSc), Haramaya University, Harar, Ethiopia; Environmental Health (R Darvishi Cheshmeh Soltani PhD), Arak University of Medical Sciences, Arak, Iran; Department of Population and Development (C A Dávila-Cervantes PhD), Latin American Faculty of Social Sciences Mexico, Mexico City, Mexico; Department of Legal Medicine, Psychiatry and Pathology (A de la Torre-Luque PhD), Universidad Complutense de Madrid (Complutense University of Madrid), Madrid, Spain; Medical College (S Debopadhaya BS), Albany Medical College, Albany, NY, USA; National Drug and Alcohol Research Centre (Prof L Degenhardt PhD), International Centre for Future Health Systems (J Lin PhD), School of Medicine (Prof P K Maulik PhD), Centre for Social Research in Health (S R Okeke PhD), School of Population Health (Prof B A Saddik PhD), University of New South Wales, Sydney, NSW, Australia; School of Medicine (I Delgado-Enciso DSc), University of Colima, Colima, Mexico; Department of Research (I Delgado-Enciso DSc), State Cancerology Institute of Colima, IMSS-BIENESTAR, Colima, Mexico; Department of Forensic Medicine (E Dervišević PhD), University of Sarajevo, Sarajevo, Bosnia and Herzegovina; Health Science Center (D Dongarwar MS), University of Texas, Houston, TX, USA; Independent Consultant, South Plainfield, NJ, USA (O P Doshi MS); Kasturba Medical College Mangalore (M Rao MD), Manipal Academy of Higher Education, Manipal, India (H L Dsouza MD); Department of Forensic Medicine and Toxicology (H L Dsouza MD), Kasturba Medical College, Mangalore, Mangalore, India; Postgraduate Program in Health Sciences (S C Dumith PhD), Federal University of Rio Grande, Rio Grande, Brazil; Faculty of Science and Humanities (S Duraisamy PhD), SRM Institute of Science and Technology, Kattankulathur, India; Department of Psychiatry (E Eboreime PhD), Dalhousie University, Halifax, NS, Canada; Department of Psychiatry (E Eboreime PhD), University of Alberta, Edmonton, AB, Canada; Advanced Nursing Department (F Efendi PhD), Universitas Airlangga (Airlangga University), Surabaya, Indonesia; Faculty of Science and Health (M Ekholuenetale PhD), University of Portsmouth, Hampshire, UK; Almoosa College of Health Sciences, Al Ahsa, Saudi Arabia (R A El Arab PhD); Faculty of Medicine (M Elhadi MD), University of Tripoli, Tripoli, Libya; Houston Methodist Hospital, Houston, TX, USA (M Elhadi MD); Department of Neuropsychiatry (Prof G ELNahas MD), Department of Entomology (A M Samy PhD), Medical Ain Shams Research Institute (MASRI) (A M Samy PhD), Ain Shams University, Cairo, Egypt; Executive Committee (Prof G ELNahas MD), International Association for Women Mental Health, Potomac, MD, USA; Department of Pediatrics (C Eltaha MD), University of Texas, Dallas, TX, USA; Department of Research (S E Emdadul Haque PhD), UChicago Research Bangladesh, Dhaka, Bangladesh; Department of Oral Biology (A Fahim PhD), Riphah International University, Islamabad, Pakistan (Z Z Piracha PhD); Department of Psychology (A Faro PhD), Federal University of Sergipe, São Cristóvão, Brazil; School of Engineering (A Fatehizadeh PhD), Edith Cowan University, Joondalup, WA, Australia; Department of Biology and Medicine (P Fazeli MSc), Brown University, Providence, RI, USA; Department of Infectious Diseases and Public Health (G Fekadu PhD), City University of Hong Kong, Hong Kong, China; Department of Pharmacy (G Fekadu PhD), Wollega University, Nekemte, Ethiopia; Department of Social Sciences (Prof N Ferreira PhD), University of Nicosia, Nicosia, Cyprus; Institute of Public Health (F Fischer PhD), Charité Medical University Berlin, Berlin, Germany; Graduate Institute of Injury Prevention and Control (N Y Fridayani MSc), International Master Program for Translational Science (H Huynh BS), Taipei Medical University, Taipei, Taiwan; Department of Oral Biology and Experimental Dental Research (M Gajdács PhD), University of Szeged,

Szeged, Hungary; Department of Community Medicine and Family Medicine (A P Gandhi MD), All India Institute of Medical Sciences, Nagpur, India; School of Public Health (B Ganesan PhD), Institute of Health &Managment, Australia @ Powered by Arizona State University,USA, Melbourne, VIC, Australia; Department of Midwifery (M W Gebregergis MSc), Department of Medical Laboratory Sciences (H N Meles MSc), Adigrat University, Adigrat, Ethiopia; Department of Environmental Health (M Gebrehiwot DSc), Wollo University, Dessie, Ethiopia; College of Medicine and Public Health (T G Gebremeskel PhD, G R Naik PhD), Flinders University, Adelaide, SA, Australia; College of Medicine and Public Health (T G Gebremeskel PhD), Department of Nursing (G M Tsegay MSc), Aksum University, Aksum, Ethiopia; Department of Medical Laboratory Science (M Getie MSc), Addis Ababa University, Addis Ababa, Ethiopia; Center of Health Management (K Y Ghailan PhD), Aden University, Aden, Yemen; School of Public Health (A Ghashghaee BSc), Qazvin University of Medical Sciences, Qazvin, Iran; Department of Radiology (A Gholamrezanezhad MD), University of Southern California, Los Angeles, CA, USA; Department of Genetics (P Goleij MSc), Sana Institute of Higher Education, Sari, Iran; Universal Scientific Education and Research Network (USERN) (P Goleij MSc), Kermanshah University of Medical Sciences, Kermanshah, Iran; Department of Dermatology (A Grada MD), Department of Neonatology (I Qattea MD), Case Western Reserve University, Cleveland, OH, USA; Institute of Public Health (Prof M Grivna PhD), United Arab Emirates University, Al Ain, United Arab Emirates; Department of Public Health and Preventive Medicine (Prof M Grivna PhD), Charles University, Prague, Czech Republic; Department of Epidemiology and Biostatistics (S Guan MD), Anhui Medical University, Hefei, China; Department of Thoracic Surgery (S Gulati MD), Cleveland Clinic, Cleveland, OH, USA; Department of Toxicology (S Gupta MSc), Shriram Institute for Industrial Research, Delhi, India; Department of Biomedical Gerontology (R S Gutiérrez-Murillo PhD), Pontifical Catholic University of Rio Grande do Sul, Porto Alegre, Brazil; Department of Pharmaceutical Technology (I Hasan M.Pharm), University of Dhaka, Dhaka, Bangladesh; Community-Oriented Nursing Midwifery Research Center (M Heidari PhD), Modeling in Health Research Center (A Mohammadian-Hafshejani PhD), Department of Health in Disasters and Emergencies (R Sheikhi B.Hlth.Sci), Shahrekord University of Medical Sciences, Shahrekord, Iran; Department of Medicine (M Hemmati MD), MedStar Health, Washington, DC, USA; Department of Medicine (M Hemmati MD), Georgetown University, Washington, DC, USA; School of Dentistry (N Hoan DDS), Hanoi Medical University, Hanoi, Viet Nam; School of Computer Science (Prof M Hosseinzadeh PhD), Duy Tan University, Da Nang, Viet Nam; Jadara University Research Center (Prof M Hosseinzadeh PhD), Jadara University, Irbid, Jordan; Department of Legal Medicine and Bioethics (Prof S Hostiuc PhD), Department of General Surgery (B Socea PhD), Carol Davila University of Medicine and Pharmacy, Bucharest, Romania; Department of Clinical Legal Medicine (Prof S Hostiuc PhD), National Institute of Legal Medicine Mina Minovici, Bucharest, Romania; Faculty of Medicine (J Huang MD), Jockey Club School of Public Health and Primary Care (C Zhong PhD), The Chinese University of Hong Kong, Hong Kong, China; West Africa RCC (O S Ilesanmi PhD), Africa Centre for Disease Control and Prevention, Abuja, Nigeria; Department of Community Medicine (O S Ilesanmi PhD), University College Hospital, Ibadan, Ibadan, Nigeria; Faculty of Medicine (I M Ilic PhD, Prof M M Santric-Milicevic PhD), School of Public Health and Health Management (Prof M M Santric-Milicevic PhD), University of Belgrade, Belgrade, Serbia; Faculty of Medical Sciences (Prof M D Ilic PhD), University of Kragujevac, Kragujevac, Serbia; Faculty of Health and Life Sciences (A Inok PhD), University of Exeter, Exeter, UK; College of Medicine and Medical Sciences (H Jahrami PhD), Arabian Gulf University, Manama, Bahrain; Ministry of Health, Manama, Bahrain (H Jahrami PhD); Department of Environmental Engineering (Prof R Jalilzadeh Yengejeh PhD), Islamic Azad University, Ahvaz, Iran; Department of Public Health (Z Ji MMed), Tongji University,

Shanghai, China; School of Biology and Engineering (School of Health Medicine Modern Industry) (S Jin MPH), Guizhou Medical University, Guiyang, China; Department of Community Medicine (N Joseph MD, R Motappa MD), Department of Forensic Medicine and Toxicology (Prof J Padubidri MD, Prof P Rastogi MD, P H Shetty MD), Manipal College of Dental Sciences, Mangalore (Prof P K Shetty MDS), Manipal Academy of Higher Education, Mangalore, India; Department of Economics (C E Joshua BSc), National Open University, Benin City, Nigeria; Department of Family Medicine and Public Health (J J Jozwiak PhD), University of Opole, Opole, Poland; School of Public Health (Z Kabir PhD), University College Cork, Cork, Ireland; Department of Oral and Maxillofacial Pathology (V Kadashetti MDS), Department of Public Health Dentistry (Prof S KM Shivakumar MD), Krishna Vishwa Vidyapeeth (Deemed to be University), Karad, India; Faculty of Dentistry (K K Kanmodi MPH), University of Puthisastra, Phnom Penh, Cambodia; Office of the Executive Director (K K Kanmodi MPH), Cephas Health Research Initiative Inc, Ibadan, Nigeria; The Hansjörg Wyss Department of Plastic and Reconstructive Surgery (R S Kantar MD), NYU Langone Health, New York, NY, USA; Cleft Lip and Palate Surgery Division (R S Kantar MD), Global Smile Foundation, Norwood, MA, USA; School of Health Professions and Human Services (I M Karaye MD), Hofstra University, Hempstead, NY, USA; Department of Anesthesiology (I M Karaye MD), Montefiore Medical Center, Bronx, NY, USA; Burns and Plastic Surgery (S Karmakar M.Ch.), Department of Community Medicine and Family Medicine (Prof P Raghav MD), Department of Pharmacology (S Singh MD), All India Institute of Medical Sciences, Jodhpur, India; Public Health Foundation of India, New Delhi, India (H Kaur MPH); Centre for Adolescent Health (J A Kerr PhD), Murdoch Childrens Research Institute, Parkville, VIC, Australia; Department of Psychological Medicine (J A Kerr PhD), University of Otago, Christchurch, New Zealand; Amity Institute of Forensic Sciences (H Khajuria PhD, B P Nayak PhD), Amity University, Noida, India; Natural and Medical Sciences Research Center (A Khan PhD), School of Pharmacy (A K Philip PhD), University of Nizwa, Nizwa, Oman; College of Health, Wellbeing and Life Sciences (Prof K Khatab PhD), Sheffield Hallam University, Sheffield, UK; College of Arts and Sciences (Prof K Khatab PhD), Ohio University, Zanesville, OH, USA; Graduate School of Public Health (K Kim PhD), Kosin University, Busan, South Korea; Broad Institute of MIT and Harvard, Cambridge, MA, USA (M Kim MD); Center for Global Health (D Lee PhD), Department of Psychiatry (R T Liu PhD), Massachusetts General Hospital, Boston, MA, USA (M Kim MD); Department of Forensic Medicine and Toxicology (V Krishna MD), Pondicherry University, Puducherry, India; University of Environment and Sustainable Development, Somanya, Ghana (N Kugbey PhD); Research and Publication Activity Division (M Kulimbet MSc), Atchabarov Scientific-Research Institute of Fundamental and Applied Medicine (A Zhumagaliuly MD), Kazakh National Medical University, Almaty, Kazakhstan; Center of Medicine and Public Health (M Kulimbet MSc), Asfendiyarov Kazakh National Medical University, Almaty, Kazakhstan; Department of Psychiatry (M Kumar PhD), University of Nairobi, Nairobi, Kenya; Public Health, School of Medicine and Dentistry (S Kundu MPH), Griffith University, Gold Coast, QLD, Australia; Clinical Research Center (V Kytö MD), Turku University Hospital, Turku, Finland; Heart Center (V Kytö MD), University of Turku and Turku University Hospital, Turku, Finland; Unidad de Genética y Salud Pública (Prof I Landires MD), Instituto de Ciencias Médicas, Las Tablas, Panama; Ministry of Health (Prof I Landires MD), Hospital Joaquín Pablo Franco Sayas, Las Tablas, Panama; Faculty of Medicine (N Le MD), Department of General Medicine (V T Nguyen MD), Department of Internal Medicine (T H Tran MD), University of Medicine and Pharmacy at Ho Chi Minh City, Ho Chi Minh City, Viet Nam; Department of Cardiovascular Research (N Le MD), Methodist Hospital, Merrillville, IN, USA; Department of Family Medicine (W Lee PhD), University of Texas Medical Branch, Galveston, TX, USA; Department of Preventive Medicine (Prof Y Lee PhD), Department of Health Policy and Management (S Park PhD), Department of Medicine (J Sharifi Rad PhD),

Korea University, Seoul, South Korea; Department of Psychiatry (R T Liu PhD), Harvard Medical School, Boston, MA, USA; One Health Research Group (J López-Gil PhD), Universidad de Las Américas (University of the Americas), Quito, Ecuador; School of Medicine (Prof G Lucchetti PhD), Federal University of Juiz de Fora, Juiz de Fora, Brazil; Centre for Public Health and Wellbeing (Z Ma PhD), University of the West of England, Bristol, UK; Department of Forensic Medicine (Prof V Maled MD), Shri Dharmasthala Manjunatheshwara University, Dharwad, India; Rama Medical College Hospital and Research Centre, Uttar Pradesh, India (K Malhotra MBBS); Institute of Applied Health Research (K Malhotra MBBS), University of Birmingham, Birmingham, UK; Rabigh Faculty of Medicine (Prof A Malik PhD), Department of Dental Public Health (Z S Natto DrPH), King Abdulaziz University, Jeddah, Saudi Arabia; University Health Services (A M Marconi MD), University of Wisconsin- Madison, Madison, WI, USA; Centro de Estudio e Investigación para la prevención y el tratamiento de las adicciones (Center for the Study and Investigation of Addiction Prevention and Treatment) (A M Marconi MD), University of Buenos Aires, Buenos Aires, Argentina; Department of Non-communicable Diseases and Mental Health (R Martinez-Piedra BSc), Pan American Health Organization, Washington, DC, USA; Faculty of Humanities and Health Sciences (Prof R R Marzo MD), Curtin University, Sarawak, Malaysia; Jeffrey Cheah School of Medicine and Health Sciences (Prof R R Marzo MD), Monash University, Subang Jaya, Malaysia; Department of Anatomy and Developmental Biology (Y Mathangasinghe PhD), Monash University, Clayton, VIC, Australia; Department of Anatomy, Genetics and Biomedical Informatics (Y Mathangasinghe PhD), University of Colombo, Colombo, Sri Lanka; Research Division (Prof P K Maulik PhD), The George Institute for Global Health, New Delhi, India; Division of Forensic Medicine (Prof R G Menezes MD), Imam Abdulrahman Bin Faisal University, Dammam, Saudi Arabia; Comprehensive Cancer Center (T J Meretoja MD), Helsinki University Hospital, Helsinki, Finland; University of Helsinki, Helsinki, Finland (T J Meretoja MD); University Centre Varazdin (T Mestrovic PhD), University North, Varazdin, Croatia; National Cancer Registry (I Michalek PhD), Department of Pathology (I Michalek PhD), Maria Sklodowska-Curie National Research Institute of Oncology, Warsaw, Poland; Pacific Institute for Research & Evaluation, Beltsville, MD, USA (T R Miller PhD); Department of Hospital Administration (M Mirza MD), Department of Community Medicine and Family Medicine (S S Sahoo MD), Department of Radiodiagnosis (P Singh MD), All India Institute of Medical Sciences, Bathinda, India; National Data Management Center for Health (A Misganaw PhD), Ethiopian Public Health Institute, Addis Ababa, Ethiopia; Department of Forensic Medicine and Toxicology (C Mittal MD), All India Institute of Medical Sciences, Patna, India; Center for Brain and Health (A Z Mohamed PhD), New York University Abu Dhabi, Abu Dhabi, United Arab Emirates; Molecular Biology Unit (N S Mohamed MSc), Bio-Statistical and Molecular Biology Department (N S Mohamed MSc), Sirius Training and Research Centre, Khartoum, Sudan; Institute of Clinical Physiology (S Molinaro PhD), National Research Council, Pisa, Italy; Clinical Epidemiology and Public Health Research Unit (L Monasta DSc), Burlo Garofolo Institute for Maternal and Child Health, Trieste, Italy; Faculty of Medicine (A Moodi Ghalibaf MD), Birjand University of Medical Sciences, Birjand, Iran; Division of Plastic and Reconstructive Surgery (S D Morrison MD), University of Washington Medical Center, Seattle, WA, USA; School of Medicine (F Mughal FRCGP), Keele University, Keele, UK; Division of Psychology and Mental Health (F Mughal FRCGP), University of Manchester, Manchester, UK; Department of Surgery (F Mulita PhD), General University Hospital of Patras, Patras, Greece; Faculty of Medicine (F Mulita PhD), Department of Emergency Medicine (Prof I Pantazopoulos PhD), University of Thessaly, Larissa, Greece; Department of Community and Global Health (Y Munkhsaikhan MD), The University of Tokyo, Tokyo, Japan; Department of Research Methods (S Muthu PhD), Orthopaedic Research Group, Coimbatore, India; Department of Biotechnology (S Muthu PhD),

Karpagam Academy of Higher Education (Deemed to be University), Coimbatore, India; Department of Psychiatry (W Myung PhD), Seoul National University, Seoul, South Korea; Department of Neuropsychiatry (W Myung PhD), Seoul National University Bundang Hospital, Seongnam, South Korea; Elderly Health Research Center (A Nafei PhD), Research and Academic Institution, Tehran, Iran; Department of Computer Science (P Naghavi MS), University of Illinois, Urbana, IL, USA; Department Health Services Research (G Naik MPH), University of Alabama at Birmingham, Birmingham, AL, USA; Department of Biotechnology (M Naveed PhD), University of Central Punjab, Lahore, Pakistan; Anatomy Department (S Navid PhD), Social Determinants of Health Research Center (S Navid PhD), Gonabad University of Medical Science, Gonabad, Iran; Department of Health Promotion (A Nazri-Panjaki MSc), Zahedan University of Medical Sciences, Zahedan, Iran; College of Medicine and Health Sciences (H B Netsere MSc), Department of Psychiatry (M Tareke MSc), Bahir Dar University, Bahir Dar, Ethiopia; National Centre for Suicide Research and Prevention, Institute of Clinical Medicine (S P Neupane PhD), University of Oslo, Oslo, Norway; Oral Health Centre of Expertise in Rogaland (S P Neupane PhD), Oral Health Center of Expertise in Rogaland, Stavanger, Norway; Department of General Medicine (H H Nguyen MD), Thai Binh University of Medicine and Pharmacy, Thai Binh City, Viet Nam; Department of General Medicine (N N Nguyen MD), Faculty of Medicine (N N Nguyen MD), Pham Ngoc Thach University of Medicine, Ho Chi Minh City, Viet Nam; Department of Surgery (P T Nguyen MD), Danang Family Hospital, Danang, Viet Nam; Hitotsubashi Institute for Advanced Study (HIAS) (P T Nguyen DrPH), Hitotsubashi University, Tokyo, Japan; Institute for Cancer Control (P T Nguyen DrPH), National Cancer Center, Chuo-ku, Japan; Tuberculosis Group (V T Nguyen MD), Oxford University Clinical Research Unit, Viet Nam, Ho Chi Minh City, Viet Nam; National School of Public Health / Mental Health and Public Health (Prof I Noguer PhD), National School of Public Health (A Padron-Monedero PhD), Institute of Health Carlos III, Madrid, Spain; Global Research Institute (Prof S Nomura PhD), Keio University, Tokyo, Japan; Department of Global Health Policy (Prof S Nomura PhD), University of Tokyo, Tokyo, Japan; Department of Paediatrics (C A Nri-Ezedi PhD), Nnamdi Azikiwe University, Awka, Nigeria; Unit of Microbiology and Public Health (V Nuñez-Samudio PhD), Instituto de Ciencias Medicas, Las Tablas, Panama; Department of Public Health (V Nuñez-Samudio PhD), Ministry of Health, Herrera, Panama; Department of Physiology (O J Nzoputam PhD), University of Benin, Edo, Nigeria; Department of Physiology (O J Nzoputam PhD), Benson Idahosa University, Benin City, Nigeria; Department of Applied Economics and Quantitative Analysis (Prof B Oancea PhD), University of Bucharest, Bucharest, Romania; Bioinformatics Department (Prof B Oancea PhD), National Institute of Research and Development for Biological Sciences, Bucharest, Romania; PSSM Data Sciences, Pfizer Research & Development (M Oduro PhD), Pfizer Inc., Groton, CT, USA; Department of Preventive Medicine (Prof I Oh MD), Department of Pediatrics (Prof D Yon MD), Kyung Hee University, Seoul, South Korea; Department of Microbiology (Y Oluwafemi PhD), University of Medical Sciences, Ondo, Ondo, Nigeria; Department of Public Health (S Ong FAMS), Ministry of Health, Bandar Seri Begawan, Brunei; Institute of Health Sciences (S Ong FAMS), Universiti Brunei Darussalam, Bandar Seri Begawan, Brunei; Department of Pharmacotherapy and Pharmaceutical Care (M Ordak PhD), Medical University of Warsaw, Warsaw, Poland; Substance Related Harms Division (H M Orpana PhD), Public Health Agency of Canada, Ottawa, ON, Canada; School of Epidemiology and Public Health (H M Orpana PhD), University of Ottawa, Ottawa, ON, Canada; One Health Global Research Group (Prof E Ortiz-Prado PhD), Universidad de las Americas (University of the Americas), Quito, Ecuador; School of Medicine (U L Osuagwu PhD), Western Sydney University, Bathurst, NSW, Australia; Department of Optometry and Vision Science (U L Osuagwu PhD), University of KwaZulu-Natal, KwaZulu-Natal, South Africa; Department of Mental Health (R F Palma-Alvarez PhD),

Hospital Universitari Vall d'Hebron (CIBERSAM), Barcelona, Spain; Biomedical Network Research Centre on Mental Health (CIBERSAM), Barcelona, Spain (R F Palma-Alvarez PhD); Research Department (A Pandey MPH), Nepal Health Research Council, Kathmandu, Nepal; Research Department (A Pandey MPH), Public Health Research Society Nepal, Kathmandu, Nepal; Department of Emergency Medicine (Prof I Pantazopoulos PhD), University of Bern, Bern, Switzerland; Department of Biomedical Data Science (S Park MD), Department of Radiology (S Ramasamy MD), Stanford University, Stanford, CA, USA; School of Nursing (A Pashaei MSc), University of British Columbia, Vancouver, BC, Canada; Global Health Governance Programme (J Patel BSc), College of Medicine and Veterinary Medicine (G Verras MSc), University of Edinburgh, Edinburgh, UK; School of Dentistry (J Patel BSc), University of Leeds, Leeds, UK; Department of Genetics (S Pawar PhD), Department of Psychiatry (T Rhee PhD), Yale University, New Haven, CT, USA; Australian Institute of Health Innovation (P Peprah MSc), Macquarie University, Sydney, NSW, Australia; International Institute for Educational Planning (IIEP) (Prof M F P Peres MD), Albert Einstein Hospital, São Paulo, Brazil; Shanghai Mental Health Center (Prof M R Phillips MD), Shanghai Jiao Tong University, Shanghai, China; International Center of Medical Sciences Research (Z Z Piracha PhD), International Center of Medical Sciences Research, Islamabad, Pakistan; Department of Humanities and Social Sciences (Prof J Pradhan PhD), National Institute of Technology Rourkela, Rourkela, India; Department of Maternal-Child Nursing and Public Health (E J S Prates BS), Federal University of Minas Gerais, Belo Horizonte, Brazil; Health Sciences Department (D R A Pribadi MSc), Muhammadiyah University of Surakarta, Sukoharjo, Indonesia; Department of Biostatistics, Epidemiology, and Informatics (J Puvvula PhD), University of Pennsylvania, Philadelphia, PA, USA; Department of Cardiology (G Qian MS), Guiqian International General Hospital, Guiyang, China; Department of Medical Oncology (Prof V Radhakrishnan MD), Cancer Institute (W.I.A), Chennai, India; Department of Population Science and Human Resource Development (Prof M Rahman PhD, Prof M Rahman DrPH), University of Rajshahi, Rajshahi, Bangladesh; Institute of Health and Wellbeing (Prof M Rahman PhD), Federation University Australia, Berwick, VIC, Australia; School of Nursing and Midwifery (Prof M Rahman PhD), La Trobe University, Melbourne, VIC, Australia; Department of Cardiology (Prof M M Ramadan PhD), Mansoura University, Mansoura, Egypt; Department of Forensic Medicine and Toxicology (S Rani MD), Department of Oral and Maxillofacial Surgery (C S N PhD), Jagadguru Sri Shivarathreeswara University, Mysore, India; Department of Oral Pathology, Microbiology and Forensic Odontology (S Rao MDS), Sharavathi Dental College and Hospital, Shimogga, India; Department of Family Medicine (Prof D Rathish PhD), Department of Community Medicine (N D Wickramasinghe MD), Rajarata University of Sri Lanka, Anuradhapura, Sri Lanka; WHO Collaborating Centre for Public Health Education and Training (D L Rawaf MD), Department of Primary Care and Public Health (C Tabche MSc), Imperial College London, London, UK; Inovus Medical, St Helens, UK (D L Rawaf MD); Melbourne School of Population and Global Health (L Reifels PhD), University of Melbourne, Melbourne, VIC, Australia; Department of Epidemiology and Biostatistics (Prof M Rezaeian PhD), Rafsanjan University of Medical Sciences, Rafsanjan, Iran; Department of Public Health Sciences (T Rhee PhD), University of Connecticut, Farmington, CT, USA; Department of Surgery (J Rickard MD), University of Minnesota, Minneapolis, MN, USA; Department of Surgery (J Rickard MD), University Teaching Hospital of Kigali, Kigali, Rwanda; Department of Clinical Research (Prof L Roever PhD), University of Sao Paulo, Ribeirão Preto, Brazil; Department of Public Health (M Rony MPH), Bangladesh Open University, Gazipur, Bangladesh; Center for Health Related Social and Behavioral Sciences Research (F Sadeghian PhD), Shahroud University of Medical Sciences, Shahroud, Iran; Department of Pharmaceutical Chemistry (Prof M Saeb PhD), International Medical University, Gdańsk, Poland; Operational Research Center in Healthcare (Prof U

Saeed PhD), Near East University (NEU), Nicosia Cyprus, Türkiye; International Center of Medical Sciences Research (ICMSR), Islamabad, Pakistan (Prof U Saeed PhD); Kiel Institute for the World Economy, Kiel, Germany (S Saeedi Moghaddam MSc); Department of Psychosocial Science (Prof D Sagoe PhD), University of Bergen, Bergen, Norway; Department of Analytical & Applied Economics (P Sahoo MA), Department of Analytical and Applied Economics (C Swain MPhil), Utkal University, Bhubaneswar, India; College of Nursing (D Salihu PhD), Jouf University, Jouf, Saudi Arabia; Department of Global Initiatives (Prof G A Salum PhD), Child Mind Institute, New York, NY, USA; Department of Community Health Sciences (S Sameen MSc), Aga Khan University, Karachi, Pakistan; Department of Urban Planning and Design (C Sarkar PhD), Centre for Suicide Research and Prevention (Prof P Yip PhD), Department of Social Work and Social Administration (Prof P Yip PhD), University of Hong Kong, Hong Kong, China; Department of Oral Pathology and Microbiology (Prof G S Sarode PhD, Prof S C Sarode PhD), Dr. D. Y. Patil Vidyapeeth, Pune (Deemed to be University), Pune, India; Faculty of Health & Social Sciences (B Sathian PhD), Bournemouth University, Bournemouth, UK; Department of Medical Statistics (M Šekerija PhD), University of Zagreb, Zagreb, Croatia; Department of Epidemiology and Prevention of Chronic Noncommunicable Diseases (M Šekerija PhD), Croatian Institute of Public Health, Zagreb, Croatia; Institute of Molecular Biology and Biotechnology (S Shahid PhD), Research Centre for Health Sciences (RCHS) (S Shahid PhD), The University of Lahore, Lahore, Pakistan; Department of Cardiology (M Vinayak MD), Icahn School of Medicine at Mount Sinai, New York, NY, USA (A Shaikh MD); Institute for Critical Care Medicine (A Shaikh MD), Mount Sinai Health System, New York, NY, USA; Independent Consultant, Karachi, Pakistan (M A Shaikh MD); Department for Evidence-based Medicine and Evaluation (A Sharifan PharmD), University for Continuing Education Krems, Krems, Austria; Department of Hemato-oncology (A Sharma MD), Fortis Hospital, Noida, India; Institute of Forensic Science & Criminology (V Sharma PhD), Panjab University, Chandigarh, India; K S Hegde Medical Academy (Prof M Shetty MD), Nitte University, Mangalore, India; Department of Experimental Research (V Shivarov PhD), Medical University Pleven, Pleven, Bulgaria; Department of Genetics (V Shivarov PhD), Sofia University "St. Kliment Ohridiski", Sofia, Bulgaria; Department of Human Genetics (P Singh PhD), Punjabi University, Patiala, India; Department of Surgery (B Socea PhD), "Sf. Pantelimon" Emergency Clinical Hospital Bucharest, Bucharest, Romania; SAMRC Unit on Risk and Resilience in Mental Disorders (Prof D J Stein FRCPC), University of Cape Town, Cape Town, South Africa; Department of Psychiatry (M B Stein MD), University of California San Diego, La Jolla, CA, USA; Rural Health Research Institute (Prof J Sun PhD), Charles Sturt University, Orange, NSW, Australia; Institute of Integrated Intelligence and Systems (Prof J Sun PhD), Griffith University, Brisbane, QLD, Australia; Department of Clinical Research and Development (Prof L Szarpak PhD), LUXMED Group, Warsaw, Poland; Collegium Medicul (Prof L Szarpak PhD), John Paul II Catholic University of Lublin, Lublin, Poland; Department of Pharmacology (S T Y MD), All India Institute of Medical Sciences, Deoghar, India; Department of Medical Informatics (S Tabatabaei PhD), Clinial Research Development Unit (S Tabatabaei PhD), Mashhad University of Medical Sciences, Mashhad, Iran; Pediatric Intensive Care Unit (Prof M Temsah MD), King Saud University, Riyadh, Saudi Arabia; Deputy Minister’s Office (C Thum MB BCh BAO), Ministry of Communications, Malaysia, Putrajaya, Malaysia; Department of Allied Health and Human Performance (T Y Tiruye PhD), University of South Australia, Adelaide, SA, Australia; Public Health Department (T Y Tiruye PhD), Debre Markos University, Debre Markos, Ethiopia; Saveetha Dental College and Hospitals (M Tovani-Palone PhD), Saveetha University, Chennai, India; Department of Health (N M Tran MD), Children's Hospital 1, Ho Chi Minh City, Viet Nam; Department of Business Analytics (T H Tran MD), University of Massachusetts Dartmouth, Dartmouth, MA, USA; Molecular Neuroscience Research Center (N Tran Minh Duc MD),

Shiga University of Medical Science, Shiga, Japan; Department of Health Sciences (S J Tromans PhD), University of Leicester, Leicester, UK; Adult Learning Disability Service (S J Tromans PhD), Leicestershire Partnership National Health Service Trust, Leicester, UK; Faculty of Medicine (T T Truyen MD), Tan Tao University, Long An, Viet Nam; Department of Internal Medicine (M Tumurkhuu PhD), Wake Forest University, Winston-Salem, NC, USA; Department of Informatics and Radiology (S Vahdati MD), Mayo Clinic, Rochester, MN, USA; College of Health and Sport Sciences (A G Vaithinathan MSc), University of Bahrain, Zallaq, Bahrain; Argentine Society of Medicine, Buenos Aires, Argentina (Prof P R Valdez M.Ed.); Velez Sarsfield Hospital, Buenos Aires, Argentina (Prof P R Valdez M.Ed.); UKK Institute, Tampere, Finland (Prof T J Vasankari PhD); Faculty of Medicine and Health Technology (Prof T J Vasankari PhD), Tampere University, Tampere, Finland; Department of Medical and Surgical Sciences and Advanced Technologies "GF Ingrassia" (Prof M Veroux PhD), University of Catania, Catania, Italy; Department of Surgery (G Verras MSc), University of Southampton, Southampton, UK; School of Life Course and Population Sciences (Prof Y Wang PhD), King's College London, London, UK; UCL Great Ormond Street Institute of Child Health (J L L Ward PhD), University College London, London, UK; Department of Research (M W Wojewodzic PhD), Cancer Registry of Norway, Oslo, Norway; Department of Chemical Toxicology (M W Wojewodzic PhD), Norwegian Institute of Public Health, Oslo, Norway; Manipal College of Nursing (R Yesodharan MPhil), Manipal Academy of Higher Education, Udupi, India; Department of Health Management (A Yiğit PhD), Süleyman Demirel Üniversitesi (Süleyman Demirel University), Isparta, Türkiye; Department of Epidemiology (D Yin DrPH), Xuzhou Medical University, Xuzhou, China; Department of Biostatistics (Prof N Yonemoto PhD), University of Toyama, Toyama, Japan; Department of Public Health (Prof N Yonemoto PhD), Juntendo University, Tokyo, Japan; Department of Epidemiology and Biostatistics (Prof C Yu PhD), Wuhan University, Wuhan, China; Research and Development Department (I Zare BSc), Sina Medical Biochemistry Technologies, Shiraz, Iran; Department of Public Health (M G M Zeariya PhD), University of Hail, Hail, Saudi Arabia; Department of Zoology and Entomology (M G M Zeariya PhD), Al-Azhar University, Cairo, Egypt; School of Public Health (H Zhang MS), Peking University, Beijing, China; School of Public Health and Emergency Management (B Zhu PhD), Southern University of Science and Technology, Shenzhen, China.

## Authors' Contributions

### Managing the overall research enterprise

Mohsen Naghavi, Emily Rosenblad, and Eve Wool.

### Writing the first draft of the manuscript

Gregory J Bertolacci, Nicole Davis Weaver, Kevin Ikuta, Mohsen Naghavi, and Emily Rosenblad.

### Primary responsibility for applying analytical methods to produce estimates

Gregory J Bertolacci, Matthew Cunningham, Sama Ghoba, Madeline Moberg, and Mohsen Naghavi.

### Primary responsibility for seeking, cataloguing, extracting, or cleaning data; designing or coding figures and tables

Gregory J Bertolacci, Matthew Cunningham, Sama Ghoba, Chieh Han, Vincent Mougin, and Mohsen Naghavi.

### Providing data or critical feedback on data sources

Yohannes Habtegiorgis Abate, Habeeb Omoponle Adewuyi, Qorinah Estiningtyas Sakilah Adnani, Leticia Akua Adzigbli, Bright Opoku Ahinkorah, Danish Ahmad, Sajjad Ahmad, Ayman Ahmed, Haroon Ahmed,

Hanadi Al Hamad, Yazan Al-Ajlouni, Rasmieh Mustafa Al-amer, Mohammed Albashtawy, Syed Shujait Ali, Waad Ali, Mahmoud A Alomari, Nelson Alvis-Guzman, Alireza Amindarolzarbi, Saeid Anvari, Jalal Arabloo, Tahira Ashraf, Seyyed Shamsadin Athari, Maha Moh'd Wahbi Atout, Ahmed Y Azzam, Ashish D Badiye, Maciej Banach, Till Winfried Bärnighausen, Amadou Barrow, Mohammad-Mahdi Bastan, Gregory J Bertolacci, Akshaya Srikanth Bhagavathula, Priyadarshini Bhattacharjee, Sri Harsha Boppana, Hamed Borhany, Periklis Charalampous, Ritesh Chimoriya, Hitesh Chopra, Dinh-Toi Chu, Matthew Cunningham, Xiaochen Dai, Lalit Dandona, Rakhi Dandona, Samuel Demissie Darcho, Alejandro de la Torre-Luque, Louisa Degenhardt, Ivan Delgado-Enciso, Michael J Diaz, Ojas Prakashbhai Doshi, Haneil Larson Dsouza, Senbagam Duraisamy, Michael Ekholuenetale, Rabie Adel El Arab, Chadi Eltaha, Syed Emdadul Emdadul Haque, Sharareh Eskandarieh, Ayesha Fahim, Ali Fatehizadeh, Alireza Feizkhah, Ginenus Fekadu, Richard Charles Franklin, Balasankar Ganesan, Teferi Gebru Gebremeskel, Molla Getie, Ahmad Ghashghaee, Sama Ghoba, Pouya Goleij, Ayman Grada, Shi-Yang Guan, Sapna Gupta, Erin B Hamilton, Chieh Han, Nasrin Hanifi, Mahgol Sadat Hassan Zadeh Tabatabaei, Mehdi Hemmati, Nguyen Quoc Hoan, Mehdi Hosseinzadeh, Hong-Han Huynh, Segun Emmanuel Ibitoye, Kevin S Ikuta, Olayinka Stephen Ilesanmi, Haitham Jahrami, Shuai Jin, Charity Ehimwenma Joshua, Jacek Jerzy Jozwiak, Zubair Kabir, Vidya Kadashetti, Rami S Kantar, Neeti Kapoor, Harkiran Kaur, Himanshu Khajuria, Khaled Khatab, Min Seo Kim, Shivakumar KM KM Shivakumar, G Anil Kumar, Manasi Kumar, Satyajit Kundu, Ville Kytö, Nhi Huu Hanh Le, Wei-Chen Lee, Stephen S Lim, Zheng Feei Ma, Kashish Malhotra, Agustina Maria Marconi, Roy Rillera Marzo, Ritesh G Menezes, Ted R Miller, Awoke Misganaw, Chaitanya Mittal, Nouh Saad Mohamed, Abdollah Mohammadian-Hafshejani, Ali H Mokdad, Rohith Motappa, Vincent Mougin, Faraz Mughal, Francesk Mulita, Yanjinlkham Munkhsaikhan, Christopher J L Murray, Mohsen Naghavi, Ganesh R Naik, Zuhair S Natto, Muhammad Naveed, Shadan Navid, Biswa Prakash Nayak, Henok Biresaw Netsere, Sudan Prasad Neupane, Hoang Anh Hoang Nguyen, Nhien Ngoc Y Nguyen, Phat Tuan Nguyen, Van Thanh Nguyen, Shuhei Nomura, Bogdan Oancea, Michael Safo Oduro, Yinka Doris Oluwafemi, Sok King Ong, Uchechukwu Levi Osuagwu, Jagadish Rao Padubidri, Anamika Pandey, Ashok Pandey, Jay Patel, Shrikant Pawar, Prince Peprah, Mario F P Peres, Zahra Zahid Piracha, Jalandhar Pradhan, Elton Junio Sady Prates, Dimas Ria Angga Pribadi, Jagadeesh Puvvula, Pankaja Raghav, Afarin Rahimi-Movaghar, Vafa Rahimi-Movaghar, Pushp Lata Rajpoot, Mahmoud Mohammed Ramadan, Shakthi Kumaran Ramasamy, Smitha Rani, Mithun Rao, Sowmya J Rao, Prateek Rastogi, Leonardo Roever, Moustaq Karim Khan Rony, Basema Ahmad Saddik, Umar Saeed, Mehdi Safari, Pragyan Monalisa Sahoo, Giovanni A Salum, Abdallah M Samy, Milena M Santric-Milicevic, Brijesh Sathian, Mohammad H Semreen, Samiah Shahid, Masood Ali Shaikh, Amin Sharifan, Javad Sharifi Rad, Vishal Sharma, Mahabalesh Shetty, Sina Shool, Paramdeep Singh, Chandan Kumar Swain, Lukasz Szarpak, Sree Sudha T Y, Seyyed Mohammad Tabatabaei, Celine Tabche, Chern Choong Thum, Marcos Roberto Tovani-Palone, Nghia Minh Tran, Guesh Mebrahtom Tsegay, Munkhtuya Tumurkhuu, Sanaz Vahdati, Pascual R Valdez, Tommi Juhani Vasankari, Georgios-Ioannis Verras, Theo Vos, Mandaras Tariku Walde, Renjulal Yesodharan, Paul Yip, Dong Keon Yon, Naohiro Yonemoto, Chuanhua Yu, Iman Zare, Mohammed G M Zeariya, and Abzal Zhumagaliuly.

## Developing methods or computational machinery

Gregory J Bertolacci, Matthew Cunningham, Xiaochen Dai, Sama Ghoba, Erin B Hamilton, Chieh Han, Ali H Mokdad, Vincent Mougin, Christopher J L Murray, Mohsen Naghavi, Austin E Schumacher, and Theo Vos,

## Providing critical feedback on methods or results

Yohannes Habtegiorgis Abate, Habeeb Omoponle Adewuyi, Qorinah Estiningtyas Sakilah Adnani, Leticia Akua Adzigbli, Aanuoluwapo Adeyimika Afolabi, Suneth Buddhika Agampodi, Bright Opoku Ahinkorah, Aqeel Ahmad, Danish Ahmad, Sajjad Ahmad, Ayman Ahmed, Haroon Ahmed, Hanadi Al Hamad, Rasmieh Mustafa Al-amer, Mohammed Albashtawy, Syed Shujait Ali, Waad Ali, Mahmoud A Alomari, Mohammed A Alsabri, Nelson Alvis-Guzman, Yaser Mohammed Al-Worafi, Alireza Amindarolzarbi, Sohrab Amiri, Tudorel Andrei, Saeid Anvari, Jalal Arabloo, Demelash Areda, Anton A Artamonov, Tahira Ashraf, Seyyed Shamsadin Athari, Maha Moh'd Wahbi Atout, Ahmed Y Azzam, Ashish D Badiye, Nayereh Baghcheghi, Saeed Bahramian, Maciej Banach, Till Winfried Bärnighausen, Amadou Barrow, Hameed Akande Bashiru, Mohammad-Mahdi Bastan, Kavita Batra, Ravi Batra, Mohsen Bayati, Gregory J Bertolacci, Paola Bertuccio, Akshaya Srikanth Bhagavathula, Priyadarshini Bhattacharjee, Corey B Bills, Sri Harsha Boppana, Guilherme Borges, Hamed Borhany, Yasser Bustanji, Florentino Luciano Caetano dos Santos, Giulio Castelpietra, Muthia Cenderadewi, Eeshwar K Chandrasekar, Periklis Charalampous, Ritesh Chimoriya, Hitesh Chopra, Sonali Gajanan Choudhari, Dinh-Toi Chu, Isaac Sunday Chukwu, Muhammad Chutiyami, Richard G Cowden, Matthew Cunningham, Berihun Assefa Dachew, Omid Dadras, Xiaochen Dai, Koustuv Dalal, Samuel Demissie Darcho, Reza Darvishi Cheshmeh Soltani, Nicole Davis Weaver, Alejandro de la Torre-Luque, Shayom Debopadhaya, Louisa Degenhardt, Ivan Delgado-Enciso, Emina Dervišević, Michael J Diaz, Deepa Dongarwar, Ojas Prakashbhai Doshi, Haneil Larson Dsouza, Senbagam Duraisamy, Ejemai Eboreime, Ferry Efendi, Michael Ekholuenetale, Rabie Adel El Arab, Muhammed Elhadi, Gihan ELNahas, Chadi Eltaha, Syed Emdadul Emdadul Haque, Sharareh Eskandarieh, Ayesha Fahim, Andre Faro, Ali Fatehizadeh, Patrick Fazeli, Alireza Feizkhah, Ginenus Fekadu, Florian Fischer, Richard Charles Franklin, Ni Kadek Yuni Fridayani, Márió Gajdács, Aravind P Gandhi, Balasankar Ganesan, Miglas Welay Gebregergis, Mesfin Gebrehiwot, Teferi Gebru Gebremeskel, Molla Getie, Delaram J Ghadimi, Khalid Yaser Ghailan, Ahmad Ghashghaee, Ali Gholamrezanezhad, Ayman Grada, Michal Grivna, Shi-Yang Guan, Sapna Gupta, Reyna Alma Gutiérrez, Roberth Steven Gutiérrez-Murillo, Erin B Hamilton, Nasrin Hanifi, Ikramul Hasan, Mahgol Sadat Hassan Zadeh Tabatabaei, Simon I Hay, Mohammad Heidari, Mehdi Hemmati, Nguyen Quoc Hoan, Mehdi Hosseinzadeh, Junjie Huang, Hong-Han Huynh, Segun Emmanuel Ibitoye, Kevin S Ikuta, Olayinka Stephen Ilesanmi, Irena M Ilic, Milena D Ilic, Mustapha Immurana, Arit Inok, Chidozie Declan Iwu, Haitham Jahrami, Sanobar Jaka, Reza Jalilzadeh Yengejeh, Zixiang Ji, Shuai Jin, Nitin Joseph, Charity Ehimwenma Joshua, Jacek Jerzy Jozwiak, Zubair Kabir, Vidya Kadashetti, Kehinde Kazeem Kanmodi, Rami S Kantar, Neeti Kapoor, Ibraheem M Karaye, Himanshu Khajuria, Ajmal Khan, Khaled Khatab, Khalid A Kheirallah, Kwanghyun Kim, Shivakumar KM KM Shivakumar, Ali-Asghar Kolahi, Hamid Reza Koohestani, Varun Krishna, Nuworza Kugbey, Manasi Kumar, Satyajit Kundu, Ville Kytö, Iván Landires, Nhi Huu Hanh Le, Doo Woong Lee, Wei-Chen Lee, Yo Han Lee, Stephen S Lim, Jialing Lin, Richard T Liu, José Francisco López-Gil, Giancarlo Lucchetti, Zheng Feei Ma, Venkatesh Maled, Kashish Malhotra, Ahmad Azam Malik, Agustina Maria Marconi, Ramon Martinez-Piedra, Roy Rillera Marzo, Yasith Mathangasinghe, Pallab K Maulik, Hadush Negash Meles, Ritesh G Menezes, Tomislav Mestrovic, Irmina Maria Michalek, Ted R Miller, Moonis Mirza, Chaitanya Mittal, Madeline E Moberg, Abdalla Z Mohamed, Nouh Saad Mohamed, Abdollah Mohammadian-Hafshejani, Ali H Mokdad, AmirAli Moodi Ghalibaf, Shane Douglas Morrison, Rohith Motappa, Faraz Mughal, Francesk Mulita, Yanjinlkham Munkhsaikhan, Christopher J L Murray, Sathish Muthu, Ayoub Nafei, Mohsen Naghavi, Pirouz Naghavi, Ganesh R Naik, Gurudatta Naik, Zuhair S Natto, Muhammad Naveed, Shadan Navid, Biswa Prakash Nayak, Athare Nazri-Panjaki, Henok Biresaw Netsere, Sudan Prasad Neupane, Hoang Anh Hoang Nguyen, Nhien Ngoc Y Nguyen, Phat Tuan Nguyen, Phuong The

Nguyen, Van Thanh Nguyen, Ali Nikoobar, Shuhei Nomura, Chisom Adaobi Nri-Ezedi, Virginia Nuñez-Samudio, Ogochukwu Janet Nzoputam, Bogdan Oancea, Michael Safo Oduro, Yinka Doris Oluwafemi, Michal Ordak, Heather M Orpana, Uchechukwu Levi Osuagwu, Jagadish Rao Padubidri, Ashok Pandey, Ioannis Pantazopoulos, Seoyeon Park, Sungchul Park, Ava Pashaei, Jay Patel, Shrikant Pawar, Prince Peprah, Mario F P Peres, Ionela-Roxana Petcu, Anil K Philip, Michael R Phillips, Zahra Zahid Piracha, Jalandhar Pradhan, Elton Junio Sady Prates, Jagadeesh Puvvula, Ibrahim Qattea, Gangzhen Qian, Venkatraman Radhakrishnan, Pankaja Raghav, Afarin Rahimi-Movaghar, Vafa Rahimi-Movaghar, Md. Mosfequr Rahman, Mosiur Rahman, Muhammad Aziz Rahman, Mohammad Rahmanian, Pushp Lata Rajpoot, Mahmoud Mohammed Ramadan, Shakthi Kumaran Ramasamy, Smitha Rani, Mithun Rao, Sowmya J Rao, Mohammad-Mahdi Rashidi, Prateek Rastogi, Devarajan Rathish, David Laith Rawaf, Lennart Reifels, Mohsen Rezaeian, Taeho Gregory Rhee, Jennifer Rickard, Leonardo Roever, Moustaq Karim Khan Rony, Basema Ahmad Saddik, Mohammad Reza Saeb, Umar Saeed, Sahar Saeedi Moghaddam, Narjes Saheb Sharif-Askari, Pragyan Monalisa Sahoo, Soumya Swaroop Sahoo, Payman Salamati, Sohrab Salimi, Giovanni A Salum, Sonia Sameen, Abdallah M Samy, Milena M Santric-Milicevic, Chinmoy Sarkar, Gargi Sachin Sarode, Sachin C Sarode, Brijesh Sathian, Mario Šekerija, Mohammad H Semreen, Sadaf G Sepanlou, Mahan Shafie, Samiah Shahid, Ahmed Shaikh, Masood Ali Shaikh, Amin Sharifan, Javad Sharifi Rad, Anupam Sharma, Vishal Sharma, Rahim Ali Sheikhi, Sina Shool, Paramdeep Singh, Puneetpal Singh, Murray B Stein, Jing Sun, Chandan Kumar Swain, Lukasz Szarpak, Sree Sudha T Y, Seyyed Mohammad Tabatabaei, Celine Tabche, Minale Tareke, Mohamad-Hani Temsah, Chern Choong Thum, Tenaw Yimer Tiruye, Marcos Roberto Tovani-Palone, Nghia Minh Tran, Nguyen Tran Minh Duc, Samuel Joseph Tromans, Guesh Mebrahtom Tsegay, Munkhtuya Tumurkhuu, Sanaz Vahdati, Pascual R Valdez, Massimiliano Veroux, Georgios-Ioannis Verras, Manish Vinayak, Theo Vos, Mandaras Tariku Walde, Yanzhong Wang, Joseph L L Ward, Nuwan Darshana Wickramasinghe, Renjulal Yesodharan, Arzu Yiğit, Dehui Yin, Paul Yip, Dong Keon Yon, Naohiro Yonemoto, Mohammed G M Zeariya, Haijun Zhang, Claire Chenwen Zhong, and Bin Zhu.

### Drafting the work or revising it critically for important intellectual content

Yohannes Habtegiorgis Abate, Habeeb Omoponle Adewuyi, Qorinah Estiningtyas Sakilah Adnani, Aanuoluwapo Adeyimika Afolabi, Suneth Buddhika Agampodi, Bright Opoku Ahinkorah, Danish Ahmad, Ayman Ahmed, Haroon Ahmed, Rasmieh Mustafa Al-amer, Mohammed Albashtawy, Wafa A Aldhaleei, Syed Shujait Ali, Waad Ali, Mohammed A Alsabri, Nelson Alvis-Guzman, Yaser Mohammed Al-Worafi, Alireza Amindarolzarbi, Sohrab Amiri, Saeid Anvari, Jalal Arabloo, Seyyed Shamsadin Athari, Maha Moh'd Wahbi Atout, Ahmed Y Azzam, Ashish D Badiye, Maciej Banach, Suzanne Lyn Barker-Collo, Till Winfried Bärnighausen, Amadou Barrow, Azadeh Bashiri, Hameed Akande Bashiru, Mohammad-Mahdi Bastan, Corina Benjet, Habib Benzian, Gregory J Bertolacci, Akshaya Srikanth Bhagavathula, Priyadarshini Bhattacharjee, Sri Harsha Boppana, Guilherme Borges, Hamed Borhany, Yasser Bustanji, Florentino Luciano Caetano dos Santos, Giulio Castelpietra, Arthur Caye, Muthia Cenderadewi, Rama Mohan Chandika, Eeshwar K Chandrasekar, Yifan Chen, Ritesh Chimoriya, Hitesh Chopra, Richard G Cowden, Koustuv Dalal, Samuel Demissie Darcho, Claudio Alberto Dávila-Cervantes, Nicole Davis Weaver, Alejandro de la Torre-Luque, Shayom Debopadhaya, Louisa Degenhardt, Ivan Delgado-Enciso, Emina Dervišević, Michael J Diaz, Deepa Dongarwar, Ojas Prakashbhai Doshi, Haneil Larson Dsouza, Samuel C Dumith, Senbagam Duraisamy, Michael Ekholuenetale, Rabie Adel El Arab, Muhammed Elhadi, Gihan ELNahas, Chadi Eltaha, Syed Emdadul Emdadul Haque, Sharareh Eskandarieh, Ayesha Fahim, Andre Faro, Ali Fatehizadeh, Nuno Ferreira, Florian Fischer, Ni Kadek Yuni Fridayani, Márió Gajdács, Balasankar Ganesan, Miglas Welay Gebregergis, Molla Getie, Delaram J Ghadimi, Ayman Grada, Michal Grivna, Shi-

Yang Guan, Snigdha Gulati, Sapna Gupta, Reyna Alma Gutiérrez, Roberth Steven Gutiérrez-Murillo, Nasrin Hanifi, Mahgol Sadat Hassan Zadeh Tabatabaei, Simon I Hay, Mehdi Hemmati, Nguyen Quoc Hoan, Mehdi Hosseinzadeh, Sorin Hostiuc, Junjie Huang, Hong-Han Huynh, Segun Emmanuel Ibitoye, Kevin S Ikuta, Olayinka Stephen Ilesanmi, Irena M Ilic, Milena D Ilic, Mustapha Immurana, Arit Inok, Chidozie Declan Iwu, Haitham Jahrami, Sanobar Jaka, Nitin Joseph, Charity Ehimwenma Joshua, Jacek Jerzy Jozwiak, Vidya Kadashetti, Kehinde Kazeem Kanmodi, Rami S Kantar, Neeti Kapoor, Shilpi Karmakar, Jessica A Kerr, Himanshu Khajuria, Ajmal Khan, Khaled Khatab, Khalid A Kheirallah, Kwanghyun Kim, Min Seo Kim, Shivakumar KM KM Shivakumar, Varun Krishna, Nuworza Kugbey, Mukhtar Kulimbet, Manasi Kumar, Iván Landires, Nhi Huu Hanh Le, Wei-Chen Lee, José Francisco López-Gil, Giancarlo Lucchetti, Zheng Feei Ma, Venkatesh Maled, Kashish Malhotra, Ahmad Azam Malik, Ramon Martinez-Piedra, Roy Rillera Marzo, Yasith Mathangasinghe, Pallab K Maulik, Hadush Negash Meles, Ritesh G Menezes, Tuomo J Meretoja, Tomislav Mestrovic, Irmina Maria Michalek, Ted R Miller, Moonis Mirza, Awoke Misganaw, Chaitanya Mittal, Madeline E Moberg, Abdalla Z Mohamed, Nouh Saad Mohamed, Abdollah Mohammadian-Hafshejani, Ali H Mokdad, Sabrina Molinaro, Lorenzo Monasta, AmirAli Moodi Ghalibaf, Shane Douglas Morrison, Faraz Mughal, Francesk Mulita, Yanjinlkham Munkhsaikhan, Christopher J L Murray, Sathish Muthu, Woojae Myung, Ayoub Nafei, Mohsen Naghavi, Zuhair S Natto, Shadan Navid, Biswa Prakash Nayak, Sudan Prasad Neupane, Hoang Anh Hoang Nguyen, Nhien Ngoc Y Nguyen, Phat Tuan Nguyen, Van Thanh Nguyen, Isabel Noguer, Chisom Adaobi Nri-Ezedi, Virginia Nuñez-Samudio, Ogochukwu Janet Nzoputam, Bogdan Oancea, In-Hwan Oh, Sylvester Reuben Okeke, Michal Ordak, Heather M Orpana, Esteban Ortiz-Prado, Uchechukwu Levi Osuagwu, Alicia Padron-Monedero, Jagadish Rao Padubidri, Raul Felipe Felipe Palma-Alvarez, Ashok Pandey, Ioannis Pantazopoulos, Jay Patel, Shrikant Pawar, Ionela-Roxana Petcu, Michael R Phillips, Zahra Zahid Piracha, Jalandhar Pradhan, Elton Junio Sady Prates, Jagadeesh Puvvula, Ibrahim Qattea, Gangzhen Qian, Venkatraman Radhakrishnan, Pankaja Raghav, Sarvenaz Rahimibarghani, Afarin Rahimi-Movaghar, Vafa Rahimi-Movaghar, Md. Mosfequr Rahman, Mohammad Rahmanian, Mahmoud Mohammed Ramadan, Shakthi Kumaran Ramasamy, Smitha Rani, Mithun Rao, Sowmya J Rao, Mohammad-Mahdi Rashidi, Devarajan Rathish, Jennifer Rickard, Leonardo Roever, Emily Rosenblad, Chandan S N, Basema Ahmad Saddik, Farideh Sadeghian, Umar Saeed, Sahar Saeedi Moghaddam, Mehdi Safari, Dominic Sagoe, Soumya Swaroop Sahoo, Dauda Salihu, Giovanni A Salum, Abdallah M Samy, Milena M Santric-Milicevic, Chinmoy Sarkar, Gargi Sachin Sarode, Sachin C Sarode, Mario Šekerija, Sadaf G Sepanlou, Mahan Shafie, Samiah Shahid, Amin Sharifan, Javad Sharifi Rad, Anupam Sharma, Vishal Sharma, Mahabalesh Shetty, Pavanchand H Shetty, Premalatha K Shetty, Velizar Shivarov, Paramdeep Singh, Puneetpal Singh, Surjit Singh, Bogdan Socea, Dan J Stein, Murray B Stein, Chandan Kumar Swain, Lukasz Szarpak, Sree Sudha T Y, Minale Tareke, Mohamad-Hani Temsah, Chern Choong Thum, Tenaw Yimer Tiruye, Marcos Roberto Tovani-Palone, Nghia Minh Tran, Thang Huu Tran, Nguyen Tran Minh Duc, Samuel Joseph Tromans, Thien Tan Tri Tai Truyen, Sanaz Vahdati, Asokan Govindaraj Vaithinathan, Tommi Juhani Vasankari, Massimiliano Veroux, Georgios-Ioannis Verras, Manish Vinayak, Mandaras Tariku Walde, Yanzhong Wang, Joseph L L Ward, Nuwan Darshana Wickramasinghe, Marcin W Wojewodzic, Arzu Yiğit, Paul Yip, Dong Keon Yon, Naohiro Yonemoto, Iman Zare, Mohammed G M Zeariya, Haijun Zhang, Claire Chenwen Zhong, and Bin Zhu.

### Managing the estimation or publications process

Nicole Davis Weaver, Erin B Hamilton, Simon I Hay, Ali H Mokdad, Christopher J L Murray, Mohsen Naghavi, Emily Rosenblad, and Eve E Wool.
